# Assessing the associations of 1,400 blood metabolites with major depressive disorder: a Mendelian randomization study

**DOI:** 10.3389/fpsyt.2024.1391535

**Published:** 2024-06-06

**Authors:** Tiantian Dong, Xingxin Wang, Zhixia Jia, Jiguo Yang, Yuanxiang Liu

**Affiliations:** ^1^ Center for External Treatment of Traditional Chinese Medicine, Affiliated Hospital of Shandong University of Traditional Chinese Medicine, Jinan, Shandong, China; ^2^ College of Acupuncture and Massage, Shandong University of Traditional Chinese Medicine, Jinan, Shandong, China; ^3^ College of First Clinical Medicine, Shandong University of Traditional Chinese Medicine, Jinan, China; ^4^ Department of Neurology, Affiliated Hospital of Shandong University of Traditional Chinese Medicine, Jinan, Shandong, China

**Keywords:** metabolites, major depressive disorder (MDD), Mendelian randomization study, pathways, metabolomics

## Abstract

**Background and objectives:**

Major Depressive Disorder (MDD) is one of the most prevalent and debilitating health conditions worldwide. Previous studies have reported a link between metabolic dysregulation and MDD. However, evidence for a causal relationship between blood metabolites and MDD is lacking.

**Methods:**

Using a two-sample bidirectional Mendelian randomization analysis (MR), we assessed the causal relationship between 1,400 serum metabolites and Major Depressive Disorder (MDD). The Inverse Variance Weighted method (IVW) was employed to estimate the causal association between exposures and outcomes. Additionally, MR-Egger regression, weighted median, simple mode, and weighted mode methods were used as supplementary approaches for a comprehensive appraisal of the causality between blood metabolites and MDD. Pleiotropy and heterogeneity tests were also conducted. Lastly, the relevant metabolites were subjected to metabolite function analysis, and a reverse MR was implemented to explore the potential influence of MDD on these metabolites.

**Results:**

After rigorous screening, we identified 34 known metabolites, 13 unknown metabolites, and 18 metabolite ratios associated with Major Depressive Disorder (MDD). Among all metabolites, 33 were found to have positive associations, and 32 had negative associations. The top five metabolites that increased the risk of MDD were the Arachidonate (20:4n6) to linoleate (18:2n6) ratio, LysoPE(18:0/0:0), N-acetyl-beta-alanine levels, Arachidonate (20:4n6) to oleate to vaccenate (18:1) ratio, Glutaminylglutamine, and Threonine to pyruvate ratio. Conversely, the top five metabolites that decreased the risk of MDD were N6-Acetyl-L-lysine, Oleoyl-linoleoyl-glycerol (18:1 to 18:2) [2] to linoleoyl-arachidonoyl-glycerol (18:2 to 20:4) [2] ratio, Methionine to phosphate ratio, Pregnanediol 3-O-glucuronide, and 6-Oxopiperidine-2-carboxylic acid. Metabolite function enrichment was primarily concentrated in pathways such as Bile Acid Biosynthesis (FDR=0.177), Glutathione Metabolism (FDR=0.177), Threonine, and 2-Oxobutanoate Degradation (FDR=0.177). In addition, enrichment was noted in pathways like Valine, Leucine, and Isoleucine Biosynthesis (p=0.04), as well as Ascorbate and Aldarate Metabolism (p=0.04).

**Discussion:**

Within a pool of 1,400 blood metabolites, we identified 34 known metabolites and 13 unknown metabolites, as well as 18 metabolite ratios associated with Major Depressive Disorder (MDD). Additionally, three functionally enriched groups and two metabolic pathways were selected. The integration of genomics and metabolomics has provided significant insights for the screening and prevention of MDD.

## 1 Introduction

Globally, Major Depressive Disorder (MDD) stands as one of the most common and incapacitating health concerns. In the past three decades, the worldwide incidence of MDD has surged by nearly 50%, impacting over 264 million individuals across various age groups (1). According to the World Health Organization, depression ranks as the foremost cause of mental and physical disability on a global scale and stands as a significant contributor to the burden of health issues faced worldwide (2). Regarding mental health challenges, Major Depressive Disorder (MDD) is regarded as one of the most critical conditions, necessitating immediate and proactive intervention (3).

Metabolites, which are the substrates and products of metabolism, not only drive essential cellular activities but also serve as functional intermediates that can predict or influence the onset and progression of diseases. In recent years, an increasing number of studies have been exploring the relationship between Major Depressive Disorder (MDD) and metabolomics, signaling a burgeoning interest in this area of research (4). These investigations have substantiated the effectiveness of metabolomics in unraveling the complex characteristics of depression, along with the molecular underpinnings associated with the clinical facets of Major Depressive Disorder (MDD). Significantly, metabolomics has illuminated the association between MDD and the gut microbiome and has further delineated the significance of lipids in the composition and operation of cellular membranes. The metabolic profile of Major Depressive Disorder (MDD) encompasses 124 metabolites, spanning across pathways of energy and lipid metabolism. The research identified 49 metabolites, including metabolites involved in the tricarboxylic acid (TCA) cycle, such as citrate and pyruvate. Notably, levels of citrate were significantly reduced, whereas pyruvate was markedly increased in patients with severe depression (5).

It is important to note that there is a paucity of cohort-based causality studies linking metabolites with Major Depressive Disorder (MDD). Understanding whether disparate abundance metabolites act as risk factors or protective agents for MDD would be meaningful for predicting the disease and aiding diagnosis through specific targeted approaches. Mendelian randomization (MR) analysis employs single nucleotide polymorphisms (SNPs), which occur randomly within human genes, as instrumental variables. This method is akin to the design of a randomized controlled trial (RCT), which enhances the randomization of the sample selection. By establishing a connection between exposure factors and outcome variables through instrumental variables, this method provides a more robust demonstration of the causal relationship between them. Moreover, since metabolic products can be either substances that influence the onset of a disease or substances that are produced as a result of the disease, bidirectional MR analysis can more effectively elucidate the causal direction between metabolic products and disease.

Therefore, this study collected a relatively complete set of serum metabolomics data and introduced a Mendelian Randomization (MR) analysis akin to the design of a randomized controlled trial (RCT). Through bidirectional MR validation, we elucidated the causal relationship between Major Depressive Disorder (MDD) and related metabolites. In this research, the MR approach utilized metabolite-associated SNPs as instrumental variables (IVs) to assess the causal effects of genetic proxy markers for metabolites and their ratios on MDD. Additionally, we evaluated integrated metabolic pathways to shed light on their biological mechanisms.

## 2 Methods

### 2.1 Study design

The dataset containing all the data from this study is made available to the public on the database website. Summarized statistical data from published genome-wide association studies (GWAS) have been included. Written informed consent for all subjects was obtained in separate studies, authorized by the Institutional Review Boards’ ethics committees. No additional ethical approval or informed consent is required.

We performed a two-sample Mendelian analysis using metabolites as exposure and MDD as outcome. Then the 65 metabolites selected were used as outcome, and MDD was used as exposure for reverse MR Analysis. Finally, the function analysis of the related metabolites was carried out.

In the current study, we conducted a comprehensive assessment of the relationship between Major Depressive Disorder (MDD) and metabolism based on a rigorous Mendelian Randomization (MR) design, evaluating a total of 1,091 metabolites and 309 metabolite ratios, encompassing 1,400 metabolic correlates. The clinical diagnosis of Major Depressive Disorder (MDD) relies on the use of the Patient Health Questionnaire-9 (PHQ-9). Kroenke et al (6) outlined the initial development of the PHQ-9 and proposed cutoff scores indicative of varying levels of depression severity, which were validated through the observation of disparities in health-related quality of life across adjacent severity categories. Consequently, the researchers employed 5-point intervals to delineate score ranges associated with varying levels of depression severity, with scores falling within the range of 20–27 indicative of severe depression (7).

**Figure 1:**
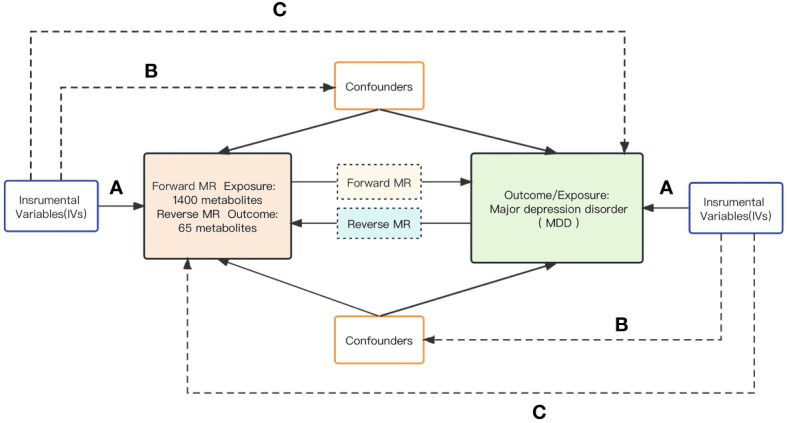
The schematic diagram of the bidirectional MR study.

Scientific MR studies must scrutinize three critical assumptions: A) The genetic instrumental variables must be strongly associated with the exposure of interest; B) The genetic instrumental variables should be independent of the outcome and not related to any known or unknown confounding factors; C) The effect of the instrumental variables on the outcome should be exclusively mediated through the exposure of interest. In summary, a bidirectional analytic strategy was employed to select genetic single nucleotide polymorphisms (SNPs) with significance to both 1,400 human serum metabolites and Major Depressive Disorder (MDD). To prevent sample overlap, the metabolite and MDD genetic information selected in this study was derived from separate GWAS datasets.The schematic illustration of this bidirectional MR study is shown in **Figure 1**.

### 2.2 Data sources

We conducted a series of large-scale Genome-Wide Association Studies (GWAS), which included metabolomic data of 1,091 metabolites and 309 metabolite ratios from a cohort of 8,299 individuals belonging to the Canadian Longitudinal Study on Aging (CLSA).

Chen et al. have obtained a comprehensive genome-wide association summary dataset for 1,091 metabolites and 309 metabolite ratios included in this study. This dataset is publicly accessible from the GWAS server (http://metabolomics.helmholtz-muenchen.de/gwas/). The service platform has gathered a relatively comprehensive set of human serum metabolomic data. The GWAS analysis included 1,091 metabolites and 309 metabolite ratios from 8,299 individuals, which are part of the Canadian Longitudinal Study on Aging (CLSA) cohort. Among the 1,091 plasma metabolites analyzed, 850 have known characteristics associated with eight super-pathways, namely lipids, amino acids, xenobiotics, nucleotides, cofactors and vitamins, carbohydrates, peptides, and energy. The remaining 241 were categorized as unknown or ‘partially’ characterized molecules. The current study features 96 metabolites that had not been tested in previous representative large-scale metabolomics GWASs. Detailed cohort characteristics and metabolite information can be found in **Supplementary Table 1**.

The summary data for Major Depressive Disorder come from the Integrated Epidemiology Unit (IEU) (https://gwas.mrcieu.ac.uk/) Open GWAS project. GWAS ID is ieu-b-102 (8). In this GWAS meta-analysis, the aggregate data comprise of 170,756 cases of Major Depressive Disorder (MDD) and 329,443 control subjects, yielding a total of 8,447,813 SNPs. We extracted the SNP information by analyzing the VCF format files shared through the platform.

### 2.3 Instrumental variable selection

In this MR (Mendelian Randomization) analysis, the selection of IVs (Instrumental Variables) was based on three core assumptions. Firstly, for each metabolite, we set a genome-wide significance threshold p < 1 × 10^−5 for the selection of strongly associated SNPs. Following the extraction of significant SNPs for each metabolite, we performed linkage disequilibrium analysis. Linkage disequilibrium was deemed present if the linkage disequilibrium coefficient (r^2) was less than 0.1 and the distance between SNPs fell within a 500-kilobase (kb) radius (9). Moreover, to mitigate the potential bias due to weak instrumentality, the F-statistic for each SNP was computed. SNPs exhibiting an F-statistic lower than 10 were deemed weak instruments and were subjected to further scrutiny (10).

### 2.4 Sensitivity analysis

The standard Inverse Variance Weighted (IVW) method (a random-effects model) (11) is the primary evaluative approach for investigating the causal relationship between metabolites and Major Depressive Disorder (MDD) in this analysis, encompassing both forward and reverse MR analyses. MR-Egger and the Weighted Median (WM) provide secondary methods of evaluation. When the instrumental variables satisfy all three core assumptions, the IVW method can estimate the causal effects of exposure with greater precision and is considered the most robust method for Mendelian Randomization. However, the analysis may yield inaccurate results if some of the instrumental variables do not adhere to the instrumental variable assumptions. Consequently, we conducted the following sensitivity analyses: 1) We performed Cochran’s Q test via the IVW and MR-Egger methods to detect potential violations of assumptions through heterogeneity in the correlations between individual IVs; 2) We implemented the MR-Egger intercept to estimate pleiotropy and ensure that genetic variations are independently associated with metabolites and MDD; 3) Additional analyses such as Weighted Median and Mode-based Estimation were used to enhance the reliability and stability of our hypothesis testing; 4) Individual SNP analyses and leave-one-out diagnostics were carried out to evaluate the likelihood of the observed associations for individual SNPs. MR analysis may breach causality assumptions under the premise of genetic correlation between exposure and study outcomes. While SNPs associated with MDD were excluded during IV selection, SNPs without known associations might also influence the incidence of MDD. Linkage Disequilibrium Score (LDSC) regression can assess pleiotropy by utilizing SNP-based chi-square statistics. Therefore, to ensure that the causal relationship is not confounded by coherence between exposure and outcome, we implemented LDSC to corroborate the genetic correlation between differential abundance of serum metabolites and MDD.

### 2.5 Statistical analysis

All MR analyses were carried out using the “TwoSampleMR” package within R (version 4.2.1). LDSC was conducted via the “ldsc” package, with a p-value of less than 0.05 considered to be statistically significant. Odds ratios (OR) were employed to estimate the magnitude and direction of the metabolic impact alongside their corresponding 95% confidence intervals (CIs).

### 2.6 Verification of genetic correlation and direction

Under the assumption of genetic correlation between exposures and study outcomes, MR analysis could violate the principles of causation. Although SNPs associated with MDD were excluded when selecting instrumental variables (IVs), non-associated SNPs might also influence the occurrence of MDD. Linkage disequilibrium score (LDSC) regression can calculate pleiotropy by invoking SNP-based chi-square statistics. Therefore, to ensure the causal inference is not confounded by the coherence between exposure and outcome, LDSC was employed to verify the genetic correlation between the differential abundance of serum metabolites and MDD.

### 2.7 Metabolic pathway analysis

Metabolic pathways were estimated using the web-based MetaboAnalyst 6.0 (https://www.Metaboanalyst.ca/). The Pathway Analysis and Enrichment Analysis modules were utilized to identify clusters of metabolites or super-pathways potentially associated with metabolic processes and their potential links to MDD. The human metabolome database (HMDB) and the Kyoto Encyclopedia of Genes and Genomes (KEGG) were used as reference databases. The significance level for pathways was set at 0.05.

## 3 Result

### 3.1 Step 1: The impact of 1400 serum metabolites on MDD

Due to the genome-wide significance threshold set at p < 1×10^-5 for selecting SNPs with strong associations, a total of 645 serum metabolites and metabolite ratios were chosen. All featured F-statistics values greater than 10, suggesting that a weak instrument bias is unlikely to be significant. All metabolic analyses employed the Inverse Variance Weighted (IVW) approach as the main method, with no evidence of heterogeneity or weak instruments. From the primary outcomes, 96 metabolites were significantly associated (with IVW p < 0.05). After screening for pleiotropy, 65 metabolites remained significantly associated (with IVW p < 0.05 and Pleiotropy p > 0.05), including 35 metabolites and 17 metabolite ratios.

**Figure 2:**
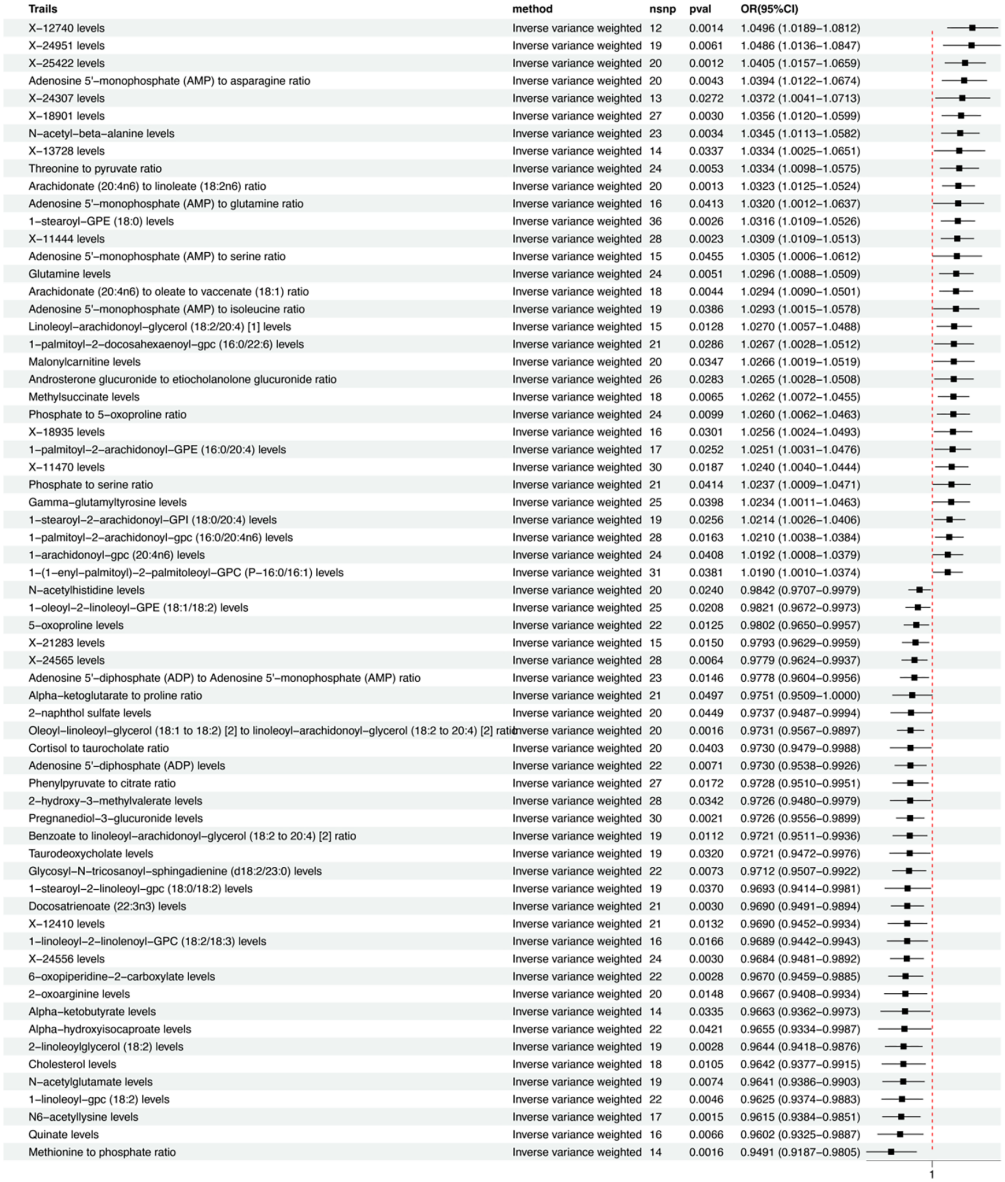
IVW forest maps of 65 significantly related metabolites.

Of the 13 metabolites, the chemical properties are unknown, while the remaining 35 identified metabolites fall into several categories including: Lipids, Carboxylic acids, Organooxygen compounds, amino acids, Keto acids, Naphthalenes, Purine nucleotides, Organic acids, Glycerophospholipids, Quinic acid, Fatty acids, Steroids, and Naphthalenes, among others. Notably, lipids are the most frequently occurring category, accounting for 60% of these identified substances. Within the group of 65 metabolites and metabolite ratios, 32 are positively associated with MDD, while 33 show a negative association with the condition. The IVW forest plot for the 65 significantly associated metabolites is presented in **Figure 2**. Alternate MR analyses, Q-tests, and sensitivity analysis results for the 65 known metabolites can be found in **Table 1**. All instrumental variables (IVs) passed the sensitivity tests (p > 0.05).

**Table 1:** Two MR Models estimated causality between 65 metabolites and their ratio and major depressive disorder, and tested for heterogeneity and level pleiotropy.

CLASS	Metabolite name and number	Method	SNP(n)	pval	OR	95%CI	Heterogeneity		Pleiotropy	
							Qvalue	P	intercept	P
Lipids	1-stearoyl-2-arachidonoyl-GPI (18:0/20:4) levels	MR Egger	19	0.220	1.03	0.99–1.07	14.36	0.641		
	GCST90199649	Inverse variance weighted	19	0.026	1.02	1–1.04	14.46	0.698	0.00	0.756
Lipids	Alpha-hydroxyisocaproate levels	MR Egger	22	0.820	0.99	0.91–1.08	33.89	0.027		
	GCST90199658	Inverse variance weighted	22	0.042	0.97	0.93–1	34.53	0.032	0.00	0.548
Lipids	2-linoleoylglycerol (18:2) levels	MR Egger	19	0.096	0.95	0.9–1.01	6.81	0.986		
	GCST90199685	Inverse variance weighted	19	0.003	0.96	0.94–0.99	7.10	0.989	0.00	0.598
Lipids	Docosatrienoate (22:3n3) levels	MR Egger	21	0.096	0.96	0.92–1	21.25	0.323		
	GCST90199710	Inverse variance weighted	21	0.003	0.97	0.95–0.99	21.48	0.370	0.00	0.657
Lipids	1-linoleoyl-gpc (18:2) levels	MR Egger	22	0.088	0.95	0.89–1.01	23.20	0.279		
	GCST90199742	Inverse variance weighted	22	0.005	0.96	0.94–0.99	23.63	0.311	0.00	0.549
Lipids	1-stearoyl-GPE (18:0) levels	MR Egger	36	0.422	1.02	0.97–1.08	41.79	0.168		
	GCST90199772	Inverse variance weighted	36	0.003	1.03	1.01–1.05	41.92	0.196	0.00	0.754
Lipids	Malonylcarnitine levels	MR Egger	20	0.472	1.02	0.96–1.09	21.48	0.256		
	GCST90199776	Inverse variance weighted	20	0.035	1.03	1–1.05	21.48	0.311	0.00	0.957
Lipids	2-hydroxy-3-methylvalerate levels	MR Egger	28	0.011	0.92	0.87–0.98	31.32	0.217		
	GCST90199786	Inverse variance weighted	28	0.034	0.97	0.95–1	36.17	0.112	0.01	0.055
Lipids	1-arachidonoyl-gpc (20:4n6) levels	MR Egger	24	0.043	1.03	1–1.06	37.47	0.021		
	GCST90199788	Inverse variance weighted	24	0.041	1.02	1–1.04	39.39	0.018	0.00	0.300
Lipids	Pregnanediol-3-glucuronide levels	MR Egger	30	0.037	0.96	0.92–1	30.21	0.353		
	GCST90199917	Inverse variance weighted	30	0.002	0.97	0.96–0.99	31.17	0.357	0.00	0.353
Lipids	1-stearoyl-2-linoleoyl-gpc (18:0/18:2) levels	MR Egger	19	0.359	0.97	0.9–1.04	29.75	0.028		
	GCST90200037	Inverse variance weighted	19	0.037	0.97	0.94–1	29.76	0.040	0.00	0.951
Lipids	1-palmitoyl-2-docosahexaenoyl-gpc (16:0/22:6) levels	MR Egger	21	0.091	1.07	0.99–1.14	21.53	0.308		
	GCST90200043	Inverse variance weighted	21	0.029	1.03	1–1.05	22.93	0.292	-0.01	0.280
Lipids	1-palmitoyl-2-arachidonoyl-GPE (16:0/20:4) levels	MR Egger	17	0.645	1.01	0.97–1.05	22.26	0.101		
	GCST90200054	Inverse variance weighted	17	0.025	1.03	1–1.05	23.40	0.103	0.00	0.395
Lipids	1-(1-enyl-palmitoyl)-2-palmitoleoyl-GPC (P-16:0/16:1) levels	MR Egger	31	0.146	1.03	0.99–1.07	38.52	0.111		
	GCST90200070	Inverse variance weighted	31	0.038	1.02	1–1.04	38.92	0.128	0.00	0.588
Lipids	1-oleoyl-2-linoleoyl-GPE (18:1/18:2) levels	MR Egger	25	0.037	0.97	0.94–1	25.82	0.310		
	GCST90200082	Inverse variance weighted	25	0.021	0.98	0.97–1	27.23	0.294	0.00	0.274
Lipids	1-linoleoyl-2-linolenoyl-GPC (18:2/18:3) levels	MR Egger	16	0.046	0.94	0.89–0.99	15.91	0.319		
	GCST90200095	Inverse variance weighted	16	0.017	0.97	0.94–0.99	17.62	0.283	0.00	0.240
Lipids	Linoleoyl-arachidonoyl-glycerol (18:2/20:4) [1] levels	MR Egger	15	0.077	1.04	1–1.08	16.44	0.226		
	GCST90200103	Inverse variance weighted	15	0.013	1.03	1.01–1.05	17.11	0.250	0.00	0.479
Lipids	Glycosyl-N-tricosanoyl-sphingadienine (d18:2/23:0) levels	MR Egger	22	0.563	0.98	0.94–1.04	20.76	0.411		
	GCST90200114	Inverse variance weighted	22	0.007	0.97	0.95–0.99	21.14	0.450	0.00	0.553
Lipids	Taurodeoxycholate levels	MR Egger	19	0.977	1.00	0.94–1.06	14.42	0.637		
	GCST90200334	Inverse variance weighted	19	0.032	0.97	0.95–1	15.54	0.625	0.00	0.305
Lipids	Methylsuccinate levels	MR Egger	18	0.004	1.05	1.02–1.09	11.42	0.783		
	GCST90200352	Inverse variance weighted	18	0.007	1.03	1.01–1.05	15.91	0.53	-0.01	0.05
Lipids	Cholesterol levels	MR Egger	18	0.167	0.96	0.91–1.01	10.01	0.866		
	GCST90200368	Inverse variance weighted	18	0.011	0.96	0.94–0.99	10.06	0.901	0	0.829
Lipids	1-palmitoyl-2-arachidonoyl-gpc (16:0/20:4n6) levels	MR Egger	28	0.115	1.02	1–1.05	46.92	0.007		
	GCST90200692	Inverse variance weighted	28	0.016	1.02	1–1.04	46.93	0.01	0	0.955
Amino acids	N-acetylglutamate levels	MR Egger	19	0.977	1.00	0.94–1.07	17.56	0.417		
	GCST90199637	Inverse variance weighted	19	0.007	0.96	0.94–0.99	18.99	0.392	0.00	0.256
Amino acids	N-acetylhistidine levels	MR Egger	20	0.039	0.98	0.96–1	18.02	0.454		
	GCST90199735	Inverse variance weighted	20	0.024	0.98	0.97–1	18.95	0.460	0.00	0.349
Amino acids	N6-acetyllysine levels	MR Egger	17	0.031	0.96	0.92–0.99	24.80	0.053		
	GCST90199826	Inverse variance weighted	17	0.002	0.96	0.94–0.99	25.08	0.068	0.00	0.690
Amino acids	6-oxopiperidine-2-carboxylate levels	MR Egger	22	0.075	0.97	0.93–1	27.22	0.129		
	GCST90199949	Inverse variance weighted	22	0.003	0.97	0.95–0.99	27.22	0.164	0.00	0.967
Amino acids	5-oxoproline levels	MR Egger	22	0.014	0.97	0.95–0.99	18.29	0.568		
	GCST90200280	Inverse variance weighted	22	0.013	0.98	0.96–1	20.07	0.517	0.00	0.198
Amino acids	Gamma-glutamyltyrosine levels	MR Egger	25	0.492	1.02	0.97–1.07	13.91	0.930		
	GCST90200295	Inverse variance weighted	25	0.040	1.02	1–1.05	14.00	0.947	0.00	0.761
Amino acids	Glutamine levels	MR Egger	24	0.045	1.05	1–1.09	22.3	0.442		
	GCST90200419	Inverse variance weighted	24	0.005	1.03	1.01–1.05	23.11	0.454	0	0.379
Keto acids	2-oxoarginine levels	MR Egger	20	0.699	0.98	0.91–1.07	21.38	0.261		
	GCST90199903	Inverse variance weighted	20	0.015	0.97	0.94–0.99	21.60	0.305	0.00	0.668
Keto acids	Alpha-ketobutyrate levels	MR Egger	14	0.224	0.96	0.89–1.02	7.51	0.822		
	GCST90200438	Inverse variance weighted	14	0.034	0.97	0.94–1	7.62	0.867	0	0.746
Quinic acid	Quinate levels	MR Egger	16	0.359	0.97	0.92–1.03	12.45	0.570		
	GCST90199645	Inverse variance weighted	16	0.007	0.96	0.93–0.99	12.67	0.628	0.00	0.649
Carboxylic acids	N-acetyl-beta-alanine levels	MR Egger	23	0.059	1.04	1–1.09	14.03	0.868		
	GCST90199866	Inverse variance weighted	23	0.003	1.03	1.01–1.06	14.31	0.890	0.00	0.606
Naphthalenes	2-naphthol sulfate levels	MR Egger	20	0.061	0.94	0.89–1	14.18	0.717		
	GCST90200213	Inverse variance weighted	20	0.045	0.97	0.95–1	15.65	0.680	0.00	0.241
Purine nucleotides	Adenosine 5’-diphosphate (ADP) levels	MR Egger	22	0.106	0.96	0.91–1.01	22.69	0.304		
	GCST90200355	Inverse variance weighted	22	0.007	0.97	0.95–0.99	23.26	0.33	0	0.487
Ratio	Adenosine 5’-diphosphate (ADP) to Adenosine 5’-monophosphate (AMP) ratio	MR Egger	23	0.411	0.98	0.94–1.03	14.22	0.86		
	GCST90200728	Inverse variance weighted	23	0.015	0.98	0.96–1	14.24	0.893	0	0.888
Ratio	Arachidonate (20:4n6) to oleate to vaccenate (18:1) ratio	MR Egger	18	0.071	1.03	1–1.07	25.37	0.063		
	GCST90200740	Inverse variance weighted	18	0.004	1.03	1.01–1.05	25.47	0.085	0	0.811
Ratio	Oleoyl-linoleoyl-glycerol (18:1 to 18:2) [2] to linoleoyl-arachidonoyl-glycerol (18:2 to 20:4) [2] ratio	MR Egger	20	0.003	0.96	0.93–0.98	20.07	0.329		
	GCST90200795	Inverse variance weighted	20	0.002	0.97	0.96–0.99	23.31	0.224	0	0.105
Ratio	Adenosine 5’-monophosphate (AMP) to glutamine ratio	MR Egger	16	0.056	1.07	1–1.14	8.18	0.88		
	GCST90200848	Inverse variance weighted	16	0.041	1.03	1–1.06	9.73	0.837	0	0.234
Ratio	Adenosine 5’-monophosphate (AMP) to asparagine ratio	MR Egger	20	0.085	1.06	1–1.13	9.33	0.952		
	GCST90200859	Inverse variance weighted	20	0.004	1.04	1.01–1.07	9.83	0.957	0	0.49
Ratio	Adenosine 5’-monophosphate (AMP) to serine ratio	MR Egger	15	0.350	1.04	0.96–1.11	11.82	0.543		
	GCST90200860	Inverse variance weighted	15	0.046	1.03	1–1.06	11.85	0.619	0	0.867
Ratio	Phosphate to serine ratio	MR Egger	21	0.160	1.05	0.98–1.11	14.85	0.732		
	GCST90200863	Inverse variance weighted	21	0.041	1.02	1–1.05	15.41	0.753	0	0.464
Ratio	Methionine to phosphate ratio	MR Egger	14	0.364	0.97	0.9–1.04	11.95	0.449		
	GCST90200864	Inverse variance weighted	14	0.002	0.95	0.92–0.98	12.27	0.506	0	0.586
Ratio	Adenosine 5’-monophosphate (AMP) to isoleucine ratio	MR Egger	19	0.041	1.09	1.01–1.17	13.9	0.674		
	GCST90200867	Inverse variance weighted	19	0.039	1.03	1–1.06	16.3	0.572	-0.01	0.14
Ratio	Phenylpyruvate to citrate ratio	MR Egger	27	0.118	0.96	0.91–1.01	17.16	0.876		
	GCST90200885	Inverse variance weighted	27	0.017	0.97	0.95–1	17.61	0.889	0	0.506
Ratio	Cortisol to taurocholate ratio	MR Egger	20	0.364	0.98	0.93–1.03	20.07	0.329		
	GCST90200890	Inverse variance weighted	20	0.040	0.97	0.95–1	20.09	0.389	0	0.896
Ratio	Alpha-ketoglutarate to proline ratio	MR Egger	21	0.313	0.97	0.91–1.03	18.23	0.507		
	GCST90200933	Inverse variance weighted	21	0.050	0.98	0.95–1	18.29	0.568	0	0.809
Ratio	Phosphate to 5-oxoproline ratio	MR Egger	24	0.194	1.02	0.99–1.05	29.15	0.141		
	GCST90200968	Inverse variance weighted	24	0.010	1.03	1.01–1.05	29.52	0.164	0	0.602
Ratio	Arachidonate (20:4n6) to linoleate (18:2n6) ratio	MR Egger	20	0.041	1.04	1–1.07	24.95	0.126		
	GCST90200979	Inverse variance weighted	20	0.001	1.03	1.01–1.05	25.08	0.158	0	0.761
Ratio	Benzoate to linoleoyl-arachidonoyl-glycerol (18:2 to 20:4) [2] ratio	MR Egger	19	0.067	0.96	0.91–1	23.01	0.149		
	GCST90200990	Inverse variance weighted	19	0.011	0.97	0.95–0.99	23.96	0.156	0	0.412
Ratio	Threonine to pyruvate ratio	MR Egger	24	0.459	1.02	0.97–1.08	22.88	0.408		
	GCST90201009	Inverse variance weighted	24	0.005	1.03	1.01–1.06	23.12	0.454	0	0.639
Ratio	Androsterone glucuronide to etiocholanolone glucuronide ratio	MR Egger	26	0.045	1.04	1–1.08	52.33	0.001		
	GCST90201013	Inverse variance weighted	26	0.028	1.03	1–1.05	53.99	0.001	0	0.391
Unknown	X-11470 levels	MR Egger	30	0.177	1.02	0.99–1.05	41.08	0.053		
	GCST90200470	Inverse variance weighted	30	0.019	1.02	1–1.04	41.12	0.067	0	0.867
Unknown	X-11444 levels	MR Egger	28	0.013	1.04	1.01–1.07	32.99	0.162		
	GCST90200474	Inverse variance weighted	28	0.002	1.03	1.01–1.05	34.08	0.164	0	0.361
Unknown	X-12410 levels	MR Egger	21	0.201	0.97	0.92–1.02	28.18	0.08		
	GCST90200480	Inverse variance weighted	21	0.013	0.97	0.95–0.99	28.19	0.105	0	0.919
Unknown	X-12740 levels	MR Egger	12	0.394	1.04	0.96–1.12	9.6	0.476		
	GCST90200497	Inverse variance weighted	12	0.001	1.05	1.02–1.08	9.71	0.556	0	0.74
Unknown	X-13728 levels	MR Egger	14	0.459	1.02	0.96–1.09	9.63	0.649		
	GCST90200522	Inverse variance weighted	14	0.034	1.03	1–1.07	9.71	0.717	0	0.778
Unknown	X-18901 levels	MR Egger	27	0.111	1.04	0.99–1.08	13.77	0.966		
	GCST90200559	Inverse variance weighted	27	0.003	1.04	1.01–1.06	13.77	0.976	0	0.979
Unknown	X-18935 levels	MR Egger	16	0.939	1	0.94–1.07	11.07	0.681		
	GCST90200573	Inverse variance weighted	16	0.030	1.03	1–1.05	11.6	0.709	0	0.477
Unknown	X-21283 levels	MR Egger	15	0.046	0.97	0.95–1	9.68	0.72		
	GCST90200575	Inverse variance weighted	15	0.015	0.98	0.96–1	10.19	0.748	0	0.488
Unknown	X-24556 levels	MR Egger	24	0.800	0.99	0.95–1.04	25.37	0.28		
	GCST90200628	Inverse variance weighted	24	0.003	0.97	0.95–0.99	26.86	0.262	0	0.269
Unknown	X-24307 levels	MR Egger	13	0.509	1.03	0.95–1.12	8.14	0.701		
	GCST90200632	Inverse variance weighted	13	0.027	1.04	1–1.07	8.18	0.771	0	0.837
Unknown	X-24951 levels	MR Egger	19	0.151	1.06	0.98–1.15	25.65	0.081		
	GCST90200643	Inverse variance weighted	19	0.006	1.05	1.01–1.08	25.82	0.104	0	0.743
Unknown	X-24565 levels	MR Egger	28	0.177	0.98	0.95–1.01	18.39	0.861		
	GCST90200645	Inverse variance weighted	28	0.006	0.98	0.96–0.99	18.39	0.891	0	0.98
Unknown	X-25422 levels	MR Egger	20	0.105	1.05	0.99–1.11	22.61	0.206		
	GCST90200661	Inverse variance weighted	20	0.001	1.04	1.02–1.07	22.72	0.25	0	0.777

Among the 34 identified metabolites, we found that N6-acetyllysine levels have the most significant negative correlation with MDD (IVW OR = 0.96; 95% CI = 0.92–0.99; P = 0.0015), followed by Pregnanediol-3-glucuronide levels (IVW OR = 0.97; 95% CI = 0.96–0.99; P = 0.002); 6-oxopiperidine-2-carboxylate levels (IVW OR = 0.97; 95% CI = 0.95–0.99; P = 0.003); 2-linoleoylglycerol (18:2) levels (IVW OR = 0.96; 95% CI = 0.94–0.99; P = 0.003); Docosatrienoate (22:3n3) levels (IVW OR = 0.97; 95% CI = 0.95–0.99; P = 0.003); 1-linoleoyl-gpc (18:2) levels (IVW OR = 0.96; 95% CI = 0.94–0.99; P = 0.005); Quinate levels (IVW OR = 0.96; 95% CI = 0.93–0.99; P = 0.007); Adenosine 5’-diphosphate (ADP) levels (IVW OR = 0.97; 95% CI = 0.95–0.99; P = 0.007); and Taurodeoxycholate levels (IVW OR = 0.97; 95% CI = 0.95–1; P = 0.032).

The most significantly positive correlation with MDD was observed in the levels of 1-stearoyl-GPE (18:0) (IVW OR = 1.03; 95% CI = 1.01–1.05; P = 0.003); followed by N-acetyl-beta-alanine levels (IVW OR = 1.03; 95% CI = 1.01–1.06; P = 0.003); Glutamine levels (IVW OR = 1.03; 95% CI = 1.01–1.05; P = 0.005); Methylsuccinate levels (IVW OR = 1.03; 95% CI = 1.01–1.05; P = 0.007); Linoleoyl-arachidonoyl-glycerol (18:2/20:4) [1] levels (IVW OR = 1.03; 95% CI = 1.01–1.05; P = 0.013); and 1-palmitoyl-2-arachidonoyl-gpc (16:0/20:4n6) levels (IVW OR = 1.02; 95% CI = 1–1.04; P = 0.016).

In terms of metabolite ratios, the most significant positive correlation with MDD was observed in the ratio of Arachidonate (20:4n6) to Linoleate (18:2n6) (IVW OR = 1.03; 95% CI = 1.01–1.05; P = 0.001); the most significant negative correlation with MDD was found in the ratio of Oleoyl-linoleoyl-glycerol (18:1 to 18:2) [2] to Linoleoyl-arachidonoyl-glycerol (18:2 to 20:4) [2] (IVW OR = 0.97; 95% CI = 0.96–0.99; P = 0.002).

In summary, MR estimates from IVW, WM, and MR-Egger regression for 34 metabolites and 18 metabolite ratios showed consistent direction and magnitude, supporting robustness in the causal inference, except for Alpha-hydroxyisocaproate levels (IVW OR = 0.97; 95% CI = 0.93–1; P = 0.042; heterogeneity Q value = 34.53; P = 0.032); 1-arachidonoyl-gpc (20:4n6) levels (IVW OR = 1.02; 95% CI = 1–1.04; P = 0.041; heterogeneity Q value = 39.39; P = 0.018); 1-stearoyl-2-linoleoyl-gpc (18:0/18:2) levels (IVW OR = 0.97; 95% CI = 0.94–1; P = 0.037; heterogeneity Q value = 29.76; P = 0.040); 1-palmitoyl-2-arachidonoyl-gpc (16:0/20:4n6) levels (IVW OR = 1.02; 95% CI = 1–1.04; P = 0.016; heterogeneity Q value = 46.93; P = 0.01); and the Androsterone glucuronide to etiocholanolone glucuronide ratio (IVW OR = 1.03; 95% CI = 1–1.05; P = 0.028; heterogeneity Q value = 53.99; P = 0.001). Heterogeneity was not detected in the p-values of the Cochran Q test for the remaining metabolites and metabolite ratios (**Table 1**; **Supplementary Tables 1**, **2**). The MR-Egger intercept did not indicate the presence of pleiotropy (**Table 1**; **Supplementary Tables 1**, **2**). Furthermore, a Leave-One-Out (LOO) analysis did not identify any highly influential SNPs that could bias the combined effect estimates (**Supplementary Table 3**). Therefore, these 34 metabolites and 18 metabolite ratios have been identified as potential candidate metabolomic markers involved in the pathogenesis of major depressive disorder (MDD) and warrant further analysis, with particular focus on the levels of N6-acetyllysine and 1-stearoyl-GPE (18:0).

### 3.2 Step 2: The impact of MDD on the inverse MR Of 65 metabolites and metabolite ratios

Given a genome-wide significance threshold of p < 1 × 10^−5, fifty significant SNPs were extracted to serve as instrumental variables (IVs) for major depressive disorder (MDD) (**Supplementary Table 4**). The analysis utilized 65 serum metabolites and metabolite ratios as outcomes. Moreover, the F-statistics were all well above 10, suggesting that a bias from weak instruments is unlikely to be significant. The Inverse Variance Weighted (IVW) method was employed as the primary estimation approach for MDD. Of the principal findings, only one metabolite, X-12740 levels, was identified as significantly associated (IVW OR = 1.21; 95% CI = 1.00–1.47; P = 0.045; heterogeneity Q value = 27.79; P = 0.980); however, since this metabolite is unidentified, it lacks research significance. Consequently, it is evident that the influence of the 34 metabolites and 18 metabolite ratios on MDD is singular.

### 3.3 Step 3: Metabolic pathway analysis

**Figure 3:**
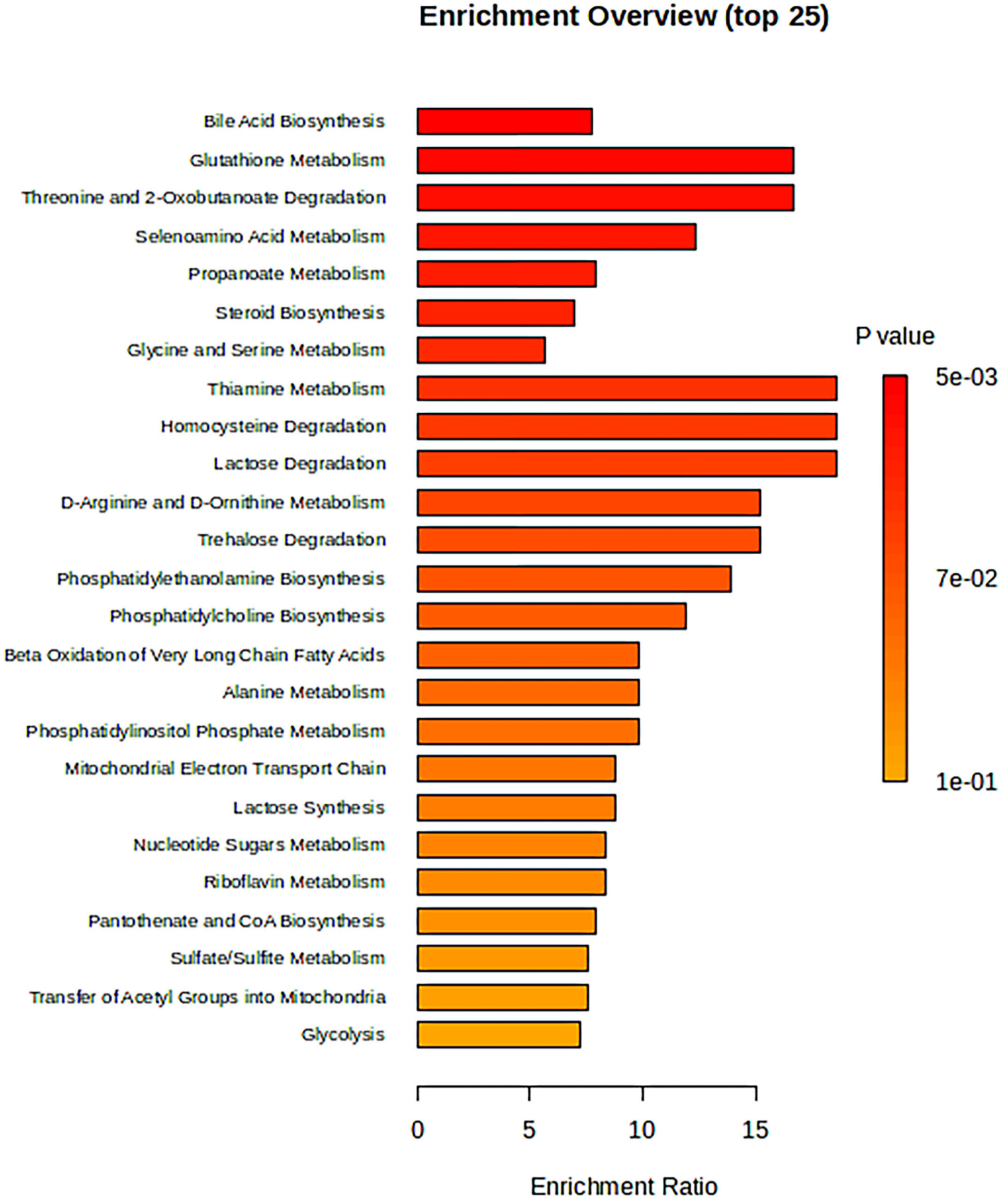
The enrichment overview (top 25).

**Figure 4:**
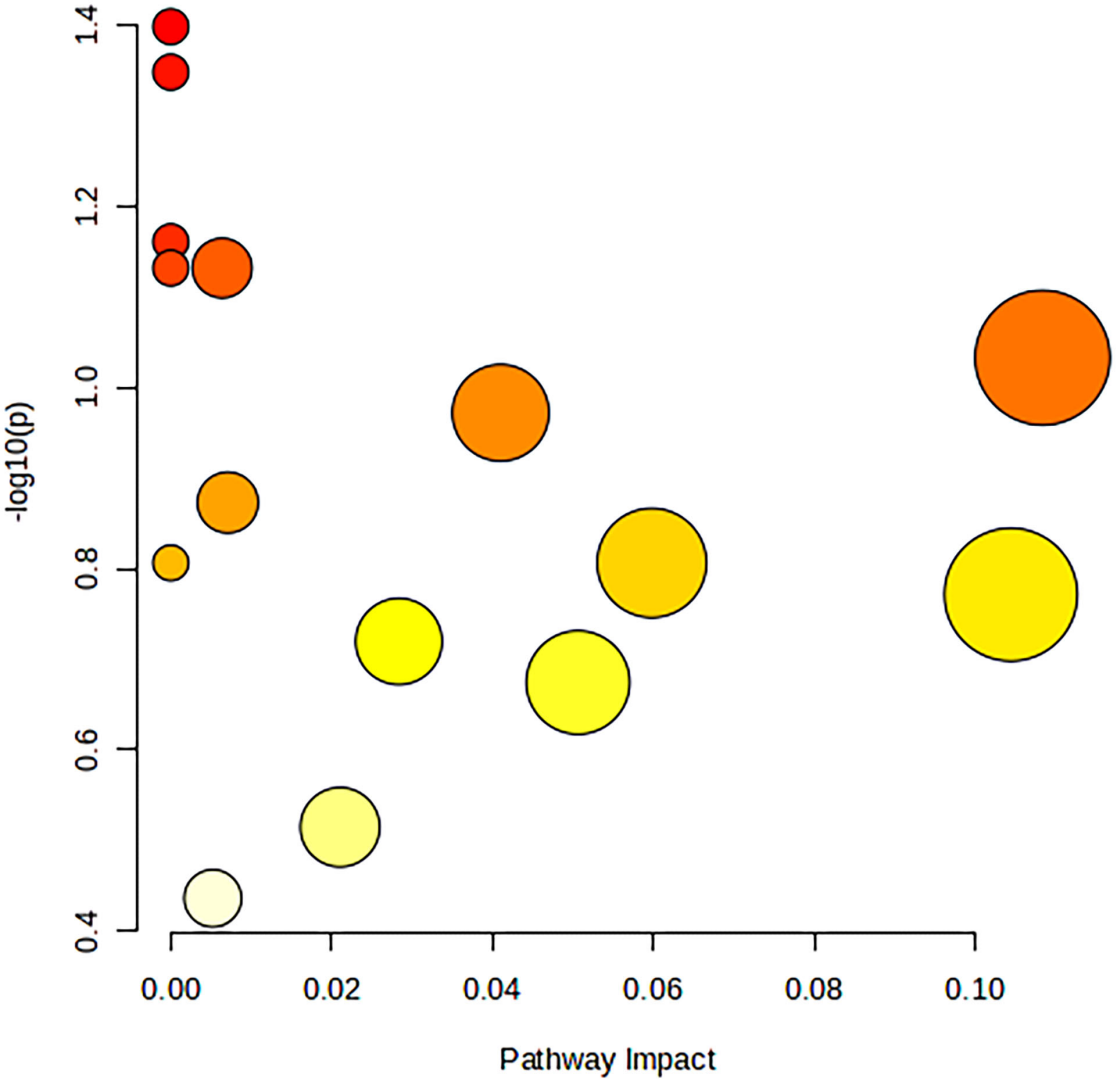
Image of the first ten bubbles in MR Enrichment pathway.

The 34 metabolites significantly associated with MDD were inputted into the MetaboAnalyst 6.0 platform to identify various potential metabolic pathways involved in the pathogenesis and immunology of MDD. Among them, Bile Acid Biosynthesis (FDR = 0.177), Glutathione Metabolism (FDR = 0.177), and Threonine and 2-Oxobutanoate Degradation (FDR = 0.177) demonstrated notable functional enrichment (**Figure 3**). Additionally, the pathways of Valine, Leucine and Isoleucine Biosynthesis (p = 0.04), and Ascorbate and Aldarate Metabolism (p = 0.04) were even more significant (**Table 2**, **Figure 4**). The metabolic mechanisms formed by these metabolites may be related to the pathogenesis affected by MDD. **Figure 3** illustrates the Enrichment Overview(top 25). **Table 2** and **Figure 4** show the top ten enrichment pathways.

**Table 2:** MR Enrichment pathway top ten table.

Pathway	Total	Expected	Hits	Raw p	FDR	-LOG10(p)	Impact
Valine, leucine and isoleucine biosynthesis	8	0.040635	1	0.040007	1	1.3979	0
Ascorbate and aldarate metabolism	9	0.045714	1	0.044908	1	1.3477	0
Arginine biosynthesis	14	0.071111	1	0.069087	1	1.1606	0
D-Amino acid metabolism	15	0.07619	1	0.073857	1	1.1316	0
Glycosylphosphatidylinositol (GPI)-anchor biosynthesis	15	0.07619	1	0.073857	1	1.1316	0.00639
Pentose and glucuronate interconversions	19	0.096508	1	0.092728	1	1.0328	0.10843
Propanoate metabolism	22	0.11175	1	0.10666	1	0.972	0.04103
Glutathione metabolism	28	0.14222	1	0.13396	1	0.87302	0.00709
Glycine, serine and threonine metabolism	33	0.16762	1	0.15615	1	0.80646	0
Cysteine and methionine metabolism	33	0.16762	1	0.15615	1	0.80646	0.05983

**Figure 4** is a figure in **Table 2**, with Impact as the horizontal coordinate and -LOG10(p) as the vertical coordinate. The pathway marked in the figure above is the significant pathway (Raw p&lt; 0.05).

## 4 Discussion

Our research findings confirm a causal relationship between 34 metabolites and 18 metabolite ratios with Major Depressive Disorder (MDD). Specifically, increased levels of 13 metabolites including 1-stearoyl-2-arachidonoyl-GPI (18:0/20:4), 1-stearoyl-GPE (18:0), Malonylcarnitine, 1-arachidonoyl-gpc (20:4n6), 1-palmitoyl-2-docosahexaenoyl-gpc (16:0/22:6), 1-palmitoyl-2-arachidonoyl-GPE (16:0/20:4), 1-(1-enyl-palmitoyl)-2-palmitoleoyl-GPC (P-16:0/16:1), Linoleoyl-arachidonoyl-glycerol (18:2/20:4), Methylsuccinate, 1-palmitoyl-2-arachidonoyl-gpc (16:0/20:4n6), Gamma-glutamyltyrosine, Glutamine, N-acetyl-beta-alanine and an increase in 7 metabolite ratios including Arachidonate (20:4n6) to oleate to vaccenate (18:1) ratio, Adenosine 5’-monophosphate (AMP) to glutamine ratio, AMP to asparagine ratio, AMP to serine ratio, Phosphate to serine ratio, AMP to isoleucine ratio, Phosphate to 5-oxoproline ratio, Arachidonate (20:4n6) to linoleate (18:2n6) ratio, and Threonine to pyruvate ratio, Androsterone glucuronide to etiocholanolone glucuronide ratio, are associated with adverse effects on MDD.

Conversely, the elevation of 22 other metabolites including Alpha-hydroxyisocaproate, 2-linoleoylglycerol (18:2), Docosatrienoate (22:3n3), 1-linoleoyl-gpc (18:2), 2-hydroxy-3-methylvalerate, Pregnanediol-3-glucuronide, 1-stearoyl-2-linoleoyl-gpc (18:0/18:2), 1-oleoyl-2-linoleoyl-GPE (18:1/18:2), 1-linoleoyl-2-linolenoyl-GPC (18:2/18:3), Glycosyl-N-tricosanoyl-sphingadienine (d18:2/23:0), Taurodeoxycholate, Cholesterol, N-acetylglutamate, N-acetylhistidine, N6-acetyllysine, 6-oxopiperidine-2-carboxylate, 5-oxoproline, 2-oxoarginine, Alpha-ketobutyrate, Quinate, 2-naphthol sulfate, Adenosine 5’-diphosphate (ADP), along with an increase in 7 additional metabolite ratios, including ADP to AMP ratio, Oleoyl-linoleoyl-glycerol (18:1 to 18:2) [2] to linoleoyl-arachidonoyl-glycerol (18:2 to 20:4) [2] ratio, Methionine to phosphate ratio, Phenylpyruvate to citrate ratio, Cortisol to taurocholate ratio, Alpha-ketoglutarate to proline ratio, Benzoate to linoleoyl-arachidonoyl-glycerol (18:2 to 20:4) [2] ratio, serve a protective role in the onset of MDD. Among the findings, the blood metabolite N6-acetyllysine displayed the most significant correlation with Major Depressive Disorder (MDD). Additionally, our research identified that three functionally enriched pathways: Bile Acid Biosynthesis (FDR = 0.177), Glutathione Metabolism (FDR = 0.177), and Threonine and 2-Oxobutanoate Degradation (FDR = 0.177), along with two metabolic pathways: Valine, Leucine and Isoleucine Biosynthesis (p = 0.04), and Ascorbate and Aldarate Metabolism (p = 0.04), play a crucial role in the progression of MDD.

Major Depressive Disorder (MDD) is a significant factor impacting people’s health and affects the entire lifespan of individuals. It is associated with a plethora of debilitating symptoms beyond mood dysregulation, ranging from cognitive and motor dysfunctions to autonomic nervous symptoms, as well as an increased risk of inflammation, immune system disruption, cardiovascular diseases, and heightened mortality. While most antidepressant medications target the monoaminergic pathways, mounting evidence suggests a more intricate interplay involving multiple pathways, reflecting extensive metabolic alterations, including those in energy and lipid metabolism. Changes in lipids, such as triglycerides, low and very-low-density lipoproteins (LDLs and VLDLs), high-density lipoproteins (HDLs), phosphatidylcholine, lysophosphatidylcholine, and sphingomyelins, have been observed. A recent study involving 5,283 MDD patients and 10,145 controls has indicated that individuals with MDD have reduced levels of HDLs and elevated levels of VLDLs and triglycerides. This indicates that metabolites, particularly lipids, play a crucial role in the onset and progression of MDD (5). To our knowledge, this is the first study utilizing a Mendelian Randomization (MR) approach in order to evaluate the causal relationship between 1,400 blood metabolites and metabolite ratios and the risk of Major Depressive Disorder (MDD).

### 4.1 Adverse reactions of metabolites to MDD

After excluding unknown metabolites and metabolite ratios, 13 metabolites were found to have adverse effects on Major Depressive Disorder (MDD), including: 1-stearoyl-GPE (18:0), N-acetyl-beta-alanine, Glutamine, Methylsuccinate, Linoleoyl-arachidonoyl-glycerol (18:2/20:4), 1-palmitoyl-2-arachidonoyl-gpc (16:0/20:4n6), 1-palmitoyl-2-arachidonoyl-GPE (16:0/20:4), 1-stearoyl-2-arachidonoyl-GPI (18:0/20:4), 1-palmitoyl-2-docosahexaenoyl-gpc (16:0/22:6), Malonylcarnitine, 1-(1-enyl-palmitoyl)-2-palmitoleoyl-GPC (P-16:0/16:1), Gamma-glutamyltyrosine, and 1-arachidonoyl-gpc (20:4n6). 1-stearoyl-GPE (18:0), also known as LysoPE(18:0/0:0), is a type of lysophospholipid. Lysophosphatidylcholines (lysoPCs) and lysophosphatidylethanolamines (lysoPEs) are categorized as glycerophospholipids, which are the metabolic products of cell membranes. Research indicates that lysoPCs and lysoPEs can be interconverted by the action of phospholipase A2 and phosphatidylethanolamine N-methyltransferase. Furthermore, LysoPC(0:0/18:0) may serve as a sensitive potential biomarker for hepatocyte death induced by chronic exposure to chlorpyrifos in Wistar rats (12, 13). Strikingly, the findings of Zhang et al. revealed a negative correlation between lysoPE (0:0/18:0) and the increased expression of genes related to Hmgcs1, Acat2, and Apoa4. This interaction suggests that lysoPE (0:0/18:0) may disrupt metabolic pathways, including the synthesis and degradation of ketone bodies, fat digestion and absorption, butanoate metabolism, and the degradation of the branched-chain amino acids valine, leucine, and isoleucine, following exposure to PFOS. Consequently, they postulated that reduced liver levels of lysoPE (0:0/18:0) in offspring exposed to PFOS could potentially elevate hepatotoxicity (14). Linoleoyl-arachidonoyl-glycerol (18:2/20:4), also known as PC(18:2(9Z,12Z)/20:4(5Z,8Z,11Z,14Z)), and 1-palmitoyl-2-docosahexaenoyl-gpc (16:0/22:6), referred to as PC(38:6), are types of phosphatidylcholines that are closely associated with breast cancer, cervical cancer, ulcerative colitis, and atherosclerosis. Studies have uncovered a relationship between the gut microbiota-dependent metabolism of dietary phosphatidylcholine and the pathogenesis of cardiovascular diseases (15). 1-palmitoyl-2-arachidonoyl-gpc (16:0/20:4n6), also known as PC(16:0/20:4(5Z,8Z,11Z,14Z)), along with 1-arachidonoyl-gpc (20:4n6), are currently under-researched. 1-palmitoyl-2-arachidonoyl-GPE (16:0/20:4)(PE(16:0/20:4(5Z,8Z,11Z,14Z))) is a phosphatidylethanolamine that has recently been recognized for its significant role in mammalian health following its association with diseases such as Alzheimer’s, Parkinson’s, non-alcoholic liver disease, and the virulence of certain pathogens through the discovery of its metabolic importance (16). 1-stearoyl-2-arachidonoyl-GPI (18:0/20:4), also known as PI(18:0/20:4(5Z,8Z,11Z,14Z)), is a phosphatidylinositol, which is an essential lipid playing dual roles as a crucial membrane component and as a participant in fundamental metabolic processes. Phosphoinositides (PI) comprise only a small fraction of the total cellular phospholipid content, yet they have a significant role in the development and progression of cancer. In various types of cancer, specific phosphoinositides such as phosphatidylinositol 3,4,5-trisphosphate [PtdIns(3,4,5)P3] and phosphatidylinositol 4,5-bisphosphate [PtdIns(4,5)P2] are critically involved in the regulation of survival, proliferation, invasion, and growth of cancer cells (17). Furthermore, they are closely associated with conditions such as Alzheimer’s disease, epileptic seizures, and Parkinson’s disease (18).

N-Acetyl-beta-alanine, also known as 3-(acetylamino)propionic acid, falls into the category of organic compounds known as carboxylic acids. It is an endogenous β amino acid that is metabolized into acetate and β alanine by the enzyme N-acetyl-β alanine dehydrogenase. β-alanine serves as an intermediate molecule between GABA (gamma-aminobutyric acid) and glycine, exhibiting a mechanism of action that is very similar to these neurotransmitters. It is considered to be an inhibitory neurotransmitter. Studies have shown that metabolomic analysis of the prefrontal cortex in mice indicates an upregulation of N-acetyl-beta-alanine during wakefulness compared to sleep, suggesting a potential link with sleep regulation (19). It is well-established that there is a close correlation between sleep and major depressive disorder. Studies support the notion that an early bedtime is associated with a protective effect against the risk of developing major depressive disorder (20, 21).

Therefore, whether N-acetyl-beta-alanine could be a metabolic product associated with the link between sleep and major depressive disorder (MDD) merits further investigation. Glutaminylglutamine, which belongs to the class of organic compounds known as dipeptides, as well as Methylsuccinic, Malonylcarnitine, and gamma-Glutamyltyrosine, have been minimally studied in relation to MDD.

### 4.2 Positive effects of metabolites on MDD

After excluding unknown metabolites and their ratios, there are 22 metabolites identified that have an adverse impact on Major Depressive Disorder (MDD), including: N6-Acetyl-L-lysine, Pregnanediol 3-O-glucuronide, 6-Oxopiperidine-2-carboxylic acid, MG(0:0/18:2(9Z,12Z)/0:0), Docosatrienoic acid, LysoPC(18:2/0:0), Quinic acid, ADP, SM(d18:2(4E,14Z)/23:0), N-Acetyl-L-glutamic acid, Cholesterol, Pyroglutamic acid, 2-Oxoarginine, PC(18:2(9Z,12Z)/18:3(6Z,9Z,12Z)), PE(18:1(9Z)/18:2(9Z,12Z)), N-Acetylhistidine, Taurodeoxycholic acid, 2-Ketobutyric acid, (+/-)-Ethyl 2-hydroxy-3-methylvalerate, PC(18:0/18:2(9Z,12Z)), Hydroxyisocaproic acid, and 2-Naphthol sulfate.

Existing research has discovered that in overweight and obese COVID-19 patients, levels of N6-acetyl-L-lysine and p-cresol are elevated. Lysine acetylation is an emerging post-translational pathway primarily induced by obesity, which has been proven to modulate the enzymatic activity involved in fatty acid and glucose metabolism. This mechanism involves the transfer of an acetyl group from acetyl coenzyme A (acetyl-CoA), which is a key mediator and metabolic regulator of protein acetylation, targeting the amino groups of lysine (22). Existing research has discovered that disruption in lysine degradation may play a role in the development of early cardiac hypertrophy. Metabolites such as N6-acetyl-L-lysine might serve as potential predictive and therapeutic targets for subclinical myocardial cell hypertrophy (23). The primary metabolic product of progesterone is pregnanediol. The excretion of pregnanediol in urine can be used as an indicator to evaluate luteal function. Pregnanediol 3-O-glucuronide is a natural metabolite of pregnanediol, produced by the action of UDP-glucuronyltransferase in the liver. A metabolomic study has indicated that the levels of pregnanediol 3-O-glucuronide are significantly reduced in the placental metabolome of women with spontaneous preterm birth (24). 2-Linoleoylglycerol (18:2) has been identified as being associated with colorectal cancer (25). LysoPC (18:2/0:0) and 1-linoleoyl-gpc (18:2) are considered to be candidate diagnostic biomarkers for Hunner’s type interstitial cystitis (26). Additionally, they may represent potential therapeutic targets for patients hospitalized with mild traumatic brain injury (27). Studies have found that dicaffeoylquinic acids (diCQAs) reduce depression-like behaviors in mice treated with corticosterone (CORT), including memory loss. The potential mechanism of diCQAs’ antidepressant effects may involve the inhibition of monoamine oxidase types A and B (MAO-A and MAO-B) activities in neurons and astrocytes, leading to a decrease in the production of reactive oxygen species (ROS) in the brain (28). This is consistent with our findings that quinic acid acts as a protective factor against Major Depressive Disorder (MDD). Adenosine 5’-diphosphate (ADP) is a vital organic compound in metabolism and is crucial for the flow of energy within living cells. Adenosine 5’-(α,β methylene)diphosphate (APCP), an ecto-5’-nucleotidase (e5NT) inhibitor, was administered through intraventricular injection to explore the regulatory effects of e5NT on nucleoside levels and the behavioral changes induced by acute restraint stress in mice.

Liu and colleagues have posited that inhibiting ecto-5’-nucleotidase (e5NT) could potentially alleviate anxious behaviors in mice. Consequently, targeting e5NT might represent a promising therapeutic strategy for the management of anxiety in murine models (29). Research indicates that the ratio between non-high-density lipoprotein cholesterol and high-density lipoprotein cholesterol is significantly correlated with an increased risk of depression among adults in the United States (30). The Reverse Cholesterol Transport (RCT) system constitutes a vital detoxification pathway that facilitates the clearance of free cholesterol from the body, thereby preventing the development of atherosclerosis. It also protects against lipid peroxidation, the oxidation of low-density lipoprotein (LDL) and high-density lipoprotein (HDL) cholesterol, as well as inflammatory responses. Current evidence suggests that dysfunction within the HDL-PON1-ApoA-LCAT complex is closely associated with the pathogenesis of affective disorders, the recurrence of Major Depressive Disorder (MDD) and Bipolar Disorder (BD), suicidal behaviors, and the severity of depression. Interestingly, in approximately 30% of patients experiencing severe Major Depressive Episodes (MDE), higher Hamilton Depression Rating Scale (HAMD) scores have also been linked with elevated levels of triglycerides, total cholesterol, and LDL cholesterol (31).

### 4.3 Metabolic pathway analysis

Bile acids, synthesized predominantly in the liver through the enzymatic oxidation of cholesterol, are essential for the digestion and absorption of lipids and fat-soluble vitamins. Emerging research has uncovered further physiological roles for bile acids that extend beyond their digestive function. These include the modulation of energy homeostasis, regulation of glucose metabolism, and influence on immune system responses. The mechanism underlying these diverse effects involves the activation of bile acid receptors, particularly the Farnesoid X receptor (FXR) and the G protein-coupled bile acid receptor 5 (TGR5). In recent years, studies have begun to examine the potential link between bile acid biosynthesis and mood regulation, particularly in relation to major depression. Some research suggests that bile acids may indirectly impact mood and cognitive functions by affecting the composition of the gut microbiota, communication along the gut-brain axis, and the release of hormones and metabolites. The gut microbiome is considered a potential key factor influencing brain function and emotional regulation, with bile acids being one of the elements that regulate the balance of gut flora (32, 33).

The antioxidant glutathione (GSH), tripeptidic in nature, plays a pivotal role in numerous biological processes. As the most abundant non-protein thiol antioxidant within cells, it primarily functions to neutralize reactive oxygen species (ROS) and peroxides, thereby shielding cells from the damaging effects of oxidative stress. Oxidative stress is thought to potentially play a role in mental illnesses, especially in major depression. Some studies have indicated that there may be an increase in oxidative stress among individuals with depression, and GSH, as a key antioxidant, could have its levels and metabolism affected in this context. Low levels of GSH may lead to a reduced capacity for antioxidant defense in the brain, potentially damaging neurons, disrupting the balance of neurotransmitters, and consequently, might be associated with the development of depressive symptoms (34). Furthermore, some studies have shown that a decline in glutathione levels may be connected with functional impairments in specific brain regions, such as the prefrontal cortex, which is a key area associated with emotion regulation and cognitive functions. In addition, inflammation has been linked to depression, and GSH plays a role in regulating inflammatory responses (35).

However, further research is needed to elucidate the precise mechanisms of GSH metabolism and how it may directly or indirectly affect the onset, progression, and treatment of depression. This could include investigating whether supplementing GSH or enhancing the activity of its metabolic pathways could serve as potential therapeutic approaches for depression. This aligns with the findings of our current research. To date, there is no direct evidence or widely recognized studies linking the metabolism of threonine and 2-oxobutanoate directly with major depression. However, the connection between metabolic pathways and mental health conditions is an area continuously explored in psychopathology and neurobiology, which includes the influence of amino acid metabolic pathways on emotion regulation and brain function.

Threonine is an essential amino acid that plays a role in protein synthesis as a building block and also participates in key metabolic pathways such as the threonine-methionine cycle and the glycine-serine cycle. Additionally, threonine can be transformed into glycine and serine and is associated with one-carbon metabolism, which involves neurotransmitter synthesis and methylation reactions (36).

2-Oxobutanoate is an intermediate product in the degradation pathways of certain amino acids such as isoleucine, lysine, methionine, and threonine. Through metabolic reactions, it can ultimately be converted into succinyl-CoA, which then enters the tricarboxylic acid cycle (TCA cycle), a critical pathway for cellular energy production (37).

Valine, leucine, and isoleucine are three branched-chain amino acids (BCAAs) which have been implicated in the pathogenesis of various mental disorders, including major depressive disorder (MDD), according to some studies. Research suggests that patients with depression might exhibit alterations in BCAAs levels in their blood compared to the healthy population, and these changes could reflect on their mood, cognitive function, and overall mental state. BCAAs serve as precursors for crucial neurotransmitters in the brain, can compete with tryptophan and tyrosine for the same transport system across the blood-brain barrier, and are also involved in energy metabolism, potentially exerting a significant impact on brain function, particularly in response to stress and during the regulation of emotions (38, 39).

Ascorbate metabolism and aldarate metabolism involve the biochemical pathways and respective metabolites of vitamin C (ascorbate) within the human body (40). Ascorbic acid, commonly known as vitamin C, is a potent antioxidant that helps neutralize free radicals and reduce oxidative stress. Oxidative stress is believed to play a critical role in the onset and progression of depression. Vitamin C is involved in the synthesis of various neurotransmitters, such as dopamine and serotonin, which are directly linked to mood regulation, depression, and other psychological disorders. The connection between inflammation and depression is increasingly recognized, and vitamin C possesses anti-inflammatory properties. Vitamin C has demonstrated neuroprotective effects in some studies, potentially aiding in the prevention of neurodegenerative diseases, which may be associated with maintaining cognitive function and emotional health (41). However, at present, there is limited research on its association with Major Depressive Disorder (MDD), which warrants further investigation.

Depression may be linked to a variety of factors, including genetics, environment, psychosocial elements, and the neurobiology of the brain. Abnormalities in certain metabolic pathways may lead to or be associated with changes in neurotransmitter balance, which in turn affect mood. For instance, disruptions in methylation reactions can impact gene expression and neurotransmitter synthesis, while aberrations in the energy metabolism implicated in the tricarboxylic acid (TCA) cycle may affect the function of the nervous system (42). Therefore, these metabolic pathways are closely related to Major Depressive Disorder (MDD) and warrant further in-depth research.

### 4.4 Advantages and limitations

This study boasts several advantages. Firstly, using GWAS data, our Mendelian Randomization (MR) analysis provides new insights into potential causal mediators for 1,400 metabolites and Major Depressive Disorder (MDD). Secondly, the multiple cohort setup based on original GWAS data enables us to make effective causal inferences within a large population scale, yielding high statistical power. Lastly, by integrating the relevant metabolites into MetaboAnalyst 6.0 for analysis, we are able to comprehensively evaluate the functional enrichment and metabolic pathways of these metabolites. This can further aid in informing the prioritization of drug targets and the development of clinical trials.

However, our study is not without its limitations. First, our MR study is based on summary data from GWAS, while psychiatric disorders are primarily caused by brain pathologies. Further research is required to analyze changes in metabolites within the cerebrospinal fluid to identify other promising biomarkers and drug targets for psychiatric conditions. Second, our study was predominantly conducted in individuals of European descent, reducing population stratification bias yet limiting the extrapolation of our findings across different ethnicities. Additional research in non-European populations is necessary to confirm our findings. Lastly, there may be participant overlap between GWAS cohorts, potentially leading to weak instrument bias. Although F-statistics suggest that there is no instrument bias in our MR study, further MR research based on independent cohorts with no participant overlap is needed to better understand the role of blood metabolites in the etiology of psychiatric diseases.

## 5 Conclusion

In summary, this MR study has determined that out of 1,400 blood metabolites analyzed, we have identified 34 known metabolites and 13 unknown metabolites, as well as 18 metabolite ratios, that are associated with Major Depressive Disorder (MDD). Additionally, we have highlighted 3 functionally enriched groups and 2 metabolic pathways. These findings provide preliminary evidence on the impact of blood metabolite dysregulation on the risk of MDD. The integration of genomics and metabolomics offers significant insights for the screening and prevention of MDD.

This study identified specific metabolites associated with increased or decreased risk of major depression. For example, N6-acetyllysine levels and the levels of 1-stearoyl-GPE (18: 0). This is crucial because these metabolites can be used as biomarkers for early detection or development risk of major depression. In clinical practice, having reliable biomarkers can significantly improve the screening process and carry out early intervention, which is of great significance for the treatment of this disease. The rich data of metabolic pathways (such as bile acid biosynthesis, glutathione metabolism) enhance the understanding of the pathophysiological mechanism behind MDD. This deeper understanding may lead to the development of new therapeutic targets. For example, interventions aimed at altering specific metabolic pathways may help prevent or alleviate symptoms of major depression. In addition, this study also paves the way for more personalized treatment interventions. Understanding individual metabolic characteristics can enable clinicians to tailor treatment plans based on the patient ‘s specific metabolic disorders, thereby potentially improving the therapeutic effect and minimizing unnecessary side effects.

## Data availability statement

The datasets presented in this study can be found in online repositories. The names of the repository/repositories and accession number(s) can be found in the article/**Supplementary Material**.

## Ethics statement

Ethical review and approval was not required for the study of human participants in accordance with the local legislation and institutional requirements.

## Author contributions

TD: Conceptualization, Methodology, Supervision, Writing – original draft, Writing – review & editing. XW: Data curation, Investigation, Software, Writing – original draft. ZJ: Formal analysis, Methodology, Project administration, Writing – original draft. YL: Formal analysis, Project administration, Resources, Writing – review & editing. JY: Funding acquisition, Project administration, Visualization, Writing – review & editing.
