# Supplementary material for: Assessing the associations of 1,400 blood metabolites with major depressive disorder: a Mendelian randomization study
Source: Front Psychiatry. 2024 Jun 6;15:1391535. doi: 10.3389/fpsyt.2024.1391535 (PMC11187323; doi:10.3389/fpsyt.2024.1391535)
Supplement: Supplementary file 1 [file Table_1.docx]

Five MR Models estimated causality between 1400 metabolites and their ratio and major depressive disorder, and tested for heterogeneity and level pleiotropy.

| CLASS | Metabolite name and number | Method | SNP(n) | pval | OR | 95%CI | heterogeneity |  | pleiotropy |  |
| --- | --- | --- | --- | --- | --- | --- | --- | --- | --- | --- |
|  |  |  |  |  |  |  | Qvalue | P | intercept | P |
| Lipids | 1-stearoyl-2-arachidonoyl-GPI (18:0/20:4) levels | MR Egger | 19 | 0.220 | 1.03 | 0.99-1.07 | 14.36 | 0.641 |  |  |
|  | GCST90199649 | Inverse variance weighted | 19 | 0.026 | 1.02 | 1-1.04 | 14.46 | 0.698 | 0.00 | 0.756 |
|  |  | Weighted median | 19 | 0.064 | 1.03 | 1-1.05 |  |  |  |  |
|  |  | Simple mode | 19 | 0.814 | 1.01 | 0.96-1.06 |  |  |  |  |
|  |  | Weighted mode | 19 | 0.015 | 1.05 | 1.01-1.08 |  |  |  |  |
|  |  |  |  |  |  |  |  |  |  |  |
| Lipids | Alpha-hydroxyisocaproate levels | MR Egger | 22 | 0.820 | 0.99 | 0.91-1.08 | 33.89 | 0.027 |  |  |
|  | GCST90199658 | Inverse variance weighted | 22 | 0.042 | 0.97 | 0.93-1 | 34.53 | 0.032 | 0.00 | 0.548 |
|  |  | Weighted median | 22 | 0.145 | 0.97 | 0.94-1.01 |  |  |  |  |
|  |  | Simple mode | 22 | 0.409 | 0.97 | 0.91-1.04 |  |  |  |  |
|  |  | Weighted mode | 22 | 0.399 | 0.97 | 0.92-1.03 |  |  |  |  |
|  |  |  |  |  |  |  |  |  |  |  |
| Lipids | 2-linoleoylglycerol (18:2) levels | MR Egger | 19 | 0.096 | 0.95 | 0.9-1.01 | 6.81 | 0.986 |  |  |
|  | GCST90199685 | Inverse variance weighted | 19 | 0.003 | 0.96 | 0.94-0.99 | 7.10 | 0.989 | 0.00 | 0.598 |
|  |  | Weighted median | 19 | 0.011 | 0.96 | 0.93-0.99 |  |  |  |  |
|  |  | Simple mode | 19 | 0.149 | 0.96 | 0.91-1.01 |  |  |  |  |
|  |  | Weighted mode | 19 | 0.146 | 0.96 | 0.91-1.01 |  |  |  |  |
|  |  |  |  |  |  |  |  |  |  |  |
| Lipids | Docosatrienoate (22:3n3) levels | MR Egger | 21 | 0.096 | 0.96 | 0.92-1 | 21.25 | 0.323 |  |  |
|  | GCST90199710 | Inverse variance weighted | 21 | 0.003 | 0.97 | 0.95-0.99 | 21.48 | 0.370 | 0.00 | 0.657 |
|  |  | Weighted median | 21 | 0.002 | 0.95 | 0.93-0.98 |  |  |  |  |
|  |  | Simple mode | 21 | 0.377 | 0.98 | 0.93-1.03 |  |  |  |  |
|  |  | Weighted mode | 21 | 0.010 | 0.95 | 0.92-0.99 |  |  |  |  |
|  |  |  |  |  |  |  |  |  |  |  |
| Lipids | 1-linoleoyl-gpc (18:2) levels | MR Egger | 22 | 0.088 | 0.95 | 0.89-1.01 | 23.20 | 0.279 |  |  |
|  | GCST90199742 | Inverse variance weighted | 22 | 0.005 | 0.96 | 0.94-0.99 | 23.63 | 0.311 | 0.00 | 0.549 |
|  |  | Weighted median | 22 | 0.231 | 0.98 | 0.94-1.01 |  |  |  |  |
|  |  | Simple mode | 22 | 0.613 | 0.98 | 0.91-1.06 |  |  |  |  |
|  |  | Weighted mode | 22 | 0.648 | 0.98 | 0.9-1.07 |  |  |  |  |
|  |  |  |  |  |  |  |  |  |  |  |
| Lipids | 1-stearoyl-GPE (18:0) levels | MR Egger | 36 | 0.422 | 1.02 | 0.97-1.08 | 41.79 | 0.168 |  |  |
|  | GCST90199772 | Inverse variance weighted | 36 | 0.003 | 1.03 | 1.01-1.05 | 41.92 | 0.196 | 0.00 | 0.754 |
|  |  | Weighted median | 36 | 0.063 | 1.03 | 1-1.06 |  |  |  |  |
|  |  | Simple mode | 36 | 0.142 | 1.05 | 0.99-1.11 |  |  |  |  |
|  |  | Weighted mode | 36 | 0.146 | 1.03 | 0.99-1.08 |  |  |  |  |
|  |  |  |  |  |  |  |  |  |  |  |
| Lipids | Malonylcarnitine levels | MR Egger | 20 | 0.472 | 1.02 | 0.96-1.09 | 21.48 | 0.256 |  |  |
|  | GCST90199776 | Inverse variance weighted | 20 | 0.035 | 1.03 | 1-1.05 | 21.48 | 0.311 | 0.00 | 0.957 |
|  |  | Weighted median | 20 | 0.148 | 1.02 | 0.99-1.06 |  |  |  |  |
|  |  | Simple mode | 20 | 0.678 | 1.01 | 0.95-1.08 |  |  |  |  |
|  |  | Weighted mode | 20 | 0.725 | 1.01 | 0.95-1.08 |  |  |  |  |
|  |  |  |  |  |  |  |  |  |  |  |
| Lipids | 2-hydroxy-3-methylvalerate levels | MR Egger | 28 | 0.011 | 0.92 | 0.87-0.98 | 31.32 | 0.217 |  |  |
|  | GCST90199786 | Inverse variance weighted | 28 | 0.034 | 0.97 | 0.95-1 | 36.17 | 0.112 | 0.01 | 0.055 |
|  |  | Weighted median | 28 | 0.773 | 1.00 | 0.96-1.03 |  |  |  |  |
|  |  | Simple mode | 28 | 0.076 | 0.94 | 0.88-1 |  |  |  |  |
|  |  | Weighted mode | 28 | 0.696 | 0.99 | 0.95-1.04 |  |  |  |  |
|  |  |  |  |  |  |  |  |  |  |  |
| Lipids | 1-arachidonoyl-gpc (20:4n6) levels | MR Egger | 24 | 0.043 | 1.03 | 1-1.06 | 37.47 | 0.021 |  |  |
|  | GCST90199788 | Inverse variance weighted | 24 | 0.041 | 1.02 | 1-1.04 | 39.39 | 0.018 | 0.00 | 0.300 |
|  |  | Weighted median | 24 | 0.001 | 1.03 | 1.01-1.04 |  |  |  |  |
|  |  | Simple mode | 24 | 0.828 | 1.01 | 0.94-1.08 |  |  |  |  |
|  |  | Weighted mode | 24 | 0.008 | 1.03 | 1.01-1.05 |  |  |  |  |
|  |  |  |  |  |  |  |  |  |  |  |
| Lipids | Pregnanediol-3-glucuronide levels | MR Egger | 30 | 0.037 | 0.96 | 0.92-1 | 30.21 | 0.353 |  |  |
|  | GCST90199917 | Inverse variance weighted | 30 | 0.002 | 0.97 | 0.96-0.99 | 31.17 | 0.357 | 0.00 | 0.353 |
|  |  | Weighted median | 30 | 0.062 | 0.98 | 0.95-1 |  |  |  |  |
|  |  | Simple mode | 30 | 0.299 | 0.98 | 0.93-1.02 |  |  |  |  |
|  |  | Weighted mode | 30 | 0.072 | 0.97 | 0.94-1 |  |  |  |  |
|  |  |  |  |  |  |  |  |  |  |  |
| Lipids | 1-stearoyl-2-linoleoyl-gpc (18:0/18:2) levels | MR Egger | 19 | 0.359 | 0.97 | 0.9-1.04 | 29.75 | 0.028 |  |  |
|  | GCST90200037 | Inverse variance weighted | 19 | 0.037 | 0.97 | 0.94-1 | 29.76 | 0.040 | 0.00 | 0.951 |
|  |  | Weighted median | 19 | 0.022 | 0.96 | 0.93-0.99 |  |  |  |  |
|  |  | Simple mode | 19 | 0.588 | 0.98 | 0.91-1.05 |  |  |  |  |
|  |  | Weighted mode | 19 | 0.006 | 0.95 | 0.91-0.98 |  |  |  |  |
|  |  |  |  |  |  |  |  |  |  |  |
| Lipids | 1-palmitoyl-2-docosahexaenoyl-gpc (16:0/22:6) levels | MR Egger | 21 | 0.091 | 1.07 | 0.99-1.14 | 21.53 | 0.308 |  |  |
|  | GCST90200043 | Inverse variance weighted | 21 | 0.029 | 1.03 | 1-1.05 | 22.93 | 0.292 | -0.01 | 0.280 |
|  |  | Weighted median | 21 | 0.315 | 1.02 | 0.98-1.05 |  |  |  |  |
|  |  | Simple mode | 21 | 0.672 | 1.01 | 0.95-1.08 |  |  |  |  |
|  |  | Weighted mode | 21 | 0.742 | 1.01 | 0.95-1.07 |  |  |  |  |
|  |  |  |  |  |  |  |  |  |  |  |
| Lipids | 1-palmitoyl-2-arachidonoyl-GPE (16:0/20:4) levels | MR Egger | 17 | 0.645 | 1.01 | 0.97-1.05 | 22.26 | 0.101 |  |  |
|  | GCST90200054 | Inverse variance weighted | 17 | 0.025 | 1.03 | 1-1.05 | 23.40 | 0.103 | 0.00 | 0.395 |
|  |  | Weighted median | 17 | 0.291 | 1.01 | 0.99-1.04 |  |  |  |  |
|  |  | Simple mode | 17 | 0.449 | 1.02 | 0.97-1.09 |  |  |  |  |
|  |  | Weighted mode | 17 | 0.355 | 1.01 | 0.99-1.04 |  |  |  |  |
|  |  |  |  |  |  |  |  |  |  |  |
| Lipids | 1-(1-enyl-palmitoyl)-2-palmitoleoyl-GPC (P-16:0/16:1) levels | MR Egger | 31 | 0.146 | 1.03 | 0.99-1.07 | 38.52 | 0.111 |  |  |
|  | GCST90200070 | Inverse variance weighted | 31 | 0.038 | 1.02 | 1-1.04 | 38.92 | 0.128 | 0.00 | 0.588 |
|  |  | Weighted median | 31 | 0.135 | 1.02 | 0.99-1.04 |  |  |  |  |
|  |  | Simple mode | 31 | 0.778 | 1.01 | 0.95-1.06 |  |  |  |  |
|  |  | Weighted mode | 31 | 0.190 | 1.02 | 0.99-1.04 |  |  |  |  |
|  |  |  |  |  |  |  |  |  |  |  |
| Lipids | 1-oleoyl-2-linoleoyl-GPE (18:1/18:2) levels | MR Egger | 25 | 0.037 | 0.97 | 0.94-1 | 25.82 | 0.310 |  |  |
|  | GCST90200082 | Inverse variance weighted | 25 | 0.021 | 0.98 | 0.97-1 | 27.23 | 0.294 | 0.00 | 0.274 |
|  |  | Weighted median | 25 | 0.001 | 0.96 | 0.94-0.99 |  |  |  |  |
|  |  | Simple mode | 25 | 0.330 | 0.97 | 0.93-1.02 |  |  |  |  |
|  |  | Weighted mode | 25 | 0.013 | 0.97 | 0.95-0.99 |  |  |  |  |
|  |  |  |  |  |  |  |  |  |  |  |
| Lipids | 1-linoleoyl-2-linolenoyl-GPC (18:2/18:3) levels | MR Egger | 16 | 0.046 | 0.94 | 0.89-0.99 | 15.91 | 0.319 |  |  |
|  | GCST90200095 | Inverse variance weighted | 16 | 0.017 | 0.97 | 0.94-0.99 | 17.62 | 0.283 | 0.00 | 0.240 |
|  |  | Weighted median | 16 | 0.001 | 0.94 | 0.91-0.97 |  |  |  |  |
|  |  | Simple mode | 16 | 0.406 | 0.96 | 0.89-1.05 |  |  |  |  |
|  |  | Weighted mode | 16 | 0.005 | 0.94 | 0.91-0.98 |  |  |  |  |
|  |  |  |  |  |  |  |  |  |  |  |
| Lipids | Linoleoyl-arachidonoyl-glycerol (18:2/20:4) [1] levels | MR Egger | 15 | 0.077 | 1.04 | 1-1.08 | 16.44 | 0.226 |  |  |
|  | GCST90200103 | Inverse variance weighted | 15 | 0.013 | 1.03 | 1.01-1.05 | 17.11 | 0.250 | 0.00 | 0.479 |
|  |  | Weighted median | 15 | 0.001 | 1.05 | 1.02-1.08 |  |  |  |  |
|  |  | Simple mode | 15 | 0.138 | 1.04 | 0.99-1.1 |  |  |  |  |
|  |  | Weighted mode | 15 | 0.006 | 1.05 | 1.02-1.08 |  |  |  |  |
|  |  |  |  |  |  |  |  |  |  |  |
| Lipids | Glycosyl-N-tricosanoyl-sphingadienine (d18:2/23:0) levels | MR Egger | 22 | 0.563 | 0.98 | 0.94-1.04 | 20.76 | 0.411 |  |  |
|  | GCST90200114 | Inverse variance weighted | 22 | 0.007 | 0.97 | 0.95-0.99 | 21.14 | 0.450 | 0.00 | 0.553 |
|  |  | Weighted median | 22 | 0.055 | 0.97 | 0.94-1 |  |  |  |  |
|  |  | Simple mode | 22 | 0.727 | 0.99 | 0.94-1.04 |  |  |  |  |
|  |  | Weighted mode | 22 | 0.106 | 0.97 | 0.93-1.01 |  |  |  |  |
|  |  |  |  |  |  |  |  |  |  |  |
| Lipids | Taurodeoxycholate levels | MR Egger | 19 | 0.977 | 1.00 | 0.94-1.06 | 14.42 | 0.637 |  |  |
|  | GCST90200334 | Inverse variance weighted | 19 | 0.032 | 0.97 | 0.95-1 | 15.54 | 0.625 | 0.00 | 0.305 |
|  |  | Weighted median | 19 | 0.476 | 0.99 | 0.95-1.02 |  |  |  |  |
|  |  | Simple mode | 19 | 0.842 | 0.99 | 0.93-1.06 |  |  |  |  |
|  |  | Weighted mode | 19 | 0.842 | 0.99 | 0.93-1.06 |  |  |  |  |
|  |  |  |  |  |  |  |  |  |  |  |
| Lipids | Methylsuccinate levels | MR Egger | 18 | 0.004 | 1.05 | 1.02-1.09 | 11.42 | 0.783 |  |  |
|  | GCST90200352 | Inverse variance weighted | 18 | 0.007 | 1.03 | 1.01-1.05 | 15.91 | 0.53 | -0.01 | 0.05 |
|  |  | Weighted median | 18 | 0.001 | 1.04 | 1.02-1.07 |  |  |  |  |
|  |  | Simple mode | 18 | 0.636 | 1.02 | 0.95-1.08 |  |  |  |  |
|  |  | Weighted mode | 18 | 0.003 | 1.04 | 1.02-1.07 |  |  |  |  |
|  |  |  |  |  |  |  |  |  |  |  |
| Lipids | Cholesterol levels | MR Egger | 18 | 0.167 | 0.96 | 0.91-1.01 | 10.01 | 0.866 |  |  |
|  | GCST90200368 | Inverse variance weighted | 18 | 0.011 | 0.96 | 0.94-0.99 | 10.06 | 0.901 | 0 | 0.829 |
|  |  | Weighted median | 18 | 0.034 | 0.96 | 0.92-1 |  |  |  |  |
|  |  | Simple mode | 18 | 0.144 | 0.95 | 0.89-1.02 |  |  |  |  |
|  |  | Weighted mode | 18 | 0.183 | 0.95 | 0.89-1.02 |  |  |  |  |
|  |  |  |  |  |  |  |  |  |  |  |
| Lipids | 1-palmitoyl-2-arachidonoyl-gpc (16:0/20:4n6) levels | MR Egger | 28 | 0.115 | 1.02 | 1-1.05 | 46.92 | 0.007 |  |  |
|  | GCST90200692 | Inverse variance weighted | 28 | 0.016 | 1.02 | 1-1.04 | 46.93 | 0.01 | 0 | 0.955 |
|  |  | Weighted median | 28 | 0.001 | 1.03 | 1.01-1.04 |  |  |  |  |
|  |  | Simple mode | 28 | 0.524 | 1.02 | 0.96-1.08 |  |  |  |  |
|  |  | Weighted mode | 28 | 0.005 | 1.02 | 1.01-1.04 |  |  |  |  |
|  |  |  |  |  |  |  |  |  |  |  |
| Amino acids | N-acetylglutamate levels | MR Egger | 19 | 0.977 | 1.00 | 0.94-1.07 | 17.56 | 0.417 |  |  |
|  | GCST90199637 | Inverse variance weighted | 19 | 0.007 | 0.96 | 0.94-0.99 | 18.99 | 0.392 | 0.00 | 0.256 |
|  |  | Weighted median | 19 | 0.250 | 0.98 | 0.94-1.02 |  |  |  |  |
|  |  | Simple mode | 19 | 0.586 | 0.98 | 0.92-1.05 |  |  |  |  |
|  |  | Weighted mode | 19 | 0.549 | 0.98 | 0.92-1.04 |  |  |  |  |
|  |  |  |  |  |  |  |  |  |  |  |
| Amino acids | N-acetylhistidine levels | MR Egger | 20 | 0.039 | 0.98 | 0.96-1 | 18.02 | 0.454 |  |  |
|  | GCST90199735 | Inverse variance weighted | 20 | 0.024 | 0.98 | 0.97-1 | 18.95 | 0.460 | 0.00 | 0.349 |
|  |  | Weighted median | 20 | 0.025 | 0.98 | 0.96-1 |  |  |  |  |
|  |  | Simple mode | 20 | 0.841 | 1.00 | 0.96-1.04 |  |  |  |  |
|  |  | Weighted mode | 20 | 0.038 | 0.98 | 0.97-1 |  |  |  |  |
|  |  |  |  |  |  |  |  |  |  |  |
| Amino acids | N6-acetyllysine levels | MR Egger | 17 | 0.031 | 0.96 | 0.92-0.99 | 24.80 | 0.053 |  |  |
|  | GCST90199826 | Inverse variance weighted | 17 | 0.002 | 0.96 | 0.94-0.99 | 25.08 | 0.068 | 0.00 | 0.690 |
|  |  | Weighted median | 17 | 0.002 | 0.96 | 0.94-0.99 |  |  |  |  |
|  |  | Simple mode | 17 | 0.031 | 0.94 | 0.89-0.99 |  |  |  |  |
|  |  | Weighted mode | 17 | 0.005 | 0.96 | 0.94-0.98 |  |  |  |  |
|  |  |  |  |  |  |  |  |  |  |  |
| Amino acids | 6-oxopiperidine-2-carboxylate levels | MR Egger | 22 | 0.075 | 0.97 | 0.93-1 | 27.22 | 0.129 |  |  |
|  | GCST90199949 | Inverse variance weighted | 22 | 0.003 | 0.97 | 0.95-0.99 | 27.22 | 0.164 | 0.00 | 0.967 |
|  |  | Weighted median | 22 | 0.009 | 0.97 | 0.94-0.99 |  |  |  |  |
|  |  | Simple mode | 22 | 0.301 | 0.97 | 0.92-1.03 |  |  |  |  |
|  |  | Weighted mode | 22 | 0.029 | 0.97 | 0.94-0.99 |  |  |  |  |
|  |  |  |  |  |  |  |  |  |  |  |
| Amino acids | 5-oxoproline levels | MR Egger | 22 | 0.014 | 0.97 | 0.95-0.99 | 18.29 | 0.568 |  |  |
|  | GCST90200280 | Inverse variance weighted | 22 | 0.013 | 0.98 | 0.96-1 | 20.07 | 0.517 | 0.00 | 0.198 |
|  |  | Weighted median | 22 | 0.010 | 0.98 | 0.96-0.99 |  |  |  |  |
|  |  | Simple mode | 22 | 0.251 | 0.97 | 0.92-1.02 |  |  |  |  |
|  |  | Weighted mode | 22 | 0.018 | 0.97 | 0.96-0.99 |  |  |  |  |
|  |  |  |  |  |  |  |  |  |  |  |
| Amino acids | Gamma-glutamyltyrosine levels | MR Egger | 25 | 0.492 | 1.02 | 0.97-1.07 | 13.91 | 0.930 |  |  |
|  | GCST90200295 | Inverse variance weighted | 25 | 0.040 | 1.02 | 1-1.05 | 14.00 | 0.947 | 0.00 | 0.761 |
|  |  | Weighted median | 25 | 0.126 | 1.03 | 0.99-1.06 |  |  |  |  |
|  |  | Simple mode | 25 | 0.392 | 1.03 | 0.97-1.09 |  |  |  |  |
|  |  | Weighted mode | 25 | 0.300 | 1.03 | 0.98-1.08 |  |  |  |  |
|  |  |  |  |  |  |  |  |  |  |  |
| Amino acids | Glutamine levels | MR Egger | 24 | 0.045 | 1.05 | 1-1.09 | 22.3 | 0.442 |  |  |
|  | GCST90200419 | Inverse variance weighted | 24 | 0.005 | 1.03 | 1.01-1.05 | 23.11 | 0.454 | 0 | 0.379 |
|  |  | Weighted median | 24 | 0.006 | 1.04 | 1.01-1.08 |  |  |  |  |
|  |  | Simple mode | 24 | 0.131 | 1.04 | 0.99-1.09 |  |  |  |  |
|  |  | Weighted mode | 24 | 0.018 | 1.05 | 1.01-1.08 |  |  |  |  |
|  |  |  |  |  |  |  |  |  |  |  |
| Keto acids | 2-oxoarginine levels | MR Egger | 20 | 0.699 | 0.98 | 0.91-1.07 | 21.38 | 0.261 |  |  |
|  | GCST90199903 | Inverse variance weighted | 20 | 0.015 | 0.97 | 0.94-0.99 | 21.60 | 0.305 | 0.00 | 0.668 |
|  |  | Weighted median | 20 | 0.258 | 0.98 | 0.94-1.02 |  |  |  |  |
|  |  | Simple mode | 20 | 0.101 | 0.93 | 0.86-1.01 |  |  |  |  |
|  |  | Weighted mode | 20 | 0.126 | 0.95 | 0.89-1.01 |  |  |  |  |
|  |  |  |  |  |  |  |  |  |  |  |
| Keto acids | Alpha-ketobutyrate levels | MR Egger | 14 | 0.224 | 0.96 | 0.89-1.02 | 7.51 | 0.822 |  |  |
|  | GCST90200438 | Inverse variance weighted | 14 | 0.034 | 0.97 | 0.94-1 | 7.62 | 0.867 | 0 | 0.746 |
|  |  | Weighted median | 14 | 0.353 | 0.98 | 0.94-1.02 |  |  |  |  |
|  |  | Simple mode | 14 | 0.669 | 0.98 | 0.92-1.06 |  |  |  |  |
|  |  | Weighted mode | 14 | 0.687 | 0.98 | 0.92-1.06 |  |  |  |  |
|  |  |  |  |  |  |  |  |  |  |  |
| Quinic acid | Quinate levels | MR Egger | 16 | 0.359 | 0.97 | 0.92-1.03 | 12.45 | 0.570 |  |  |
|  | GCST90199645 | Inverse variance weighted | 16 | 0.007 | 0.96 | 0.93-0.99 | 12.67 | 0.628 | 0.00 | 0.649 |
|  |  | Weighted median | 16 | 0.034 | 0.96 | 0.92-1 |  |  |  |  |
|  |  | Simple mode | 16 | 0.220 | 0.95 | 0.89-1.03 |  |  |  |  |
|  |  | Weighted mode | 16 | 0.207 | 0.95 | 0.89-1.02 |  |  |  |  |
|  |  |  |  |  |  |  |  |  |  |  |
| Carboxylic acids | N-acetyl-beta-alanine levels | MR Egger | 23 | 0.059 | 1.04 | 1-1.09 | 14.03 | 0.868 |  |  |
|  | GCST90199866 | Inverse variance weighted | 23 | 0.003 | 1.03 | 1.01-1.06 | 14.31 | 0.890 | 0.00 | 0.606 |
|  |  | Weighted median | 23 | 0.007 | 1.05 | 1.01-1.08 |  |  |  |  |
|  |  | Simple mode | 23 | 0.256 | 1.04 | 0.98-1.1 |  |  |  |  |
|  |  | Weighted mode | 23 | 0.039 | 1.05 | 1-1.09 |  |  |  |  |
|  |  |  |  |  |  |  |  |  |  |  |
| Naphthalenes | 2-naphthol sulfate levels | MR Egger | 20 | 0.061 | 0.94 | 0.89-1 | 14.18 | 0.717 |  |  |
|  | GCST90200213 | Inverse variance weighted | 20 | 0.045 | 0.97 | 0.95-1 | 15.65 | 0.680 | 0.00 | 0.241 |
|  |  | Weighted median | 20 | 0.104 | 0.97 | 0.93-1.01 |  |  |  |  |
|  |  | Simple mode | 20 | 0.347 | 0.96 | 0.9-1.04 |  |  |  |  |
|  |  | Weighted mode | 20 | 0.392 | 0.97 | 0.91-1.04 |  |  |  |  |
|  |  |  |  |  |  |  |  |  |  |  |
| Purine nucleotides | Adenosine 5'-diphosphate (ADP) levels | MR Egger | 22 | 0.106 | 0.96 | 0.91-1.01 | 22.69 | 0.304 |  |  |
|  | GCST90200355 | Inverse variance weighted | 22 | 0.007 | 0.97 | 0.95-0.99 | 23.26 | 0.33 | 0 | 0.487 |
|  |  | Weighted median | 22 | 0.018 | 0.97 | 0.94-0.99 |  |  |  |  |
|  |  | Simple mode | 22 | 0.171 | 0.96 | 0.91-1.02 |  |  |  |  |
|  |  | Weighted mode | 22 | 0.137 | 0.96 | 0.91-1.01 |  |  |  |  |
|  |  |  |  |  |  |  |  |  |  |  |
| Ratio | Adenosine 5'-diphosphate (ADP) to Adenosine 5'-monophosphate (AMP) ratio | MR Egger | 23 | 0.411 | 0.98 | 0.94-1.03 | 14.22 | 0.86 |  |  |
|  | GCST90200728 | Inverse variance weighted | 23 | 0.015 | 0.98 | 0.96-1 | 14.24 | 0.893 | 0 | 0.888 |
|  |  | Weighted median | 23 | 0.111 | 0.98 | 0.96-1 |  |  |  |  |
|  |  | Simple mode | 23 | 0.576 | 0.99 | 0.94-1.03 |  |  |  |  |
|  |  | Weighted mode | 23 | 0.560 | 0.99 | 0.94-1.03 |  |  |  |  |
|  |  |  |  |  |  |  |  |  |  |  |
| Ratio | Arachidonate (20:4n6) to oleate to vaccenate (18:1) ratio | MR Egger | 18 | 0.071 | 1.03 | 1-1.07 | 25.37 | 0.063 |  |  |
|  | GCST90200740 | Inverse variance weighted | 18 | 0.004 | 1.03 | 1.01-1.05 | 25.47 | 0.085 | 0 | 0.811 |
|  |  | Weighted median | 18 | 0.001 | 1.04 | 1.02-1.06 |  |  |  |  |
|  |  | Simple mode | 18 | 0.897 | 1 | 0.95-1.06 |  |  |  |  |
|  |  | Weighted mode | 18 | 0.005 | 1.03 | 1.01-1.05 |  |  |  |  |
|  |  |  |  |  |  |  |  |  |  |  |
| Ratio | Oleoyl-linoleoyl-glycerol (18:1 to 18:2) [2] to linoleoyl-arachidonoyl-glycerol (18:2 to 20:4) [2] ratio | MR Egger | 20 | 0.003 | 0.96 | 0.93-0.98 | 20.07 | 0.329 |  |  |
|  | GCST90200795 | Inverse variance weighted | 20 | 0.002 | 0.97 | 0.96-0.99 | 23.31 | 0.224 | 0 | 0.105 |
|  |  | Weighted median | 20 | 0.001 | 0.97 | 0.95-0.99 |  |  |  |  |
|  |  | Simple mode | 20 | 0.845 | 0.99 | 0.95-1.05 |  |  |  |  |
|  |  | Weighted mode | 20 | 0.003 | 0.97 | 0.95-0.99 |  |  |  |  |
|  |  |  |  |  |  |  |  |  |  |  |
| Ratio | Adenosine 5'-monophosphate (AMP) to glutamine ratio | MR Egger | 16 | 0.056 | 1.07 | 1-1.14 | 8.18 | 0.88 |  |  |
|  | GCST90200848 | Inverse variance weighted | 16 | 0.041 | 1.03 | 1-1.06 | 9.73 | 0.837 | 0 | 0.234 |
|  |  | Weighted median | 16 | 0.086 | 1.04 | 0.99-1.08 |  |  |  |  |
|  |  | Simple mode | 16 | 0.100 | 1.06 | 0.99-1.14 |  |  |  |  |
|  |  | Weighted mode | 16 | 0.107 | 1.06 | 0.99-1.13 |  |  |  |  |
|  |  |  |  |  |  |  |  |  |  |  |
| Ratio | Adenosine 5'-monophosphate (AMP) to asparagine ratio | MR Egger | 20 | 0.085 | 1.06 | 1-1.13 | 9.33 | 0.952 |  |  |
|  | GCST90200859 | Inverse variance weighted | 20 | 0.004 | 1.04 | 1.01-1.07 | 9.83 | 0.957 | 0 | 0.49 |
|  |  | Weighted median | 20 | 0.048 | 1.04 | 1-1.07 |  |  |  |  |
|  |  | Simple mode | 20 | 0.369 | 1.03 | 0.97-1.1 |  |  |  |  |
|  |  | Weighted mode | 20 | 0.324 | 1.03 | 0.97-1.1 |  |  |  |  |
|  |  |  |  |  |  |  |  |  |  |  |
| Ratio | Adenosine 5'-monophosphate (AMP) to serine ratio | MR Egger | 15 | 0.350 | 1.04 | 0.96-1.11 | 11.82 | 0.543 |  |  |
|  | GCST90200860 | Inverse variance weighted | 15 | 0.046 | 1.03 | 1-1.06 | 11.85 | 0.619 | 0 | 0.867 |
|  |  | Weighted median | 15 | 0.112 | 1.03 | 0.99-1.08 |  |  |  |  |
|  |  | Simple mode | 15 | 0.123 | 1.06 | 0.99-1.14 |  |  |  |  |
|  |  | Weighted mode | 15 | 0.142 | 1.06 | 0.99-1.14 |  |  |  |  |
|  |  |  |  |  |  |  |  |  |  |  |
| Ratio | Phosphate to serine ratio | MR Egger | 21 | 0.160 | 1.05 | 0.98-1.11 | 14.85 | 0.732 |  |  |
|  | GCST90200863 | Inverse variance weighted | 21 | 0.041 | 1.02 | 1-1.05 | 15.41 | 0.753 | 0 | 0.464 |
|  |  | Weighted median | 21 | 0.033 | 1.04 | 1-1.07 |  |  |  |  |
|  |  | Simple mode | 21 | 0.310 | 1.03 | 0.97-1.1 |  |  |  |  |
|  |  | Weighted mode | 21 | 0.166 | 1.03 | 0.99-1.08 |  |  |  |  |
|  |  |  |  |  |  |  |  |  |  |  |
| Ratio | Methionine to phosphate ratio | MR Egger | 14 | 0.364 | 0.97 | 0.9-1.04 | 11.95 | 0.449 |  |  |
|  | GCST90200864 | Inverse variance weighted | 14 | 0.002 | 0.95 | 0.92-0.98 | 12.27 | 0.506 | 0 | 0.586 |
|  |  | Weighted median | 14 | 0.107 | 0.96 | 0.92-1.01 |  |  |  |  |
|  |  | Simple mode | 14 | 0.635 | 0.98 | 0.9-1.06 |  |  |  |  |
|  |  | Weighted mode | 14 | 0.609 | 0.98 | 0.91-1.06 |  |  |  |  |
|  |  |  |  |  |  |  |  |  |  |  |
| Ratio | Adenosine 5'-monophosphate (AMP) to isoleucine ratio | MR Egger | 19 | 0.041 | 1.09 | 1.01-1.17 | 13.9 | 0.674 |  |  |
|  | GCST90200867 | Inverse variance weighted | 19 | 0.039 | 1.03 | 1-1.06 | 16.3 | 0.572 | -0.01 | 0.14 |
|  |  | Weighted median | 19 | 0.264 | 1.02 | 0.98-1.06 |  |  |  |  |
|  |  | Simple mode | 19 | 0.702 | 1.01 | 0.95-1.09 |  |  |  |  |
|  |  | Weighted mode | 19 | 0.710 | 1.01 | 0.95-1.08 |  |  |  |  |
|  |  |  |  |  |  |  |  |  |  |  |
| Ratio | Phenylpyruvate to citrate ratio | MR Egger | 27 | 0.118 | 0.96 | 0.91-1.01 | 17.16 | 0.876 |  |  |
|  | GCST90200885 | Inverse variance weighted | 27 | 0.017 | 0.97 | 0.95-1 | 17.61 | 0.889 | 0 | 0.506 |
|  |  | Weighted median | 27 | 0.020 | 0.96 | 0.93-0.99 |  |  |  |  |
|  |  | Simple mode | 27 | 0.146 | 0.96 | 0.9-1.01 |  |  |  |  |
|  |  | Weighted mode | 27 | 0.130 | 0.96 | 0.9-1.01 |  |  |  |  |
|  |  |  |  |  |  |  |  |  |  |  |
| Ratio | Cortisol to taurocholate ratio | MR Egger | 20 | 0.364 | 0.98 | 0.93-1.03 | 20.07 | 0.329 |  |  |
|  | GCST90200890 | Inverse variance weighted | 20 | 0.040 | 0.97 | 0.95-1 | 20.09 | 0.389 | 0 | 0.896 |
|  |  | Weighted median | 20 | 0.070 | 0.97 | 0.93-1 |  |  |  |  |
|  |  | Simple mode | 20 | 0.248 | 0.96 | 0.9-1.02 |  |  |  |  |
|  |  | Weighted mode | 20 | 0.279 | 0.96 | 0.91-1.03 |  |  |  |  |
|  |  |  |  |  |  |  |  |  |  |  |
| Ratio | Alpha-ketoglutarate to proline ratio | MR Egger | 21 | 0.313 | 0.97 | 0.91-1.03 | 18.23 | 0.507 |  |  |
|  | GCST90200933 | Inverse variance weighted | 21 | 0.050 | 0.98 | 0.95-1 | 18.29 | 0.568 | 0 | 0.809 |
|  |  | Weighted median | 21 | 0.490 | 0.99 | 0.95-1.02 |  |  |  |  |
|  |  | Simple mode | 21 | 0.853 | 0.99 | 0.93-1.06 |  |  |  |  |
|  |  | Weighted mode | 21 | 0.830 | 0.99 | 0.94-1.06 |  |  |  |  |
|  |  |  |  |  |  |  |  |  |  |  |
| Ratio | Phosphate to 5-oxoproline ratio | MR Egger | 24 | 0.194 | 1.02 | 0.99-1.05 | 29.15 | 0.141 |  |  |
|  | GCST90200968 | Inverse variance weighted | 24 | 0.010 | 1.03 | 1.01-1.05 | 29.52 | 0.164 | 0 | 0.602 |
|  |  | Weighted median | 24 | 0.012 | 1.03 | 1.01-1.05 |  |  |  |  |
|  |  | Simple mode | 24 | 0.299 | 1.03 | 0.97-1.09 |  |  |  |  |
|  |  | Weighted mode | 24 | 0.026 | 1.03 | 1.01-1.05 |  |  |  |  |
|  |  |  |  |  |  |  |  |  |  |  |
| Ratio | Arachidonate (20:4n6) to linoleate (18:2n6) ratio | MR Egger | 20 | 0.041 | 1.04 | 1-1.07 | 24.95 | 0.126 |  |  |
|  | GCST90200979 | Inverse variance weighted | 20 | 0.001 | 1.03 | 1.01-1.05 | 25.08 | 0.158 | 0 | 0.761 |
|  |  | Weighted median | 20 | 0.000 | 1.04 | 1.02-1.06 |  |  |  |  |
|  |  | Simple mode | 20 | 0.275 | 1.03 | 0.98-1.09 |  |  |  |  |
|  |  | Weighted mode | 20 | 0.002 | 1.04 | 1.02-1.06 |  |  |  |  |
|  |  |  |  |  |  |  |  |  |  |  |
| Ratio | Benzoate to linoleoyl-arachidonoyl-glycerol (18:2 to 20:4) [2] ratio | MR Egger | 19 | 0.067 | 0.96 | 0.91-1 | 23.01 | 0.149 |  |  |
|  | GCST90200990 | Inverse variance weighted | 19 | 0.011 | 0.97 | 0.95-0.99 | 23.96 | 0.156 | 0 | 0.412 |
|  |  | Weighted median | 19 | 0.001 | 0.95 | 0.93-0.98 |  |  |  |  |
|  |  | Simple mode | 19 | 0.162 | 0.96 | 0.9-1.01 |  |  |  |  |
|  |  | Weighted mode | 19 | 0.006 | 0.95 | 0.92-0.98 |  |  |  |  |
|  |  |  |  |  |  |  |  |  |  |  |
| Ratio | Threonine to pyruvate ratio | MR Egger | 24 | 0.459 | 1.02 | 0.97-1.08 | 22.88 | 0.408 |  |  |
|  | GCST90201009 | Inverse variance weighted | 24 | 0.005 | 1.03 | 1.01-1.06 | 23.12 | 0.454 | 0 | 0.639 |
|  |  | Weighted median | 24 | 0.091 | 1.03 | 1-1.06 |  |  |  |  |
|  |  | Simple mode | 24 | 0.451 | 1.03 | 0.96-1.1 |  |  |  |  |
|  |  | Weighted mode | 24 | 0.388 | 1.03 | 0.97-1.08 |  |  |  |  |
|  |  |  |  |  |  |  |  |  |  |  |
| Ratio | Androsterone glucuronide to etiocholanolone glucuronide ratio | MR Egger | 26 | 0.045 | 1.04 | 1-1.08 | 52.33 | 0.001 |  |  |
|  | GCST90201013 | Inverse variance weighted | 26 | 0.028 | 1.03 | 1-1.05 | 53.99 | 0.001 | 0 | 0.391 |
|  |  | Weighted median | 26 | 0.002 | 1.04 | 1.01-1.06 |  |  |  |  |
|  |  | Simple mode | 26 | 0.176 | 1.04 | 0.99-1.09 |  |  |  |  |
|  |  | Weighted mode | 26 | 0.005 | 1.04 | 1.01-1.06 |  |  |  |  |
|  |  |  |  |  |  |  |  |  |  |  |
| Unknown | X-11470 levels | MR Egger | 30 | 0.177 | 1.02 | 0.99-1.05 | 41.08 | 0.053 |  |  |
|  | GCST90200470 | Inverse variance weighted | 30 | 0.019 | 1.02 | 1-1.04 | 41.12 | 0.067 | 0 | 0.867 |
|  |  | Weighted median | 30 | 0.288 | 1.02 | 0.99-1.04 |  |  |  |  |
|  |  | Simple mode | 30 | 0.088 | 1.05 | 0.99-1.1 |  |  |  |  |
|  |  | Weighted mode | 30 | 0.523 | 1.01 | 0.98-1.05 |  |  |  |  |
|  |  |  |  |  |  |  |  |  |  |  |
| Unknown | X-11444 levels | MR Egger | 28 | 0.013 | 1.04 | 1.01-1.07 | 32.99 | 0.162 |  |  |
|  | GCST90200474 | Inverse variance weighted | 28 | 0.002 | 1.03 | 1.01-1.05 | 34.08 | 0.164 | 0 | 0.361 |
|  |  | Weighted median | 28 | 0.089 | 1.02 | 1-1.05 |  |  |  |  |
|  |  | Simple mode | 28 | 0.358 | 1.03 | 0.97-1.09 |  |  |  |  |
|  |  | Weighted mode | 28 | 0.028 | 1.04 | 1.01-1.07 |  |  |  |  |
|  |  |  |  |  |  |  |  |  |  |  |
| Unknown | X-12410 levels | MR Egger | 21 | 0.201 | 0.97 | 0.92-1.02 | 28.18 | 0.08 |  |  |
|  | GCST90200480 | Inverse variance weighted | 21 | 0.013 | 0.97 | 0.95-0.99 | 28.19 | 0.105 | 0 | 0.919 |
|  |  | Weighted median | 21 | 0.022 | 0.96 | 0.93-0.99 |  |  |  |  |
|  |  | Simple mode | 21 | 0.888 | 1 | 0.94-1.06 |  |  |  |  |
|  |  | Weighted mode | 21 | 0.082 | 0.97 | 0.93-1 |  |  |  |  |
|  |  |  |  |  |  |  |  |  |  |  |
| Unknown | X-12740 levels | MR Egger | 12 | 0.394 | 1.04 | 0.96-1.12 | 9.6 | 0.476 |  |  |
|  | GCST90200497 | Inverse variance weighted | 12 | 0.001 | 1.05 | 1.02-1.08 | 9.71 | 0.556 | 0 | 0.74 |
|  |  | Weighted median | 12 | 0.021 | 1.05 | 1.01-1.1 |  |  |  |  |
|  |  | Simple mode | 12 | 0.576 | 1.02 | 0.95-1.1 |  |  |  |  |
|  |  | Weighted mode | 12 | 0.696 | 1.01 | 0.95-1.09 |  |  |  |  |
|  |  |  |  |  |  |  |  |  |  |  |
| Unknown | X-13728 levels | MR Egger | 14 | 0.459 | 1.02 | 0.96-1.09 | 9.63 | 0.649 |  |  |
|  | GCST90200522 | Inverse variance weighted | 14 | 0.034 | 1.03 | 1-1.07 | 9.71 | 0.717 | 0 | 0.778 |
|  |  | Weighted median | 14 | 0.062 | 1.04 | 1-1.08 |  |  |  |  |
|  |  | Simple mode | 14 | 0.554 | 1.02 | 0.95-1.09 |  |  |  |  |
|  |  | Weighted mode | 14 | 0.386 | 1.03 | 0.96-1.1 |  |  |  |  |
|  |  |  |  |  |  |  |  |  |  |  |
| Unknown | X-18901 levels | MR Egger | 27 | 0.111 | 1.04 | 0.99-1.08 | 13.77 | 0.966 |  |  |
|  | GCST90200559 | Inverse variance weighted | 27 | 0.003 | 1.04 | 1.01-1.06 | 13.77 | 0.976 | 0 | 0.979 |
|  |  | Weighted median | 27 | 0.051 | 1.03 | 1-1.06 |  |  |  |  |
|  |  | Simple mode | 27 | 0.370 | 1.03 | 0.97-1.09 |  |  |  |  |
|  |  | Weighted mode | 27 | 0.363 | 1.03 | 0.97-1.09 |  |  |  |  |
|  |  |  |  |  |  |  |  |  |  |  |
| Unknown | X-18935 levels | MR Egger | 16 | 0.939 | 1 | 0.94-1.07 | 11.07 | 0.681 |  |  |
|  | GCST90200573 | Inverse variance weighted | 16 | 0.030 | 1.03 | 1-1.05 | 11.6 | 0.709 | 0 | 0.477 |
|  |  | Weighted median | 16 | 0.370 | 1.02 | 0.98-1.05 |  |  |  |  |
|  |  | Simple mode | 16 | 0.878 | 1 | 0.95-1.07 |  |  |  |  |
|  |  | Weighted mode | 16 | 0.795 | 1.01 | 0.95-1.06 |  |  |  |  |
|  |  |  |  |  |  |  |  |  |  |  |
| Unknown | X-21283 levels | MR Egger | 15 | 0.046 | 0.97 | 0.95-1 | 9.68 | 0.72 |  |  |
|  | GCST90200575 | Inverse variance weighted | 15 | 0.015 | 0.98 | 0.96-1 | 10.19 | 0.748 | 0 | 0.488 |
|  |  | Weighted median | 15 | 0.013 | 0.98 | 0.96-0.99 |  |  |  |  |
|  |  | Simple mode | 15 | 0.767 | 0.99 | 0.94-1.04 |  |  |  |  |
|  |  | Weighted mode | 15 | 0.021 | 0.97 | 0.96-0.99 |  |  |  |  |
|  |  |  |  |  |  |  |  |  |  |  |
| Unknown | X-24556 levels | MR Egger | 24 | 0.800 | 0.99 | 0.95-1.04 | 25.37 | 0.28 |  |  |
|  | GCST90200628 | Inverse variance weighted | 24 | 0.003 | 0.97 | 0.95-0.99 | 26.86 | 0.262 | 0 | 0.269 |
|  |  | Weighted median | 24 | 0.077 | 0.97 | 0.94-1 |  |  |  |  |
|  |  | Simple mode | 24 | 0.164 | 0.96 | 0.91-1.01 |  |  |  |  |
|  |  | Weighted mode | 24 | 0.134 | 0.97 | 0.94-1.01 |  |  |  |  |
|  |  |  |  |  |  |  |  |  |  |  |
| Unknown | X-24307 levels | MR Egger | 13 | 0.509 | 1.03 | 0.95-1.12 | 8.14 | 0.701 |  |  |
|  | GCST90200632 | Inverse variance weighted | 13 | 0.027 | 1.04 | 1-1.07 | 8.18 | 0.771 | 0 | 0.837 |
|  |  | Weighted median | 13 | 0.443 | 1.02 | 0.98-1.06 |  |  |  |  |
|  |  | Simple mode | 13 | 0.799 | 1.01 | 0.94-1.08 |  |  |  |  |
|  |  | Weighted mode | 13 | 0.784 | 1.01 | 0.94-1.08 |  |  |  |  |
|  |  |  |  |  |  |  |  |  |  |  |
| Unknown | X-24951 levels | MR Egger | 19 | 0.151 | 1.06 | 0.98-1.15 | 25.65 | 0.081 |  |  |
|  | GCST90200643 | Inverse variance weighted | 19 | 0.006 | 1.05 | 1.01-1.08 | 25.82 | 0.104 | 0 | 0.743 |
|  |  | Weighted median | 19 | 0.062 | 1.04 | 1-1.09 |  |  |  |  |
|  |  | Simple mode | 19 | 0.577 | 1.03 | 0.94-1.12 |  |  |  |  |
|  |  | Weighted mode | 19 | 0.630 | 1.02 | 0.94-1.12 |  |  |  |  |
|  |  |  |  |  |  |  |  |  |  |  |
| Unknown | X-24565 levels | MR Egger | 28 | 0.177 | 0.98 | 0.95-1.01 | 18.39 | 0.861 |  |  |
|  | GCST90200645 | Inverse variance weighted | 28 | 0.006 | 0.98 | 0.96-0.99 | 18.39 | 0.891 | 0 | 0.98 |
|  |  | Weighted median | 28 | 0.063 | 0.98 | 0.96-1 |  |  |  |  |
|  |  | Simple mode | 28 | 0.372 | 0.98 | 0.94-1.02 |  |  |  |  |
|  |  | Weighted mode | 28 | 0.347 | 0.98 | 0.94-1.02 |  |  |  |  |
|  |  |  |  |  |  |  |  |  |  |  |
| Unknown | X-25422 levels | MR Egger | 20 | 0.105 | 1.05 | 0.99-1.11 | 22.61 | 0.206 |  |  |
|  | GCST90200661 | Inverse variance weighted | 20 | 0.001 | 1.04 | 1.02-1.07 | 22.72 | 0.25 | 0 | 0.777 |
|  |  | Weighted median | 20 | 0.016 | 1.04 | 1.01-1.07 |  |  |  |  |
|  |  | Simple mode | 20 | 0.191 | 1.04 | 0.98-1.1 |  |  |  |  |
|  |  | Weighted mode | 20 | 0.029 | 1.04 | 1.01-1.07 |  |  |  |  |

Table 1:Two MR Models estimated causality between 1400 metabolites and their ratio and major depressive disorder, and tested for heterogeneity and level pleiotropy.

| CLASS | Metabolite name and number | Method | SNP(n) | pval | OR | 95%CI | heterogeneity | | pleiotropy | |
| --- | --- | --- | --- | --- | --- | --- | --- | --- | --- | --- |
|  |  |  |  |  |  |  | Qvalue | P | intercept | P |
| Lipids | 1-stearoyl-2-arachidonoyl-GPI (18:0/20:4) levels | MR Egger | 19 | 0.220 | 1.03 | 0.99-1.07 | 14.36 | 0.641 |  |  |
|  | GCST90199649 | Inverse variance weighted | 19 | 0.026 | 1.02 | 1-1.04 | 14.46 | 0.698 | 0.00 | 0.756 |
| Lipids | Alpha-hydroxyisocaproate levels | MR Egger | 22 | 0.820 | 0.99 | 0.91-1.08 | 33.89 | 0.027 |  |  |
|  | GCST90199658 | Inverse variance weighted | 22 | 0.042 | 0.97 | 0.93-1 | 34.53 | 0.032 | 0.00 | 0.548 |
| Lipids | 2-linoleoylglycerol (18:2) levels | MR Egger | 19 | 0.096 | 0.95 | 0.9-1.01 | 6.81 | 0.986 |  |  |
|  | GCST90199685 | Inverse variance weighted | 19 | 0.003 | 0.96 | 0.94-0.99 | 7.10 | 0.989 | 0.00 | 0.598 |
| Lipids | Docosatrienoate (22:3n3) levels | MR Egger | 21 | 0.096 | 0.96 | 0.92-1 | 21.25 | 0.323 |  |  |
|  | GCST90199710 | Inverse variance weighted | 21 | 0.003 | 0.97 | 0.95-0.99 | 21.48 | 0.370 | 0.00 | 0.657 |
| Lipids | 1-linoleoyl-gpc (18:2) levels | MR Egger | 22 | 0.088 | 0.95 | 0.89-1.01 | 23.20 | 0.279 |  |  |
|  | GCST90199742 | Inverse variance weighted | 22 | 0.005 | 0.96 | 0.94-0.99 | 23.63 | 0.311 | 0.00 | 0.549 |
| Lipids | 1-stearoyl-GPE (18:0) levels | MR Egger | 36 | 0.422 | 1.02 | 0.97-1.08 | 41.79 | 0.168 |  |  |
|  | GCST90199772 | Inverse variance weighted | 36 | 0.003 | 1.03 | 1.01-1.05 | 41.92 | 0.196 | 0.00 | 0.754 |
| Lipids | Malonylcarnitine levels | MR Egger | 20 | 0.472 | 1.02 | 0.96-1.09 | 21.48 | 0.256 |  |  |
|  | GCST90199776 | Inverse variance weighted | 20 | 0.035 | 1.03 | 1-1.05 | 21.48 | 0.311 | 0.00 | 0.957 |
| Lipids | 2-hydroxy-3-methylvalerate levels | MR Egger | 28 | 0.011 | 0.92 | 0.87-0.98 | 31.32 | 0.217 |  |  |
|  | GCST90199786 | Inverse variance weighted | 28 | 0.034 | 0.97 | 0.95-1 | 36.17 | 0.112 | 0.01 | 0.055 |
| Lipids | 1-arachidonoyl-gpc (20:4n6) levels | MR Egger | 24 | 0.043 | 1.03 | 1-1.06 | 37.47 | 0.021 |  |  |
|  | GCST90199788 | Inverse variance weighted | 24 | 0.041 | 1.02 | 1-1.04 | 39.39 | 0.018 | 0.00 | 0.300 |
| Lipids | Pregnanediol-3-glucuronide levels | MR Egger | 30 | 0.037 | 0.96 | 0.92-1 | 30.21 | 0.353 |  |  |
|  | GCST90199917 | Inverse variance weighted | 30 | 0.002 | 0.97 | 0.96-0.99 | 31.17 | 0.357 | 0.00 | 0.353 |
| Lipids | 1-stearoyl-2-linoleoyl-gpc (18:0/18:2) levels | MR Egger | 19 | 0.359 | 0.97 | 0.9-1.04 | 29.75 | 0.028 |  |  |
|  | GCST90200037 | Inverse variance weighted | 19 | 0.037 | 0.97 | 0.94-1 | 29.76 | 0.040 | 0.00 | 0.951 |
| Lipids | 1-palmitoyl-2-docosahexaenoyl-gpc (16:0/22:6) levels | MR Egger | 21 | 0.091 | 1.07 | 0.99-1.14 | 21.53 | 0.308 |  |  |
|  | GCST90200043 | Inverse variance weighted | 21 | 0.029 | 1.03 | 1-1.05 | 22.93 | 0.292 | -0.01 | 0.280 |
| Lipids | 1-palmitoyl-2-arachidonoyl-GPE (16:0/20:4) levels | MR Egger | 17 | 0.645 | 1.01 | 0.97-1.05 | 22.26 | 0.101 |  |  |
|  | GCST90200054 | Inverse variance weighted | 17 | 0.025 | 1.03 | 1-1.05 | 23.40 | 0.103 | 0.00 | 0.395 |
| Lipids | 1-(1-enyl-palmitoyl)-2-palmitoleoyl-GPC (P-16:0/16:1) levels | MR Egger | 31 | 0.146 | 1.03 | 0.99-1.07 | 38.52 | 0.111 |  |  |
|  | GCST90200070 | Inverse variance weighted | 31 | 0.038 | 1.02 | 1-1.04 | 38.92 | 0.128 | 0.00 | 0.588 |
| Lipids | 1-oleoyl-2-linoleoyl-GPE (18:1/18:2) levels | MR Egger | 25 | 0.037 | 0.97 | 0.94-1 | 25.82 | 0.310 |  |  |
|  | GCST90200082 | Inverse variance weighted | 25 | 0.021 | 0.98 | 0.97-1 | 27.23 | 0.294 | 0.00 | 0.274 |
| Lipids | 1-linoleoyl-2-linolenoyl-GPC (18:2/18:3) levels | MR Egger | 16 | 0.046 | 0.94 | 0.89-0.99 | 15.91 | 0.319 |  |  |
|  | GCST90200095 | Inverse variance weighted | 16 | 0.017 | 0.97 | 0.94-0.99 | 17.62 | 0.283 | 0.00 | 0.240 |
| Lipids | Linoleoyl-arachidonoyl-glycerol (18:2/20:4) [1] levels | MR Egger | 15 | 0.077 | 1.04 | 1-1.08 | 16.44 | 0.226 |  |  |
|  | GCST90200103 | Inverse variance weighted | 15 | 0.013 | 1.03 | 1.01-1.05 | 17.11 | 0.250 | 0.00 | 0.479 |
| Lipids | Glycosyl-N-tricosanoyl-sphingadienine (d18:2/23:0) levels | MR Egger | 22 | 0.563 | 0.98 | 0.94-1.04 | 20.76 | 0.411 |  |  |
|  | GCST90200114 | Inverse variance weighted | 22 | 0.007 | 0.97 | 0.95-0.99 | 21.14 | 0.450 | 0.00 | 0.553 |
| Lipids | Taurodeoxycholate levels | MR Egger | 19 | 0.977 | 1.00 | 0.94-1.06 | 14.42 | 0.637 |  |  |
|  | GCST90200334 | Inverse variance weighted | 19 | 0.032 | 0.97 | 0.95-1 | 15.54 | 0.625 | 0.00 | 0.305 |
| Lipids | Methylsuccinate levels | MR Egger | 18 | 0.004 | 1.05 | 1.02-1.09 | 11.42 | 0.783 |  |  |
|  | GCST90200352 | Inverse variance weighted | 18 | 0.007 | 1.03 | 1.01-1.05 | 15.91 | 0.53 | -0.01 | 0.05 |
| Lipids | Cholesterol levels | MR Egger | 18 | 0.167 | 0.96 | 0.91-1.01 | 10.01 | 0.866 |  |  |
|  | GCST90200368 | Inverse variance weighted | 18 | 0.011 | 0.96 | 0.94-0.99 | 10.06 | 0.901 | 0 | 0.829 |
| Lipids | 1-palmitoyl-2-arachidonoyl-gpc (16:0/20:4n6) levels | MR Egger | 28 | 0.115 | 1.02 | 1-1.05 | 46.92 | 0.007 |  |  |
|  | GCST90200692 | Inverse variance weighted | 28 | 0.016 | 1.02 | 1-1.04 | 46.93 | 0.01 | 0 | 0.955 |
| Amino acids | N-acetylglutamate levels | MR Egger | 19 | 0.977 | 1.00 | 0.94-1.07 | 17.56 | 0.417 |  |  |
|  | GCST90199637 | Inverse variance weighted | 19 | 0.007 | 0.96 | 0.94-0.99 | 18.99 | 0.392 | 0.00 | 0.256 |
| Amino acids | N-acetylhistidine levels | MR Egger | 20 | 0.039 | 0.98 | 0.96-1 | 18.02 | 0.454 |  |  |
|  | GCST90199735 | Inverse variance weighted | 20 | 0.024 | 0.98 | 0.97-1 | 18.95 | 0.460 | 0.00 | 0.349 |
| Amino acids | N6-acetyllysine levels | MR Egger | 17 | 0.031 | 0.96 | 0.92-0.99 | 24.80 | 0.053 |  |  |
|  | GCST90199826 | Inverse variance weighted | 17 | 0.002 | 0.96 | 0.94-0.99 | 25.08 | 0.068 | 0.00 | 0.690 |
| Amino acids | 6-oxopiperidine-2-carboxylate levels | MR Egger | 22 | 0.075 | 0.97 | 0.93-1 | 27.22 | 0.129 |  |  |
|  | GCST90199949 | Inverse variance weighted | 22 | 0.003 | 0.97 | 0.95-0.99 | 27.22 | 0.164 | 0.00 | 0.967 |
| Amino acids | 5-oxoproline levels | MR Egger | 22 | 0.014 | 0.97 | 0.95-0.99 | 18.29 | 0.568 |  |  |
|  | GCST90200280 | Inverse variance weighted | 22 | 0.013 | 0.98 | 0.96-1 | 20.07 | 0.517 | 0.00 | 0.198 |
| Amino acids | Gamma-glutamyltyrosine levels | MR Egger | 25 | 0.492 | 1.02 | 0.97-1.07 | 13.91 | 0.930 |  |  |
|  | GCST90200295 | Inverse variance weighted | 25 | 0.040 | 1.02 | 1-1.05 | 14.00 | 0.947 | 0.00 | 0.761 |
| Amino acids | Glutamine levels | MR Egger | 24 | 0.045 | 1.05 | 1-1.09 | 22.3 | 0.442 |  |  |
|  | GCST90200419 | Inverse variance weighted | 24 | 0.005 | 1.03 | 1.01-1.05 | 23.11 | 0.454 | 0 | 0.379 |
| Keto acids | 2-oxoarginine levels | MR Egger | 20 | 0.699 | 0.98 | 0.91-1.07 | 21.38 | 0.261 |  |  |
|  | GCST90199903 | Inverse variance weighted | 20 | 0.015 | 0.97 | 0.94-0.99 | 21.60 | 0.305 | 0.00 | 0.668 |
| Keto acids | Alpha-ketobutyrate levels | MR Egger | 14 | 0.224 | 0.96 | 0.89-1.02 | 7.51 | 0.822 |  |  |
|  | GCST90200438 | Inverse variance weighted | 14 | 0.034 | 0.97 | 0.94-1 | 7.62 | 0.867 | 0 | 0.746 |
| Quinic acid | Quinate levels | MR Egger | 16 | 0.359 | 0.97 | 0.92-1.03 | 12.45 | 0.570 |  |  |
|  | GCST90199645 | Inverse variance weighted | 16 | 0.007 | 0.96 | 0.93-0.99 | 12.67 | 0.628 | 0.00 | 0.649 |
| Carboxylic acids | N-acetyl-beta-alanine levels | MR Egger | 23 | 0.059 | 1.04 | 1-1.09 | 14.03 | 0.868 |  |  |
|  | GCST90199866 | Inverse variance weighted | 23 | 0.003 | 1.03 | 1.01-1.06 | 14.31 | 0.890 | 0.00 | 0.606 |
| Naphthalenes | 2-naphthol sulfate levels | MR Egger | 20 | 0.061 | 0.94 | 0.89-1 | 14.18 | 0.717 |  |  |
|  | GCST90200213 | Inverse variance weighted | 20 | 0.045 | 0.97 | 0.95-1 | 15.65 | 0.680 | 0.00 | 0.241 |
| Purine nucleotides | Adenosine 5'-diphosphate (ADP) levels | MR Egger | 22 | 0.106 | 0.96 | 0.91-1.01 | 22.69 | 0.304 |  |  |
|  | GCST90200355 | Inverse variance weighted | 22 | 0.007 | 0.97 | 0.95-0.99 | 23.26 | 0.33 | 0 | 0.487 |
| Ratio | Adenosine 5'-diphosphate (ADP) to Adenosine 5'-monophosphate (AMP) ratio | MR Egger | 23 | 0.411 | 0.98 | 0.94-1.03 | 14.22 | 0.86 |  |  |
|  | GCST90200728 | Inverse variance weighted | 23 | 0.015 | 0.98 | 0.96-1 | 14.24 | 0.893 | 0 | 0.888 |
| Ratio | Arachidonate (20:4n6) to oleate to vaccenate (18:1) ratio | MR Egger | 18 | 0.071 | 1.03 | 1-1.07 | 25.37 | 0.063 |  |  |
|  | GCST90200740 | Inverse variance weighted | 18 | 0.004 | 1.03 | 1.01-1.05 | 25.47 | 0.085 | 0 | 0.811 |
| Ratio | Oleoyl-linoleoyl-glycerol (18:1 to 18:2) [2] to linoleoyl-arachidonoyl-glycerol (18:2 to 20:4) [2] ratio | MR Egger | 20 | 0.003 | 0.96 | 0.93-0.98 | 20.07 | 0.329 |  |  |
|  | GCST90200795 | Inverse variance weighted | 20 | 0.002 | 0.97 | 0.96-0.99 | 23.31 | 0.224 | 0 | 0.105 |
| Ratio | Adenosine 5'-monophosphate (AMP) to glutamine ratio | MR Egger | 16 | 0.056 | 1.07 | 1-1.14 | 8.18 | 0.88 |  |  |
|  | GCST90200848 | Inverse variance weighted | 16 | 0.041 | 1.03 | 1-1.06 | 9.73 | 0.837 | 0 | 0.234 |
| Ratio | Adenosine 5'-monophosphate (AMP) to asparagine ratio | MR Egger | 20 | 0.085 | 1.06 | 1-1.13 | 9.33 | 0.952 |  |  |
|  | GCST90200859 | Inverse variance weighted | 20 | 0.004 | 1.04 | 1.01-1.07 | 9.83 | 0.957 | 0 | 0.49 |
| Ratio | Adenosine 5'-monophosphate (AMP) to serine ratio | MR Egger | 15 | 0.350 | 1.04 | 0.96-1.11 | 11.82 | 0.543 |  |  |
|  | GCST90200860 | Inverse variance weighted | 15 | 0.046 | 1.03 | 1-1.06 | 11.85 | 0.619 | 0 | 0.867 |
| Ratio | Phosphate to serine ratio | MR Egger | 21 | 0.160 | 1.05 | 0.98-1.11 | 14.85 | 0.732 |  |  |
|  | GCST90200863 | Inverse variance weighted | 21 | 0.041 | 1.02 | 1-1.05 | 15.41 | 0.753 | 0 | 0.464 |
| Ratio | Methionine to phosphate ratio | MR Egger | 14 | 0.364 | 0.97 | 0.9-1.04 | 11.95 | 0.449 |  |  |
|  | GCST90200864 | Inverse variance weighted | 14 | 0.002 | 0.95 | 0.92-0.98 | 12.27 | 0.506 | 0 | 0.586 |
| Ratio | Adenosine 5'-monophosphate (AMP) to isoleucine ratio | MR Egger | 19 | 0.041 | 1.09 | 1.01-1.17 | 13.9 | 0.674 |  |  |
|  | GCST90200867 | Inverse variance weighted | 19 | 0.039 | 1.03 | 1-1.06 | 16.3 | 0.572 | -0.01 | 0.14 |
| Ratio | Phenylpyruvate to citrate ratio | MR Egger | 27 | 0.118 | 0.96 | 0.91-1.01 | 17.16 | 0.876 |  |  |
|  | GCST90200885 | Inverse variance weighted | 27 | 0.017 | 0.97 | 0.95-1 | 17.61 | 0.889 | 0 | 0.506 |
| Ratio | Cortisol to taurocholate ratio | MR Egger | 20 | 0.364 | 0.98 | 0.93-1.03 | 20.07 | 0.329 |  |  |
|  | GCST90200890 | Inverse variance weighted | 20 | 0.040 | 0.97 | 0.95-1 | 20.09 | 0.389 | 0 | 0.896 |
| Ratio | Alpha-ketoglutarate to proline ratio | MR Egger | 21 | 0.313 | 0.97 | 0.91-1.03 | 18.23 | 0.507 |  |  |
|  | GCST90200933 | Inverse variance weighted | 21 | 0.050 | 0.98 | 0.95-1 | 18.29 | 0.568 | 0 | 0.809 |
| Ratio | Phosphate to 5-oxoproline ratio | MR Egger | 24 | 0.194 | 1.02 | 0.99-1.05 | 29.15 | 0.141 |  |  |
|  | GCST90200968 | Inverse variance weighted | 24 | 0.010 | 1.03 | 1.01-1.05 | 29.52 | 0.164 | 0 | 0.602 |
| Ratio | Arachidonate (20:4n6) to linoleate (18:2n6) ratio | MR Egger | 20 | 0.041 | 1.04 | 1-1.07 | 24.95 | 0.126 |  |  |
|  | GCST90200979 | Inverse variance weighted | 20 | 0.001 | 1.03 | 1.01-1.05 | 25.08 | 0.158 | 0 | 0.761 |
| Ratio | Benzoate to linoleoyl-arachidonoyl-glycerol (18:2 to 20:4) [2] ratio | MR Egger | 19 | 0.067 | 0.96 | 0.91-1 | 23.01 | 0.149 |  |  |
|  | GCST90200990 | Inverse variance weighted | 19 | 0.011 | 0.97 | 0.95-0.99 | 23.96 | 0.156 | 0 | 0.412 |
| Ratio | Threonine to pyruvate ratio | MR Egger | 24 | 0.459 | 1.02 | 0.97-1.08 | 22.88 | 0.408 |  |  |
|  | GCST90201009 | Inverse variance weighted | 24 | 0.005 | 1.03 | 1.01-1.06 | 23.12 | 0.454 | 0 | 0.639 |
| Ratio | Androsterone glucuronide to etiocholanolone glucuronide ratio | MR Egger | 26 | 0.045 | 1.04 | 1-1.08 | 52.33 | 0.001 |  |  |
|  | GCST90201013 | Inverse variance weighted | 26 | 0.028 | 1.03 | 1-1.05 | 53.99 | 0.001 | 0 | 0.391 |
| Unknown | X-11470 levels | MR Egger | 30 | 0.177 | 1.02 | 0.99-1.05 | 41.08 | 0.053 |  |  |
|  | GCST90200470 | Inverse variance weighted | 30 | 0.019 | 1.02 | 1-1.04 | 41.12 | 0.067 | 0 | 0.867 |
| Unknown | X-11444 levels | MR Egger | 28 | 0.013 | 1.04 | 1.01-1.07 | 32.99 | 0.162 |  |  |
|  | GCST90200474 | Inverse variance weighted | 28 | 0.002 | 1.03 | 1.01-1.05 | 34.08 | 0.164 | 0 | 0.361 |
| Unknown | X-12410 levels | MR Egger | 21 | 0.201 | 0.97 | 0.92-1.02 | 28.18 | 0.08 |  |  |
|  | GCST90200480 | Inverse variance weighted | 21 | 0.013 | 0.97 | 0.95-0.99 | 28.19 | 0.105 | 0 | 0.919 |
| Unknown | X-12740 levels | MR Egger | 12 | 0.394 | 1.04 | 0.96-1.12 | 9.6 | 0.476 |  |  |
|  | GCST90200497 | Inverse variance weighted | 12 | 0.001 | 1.05 | 1.02-1.08 | 9.71 | 0.556 | 0 | 0.74 |
| Unknown | X-13728 levels | MR Egger | 14 | 0.459 | 1.02 | 0.96-1.09 | 9.63 | 0.649 |  |  |
|  | GCST90200522 | Inverse variance weighted | 14 | 0.034 | 1.03 | 1-1.07 | 9.71 | 0.717 | 0 | 0.778 |
| Unknown | X-18901 levels | MR Egger | 27 | 0.111 | 1.04 | 0.99-1.08 | 13.77 | 0.966 |  |  |
|  | GCST90200559 | Inverse variance weighted | 27 | 0.003 | 1.04 | 1.01-1.06 | 13.77 | 0.976 | 0 | 0.979 |
| Unknown | X-18935 levels | MR Egger | 16 | 0.939 | 1 | 0.94-1.07 | 11.07 | 0.681 |  |  |
|  | GCST90200573 | Inverse variance weighted | 16 | 0.030 | 1.03 | 1-1.05 | 11.6 | 0.709 | 0 | 0.477 |
| Unknown | X-21283 levels | MR Egger | 15 | 0.046 | 0.97 | 0.95-1 | 9.68 | 0.72 |  |  |
|  | GCST90200575 | Inverse variance weighted | 15 | 0.015 | 0.98 | 0.96-1 | 10.19 | 0.748 | 0 | 0.488 |
| Unknown | X-24556 levels | MR Egger | 24 | 0.800 | 0.99 | 0.95-1.04 | 25.37 | 0.28 |  |  |
|  | GCST90200628 | Inverse variance weighted | 24 | 0.003 | 0.97 | 0.95-0.99 | 26.86 | 0.262 | 0 | 0.269 |
| Unknown | X-24307 levels | MR Egger | 13 | 0.509 | 1.03 | 0.95-1.12 | 8.14 | 0.701 |  |  |
|  | GCST90200632 | Inverse variance weighted | 13 | 0.027 | 1.04 | 1-1.07 | 8.18 | 0.771 | 0 | 0.837 |
| Unknown | X-24951 levels | MR Egger | 19 | 0.151 | 1.06 | 0.98-1.15 | 25.65 | 0.081 |  |  |
|  | GCST90200643 | Inverse variance weighted | 19 | 0.006 | 1.05 | 1.01-1.08 | 25.82 | 0.104 | 0 | 0.743 |
| Unknown | X-24565 levels | MR Egger | 28 | 0.177 | 0.98 | 0.95-1.01 | 18.39 | 0.861 |  |  |
|  | GCST90200645 | Inverse variance weighted | 28 | 0.006 | 0.98 | 0.96-0.99 | 18.39 | 0.891 | 0 | 0.98 |
| Unknown | X-25422 levels | MR Egger | 20 | 0.105 | 1.05 | 0.99-1.11 | 22.61 | 0.206 |  |  |
|  | GCST90200661 | Inverse variance weighted | 20 | 0.001 | 1.04 | 1.02-1.07 | 22.72 | 0.25 | 0 | 0.777 |

The top ten metabolite enrichment pathways

|  | Total | Expected | Hits | Raw p | FDR |
| --- | --- | --- | --- | --- | --- |
| Valine, leucine and isoleucine biosynthesis | 8 | 0.040635 | 1 | 0.040007 | 1 |
| Ascorbate and aldarate metabolism | 9 | 0.045714 | 1 | 0.044908 | 1 |
| Arginine biosynthesis | 14 | 0.071111 | 1 | 0.069087 | 1 |
| D-Amino acid metabolism | 15 | 0.07619 | 1 | 0.073857 | 1 |
| Glycosylphosphatidylinositol (GPI)-anchor biosynthesis | 15 | 0.07619 | 1 | 0.073857 | 1 |
| Pentose and glucuronate interconversions | 19 | 0.096508 | 1 | 0.092728 | 1 |
| Propanoate metabolism | 22 | 0.11175 | 1 | 0.10666 | 1 |
| Glutathione metabolism | 28 | 0.14222 | 1 | 0.13396 | 1 |
| Glycine, serine and threonine metabolism | 33 | 0.16762 | 1 | 0.15615 | 1 |
| Cysteine and methionine metabolism | 33 | 0.16762 | 1 | 0.15615 | 1 |
