# Supplementary material for: Assessing the associations of 1,400 blood metabolites with major depressive disorder: a Mendelian randomization study
Source: Front Psychiatry. 2024 Jun 6;15:1391535. doi: 10.3389/fpsyt.2024.1391535 (PMC11187323; doi:10.3389/fpsyt.2024.1391535)
Supplement: Supplementary file 2 [file DataSheet_1.docx]

Catalogue

[1.Supplementary Table 1: Five MR Models estimated the causal relationship between 96 metabolites, or metabolite ratios, and MDD risk and tested for heterogeneity and horizontal pleiotropy](#_Toc304993060)

[2.Supplementary Table 2: Five MR Models estimated the causal relationship between 65 metabolites, or metabolite ratios, and MDD risk and tested for heterogeneity and horizontal pleiotropy.](#_Toc2122377678)

[3.Supplementary Table 3: Leave-One-Out plots of 96 metabolites](#_Toc1098257476)

[1.GCST90199637](#_Toc791453167)

[4.Supplementary Table 4: 50 significant SNPS were extracted as instrumental variables (IVs) for Major depressive Disorder (MDD)](#_Toc439668251)

1.Supplementary Table 1: Five MR Models estimated the causal relationship between 96 metabolites, or metabolite ratios, and MDD risk and tested for heterogeneity and horizontal pleiotropy

| \| method \| nsnp \| b \| se \| pval \| lo_ci \| up_ci \| or \| or_lci95 \| or_uci95 \| id \| \| --- \| --- \| --- \| --- \| --- \| --- \| --- \| --- \| --- \| --- \| --- \| \| MR Egger \| 19 \| -0.000970566 \| 0.03317678 \| 0.977002409 \| -0.065997054 \| 0.064055922 \| 0.999029905 \| 0.936133622 \| 1.066152018 \| GCST90199637 \| \| Weighted median \| 19 \| -0.022581091 \| 0.018360931 \| 0.218755303 \| -0.058568515 \| 0.013406333 \| 0.977671953 \| 0.94311362 \| 1.0134966 \| GCST90199637 \| \| Inverse variance weighted \| 19 \| -0.036599277 \| 0.013676237 \| 0.007447959 \| -0.063404702 \| -0.009793851 \| 0.96406238 \| 0.938563558 \| 0.990253953 \| GCST90199637 \| \| Simple mode \| 19 \| -0.018753669 \| 0.034307218 \| 0.591339266 \| -0.085995817 \| 0.048488479 \| 0.981421087 \| 0.91759807 \| 1.049683279 \| GCST90199637 \| \| Weighted mode \| 19 \| -0.018753669 \| 0.032638589 \| 0.572683424 \| -0.082725304 \| 0.045217966 \| 0.981421087 \| 0.920603999 \| 1.046255884 \| GCST90199637 \| \| MR Egger \| 16 \| -0.028482945 \| 0.030045041 \| 0.3592031 \| -0.087371226 \| 0.030405335 \| 0.97191887 \| 0.916336865 \| 1.030872298 \| GCST90199645 \| \| Weighted median \| 16 \| -0.042841156 \| 0.020970144 \| 0.041056444 \| -0.083942638 \| -0.001739673 \| 0.958063561 \| 0.919483998 \| 0.998261839 \| GCST90199645 \| \| Inverse variance weighted \| 16 \| -0.040617655 \| 0.014949142 \| 0.006586548 \| -0.069917973 \| -0.011317336 \| 0.960196186 \| 0.932470304 \| 0.988746464 \| GCST90199645 \| \| Simple mode \| 16 \| -0.047738299 \| 0.038331081 \| 0.232081709 \| -0.122867219 \| 0.02739062 \| 0.953383255 \| 0.884381084 \| 1.027769191 \| GCST90199645 \| \| Weighted mode \| 16 \| -0.046378947 \| 0.035615387 \| 0.212483941 \| -0.116185104 \| 0.023427211 \| 0.954680121 \| 0.890310408 \| 1.023703784 \| GCST90199645 \| \| MR Egger \| 19 \| 0.02724164 \| 0.021407674 \| 0.220319221 \| -0.014717402 \| 0.069200682 \| 1.027616086 \| 0.98539037 \| 1.071651249 \| GCST90199649 \| \| Weighted median \| 19 \| 0.024973962 \| 0.014346985 \| 0.081734154 \| -0.003146128 \| 0.053094052 \| 1.025288424 \| 0.996858816 \| 1.054528821 \| GCST90199649 \| \| Inverse variance weighted \| 19 \| 0.021170463 \| 0.00948264 \| 0.025578679 \| 0.002584487 \| 0.039756438 \| 1.021396147 \| 1.00258783 \| 1.040557303 \| GCST90199649 \| \| Simple mode \| 19 \| 0.006022382 \| 0.02631891 \| 0.821585844 \| -0.045562683 \| 0.057607446 \| 1.006040553 \| 0.95545971 \| 1.059299082 \| GCST90199649 \| \| Weighted mode \| 19 \| 0.046208462 \| 0.016472 \| 0.011703695 \| 0.013923342 \| 0.078493582 \| 1.047292709 \| 1.014020723 \| 1.081656413 \| GCST90199649 \| \| MR Egger \| 22 \| -0.010199719 \| 0.044311793 \| 0.820289355 \| -0.097050833 \| 0.076651395 \| 0.989852121 \| 0.907509873 \| 1.079665634 \| GCST90199658 \| \| Weighted median \| 22 \| -0.028263831 \| 0.019433396 \| 0.145836882 \| -0.066353288 \| 0.009825626 \| 0.972131855 \| 0.935800199 \| 1.009874056 \| GCST90199658 \| \| Inverse variance weighted \| 22 \| -0.035065555 \| 0.017248245 \| 0.04205327 \| -0.068872116 \| -0.001258994 \| 0.965542118 \| 0.933446045 \| 0.998741798 \| GCST90199658 \| \| Simple mode \| 22 \| -0.027996323 \| 0.035963857 \| 0.444982961 \| -0.098485483 \| 0.042492838 \| 0.972391943 \| 0.906208848 \| 1.043408583 \| GCST90199658 \| \| Weighted mode \| 22 \| -0.026952447 \| 0.032419797 \| 0.415124929 \| -0.090495249 \| 0.036590355 \| 0.973407529 \| 0.913478674 \| 1.037268022 \| GCST90199658 \| \| MR Egger \| 19 \| -0.050013073 \| 0.028350583 \| 0.095676678 \| -0.105580216 \| 0.00555407 \| 0.951216989 \| 0.899802292 \| 1.005569522 \| GCST90199685 \| \| Weighted median \| 19 \| -0.039630252 \| 0.015950363 \| 0.012969754 \| -0.070892963 \| -0.00836754 \| 0.961144755 \| 0.931561598 \| 0.99166737 \| GCST90199685 \| \| Inverse variance weighted \| 19 \| -0.036222428 \| 0.012111631 \| 0.002783257 \| -0.059961224 \| -0.012483632 \| 0.964425754 \| 0.941801052 \| 0.987593965 \| GCST90199685 \| \| Simple mode \| 19 \| -0.040576737 \| 0.027474802 \| 0.156992434 \| -0.094427349 \| 0.013273875 \| 0.960235476 \| 0.909893836 \| 1.013362364 \| GCST90199685 \| \| Weighted mode \| 19 \| -0.041422733 \| 0.026852211 \| 0.140320962 \| -0.094053068 \| 0.011207601 \| 0.959423464 \| 0.910234457 \| 1.011270641 \| GCST90199685 \| \| MR Egger \| 22 \| -0.013416221 \| 0.035976901 \| 0.713136803 \| -0.083930946 \| 0.057098504 \| 0.986673375 \| 0.919494748 \| 1.058760097 \| GCST90199697 \| \| Weighted median \| 22 \| 0.02372706 \| 0.017711253 \| 0.180355837 \| -0.010986995 \| 0.058441116 \| 1.024010787 \| 0.989073142 \| 1.060182556 \| GCST90199697 \| \| Inverse variance weighted \| 22 \| 0.039143831 \| 0.01566954 \| 0.012486651 \| 0.008431533 \| 0.069856129 \| 1.039920045 \| 1.008467178 \| 1.072353889 \| GCST90199697 \| \| Simple mode \| 22 \| 0.00786341 \| 0.031304003 \| 0.804104961 \| -0.053492435 \| 0.069219255 \| 1.007894408 \| 0.947913112 \| 1.071671153 \| GCST90199697 \| \| Weighted mode \| 22 \| 0.011249035 \| 0.030575499 \| 0.716622532 \| -0.048678943 \| 0.071177013 \| 1.011312543 \| 0.952486883 \| 1.07377128 \| GCST90199697 \| \| MR Egger \| 18 \| -0.034081283 \| 0.014379345 \| 0.030685841 \| -0.062264799 \| -0.005897767 \| 0.966492942 \| 0.93963404 \| 0.994119591 \| GCST90199706 \| \| Weighted median \| 18 \| -0.027069184 \| 0.007991002 \| 0.000705436 \| -0.042731548 \| -0.011406819 \| 0.973293903 \| 0.958168577 \| 0.988657992 \| GCST90199706 \| \| Inverse variance weighted \| 18 \| -0.023376803 \| 0.008997423 \| 0.009372299 \| -0.041011752 \| -0.005741855 \| 0.976894317 \| 0.959817851 \| 0.994274598 \| GCST90199706 \| \| Simple mode \| 18 \| 0.019377259 \| 0.025220448 \| 0.452841435 \| -0.03005482 \| 0.068809337 \| 1.019566216 \| 0.970392335 \| 1.071231946 \| GCST90199706 \| \| Weighted mode \| 18 \| -0.027094649 \| 0.008115808 \| 0.003892535 \| -0.043001633 \| -0.011187665 \| 0.973269118 \| 0.957909826 \| 0.988874684 \| GCST90199706 \| \| MR Egger \| 21 \| -0.04071045 \| 0.023206974 \| 0.095505898 \| -0.086196118 \| 0.004775219 \| 0.960107089 \| 0.917414292 \| 1.004786639 \| GCST90199710 \| \| Weighted median \| 21 \| -0.047489229 \| 0.014975283 \| 0.001518241 \| -0.076840784 \| -0.018137675 \| 0.953620744 \| 0.926037282 \| 0.982025823 \| GCST90199710 \| \| Inverse variance weighted \| 21 \| -0.031457083 \| 0.010593201 \| 0.002982324 \| -0.052219757 \| -0.010694409 \| 0.969032544 \| 0.949120268 \| 0.989362573 \| GCST90199710 \| \| Simple mode \| 21 \| -0.023085731 \| 0.024520543 \| 0.357690113 \| -0.071145996 \| 0.024974534 \| 0.977178706 \| 0.931325913 \| 1.02528901 \| GCST90199710 \| \| Weighted mode \| 21 \| -0.04816769 \| 0.016385789 \| 0.008103685 \| -0.080283836 \| -0.016051545 \| 0.952973969 \| 0.92285437 \| 0.984076595 \| GCST90199710 \| \| MR Egger \| 14 \| 0.051941804 \| 0.037352648 \| 0.18960373 \| -0.021269386 \| 0.125152994 \| 1.053314442 \| 0.978955212 \| 1.133321832 \| GCST90199712 \| \| Weighted median \| 14 \| -0.052462658 \| 0.02145765 \| 0.014487641 \| -0.094519652 \| -0.010405664 \| 0.948889754 \| 0.909809855 \| 0.989648288 \| GCST90199712 \| \| Inverse variance weighted \| 14 \| -0.031915505 \| 0.016076005 \| 0.0471124 \| -0.063424474 \| -0.000406536 \| 0.96858842 \| 0.938545001 \| 0.999593547 \| GCST90199712 \| \| Simple mode \| 14 \| -0.055686773 \| 0.037910764 \| 0.165641761 \| -0.129991871 \| 0.018618325 \| 0.945835351 \| 0.878102569 \| 1.018792727 \| GCST90199712 \| \| Weighted mode \| 14 \| -0.052400261 \| 0.034406241 \| 0.151711133 \| -0.119836494 \| 0.015035971 \| 0.948948963 \| 0.887065466 \| 1.01514958 \| GCST90199712 \| \| MR Egger \| 20 \| -0.023441995 \| 0.010540804 \| 0.039190755 \| -0.044101971 \| -0.00278202 \| 0.976830634 \| 0.956856381 \| 0.997221846 \| GCST90199735 \| \| Weighted median \| 20 \| -0.020807486 \| 0.009943667 \| 0.036390564 \| -0.040297074 \| -0.001317897 \| 0.979407497 \| 0.960504056 \| 0.998682971 \| GCST90199735 \| \| Inverse variance weighted \| 20 \| -0.015909561 \| 0.007051065 \| 0.024049696 \| -0.029729648 \| -0.002089473 \| 0.984216328 \| 0.970707931 \| 0.997912708 \| GCST90199735 \| \| Simple mode \| 20 \| -0.004151047 \| 0.020587683 \| 0.842350796 \| -0.044502906 \| 0.036200812 \| 0.995857557 \| 0.95647282 \| 1.03686404 \| GCST90199735 \| \| Weighted mode \| 20 \| -0.018274394 \| 0.008379173 \| 0.041961233 \| -0.034697573 \| -0.001851215 \| 0.98189157 \| 0.965897485 \| 0.998150498 \| GCST90199735 \| \| MR Egger \| 22 \| -0.054930688 \| 0.030617887 \| 0.087934909 \| -0.114941747 \| 0.00508037 \| 0.946550753 \| 0.89141807 \| 1.005093297 \| GCST90199742 \| \| Weighted median \| 22 \| -0.022468997 \| 0.018025926 \| 0.212587401 \| -0.057799811 \| 0.012861818 \| 0.977781551 \| 0.943838874 \| 1.012944887 \| GCST90199742 \| \| Inverse variance weighted \| 22 \| -0.038230081 \| 0.013488046 \| 0.004591647 \| -0.06466665 \| -0.011793511 \| 0.962491465 \| 0.937379887 \| 0.98827576 \| GCST90199742 \| \| Simple mode \| 22 \| -0.019150737 \| 0.037738513 \| 0.617121107 \| -0.093118221 \| 0.054816748 \| 0.981031473 \| 0.911085784 \| 1.056347019 \| GCST90199742 \| \| Weighted mode \| 22 \| -0.020563571 \| 0.043012294 \| 0.637528292 \| -0.104867668 \| 0.063740525 \| 0.979646417 \| 0.900443673 \| 1.065815811 \| GCST90199742 \| \| MR Egger \| 18 \| 0.069557721 \| 0.02466883 \| 0.012330669 \| 0.021206815 \| 0.117908628 \| 1.072033939 \| 1.021433277 \| 1.1251413 \| GCST90199770 \| \| Weighted median \| 18 \| 0.046973441 \| 0.015503777 \| 0.002447107 \| 0.016586037 \| 0.077360845 \| 1.048094173 \| 1.016724349 \| 1.080431875 \| GCST90199770 \| \| Inverse variance weighted \| 18 \| 0.032249126 \| 0.012544858 \| 0.010149181 \| 0.007661204 \| 0.056837048 \| 1.032774765 \| 1.007690627 \| 1.058483315 \| GCST90199770 \| \| Simple mode \| 18 \| -0.012750004 \| 0.037955684 \| 0.741042921 \| -0.087143145 \| 0.061643137 \| 0.987330933 \| 0.916545888 \| 1.063582723 \| GCST90199770 \| \| Weighted mode \| 18 \| 0.051394295 \| 0.016569132 \| 0.006479365 \| 0.018918795 \| 0.083869794 \| 1.0527379 \| 1.019098889 \| 1.087487288 \| GCST90199770 \| \| MR Egger \| 36 \| 0.022836353 \| 0.028091582 \| 0.421917135 \| -0.032223148 \| 0.077895853 \| 1.023099098 \| 0.968290486 \| 1.081010069 \| GCST90199772 \| \| Weighted median \| 36 \| 0.028427644 \| 0.014470734 \| 0.049473049 \| 6.50E-05 \| 0.056790283 \| 1.028835565 \| 1.000065006 \| 1.058433816 \| GCST90199772 \| \| Inverse variance weighted \| 36 \| 0.031089559 \| 0.010311034 \| 0.002568322 \| 0.010879932 \| 0.051299186 \| 1.031577887 \| 1.010939333 \| 1.052637781 \| GCST90199772 \| \| Simple mode \| 36 \| 0.045608441 \| 0.031752293 \| 0.159772431 \| -0.016626054 \| 0.107842936 \| 1.0466645 \| 0.983511396 \| 1.113872782 \| GCST90199772 \| \| Weighted mode \| 36 \| 0.034056826 \| 0.024870309 \| 0.179604756 \| -0.01468898 \| 0.082802633 \| 1.0346434 \| 0.985418376 \| 1.086327382 \| GCST90199772 \| \| MR Egger \| 20 \| 0.024550084 \| 0.033400918 \| 0.471800634 \| -0.040915716 \| 0.090015884 \| 1.024853919 \| 0.959910032 \| 1.094191664 \| GCST90199776 \| \| Weighted median \| 20 \| 0.024371051 \| 0.016935346 \| 0.150132313 \| -0.008822227 \| 0.057564328 \| 1.024670452 \| 0.991216575 \| 1.059253408 \| GCST90199776 \| \| Inverse variance weighted \| 20 \| 0.026231231 \| 0.012418528 \| 0.034663663 \| 0.001890916 \| 0.050571545 \| 1.026578297 \| 1.001892705 \| 1.051872117 \| GCST90199776 \| \| Simple mode \| 20 \| 0.013645231 \| 0.031398694 \| 0.668763162 \| -0.047896209 \| 0.075186671 \| 1.013738752 \| 0.953232719 \| 1.078085379 \| GCST90199776 \| \| Weighted mode \| 20 \| 0.011327356 \| 0.031503593 \| 0.723143303 \| -0.050419686 \| 0.073074398 \| 1.011391753 \| 0.950830291 \| 1.075810572 \| GCST90199776 \| \| MR Egger \| 28 \| -0.082916306 \| 0.030170966 \| 0.01074522 \| -0.1420514 \| -0.023781213 \| 0.920428178 \| 0.867576662 \| 0.976499332 \| GCST90199786 \| \| Weighted median \| 28 \| -0.004825392 \| 0.017215826 \| 0.779256459 \| -0.038568411 \| 0.028917627 \| 0.995186232 \| 0.96216588 \| 1.029339802 \| GCST90199786 \| \| Inverse variance weighted \| 28 \| -0.027733605 \| 0.013093163 \| 0.034160277 \| -0.053396204 \| -0.002071007 \| 0.97264744 \| 0.948004335 \| 0.997931136 \| GCST90199786 \| \| Simple mode \| 28 \| -0.060604935 \| 0.034846996 \| 0.093395556 \| -0.128905048 \| 0.007695177 \| 0.941194999 \| 0.87905743 \| 1.007724861 \| GCST90199786 \| \| Weighted mode \| 28 \| -0.009128936 \| 0.024133435 \| 0.708188463 \| -0.056430469 \| 0.038172596 \| 0.990912606 \| 0.945132198 \| 1.03891053 \| GCST90199786 \| \| MR Egger \| 24 \| 0.030111972 \| 0.013980383 \| 0.042468145 \| 0.00271042 \| 0.057513523 \| 1.030569922 \| 1.002714097 \| 1.059199595 \| GCST90199788 \| \| Weighted median \| 24 \| 0.026984802 \| 0.008418085 \| 0.00134793 \| 0.010485355 \| 0.043484249 \| 1.027352189 \| 1.010540519 \| 1.044443543 \| GCST90199788 \| \| Inverse variance weighted \| 24 \| 0.018978037 \| 0.009279228 \| 0.040833457 \| 0.000790749 \| 0.037165324 \| 1.019159264 \| 1.000791062 \| 1.037864591 \| GCST90199788 \| \| Simple mode \| 24 \| 0.007336673 \| 0.035034057 \| 0.835967725 \| -0.06133008 \| 0.076003425 \| 1.007363652 \| 0.940512744 \| 1.07896627 \| GCST90199788 \| \| Weighted mode \| 24 \| 0.026792038 \| 0.009213574 \| 0.007925086 \| 0.008733433 \| 0.044850643 \| 1.027154171 \| 1.00877168 \| 1.04587164 \| GCST90199788 \| \| MR Egger \| 17 \| -0.04488423 \| 0.01880247 \| 0.030583236 \| -0.08173707 \| -0.008031389 \| 0.956108165 \| 0.921514221 \| 0.992000776 \| GCST90199826 \| \| Weighted median \| 17 \| -0.039265149 \| 0.012945495 \| 0.00242045 \| -0.064638319 \| -0.013891978 \| 0.961495736 \| 0.937406444 \| 0.98620407 \| GCST90199826 \| \| Inverse variance weighted \| 17 \| -0.039257371 \| 0.012388624 \| 0.00153057 \| -0.063539074 \| -0.014975668 \| 0.961503214 \| 0.93843745 \| 0.985135909 \| GCST90199826 \| \| Simple mode \| 17 \| -0.065274974 \| 0.02560944 \| 0.021452478 \| -0.115469476 \| -0.015080472 \| 0.93680983 \| 0.890947767 \| 0.985032669 \| GCST90199826 \| \| Weighted mode \| 17 \| -0.041215929 \| 0.01255656 \| 0.004689757 \| -0.065826785 \| -0.016605072 \| 0.959621898 \| 0.93629303 \| 0.983532032 \| GCST90199826 \| \| MR Egger \| 20 \| -0.048467018 \| 0.030888907 \| 0.134042107 \| -0.109009276 \| 0.012075241 \| 0.952688761 \| 0.896722099 \| 1.012148441 \| GCST90199832 \| \| Weighted median \| 20 \| -0.052122997 \| 0.019265896 \| 0.006821109 \| -0.089884154 \| -0.01436184 \| 0.949212109 \| 0.914037067 \| 0.985740799 \| GCST90199832 \| \| Inverse variance weighted \| 20 \| -0.032425089 \| 0.013741363 \| 0.018291168 \| -0.05935816 \| -0.005492019 \| 0.968094968 \| 0.94236919 \| 0.994523035 \| GCST90199832 \| \| Simple mode \| 20 \| 0.01955187 \| 0.039811514 \| 0.628972668 \| -0.058478699 \| 0.097582438 \| 1.019744259 \| 0.943198331 \| 1.102502326 \| GCST90199832 \| \| Weighted mode \| 20 \| -0.065403587 \| 0.029144723 \| 0.036933714 \| -0.122527244 \| -0.00827993 \| 0.936689351 \| 0.884681802 \| 0.991754254 \| GCST90199832 \| \| MR Egger \| 23 \| 0.043627095 \| 0.02187005 \| 0.05920128 \| 0.000761797 \| 0.086492393 \| 1.044592748 \| 1.000762087 \| 1.090343073 \| GCST90199866 \| \| Weighted median \| 23 \| 0.04689882 \| 0.017598739 \| 0.007701201 \| 0.012405291 \| 0.081392349 \| 1.048015965 \| 1.012482555 \| 1.084796432 \| GCST90199866 \| \| Inverse variance weighted \| 23 \| 0.033916505 \| 0.011567962 \| 0.003368579 \| 0.0112433 \| 0.056589711 \| 1.034498228 \| 1.011306743 \| 1.058221545 \| GCST90199866 \| \| Simple mode \| 23 \| 0.035949511 \| 0.031328907 \| 0.263506414 \| -0.025455147 \| 0.09735417 \| 1.036603508 \| 0.974866103 \| 1.102250688 \| GCST90199866 \| \| Weighted mode \| 23 \| 0.046016709 \| 0.020352143 \| 0.033988229 \| 0.006126509 \| 0.08590691 \| 1.047091907 \| 1.006145314 \| 1.089704883 \| GCST90199866 \| \| MR Egger \| 14 \| -0.033946525 \| 0.046435386 \| 0.478781557 \| -0.124959883 \| 0.057066832 \| 0.966623193 \| 0.882532307 \| 1.058726565 \| GCST90199895 \| \| Weighted median \| 14 \| 0.036764135 \| 0.023420333 \| 0.116472622 \| -0.009139718 \| 0.082667988 \| 1.037448294 \| 0.990901922 \| 1.086181123 \| GCST90199895 \| \| Inverse variance weighted \| 14 \| 0.039794932 \| 0.019343804 \| 0.039662728 \| 0.001881076 \| 0.077708788 \| 1.040597359 \| 1.001882846 \| 1.080807869 \| GCST90199895 \| \| Simple mode \| 14 \| 0.038847065 \| 0.047870126 \| 0.431681177 \| -0.054978382 \| 0.132672511 \| 1.039611478 \| 0.94650561 \| 1.141875986 \| GCST90199895 \| \| Weighted mode \| 14 \| 0.049212767 \| 0.046396594 \| 0.308136719 \| -0.041724557 \| 0.140150091 \| 1.050443827 \| 0.959133931 \| 1.150446458 \| GCST90199895 \| \| MR Egger \| 20 \| -0.016565277 \| 0.04208464 \| 0.698486574 \| -0.099051171 \| 0.065920617 \| 0.983571173 \| 0.905696361 \| 1.068141921 \| GCST90199903 \| \| Weighted median \| 20 \| -0.020944995 \| 0.018965471 \| 0.269430438 \| -0.058117318 \| 0.016227328 \| 0.979272828 \| 0.943539247 \| 1.016359706 \| GCST90199903 \| \| Inverse variance weighted \| 20 \| -0.033817343 \| 0.013871517 \| 0.014772941 \| -0.061005515 \| -0.00662917 \| 0.966748072 \| 0.940818051 \| 0.993392754 \| GCST90199903 \| \| Simple mode \| 20 \| -0.068472254 \| 0.03689183 \| 0.079027968 \| -0.140780241 \| 0.003835734 \| 0.93381937 \| 0.868680191 \| 1.0038431 \| GCST90199903 \| \| Weighted mode \| 20 \| -0.054679213 \| 0.033237179 \| 0.116387746 \| -0.119824084 \| 0.010465658 \| 0.946788817 \| 0.887076474 \| 1.010520614 \| GCST90199903 \| \| MR Egger \| 30 \| -0.045393257 \| 0.020732447 \| 0.037058225 \| -0.086028854 \| -0.00475766 \| 0.955621603 \| 0.917567755 \| 0.99525364 \| GCST90199917 \| \| Weighted median \| 30 \| -0.023840823 \| 0.012732803 \| 0.061152126 \| -0.048797117 \| 0.001115471 \| 0.976441124 \| 0.95237433 \| 1.001116093 \| GCST90199917 \| \| Inverse variance weighted \| 30 \| -0.027774402 \| 0.00901378 \| 0.002060804 \| -0.045441411 \| -0.010107392 \| 0.972607761 \| 0.955575587 \| 0.989943516 \| GCST90199917 \| \| Simple mode \| 30 \| -0.02419539 \| 0.023904258 \| 0.31982346 \| -0.071047735 \| 0.022656955 \| 0.976094972 \| 0.93141743 \| 1.022915573 \| GCST90199917 \| \| Weighted mode \| 30 \| -0.031195253 \| 0.016049885 \| 0.061699489 \| -0.062653028 \| 0.000262523 \| 0.969286299 \| 0.939269317 \| 1.000262557 \| GCST90199917 \| \| MR Egger \| 22 \| -0.034187249 \| 0.018196483 \| 0.074928911 \| -0.069852356 \| 0.001477858 \| 0.966390532 \| 0.932531493 \| 1.00147895 \| GCST90199949 \| \| Weighted median \| 22 \| -0.035129711 \| 0.013849677 \| 0.011196661 \| -0.062275077 \| -0.007984345 \| 0.965480175 \| 0.939624382 \| 0.992047446 \| GCST90199949 \| \| Inverse variance weighted \| 22 \| -0.033603621 \| 0.011232365 \| 0.002774487 \| -0.055619057 \| -0.011588185 \| 0.966954709 \| 0.945899401 \| 0.9884787 \| GCST90199949 \| \| Simple mode \| 22 \| -0.031180278 \| 0.030172553 \| 0.313172247 \| -0.090318482 \| 0.027957925 \| 0.969300813 \| 0.913640161 \| 1.028352416 \| GCST90199949 \| \| Weighted mode \| 22 \| -0.034105992 \| 0.014296746 \| 0.026550336 \| -0.062127614 \| -0.00608437 \| 0.966469061 \| 0.939762952 \| 0.993934103 \| GCST90199949 \| \| MR Egger \| 20 \| -0.001285952 \| 0.033187419 \| 0.969517679 \| -0.066333293 \| 0.063761389 \| 0.998714874 \| 0.93581891 \| 1.065838047 \| GCST90199990 \| \| Weighted median \| 20 \| -0.010258675 \| 0.019390372 \| 0.596763654 \| -0.048263804 \| 0.027746454 \| 0.989793766 \| 0.95288238 \| 1.028134972 \| GCST90199990 \| \| Inverse variance weighted \| 20 \| -0.031266091 \| 0.015150453 \| 0.039045532 \| -0.060960979 \| -0.001571204 \| 0.969217638 \| 0.940859953 \| 0.99843003 \| GCST90199990 \| \| Simple mode \| 20 \| 0.012854095 \| 0.040919013 \| 0.756841577 \| -0.06734717 \| 0.093055361 \| 1.012937064 \| 0.934870586 \| 1.097522494 \| GCST90199990 \| \| Weighted mode \| 20 \| 0.011970045 \| 0.036986322 \| 0.749750063 \| -0.060523146 \| 0.084463235 \| 1.012041973 \| 0.941271983 \| 1.088132839 \| GCST90199990 \| \| MR Egger \| 19 \| -0.033117108 \| 0.035091085 \| 0.358519365 \| -0.101895634 \| 0.035661418 \| 0.967425259 \| 0.903123802 \| 1.036304913 \| GCST90200037 \| \| Weighted median \| 19 \| -0.038757773 \| 0.017681548 \| 0.02838024 \| -0.073413606 \| -0.004101939 \| 0.961983699 \| 0.929216421 \| 0.995906462 \| GCST90200037 \| \| Inverse variance weighted \| 19 \| -0.031137605 \| 0.014925769 \| 0.036963746 \| -0.060392111 \| -0.001883099 \| 0.969342178 \| 0.94139533 \| 0.998118673 \| GCST90200037 \| \| Simple mode \| 19 \| -0.02020283 \| 0.036446709 \| 0.586187448 \| -0.091638381 \| 0.051232721 \| 0.97999988 \| 0.912435044 \| 1.052567819 \| GCST90200037 \| \| Weighted mode \| 19 \| -0.055194187 \| 0.017936236 \| 0.006494475 \| -0.090349209 \| -0.020039166 \| 0.94630137 \| 0.913612088 \| 0.980160284 \| GCST90200037 \| \| MR Egger \| 21 \| 0.064069124 \| 0.035969378 \| 0.090871192 \| -0.006430856 \| 0.134569105 \| 1.066166094 \| 0.993589778 \| 1.144043715 \| GCST90200043 \| \| Weighted median \| 21 \| 0.016426374 \| 0.017774981 \| 0.355419185 \| -0.018412588 \| 0.051265337 \| 1.016562029 \| 0.981755888 \| 1.05260215 \| GCST90200043 \| \| Inverse variance weighted \| 21 \| 0.026339035 \| 0.012033164 \| 0.028606276 \| 0.002754033 \| 0.049924037 \| 1.026688973 \| 1.002757828 \| 1.051191242 \| GCST90200043 \| \| Simple mode \| 21 \| 0.013244293 \| 0.031404633 \| 0.677720085 \| -0.048308788 \| 0.074797373 \| 1.013332387 \| 0.952839516 \| 1.077665765 \| GCST90200043 \| \| Weighted mode \| 21 \| 0.010506284 \| 0.032261411 \| 0.748063435 \| -0.052726081 \| 0.073738648 \| 1.010561668 \| 0.948639827 \| 1.076525417 \| GCST90200043 \| \| MR Egger \| 31 \| 0.034720642 \| 0.010953474 \| 0.003583855 \| 0.013251832 \| 0.056189451 \| 1.03533044 \| 1.013340027 \| 1.057798065 \| GCST90200052 \| \| Weighted median \| 31 \| 0.030197131 \| 0.008478512 \| 0.000368591 \| 0.013579247 \| 0.046815015 \| 1.030657688 \| 1.013671864 \| 1.04792814 \| GCST90200052 \| \| Inverse variance weighted \| 31 \| 0.017922552 \| 0.007059923 \| 0.011128662 \| 0.004085102 \| 0.031760001 \| 1.018084124 \| 1.004093457 \| 1.032269732 \| GCST90200052 \| \| Simple mode \| 31 \| -0.039087829 \| 0.028783818 \| 0.184596715 \| -0.095504112 \| 0.017328453 \| 0.961666243 \| 0.908914624 \| 1.017479462 \| GCST90200052 \| \| Weighted mode \| 31 \| 0.030090501 \| 0.008373169 \| 0.001150472 \| 0.013679089 \| 0.046501913 \| 1.030547795 \| 1.013773076 \| 1.047600083 \| GCST90200052 \| \| MR Egger \| 17 \| 0.009665822 \| 0.020565854 \| 0.645119767 \| -0.030643253 \| 0.049974896 \| 1.009712687 \| 0.969821493 \| 1.051244706 \| GCST90200054 \| \| Weighted median \| 17 \| 0.012644993 \| 0.011864614 \| 0.286525987 \| -0.010609651 \| 0.035899636 \| 1.012725279 \| 0.989446433 \| 1.036551809 \| GCST90200054 \| \| Inverse variance weighted \| 17 \| 0.024791242 \| 0.011074621 \| 0.025184339 \| 0.003084984 \| 0.046497499 \| 1.0251011 \| 1.003089747 \| 1.047595459 \| GCST90200054 \| \| Simple mode \| 17 \| 0.023311021 \| 0.029787832 \| 0.445309026 \| -0.03507313 \| 0.081695172 \| 1.023584846 \| 0.965534804 \| 1.085124983 \| GCST90200054 \| \| Weighted mode \| 17 \| 0.011318348 \| 0.012679855 \| 0.385287114 \| -0.013534169 \| 0.036170865 \| 1.011382643 \| 0.986557007 \| 1.03683299 \| GCST90200054 \| \| MR Egger \| 22 \| 0.023924686 \| 0.020760464 \| 0.262739278 \| -0.016765823 \| 0.064615196 \| 1.024213178 \| 0.983373941 \| 1.066748456 \| GCST90200058 \| \| Weighted median \| 22 \| 0.035520078 \| 0.014143187 \| 0.012023217 \| 0.007799432 \| 0.063240724 \| 1.036158452 \| 1.007829927 \| 1.065283247 \| GCST90200058 \| \| Inverse variance weighted \| 22 \| 0.024885114 \| 0.010086887 \| 0.013622156 \| 0.005114815 \| 0.044655412 \| 1.025197333 \| 1.005127918 \| 1.045667474 \| GCST90200058 \| \| Simple mode \| 22 \| -0.016242187 \| 0.035223938 \| 0.649458276 \| -0.085281105 \| 0.052796731 \| 0.983889006 \| 0.918254123 \| 1.054215334 \| GCST90200058 \| \| Weighted mode \| 22 \| 0.053599231 \| 0.015291663 \| 0.002106713 \| 0.02362757 \| 0.083570891 \| 1.055061681 \| 1.023908913 \| 1.087162282 \| GCST90200058 \| \| MR Egger \| 21 \| 0.033282002 \| 0.033390666 \| 0.331413652 \| -0.032163704 \| 0.098727709 \| 1.033842044 \| 0.968348047 \| 1.103765713 \| GCST90200062 \| \| Weighted median \| 21 \| 0.026043162 \| 0.018570299 \| 0.160793026 \| -0.010354624 \| 0.062440948 \| 1.026385248 \| 0.989698801 \| 1.064431601 \| GCST90200062 \| \| Inverse variance weighted \| 21 \| 0.029206339 \| 0.012196842 \| 0.016639321 \| 0.005300529 \| 0.05311215 \| 1.029637027 \| 1.005314601 \| 1.054547906 \| GCST90200062 \| \| Simple mode \| 21 \| -0.020217039 \| 0.033067237 \| 0.547827346 \| -0.085028825 \| 0.044594746 \| 0.979985955 \| 0.918485809 \| 1.045604039 \| GCST90200062 \| \| Weighted mode \| 21 \| -0.012743607 \| 0.041194247 \| 0.760252062 \| -0.09348433 \| 0.067997117 \| 0.987337249 \| 0.910752288 \| 1.070362222 \| GCST90200062 \| \| MR Egger \| 31 \| 0.027701952 \| 0.018553971 \| 0.14622571 \| -0.008663831 \| 0.064067734 \| 1.028089218 \| 0.991373592 \| 1.066164612 \| GCST90200070 \| \| Weighted median \| 31 \| 0.018237584 \| 0.012201675 \| 0.134998297 \| -0.005677698 \| 0.042152867 \| 1.018404905 \| 0.994338389 \| 1.043053915 \| GCST90200070 \| \| Inverse variance weighted \| 31 \| 0.018868104 \| 0.009097655 \| 0.038083729 \| 0.0010367 \| 0.036699509 \| 1.019047232 \| 1.001037237 \| 1.03738125 \| GCST90200070 \| \| Simple mode \| 31 \| 0.007919016 \| 0.027695112 \| 0.776892697 \| -0.046363404 \| 0.062201436 \| 1.007950455 \| 0.954694959 \| 1.064176687 \| GCST90200070 \| \| Weighted mode \| 31 \| 0.017490322 \| 0.012713434 \| 0.179088748 \| -0.007428009 \| 0.042408653 \| 1.017644173 \| 0.99259951 \| 1.043320748 \| GCST90200070 \| \| MR Egger \| 25 \| -0.031223024 \| 0.014075066 \| 0.036684266 \| -0.058810152 \| -0.003635895 \| 0.969259381 \| 0.942885757 \| 0.996370707 \| GCST90200082 \| \| Weighted median \| 25 \| -0.036750975 \| 0.011470908 \| 0.00135607 \| -0.059233955 \| -0.014267994 \| 0.963916145 \| 0.942486244 \| 0.985833312 \| GCST90200082 \| \| Inverse variance weighted \| 25 \| -0.018061403 \| 0.007812278 \| 0.020781798 \| -0.033373467 \| -0.002749339 \| 0.982100727 \| 0.967177283 \| 0.997254437 \| GCST90200082 \| \| Simple mode \| 25 \| -0.025334956 \| 0.023428187 \| 0.290271343 \| -0.071254202 \| 0.020584291 \| 0.974983281 \| 0.931225142 \| 1.020797609 \| GCST90200082 \| \| Weighted mode \| 25 \| -0.029294903 \| 0.011101119 \| 0.014378383 \| -0.051053097 \| -0.00753671 \| 0.971130033 \| 0.950228215 \| 0.99249162 \| GCST90200082 \| \| MR Egger \| 16 \| -0.063120034 \| 0.028769003 \| 0.045607689 \| -0.119507279 \| -0.006732789 \| 0.938830776 \| 0.887357549 \| 0.993289826 \| GCST90200095 \| \| Weighted median \| 16 \| -0.061522533 \| 0.017346704 \| 0.000390176 \| -0.095522073 \| -0.027522994 \| 0.940331757 \| 0.9088983 \| 0.972852313 \| GCST90200095 \| \| Inverse variance weighted \| 16 \| -0.031573286 \| 0.013186257 \| 0.016647192 \| -0.057418349 \| -0.005728223 \| 0.968919946 \| 0.944198982 \| 0.994288152 \| GCST90200095 \| \| Simple mode \| 16 \| -0.036228565 \| 0.041032909 \| 0.391212975 \| -0.116653066 \| 0.044195936 \| 0.964419836 \| 0.889893874 \| 1.045187124 \| GCST90200095 \| \| Weighted mode \| 16 \| -0.057980866 \| 0.019223602 \| 0.008682757 \| -0.095659126 \| -0.020302606 \| 0.943668003 \| 0.90877374 \| 0.979902104 \| GCST90200095 \| \| MR Egger \| 15 \| 0.03927657 \| 0.020446348 \| 0.076939541 \| -0.000798273 \| 0.079351413 \| 1.040058093 \| 0.999202046 \| 1.08258469 \| GCST90200103 \| \| Weighted median \| 15 \| 0.046482018 \| 0.013153288 \| 0.000409521 \| 0.020701574 \| 0.072262462 \| 1.047579241 \| 1.020917337 \| 1.074937438 \| GCST90200103 \| \| Inverse variance weighted \| 15 \| 0.026658744 \| 0.010710485 \| 0.012809133 \| 0.005666193 \| 0.047651295 \| 1.027017267 \| 1.005682276 \| 1.048804868 \| GCST90200103 \| \| Simple mode \| 15 \| 0.042870058 \| 0.027095359 \| 0.135928223 \| -0.010236845 \| 0.095976961 \| 1.043802252 \| 0.989815373 \| 1.100733704 \| GCST90200103 \| \| Weighted mode \| 15 \| 0.046994513 \| 0.013663004 \| 0.003986674 \| 0.020215025 \| 0.073774 \| 1.048116258 \| 1.020420733 \| 1.076563475 \| GCST90200103 \| \| MR Egger \| 22 \| -0.01516414 \| 0.025781838 \| 0.563000823 \| -0.065696542 \| 0.035368262 \| 0.984950257 \| 0.936414984 \| 1.036001159 \| GCST90200114 \| \| Weighted median \| 22 \| -0.03051869 \| 0.015461493 \| 0.048398641 \| -0.060823217 \| -0.000214164 \| 0.969942303 \| 0.940989576 \| 0.999785859 \| GCST90200114 \| \| Inverse variance weighted \| 22 \| -0.029230295 \| 0.010892615 \| 0.007285672 \| -0.050579821 \| -0.00788077 \| 0.971192777 \| 0.950678041 \| 0.992150202 \| GCST90200114 \| \| Simple mode \| 22 \| -0.009579066 \| 0.026073448 \| 0.717006298 \| -0.060683024 \| 0.041524892 \| 0.990466667 \| 0.941121505 \| 1.042399109 \| GCST90200114 \| \| Weighted mode \| 22 \| -0.03441228 \| 0.019672316 \| 0.09484568 \| -0.07297002 \| 0.00414546 \| 0.966173089 \| 0.9296287 \| 1.004154065 \| GCST90200114 \| \| MR Egger \| 23 \| -0.014980889 \| 0.022745224 \| 0.517284651 \| -0.059561527 \| 0.029599749 \| 0.985130766 \| 0.942177562 \| 1.030042176 \| GCST90200136 \| \| Weighted median \| 23 \| 0.016018025 \| 0.014362177 \| 0.264725193 \| -0.012131842 \| 0.044167892 \| 1.016147001 \| 0.987941452 \| 1.045157813 \| GCST90200136 \| \| Inverse variance weighted \| 23 \| 0.02451048 \| 0.010953717 \| 0.025244509 \| 0.003041195 \| 0.045979765 \| 1.024813331 \| 1.003045824 \| 1.047053223 \| GCST90200136 \| \| Simple mode \| 23 \| 0.033781434 \| 0.026439762 \| 0.214682302 \| -0.018040499 \| 0.085603367 \| 1.034358507 \| 0.982121257 \| 1.089374161 \| GCST90200136 \| \| Weighted mode \| 23 \| 0.016198325 \| 0.01929682 \| 0.410257595 \| -0.021623441 \| 0.054020092 \| 1.016330229 \| 0.978608669 \| 1.055505809 \| GCST90200136 \| \| MR Egger \| 22 \| -0.057416195 \| 0.031851134 \| 0.086535298 \| -0.119844418 \| 0.005012027 \| 0.944201016 \| 0.887058436 \| 1.005024609 \| GCST90200186 \| \| Weighted median \| 22 \| 0.025017857 \| 0.01778823 \| 0.1595972 \| -0.009847074 \| 0.059882788 \| 1.02533343 \| 0.99020125 \| 1.061712093 \| GCST90200186 \| \| Inverse variance weighted \| 22 \| 0.027855538 \| 0.014205875 \| 0.04989695 \| 1.20E-05 \| 0.055699053 \| 1.028247131 \| 1.000012023 \| 1.05727945 \| GCST90200186 \| \| Simple mode \| 22 \| -0.001753346 \| 0.040160901 \| 0.965589455 \| -0.080468711 \| 0.076962019 \| 0.99824819 \| 0.922683773 \| 1.080001056 \| GCST90200186 \| \| Weighted mode \| 22 \| 0.012058039 \| 0.036161033 \| 0.742098171 \| -0.058817586 \| 0.082933664 \| 1.01213103 \| 0.942878748 \| 1.086469734 \| GCST90200186 \| \| MR Egger \| 20 \| -0.057838321 \| 0.028941509 \| 0.061002316 \| -0.114563679 \| -0.001112964 \| 0.943802528 \| 0.89175515 \| 0.998887656 \| GCST90200213 \| \| Weighted median \| 20 \| -0.030947604 \| 0.018938649 \| 0.102238317 \| -0.068067356 \| 0.006172147 \| 0.969526371 \| 0.934197548 \| 1.006191234 \| GCST90200213 \| \| Inverse variance weighted \| 20 \| -0.026618408 \| 0.013269547 \| 0.044858683 \| -0.052626719 \| -0.000610096 \| 0.97373274 \| 0.948734091 \| 0.99939009 \| GCST90200213 \| \| Simple mode \| 20 \| -0.03606441 \| 0.038066722 \| 0.355329828 \| -0.110675186 \| 0.038546366 \| 0.964578163 \| 0.895229485 \| 1.039298915 \| GCST90200213 \| \| Weighted mode \| 20 \| -0.030601442 \| 0.035765414 \| 0.402871898 \| -0.100701653 \| 0.039498769 \| 0.969862042 \| 0.904202759 \| 1.040289218 \| GCST90200213 \| \| MR Egger \| 19 \| 0.01009175 \| 0.015651631 \| 0.527680169 \| -0.020585447 \| 0.040768946 \| 1.010142843 \| 0.979624987 \| 1.04161141 \| GCST90200268 \| \| Weighted median \| 19 \| -0.00631001 \| 0.012233631 \| 0.605999625 \| -0.030287928 \| 0.017667907 \| 0.993709856 \| 0.970166156 \| 1.017824908 \| GCST90200268 \| \| Inverse variance weighted \| 19 \| -0.023159171 \| 0.010385018 \| 0.025743729 \| -0.043513805 \| -0.002804536 \| 0.977106945 \| 0.957419336 \| 0.997199393 \| GCST90200268 \| \| Simple mode \| 19 \| -0.063979907 \| 0.032676402 \| 0.06591677 \| -0.128025655 \| 6.58E-05 \| 0.938023847 \| 0.879830807 \| 1.000065843 \| GCST90200268 \| \| Weighted mode \| 19 \| -0.006020846 \| 0.012345354 \| 0.631648542 \| -0.03021774 \| 0.018176048 \| 0.993997243 \| 0.970234251 \| 1.018342238 \| GCST90200268 \| \| MR Egger \| 22 \| -0.031356752 \| 0.011704058 \| 0.01442089 \| -0.054296705 \| -0.008416798 \| 0.969129773 \| 0.94715104 \| 0.991618524 \| GCST90200280 \| \| Weighted median \| 22 \| -0.024944245 \| 0.009697135 \| 0.010101617 \| -0.04395063 \| -0.00593786 \| 0.975364292 \| 0.957001204 \| 0.994079735 \| GCST90200280 \| \| Inverse variance weighted \| 22 \| -0.019979867 \| 0.007999917 \| 0.012506916 \| -0.035659704 \| -0.00430003 \| 0.980218408 \| 0.964968613 \| 0.995709202 \| GCST90200280 \| \| Simple mode \| 22 \| -0.03070482 \| 0.027122537 \| 0.270363475 \| -0.083864992 \| 0.022455352 \| 0.969761785 \| 0.919555395 \| 1.022709371 \| GCST90200280 \| \| Weighted mode \| 22 \| -0.025382393 \| 0.010155636 \| 0.020807886 \| -0.045287439 \| -0.005477346 \| 0.974937032 \| 0.95572273 \| 0.994537627 \| GCST90200280 \| \| MR Egger \| 25 \| 0.016683117 \| 0.023862564 \| 0.491478927 \| -0.030087509 \| 0.063453742 \| 1.016823057 \| 0.970360615 \| 1.065510196 \| GCST90200295 \| \| Weighted median \| 25 \| 0.024846719 \| 0.015698238 \| 0.11347362 \| -0.005921827 \| 0.055615265 \| 1.025157971 \| 0.994095672 \| 1.057190867 \| GCST90200295 \| \| Inverse variance weighted \| 25 \| 0.023148793 \| 0.011261758 \| 0.039828599 \| 0.001075747 \| 0.04522184 \| 1.023418806 \| 1.001076326 \| 1.046259936 \| GCST90200295 \| \| Simple mode \| 25 \| 0.027065692 \| 0.026846987 \| 0.323440959 \| -0.025554401 \| 0.079685786 \| 1.027435295 \| 0.974769349 \| 1.082946738 \| GCST90200295 \| \| Weighted mode \| 25 \| 0.028165917 \| 0.026299093 \| 0.294829053 \| -0.023380306 \| 0.07971214 \| 1.028566327 \| 0.976890896 \| 1.082975278 \| GCST90200295 \| \| MR Egger \| 21 \| -0.091926685 \| 0.032168026 \| 0.010071005 \| -0.154976015 \| -0.028877354 \| 0.912172023 \| 0.856435718 \| 0.971535612 \| GCST90200323 \| \| Weighted median \| 21 \| -0.03231097 \| 0.018902027 \| 0.087378399 \| -0.069358943 \| 0.004737003 \| 0.968205452 \| 0.932991729 \| 1.00474824 \| GCST90200323 \| \| Inverse variance weighted \| 21 \| -0.029372538 \| 0.014764548 \| 0.046657476 \| -0.058311052 \| -0.000434023 \| 0.971054643 \| 0.943356469 \| 0.999566071 \| GCST90200323 \| \| Simple mode \| 21 \| -0.029747619 \| 0.027747481 \| 0.296449212 \| -0.084132681 \| 0.024637444 \| 0.970690487 \| 0.919309273 \| 1.024943454 \| GCST90200323 \| \| Weighted mode \| 21 \| -0.032457447 \| 0.020094681 \| 0.121927967 \| -0.071843021 \| 0.006928126 \| 0.968063643 \| 0.930676981 \| 1.006952181 \| GCST90200323 \| \| MR Egger \| 19 \| -0.000841785 \| 0.029145089 \| 0.977294626 \| -0.057966159 \| 0.05628259 \| 0.99915857 \| 0.943681882 \| 1.057896593 \| GCST90200334 \| \| Weighted median \| 19 \| -0.013669313 \| 0.018793772 \| 0.46702256 \| -0.050505106 \| 0.02316648 \| 0.986423688 \| 0.950749074 \| 1.023436907 \| GCST90200334 \| \| Inverse variance weighted \| 19 \| -0.028342456 \| 0.013217601 \| 0.03200914 \| -0.054248955 \| -0.002435957 \| 0.972055424 \| 0.947196268 \| 0.997567007 \| GCST90200334 \| \| Simple mode \| 19 \| -0.007214077 \| 0.033227911 \| 0.830565302 \| -0.072340783 \| 0.057912629 \| 0.992811882 \| 0.930213841 \| 1.059622411 \| GCST90200334 \| \| Weighted mode \| 19 \| -0.007214077 \| 0.034011211 \| 0.834405481 \| -0.073876051 \| 0.059447897 \| 0.992811882 \| 0.928786809 \| 1.061250466 \| GCST90200334 \| \| MR Egger \| 18 \| 0.052586528 \| 0.015797158 \| 0.004252655 \| 0.021624099 \| 0.083548957 \| 1.053993758 \| 1.021859594 \| 1.087138437 \| GCST90200352 \| \| Weighted median \| 18 \| 0.039595843 \| 0.012140676 \| 0.001108557 \| 0.015800118 \| 0.063391569 \| 1.040390209 \| 1.0159256 \| 1.065443952 \| GCST90200352 \| \| Inverse variance weighted \| 18 \| 0.025835245 \| 0.009497702 \| 0.006525083 \| 0.007219749 \| 0.04445074 \| 1.026171867 \| 1.007245875 \| 1.045453477 \| GCST90200352 \| \| Simple mode \| 18 \| 0.015812557 \| 0.032848425 \| 0.636384713 \| -0.048570355 \| 0.08019547 \| 1.015938238 \| 0.952590318 \| 1.083498838 \| GCST90200352 \| \| Weighted mode \| 18 \| 0.04117087 \| 0.012146639 \| 0.003486196 \| 0.017363459 \| 0.064978282 \| 1.042030143 \| 1.01751508 \| 1.067135848 \| GCST90200352 \| \| MR Egger \| 22 \| -0.04452123 \| 0.026285049 \| 0.105823984 \| -0.096039926 \| 0.006997466 \| 0.956455294 \| 0.908427745 \| 1.007022006 \| GCST90200355 \| \| Weighted median \| 22 \| -0.032890592 \| 0.01392733 \| 0.018196939 \| -0.06018816 \| -0.005593024 \| 0.967644422 \| 0.941587348 \| 0.994422588 \| GCST90200355 \| \| Inverse variance weighted \| 22 \| -0.027407323 \| 0.010176206 \| 0.007075382 \| -0.047352686 \| -0.00746196 \| 0.97296485 \| 0.953750963 \| 0.992565811 \| GCST90200355 \| \| Simple mode \| 22 \| -0.041494599 \| 0.029274344 \| 0.17102173 \| -0.098872313 \| 0.015883115 \| 0.959354517 \| 0.905858367 \| 1.016009923 \| GCST90200355 \| \| Weighted mode \| 22 \| -0.040258589 \| 0.026024405 \| 0.136812532 \| -0.091266423 \| 0.010749244 \| 0.960541021 \| 0.912774495 \| 1.010807225 \| GCST90200355 \| \| MR Egger \| 27 \| 0.014461469 \| 0.023901621 \| 0.550604592 \| -0.032385709 \| 0.061308647 \| 1.014566542 \| 0.968133093 \| 1.063227026 \| GCST90200358 \| \| Weighted median \| 27 \| 0.049113355 \| 0.013850392 \| 0.000391141 \| 0.021966587 \| 0.076260123 \| 1.050339405 \| 1.022209628 \| 1.079243273 \| GCST90200358 \| \| Inverse variance weighted \| 27 \| 0.03445669 \| 0.012897903 \| 0.007551411 \| 0.009176799 \| 0.059736581 \| 1.035057199 \| 1.009219035 \| 1.061556875 \| GCST90200358 \| \| Simple mode \| 27 \| -0.024742238 \| 0.039794354 \| 0.539518783 \| -0.102739172 \| 0.053254696 \| 0.975561342 \| 0.902362304 \| 1.054698238 \| GCST90200358 \| \| Weighted mode \| 27 \| 0.049128043 \| 0.014242316 \| 0.001928233 \| 0.021213104 \| 0.077042982 \| 1.050354833 \| 1.021439701 \| 1.0800885 \| GCST90200358 \| \| MR Egger \| 18 \| -0.041960998 \| 0.028997169 \| 0.167185068 \| -0.098795448 \| 0.014873453 \| 0.95890718 \| 0.905927998 \| 1.014984613 \| GCST90200368 \| \| Weighted median \| 18 \| -0.041414508 \| 0.019549156 \| 0.034134387 \| -0.079730854 \| -0.003098162 \| 0.959431356 \| 0.923364833 \| 0.996906632 \| GCST90200368 \| \| Inverse variance weighted \| 18 \| -0.036427008 \| 0.01423814 \| 0.010515186 \| -0.064333762 \| -0.008520254 \| 0.964228473 \| 0.937691981 \| 0.991515941 \| GCST90200368 \| \| Simple mode \| 18 \| -0.053500803 \| 0.034938097 \| 0.144091054 \| -0.121979473 \| 0.014977867 \| 0.94790518 \| 0.885166539 \| 1.015090598 \| GCST90200368 \| \| Weighted mode \| 18 \| -0.048935314 \| 0.035262499 \| 0.183137386 \| -0.118049812 \| 0.020179185 \| 0.952242725 \| 0.888651786 \| 1.020384161 \| GCST90200368 \| \| MR Egger \| 24 \| 0.046369958 \| 0.021826896 \| 0.045111168 \| 0.003589241 \| 0.089150674 \| 1.047461856 \| 1.003595691 \| 1.093245368 \| GCST90200419 \| \| Weighted median \| 24 \| 0.043100829 \| 0.01577695 \| 0.006297294 \| 0.012178006 \| 0.074023652 \| 1.044043159 \| 1.01225246 \| 1.076832274 \| GCST90200419 \| \| Inverse variance weighted \| 24 \| 0.029193264 \| 0.010431723 \| 0.005133925 \| 0.008747086 \| 0.049639441 \| 1.029623564 \| 1.008785454 \| 1.05089212 \| GCST90200419 \| \| Simple mode \| 24 \| 0.0400697 \| 0.025616801 \| 0.13142754 \| -0.010139231 \| 0.09027863 \| 1.040883321 \| 0.989911998 \| 1.094479196 \| GCST90200419 \| \| Weighted mode \| 24 \| 0.044446658 \| 0.017482607 \| 0.018206101 \| 0.010180749 \| 0.078712567 \| 1.045449209 \| 1.010232749 \| 1.081893306 \| GCST90200419 \| \| MR Egger \| 14 \| -0.044473118 \| 0.034682808 \| 0.223961879 \| -0.112451421 \| 0.023505185 \| 0.956501312 \| 0.893640758 \| 1.023783609 \| GCST90200438 \| \| Weighted median \| 14 \| -0.019487354 \| 0.021001729 \| 0.35346316 \| -0.060650742 \| 0.021676035 \| 0.980701297 \| 0.941151887 \| 1.021912666 \| GCST90200438 \| \| Inverse variance weighted \| 14 \| -0.034306894 \| 0.016138383 \| 0.033520337 \| -0.065938126 \| -0.002675662 \| 0.966274915 \| 0.936188789 \| 0.997327914 \| GCST90200438 \| \| Simple mode \| 14 \| -0.015804595 \| 0.036081953 \| 0.668562235 \| -0.086525223 \| 0.054916033 \| 0.984319642 \| 0.917112416 \| 1.056451904 \| GCST90200438 \| \| Weighted mode \| 14 \| -0.015211633 \| 0.036881493 \| 0.686733136 \| -0.087499359 \| 0.057076093 \| 0.984903479 \| 0.916219459 \| 1.058736369 \| GCST90200438 \| \| MR Egger \| 17 \| -0.014001376 \| 0.029982286 \| 0.647219387 \| -0.072766657 \| 0.044763905 \| 0.986096187 \| 0.929817771 \| 1.045780927 \| GCST90200447 \| \| Weighted median \| 17 \| 0.021799119 \| 0.017583055 \| 0.215056806 \| -0.012663669 \| 0.056261907 \| 1.022038455 \| 0.987416178 \| 1.057874712 \| GCST90200447 \| \| Inverse variance weighted \| 17 \| 0.03115679 \| 0.014690067 \| 0.033926623 \| 0.002364259 \| 0.059949321 \| 1.031647243 \| 1.002367056 \| 1.061782735 \| GCST90200447 \| \| Simple mode \| 17 \| 0.027135492 \| 0.031296692 \| 0.398736825 \| -0.034206024 \| 0.088477009 \| 1.027507012 \| 0.966372388 \| 1.092509134 \| GCST90200447 \| \| Weighted mode \| 17 \| 0.024309884 \| 0.030513145 \| 0.43728709 \| -0.03549588 \| 0.084115647 \| 1.024607778 \| 0.965126711 \| 1.087754682 \| GCST90200447 \| \| MR Egger \| 28 \| 0.02890055 \| 0.028307066 \| 0.31667706 \| -0.0265813 \| 0.084382399 \| 1.029322223 \| 0.973768874 \| 1.088044882 \| GCST90200464 \| \| Weighted median \| 28 \| 0.008798129 \| 0.015447918 \| 0.56899316 \| -0.02147979 \| 0.039076048 \| 1.008836946 \| 0.978749258 \| 1.039849559 \| GCST90200464 \| \| Inverse variance weighted \| 28 \| 0.022996595 \| 0.011441518 \| 0.044439105 \| 0.00057122 \| 0.045421969 \| 1.023263055 \| 1.000571383 \| 1.046469345 \| GCST90200464 \| \| Simple mode \| 28 \| -0.000351416 \| 0.03048097 \| 0.990886132 \| -0.060094118 \| 0.059391285 \| 0.999648646 \| 0.941675901 \| 1.061190388 \| GCST90200464 \| \| Weighted mode \| 28 \| -0.003520508 \| 0.026021763 \| 0.893385466 \| -0.054523164 \| 0.047482148 \| 0.996485682 \| 0.946936574 \| 1.048627481 \| GCST90200464 \| \| MR Egger \| 30 \| 0.021700392 \| 0.015670659 \| 0.177056259 \| -0.009014099 \| 0.052414884 \| 1.021937558 \| 0.991026406 \| 1.053812861 \| GCST90200470 \| \| Weighted median \| 30 \| 0.015033926 \| 0.014153334 \| 0.288136719 \| -0.012706608 \| 0.042774461 \| 1.015147504 \| 0.98737378 \| 1.043702473 \| GCST90200470 \| \| Inverse variance weighted \| 30 \| 0.023696704 \| 0.010074159 \| 0.018661405 \| 0.003951353 \| 0.043442055 \| 1.023979702 \| 1.00395917 \| 1.044399475 \| GCST90200470 \| \| Simple mode \| 30 \| 0.046390753 \| 0.026277083 \| 0.088017876 \| -0.005112329 \| 0.097893836 \| 1.047483639 \| 0.994900717 \| 1.102845696 \| GCST90200470 \| \| Weighted mode \| 30 \| 0.011705434 \| 0.018107433 \| 0.523075663 \| -0.023785134 \| 0.047196002 \| 1.011774211 \| 0.976495503 \| 1.048327463 \| GCST90200470 \| \| MR Egger \| 28 \| 0.041412106 \| 0.015477568 \| 0.012734237 \| 0.011076073 \| 0.071748139 \| 1.042281547 \| 1.01113764 \| 1.074384714 \| GCST90200474 \| \| Weighted median \| 28 \| 0.023718434 \| 0.013951079 \| 0.089109372 \| -0.003625682 \| 0.051062549 \| 1.024001953 \| 0.996380883 \| 1.052388717 \| GCST90200474 \| \| Inverse variance weighted \| 28 \| 0.030443973 \| 0.009991247 \| 0.002310882 \| 0.010861129 \| 0.050026817 \| 1.03091213 \| 1.010920325 \| 1.051299289 \| GCST90200474 \| \| Simple mode \| 28 \| 0.027927505 \| 0.029844927 \| 0.357694658 \| -0.030568552 \| 0.086423562 \| 1.028321133 \| 0.969893942 \| 1.090268026 \| GCST90200474 \| \| Weighted mode \| 28 \| 0.038116815 \| 0.01646034 \| 0.028411327 \| 0.005854548 \| 0.070379082 \| 1.038852579 \| 1.005871719 \| 1.072914827 \| GCST90200474 \| \| MR Egger \| 21 \| -0.033736586 \| 0.025477188 \| 0.201150554 \| -0.083671875 \| 0.016198703 \| 0.966826147 \| 0.919732994 \| 1.016330613 \| GCST90200480 \| \| Weighted median \| 21 \| -0.036981508 \| 0.016095685 \| 0.021584347 \| -0.068529051 \| -0.005433965 \| 0.963693956 \| 0.933766333 \| 0.994580772 \| GCST90200480 \| \| Inverse variance weighted \| 21 \| -0.031492519 \| 0.012707957 \| 0.013205697 \| -0.056400114 \| -0.006584925 \| 0.968998205 \| 0.945160888 \| 0.993436709 \| GCST90200480 \| \| Simple mode \| 21 \| -0.004432755 \| 0.031158797 \| 0.888295381 \| -0.065503998 \| 0.056638488 \| 0.995577055 \| 0.936595302 \| 1.058273163 \| GCST90200480 \| \| Weighted mode \| 21 \| -0.034539752 \| 0.018865755 \| 0.082069595 \| -0.071516632 \| 0.002437127 \| 0.966049936 \| 0.930980794 \| 1.0024401 \| GCST90200480 \| \| MR Egger \| 12 \| 0.035725066 \| 0.040138297 \| 0.394347818 \| -0.042945997 \| 0.114396128 \| 1.036370874 \| 0.957963122 \| 1.121196174 \| GCST90200497 \| \| Weighted median \| 12 \| 0.049979632 \| 0.021732575 \| 0.021462017 \| 0.007383785 \| 0.09257548 \| 1.051249685 \| 1.007411112 \| 1.09699594 \| GCST90200497 \| \| Inverse variance weighted \| 12 \| 0.048401011 \| 0.015137357 \| 0.001386463 \| 0.018731791 \| 0.078070232 \| 1.049591469 \| 1.018908331 \| 1.08119859 \| GCST90200497 \| \| Simple mode \| 12 \| 0.021622577 \| 0.037485981 \| 0.575676574 \| -0.051849947 \| 0.0950951 \| 1.021858039 \| 0.949471327 \| 1.099763438 \| GCST90200497 \| \| Weighted mode \| 12 \| 0.014436483 \| 0.036016805 \| 0.696223657 \| -0.056156454 \| 0.08502942 \| 1.014541193 \| 0.945391214 \| 1.088749098 \| GCST90200497 \| \| MR Egger \| 14 \| 0.024655826 \| 0.032232594 \| 0.459090217 \| -0.038520057 \| 0.087831709 \| 1.024962294 \| 0.962212405 \| 1.091804366 \| GCST90200522 \| \| Weighted median \| 14 \| 0.039614696 \| 0.021209881 \| 0.061797321 \| -0.001956672 \| 0.081186063 \| 1.040409823 \| 0.998045241 \| 1.084572677 \| GCST90200522 \| \| Inverse variance weighted \| 14 \| 0.032812287 \| 0.015450455 \| 0.033694398 \| 0.002529395 \| 0.06309518 \| 1.033356547 \| 1.002532596 \| 1.065128213 \| GCST90200522 \| \| Simple mode \| 14 \| 0.021113108 \| 0.034739143 \| 0.55381509 \| -0.046975613 \| 0.089201828 \| 1.021337566 \| 0.954110665 \| 1.093301293 \| GCST90200522 \| \| Weighted mode \| 14 \| 0.031280272 \| 0.034853757 \| 0.385786715 \| -0.037033093 \| 0.099593636 \| 1.03177464 \| 0.963644245 \| 1.104721908 \| GCST90200522 \| \| MR Egger \| 25 \| -0.001112567 \| 0.016895596 \| 0.948066652 \| -0.034227935 \| 0.0320028 \| 0.998888051 \| 0.966351215 \| 1.032520397 \| GCST90200544 \| \| Weighted median \| 25 \| 0.018290527 \| 0.013718002 \| 0.182425834 \| -0.008596756 \| 0.045177811 \| 1.018458823 \| 0.99144009 \| 1.046213871 \| GCST90200544 \| \| Inverse variance weighted \| 25 \| 0.023014215 \| 0.010051837 \| 0.02204724 \| 0.003312614 \| 0.042715816 \| 1.023281085 \| 1.003318106 \| 1.043641267 \| GCST90200544 \| \| Simple mode \| 25 \| 0.011964505 \| 0.030034729 \| 0.693889795 \| -0.046903564 \| 0.070832574 \| 1.012036366 \| 0.954179411 \| 1.073401495 \| GCST90200544 \| \| Weighted mode \| 25 \| -0.003276479 \| 0.027086979 \| 0.904728633 \| -0.056366959 \| 0.049814001 \| 0.996728883 \| 0.945192226 \| 1.051075579 \| GCST90200544 \| \| MR Egger \| 27 \| 0.035494362 \| 0.021457402 \| 0.110594565 \| -0.006562146 \| 0.077550869 \| 1.036131806 \| 0.993459338 \| 1.080637202 \| GCST90200559 \| \| Weighted median \| 27 \| 0.029556984 \| 0.015126466 \| 0.050702231 \| -9.09E-05 \| 0.059204857 \| 1.029998127 \| 0.999909116 \| 1.06099257 \| GCST90200559 \| \| Inverse variance weighted \| 27 \| 0.035021188 \| 0.011802405 \| 0.003004349 \| 0.011888475 \| 0.058153902 \| 1.035641652 \| 1.011959424 \| 1.0598781 \| GCST90200559 \| \| Simple mode \| 27 \| 0.028587751 \| 0.03131799 \| 0.369723314 \| -0.032795509 \| 0.089971012 \| 1.029000303 \| 0.967736433 \| 1.094142566 \| GCST90200559 \| \| Weighted mode \| 27 \| 0.027421263 \| 0.029645671 \| 0.36348915 \| -0.030684252 \| 0.085526778 \| 1.027800686 \| 0.969781731 \| 1.08929073 \| GCST90200559 \| \| MR Egger \| 22 \| 0.013689872 \| 0.046322953 \| 0.770634603 \| -0.077103116 \| 0.104482859 \| 1.013784007 \| 0.925794385 \| 1.110136365 \| GCST90200562 \| \| Weighted median \| 22 \| -0.036789119 \| 0.01764227 \| 0.037043652 \| -0.071367968 \| -0.00221027 \| 0.963879378 \| 0.931119207 \| 0.997792171 \| GCST90200562 \| \| Inverse variance weighted \| 22 \| -0.026757389 \| 0.013433607 \| 0.046390309 \| -0.05308726 \| -0.000427519 \| 0.973597418 \| 0.948297261 \| 0.999572573 \| GCST90200562 \| \| Simple mode \| 22 \| -0.038705929 \| 0.03225313 \| 0.243477028 \| -0.101922065 \| 0.024510207 \| 0.962033574 \| 0.903099932 \| 1.024813051 \| GCST90200562 \| \| Weighted mode \| 22 \| -0.038705929 \| 0.025736855 \| 0.147495965 \| -0.089150166 \| 0.011738307 \| 0.962033574 \| 0.914708206 \| 1.011807472 \| GCST90200562 \| \| MR Egger \| 15 \| -0.003303803 \| 0.016019129 \| 0.839797465 \| -0.034701296 \| 0.028093689 \| 0.996701648 \| 0.96589389 \| 1.028492039 \| GCST90200570 \| \| Weighted median \| 15 \| 0.014200273 \| 0.01106448 \| 0.199348169 \| -0.007486107 \| 0.035886653 \| 1.014301576 \| 0.992541844 \| 1.036538352 \| GCST90200570 \| \| Inverse variance weighted \| 15 \| 0.021886729 \| 0.010209914 \| 0.032059012 \| 0.001875296 \| 0.041898161 \| 1.022128 \| 1.001877056 \| 1.042788276 \| GCST90200570 \| \| Simple mode \| 15 \| 0.025690762 \| 0.028162541 \| 0.37708796 \| -0.029507818 \| 0.080889341 \| 1.026023614 \| 0.970923287 \| 1.084250908 \| GCST90200570 \| \| Weighted mode \| 15 \| 0.014356774 \| 0.011042576 \| 0.214554574 \| -0.007286675 \| 0.036000222 \| 1.014460327 \| 0.992739808 \| 1.036656077 \| GCST90200570 \| \| MR Egger \| 16 \| 0.002587436 \| 0.033155759 \| 0.938901572 \| -0.062397851 \| 0.067572724 \| 1.002590787 \| 0.939509028 \| 1.069908065 \| GCST90200573 \| \| Weighted median \| 16 \| 0.015328116 \| 0.017084006 \| 0.369601403 \| -0.018156536 \| 0.048812769 \| 1.015446194 \| 0.982007301 \| 1.050023735 \| GCST90200573 \| \| Inverse variance weighted \| 16 \| 0.025250683 \| 0.011645935 \| 0.030143694 \| 0.00242465 \| 0.048076717 \| 1.025572182 \| 1.002427592 \| 1.049251148 \| GCST90200573 \| \| Simple mode \| 16 \| 0.004767619 \| 0.030652396 \| 0.878470823 \| -0.055311077 \| 0.064846316 \| 1.004779002 \| 0.946190764 \| 1.066995031 \| GCST90200573 \| \| Weighted mode \| 16 \| 0.007422925 \| 0.028059811 \| 0.794964688 \| -0.047574304 \| 0.062420155 \| 1.007450543 \| 0.953539618 \| 1.064409467 \| GCST90200573 \| \| MR Egger \| 15 \| -0.027339006 \| 0.01240918 \| 0.046231173 \| -0.051661 \| -0.003017012 \| 0.973031322 \| 0.949650744 \| 0.996987534 \| GCST90200575 \| \| Weighted median \| 15 \| -0.024515541 \| 0.009908746 \| 0.013356057 \| -0.043936684 \| -0.005094397 \| 0.975782525 \| 0.95701455 \| 0.994918557 \| GCST90200575 \| \| Inverse variance weighted \| 15 \| -0.020960869 \| 0.008614431 \| 0.014964901 \| -0.037845154 \| -0.004076584 \| 0.979257283 \| 0.962862024 \| 0.995931714 \| GCST90200575 \| \| Simple mode \| 15 \| -0.007681256 \| 0.025380783 \| 0.766613676 \| -0.057427591 \| 0.042065079 \| 0.992348169 \| 0.944190256 \| 1.042962352 \| GCST90200575 \| \| Weighted mode \| 15 \| -0.025444191 \| 0.009833132 \| 0.021489621 \| -0.044717131 \| -0.006171252 \| 0.974876784 \| 0.956267942 \| 0.993847751 \| GCST90200575 \| \| MR Egger \| 24 \| -0.006416926 \| 0.025075558 \| 0.800402821 \| -0.05556502 \| 0.042731168 \| 0.993603619 \| 0.945950516 \| 1.043657289 \| GCST90200628 \| \| Weighted median \| 24 \| -0.027906386 \| 0.01578124 \| 0.077006319 \| -0.058837615 \| 0.003024844 \| 0.972479401 \| 0.942859863 \| 1.003029423 \| GCST90200628 \| \| Inverse variance weighted \| 24 \| -0.03210078 \| 0.01082728 \| 0.003028742 \| -0.053322248 \| -0.010879312 \| 0.968408981 \| 0.948074448 \| 0.989179654 \| GCST90200628 \| \| Simple mode \| 24 \| -0.038303055 \| 0.026655498 \| 0.164192783 \| -0.090547831 \| 0.013941721 \| 0.96242123 \| 0.913430643 \| 1.01403936 \| GCST90200628 \| \| Weighted mode \| 24 \| -0.02615816 \| 0.016834981 \| 0.133885969 \| -0.059154724 \| 0.006838403 \| 0.974181001 \| 0.942560922 \| 1.006861839 \| GCST90200628 \| \| MR Egger \| 13 \| 0.028463672 \| 0.041688939 \| 0.50888032 \| -0.053246648 \| 0.110173993 \| 1.028872634 \| 0.948146126 \| 1.116472311 \| GCST90200632 \| \| Weighted median \| 13 \| 0.016162715 \| 0.021085173 \| 0.443352623 \| -0.025164225 \| 0.057489655 \| 1.016294039 \| 0.975149755 \| 1.059174314 \| GCST90200632 \| \| Inverse variance weighted \| 13 \| 0.0365066 \| 0.016526425 \| 0.027175788 \| 0.004114807 \| 0.068898392 \| 1.037181149 \| 1.004123285 \| 1.071327348 \| GCST90200632 \| \| Simple mode \| 13 \| 0.009528041 \| 0.036627512 \| 0.799170608 \| -0.062261883 \| 0.081317965 \| 1.009573577 \| 0.939636779 \| 1.084715743 \| GCST90200632 \| \| Weighted mode \| 13 \| 0.009974041 \| 0.035536293 \| 0.783742373 \| -0.059677093 \| 0.079625175 \| 1.010023948 \| 0.942068685 \| 1.082881101 \| GCST90200632 \| \| MR Egger \| 19 \| 0.059159824 \| 0.039379323 \| 0.151365452 \| -0.018023649 \| 0.136343297 \| 1.060944792 \| 0.982137806 \| 1.14607527 \| GCST90200643 \| \| Weighted median \| 19 \| 0.042376988 \| 0.022698752 \| 0.061911325 \| -0.002112567 \| 0.086866542 \| 1.043287711 \| 0.997889663 \| 1.090751101 \| GCST90200643 \| \| Inverse variance weighted \| 19 \| 0.047441481 \| 0.017287857 \| 0.006065704 \| 0.013557281 \| 0.08132568 \| 1.048584837 \| 1.013649597 \| 1.084724112 \| GCST90200643 \| \| Simple mode \| 19 \| 0.026256433 \| 0.046279443 \| 0.577487514 \| -0.064451276 \| 0.116964143 \| 1.02660417 \| 0.937581796 \| 1.124079122 \| GCST90200643 \| \| Weighted mode \| 19 \| 0.022365326 \| 0.045632694 \| 0.629972114 \| -0.067074754 \| 0.111805405 \| 1.022617304 \| 0.935125294 \| 1.118295225 \| GCST90200643 \| \| MR Egger \| 28 \| 0.032715716 \| 0.026423318 \| 0.226728604 \| -0.019073987 \| 0.08450542 \| 1.033256759 \| 0.98110677 \| 1.088178742 \| GCST90200644 \| \| Weighted median \| 28 \| -0.027149633 \| 0.017085936 \| 0.112059338 \| -0.060638068 \| 0.006338803 \| 0.973215606 \| 0.941163816 \| 1.006358935 \| GCST90200644 \| \| Inverse variance weighted \| 28 \| -0.02463456 \| 0.011919009 \| 0.038750227 \| -0.047995818 \| -0.001273301 \| 0.975666395 \| 0.953137773 \| 0.998727509 \| GCST90200644 \| \| Simple mode \| 28 \| -0.042801746 \| 0.033717172 \| 0.215119433 \| -0.108887402 \| 0.023283911 \| 0.958101319 \| 0.896831393 \| 1.023557097 \| GCST90200644 \| \| Weighted mode \| 28 \| -0.007458511 \| 0.032639133 \| 0.820968313 \| -0.071431211 \| 0.05651419 \| 0.992569235 \| 0.931060322 \| 1.058141629 \| GCST90200644 \| \| MR Egger \| 28 \| -0.022662211 \| 0.016312036 \| 0.176528125 \| -0.054633801 \| 0.00930938 \| 0.977592648 \| 0.946831813 \| 1.009352847 \| GCST90200645 \| \| Weighted median \| 28 \| -0.021410338 \| 0.011504786 \| 0.062745025 \| -0.043959719 \| 0.001139043 \| 0.978817236 \| 0.956992506 \| 1.001139692 \| GCST90200645 \| \| Inverse variance weighted \| 28 \| -0.022307847 \| 0.008178177 \| 0.006377203 \| -0.038337074 \| -0.006278621 \| 0.977939133 \| 0.962388491 \| 0.993741048 \| GCST90200645 \| \| Simple mode \| 28 \| -0.02071985 \| 0.022826168 \| 0.372055284 \| -0.065459139 \| 0.02401944 \| 0.979493332 \| 0.936637318 \| 1.02431023 \| GCST90200645 \| \| Weighted mode \| 28 \| -0.02071985 \| 0.021668172 \| 0.347438045 \| -0.063189466 \| 0.021749767 \| 0.979493332 \| 0.938765592 \| 1.021988017 \| GCST90200645 \| \| MR Egger \| 24 \| -0.001937849 \| 0.021659952 \| 0.929520338 \| -0.044391355 \| 0.040515656 \| 0.998064027 \| 0.956579522 \| 1.041347613 \| GCST90200651 \| \| Weighted median \| 24 \| 0.000996588 \| 0.015079772 \| 0.947307977 \| -0.028559766 \| 0.030552942 \| 1.000997085 \| 0.97184421 \| 1.031024473 \| GCST90200651 \| \| Inverse variance weighted \| 24 \| 0.02153336 \| 0.010480861 \| 0.039923348 \| 0.000990872 \| 0.042075847 \| 1.021766876 \| 1.000991363 \| 1.042973582 \| GCST90200651 \| \| Simple mode \| 24 \| 0.054838374 \| 0.032922187 \| 0.109337706 \| -0.009689113 \| 0.119365861 \| 1.056369864 \| 0.990357675 \| 1.126782089 \| GCST90200651 \| \| Weighted mode \| 24 \| -0.011795666 \| 0.023028865 \| 0.613386175 \| -0.056932241 \| 0.033340908 \| 0.98827363 \| 0.944658076 \| 1.033902945 \| GCST90200651 \| \| MR Egger \| 20 \| 0.046705116 \| 0.027330188 \| 0.104650812 \| -0.006862054 \| 0.100272285 \| 1.04781298 \| 0.993161437 \| 1.10547188 \| GCST90200661 \| \| Weighted median \| 20 \| 0.039691157 \| 0.016453349 \| 0.015850271 \| 0.007442592 \| 0.071939722 \| 1.040489377 \| 1.007470357 \| 1.074590568 \| GCST90200661 \| \| Inverse variance weighted \| 20 \| 0.039717746 \| 0.012300119 \| 0.001242005 \| 0.015609513 \| 0.06382598 \| 1.040517043 \| 1.015731978 \| 1.065906894 \| GCST90200661 \| \| Simple mode \| 20 \| 0.038330207 \| 0.02829537 \| 0.191421047 \| -0.017128719 \| 0.093789133 \| 1.039074286 \| 0.983017144 \| 1.098328121 \| GCST90200661 \| \| Weighted mode \| 20 \| 0.039230694 \| 0.016602982 \| 0.028951199 \| 0.00668885 \| 0.071772538 \| 1.04001038 \| 1.00671127 \| 1.074410929 \| GCST90200661 \| \| MR Egger \| 32 \| 0.02176031 \| 0.01112498 \| 0.059834713 \| -4.47E-05 \| 0.043565271 \| 1.021998792 \| 0.999955349 \| 1.04452817 \| GCST90200685 \| \| Weighted median \| 32 \| 0.022165331 \| 0.007048232 \| 0.001661963 \| 0.008350796 \| 0.035979866 \| 1.022412807 \| 1.008385761 \| 1.036634974 \| GCST90200685 \| \| Inverse variance weighted \| 32 \| 0.015401454 \| 0.007762289 \| 0.047240449 \| 0.000187367 \| 0.030615541 \| 1.015520668 \| 1.000187384 \| 1.031089016 \| GCST90200685 \| \| Simple mode \| 32 \| -0.000504485 \| 0.029590141 \| 0.986506699 \| -0.058501163 \| 0.057492192 \| 0.999495642 \| 0.943177144 \| 1.059177 \| GCST90200685 \| \| Weighted mode \| 32 \| 0.021428794 \| 0.00751481 \| 0.007675199 \| 0.006699767 \| 0.036157821 \| 1.02166004 \| 1.006722261 \| 1.036819466 \| GCST90200685 \| \| MR Egger \| 28 \| 0.021290794 \| 0.013070941 \| 0.115399919 \| -0.004328249 \| 0.046909838 \| 1.02151906 \| 0.995681104 \| 1.048027512 \| GCST90200692 \| \| Weighted median \| 28 \| 0.024971339 \| 0.007761923 \| 0.001294669 \| 0.00975797 \| 0.040184708 \| 1.025285735 \| 1.009805734 \| 1.041003038 \| GCST90200692 \| \| Inverse variance weighted \| 28 \| 0.02073416 \| 0.008635226 \| 0.016345259 \| 0.003809116 \| 0.037659204 \| 1.020950606 \| 1.00381638 \| 1.038377298 \| GCST90200692 \| \| Simple mode \| 28 \| 0.018326582 \| 0.028406185 \| 0.524267282 \| -0.037349541 \| 0.074002706 \| 1.018495545 \| 0.96333935 \| 1.076809719 \| GCST90200692 \| \| Weighted mode \| 28 \| 0.024587227 \| 0.007944134 \| 0.004545973 \| 0.009016724 \| 0.04015773 \| 1.024891986 \| 1.009057497 \| 1.040974954 \| GCST90200692 \| \| MR Egger \| 36 \| -0.027085832 \| 0.00919779 \| 0.005793043 \| -0.0451135 \| -0.009058164 \| 0.9732777 \| 0.955888983 \| 0.990982738 \| GCST90200705 \| \| Weighted median \| 36 \| -0.017065871 \| 0.008165839 \| 0.036625862 \| -0.033070916 \| -0.001060826 \| 0.983078926 \| 0.967469948 \| 0.998939736 \| GCST90200705 \| \| Inverse variance weighted \| 36 \| -0.01356649 \| 0.006782745 \| 0.045484361 \| -0.02686067 \| -0.000272309 \| 0.98652512 \| 0.973496869 \| 0.999727728 \| GCST90200705 \| \| Simple mode \| 36 \| -0.004857577 \| 0.019413916 \| 0.80388701 \| -0.042908853 \| 0.033193698 \| 0.995154202 \| 0.957998705 \| 1.033750756 \| GCST90200705 \| \| Weighted mode \| 36 \| -0.018615888 \| 0.008378086 \| 0.032845771 \| -0.035036937 \| -0.002194838 \| 0.981556318 \| 0.96556975 \| 0.997807568 \| GCST90200705 \| \| MR Egger \| 23 \| -0.019390492 \| 0.023091771 \| 0.410526858 \| -0.064650364 \| 0.025869379 \| 0.980796294 \| 0.937395154 \| 1.026206896 \| GCST90200728 \| \| Weighted median \| 23 \| -0.019703174 \| 0.012345799 \| 0.110501846 \| -0.043900941 \| 0.004494593 \| 0.980489665 \| 0.957048757 \| 1.004504709 \| GCST90200728 \| \| Inverse variance weighted \| 23 \| -0.022409792 \| 0.009179624 \| 0.014636364 \| -0.040401855 \| -0.004417729 \| 0.977839442 \| 0.960403418 \| 0.995592015 \| GCST90200728 \| \| Simple mode \| 23 \| -0.013181595 \| 0.023232417 \| 0.576201013 \| -0.058717133 \| 0.032353943 \| 0.986904902 \| 0.942973468 \| 1.032883022 \| GCST90200728 \| \| Weighted mode \| 23 \| -0.01439639 \| 0.024337577 \| 0.560195634 \| -0.06209804 \| 0.033305261 \| 0.985706743 \| 0.939790745 \| 1.03386609 \| GCST90200728 \| \| MR Egger \| 18 \| 0.032083033 \| 0.016607702 \| 0.071289949 \| -0.000468063 \| 0.064634129 \| 1.032603242 \| 0.999532047 \| 1.066768653 \| GCST90200740 \| \| Weighted median \| 18 \| 0.035106696 \| 0.010110111 \| 0.000515761 \| 0.015290878 \| 0.054922515 \| 1.035730212 \| 1.015408382 \| 1.056458752 \| GCST90200740 \| \| Inverse variance weighted \| 18 \| 0.028944602 \| 0.010175135 \| 0.004446157 \| 0.009001337 \| 0.048887866 \| 1.029367568 \| 1.009041971 \| 1.050102592 \| GCST90200740 \| \| Simple mode \| 18 \| 0.003913834 \| 0.029717027 \| 0.89676506 \| -0.05433154 \| 0.062159207 \| 1.003921503 \| 0.947118047 \| 1.064131749 \| GCST90200740 \| \| Weighted mode \| 18 \| 0.032369931 \| 0.010154552 \| 0.005387955 \| 0.012467009 \| 0.052272853 \| 1.032899536 \| 1.012545046 \| 1.053663198 \| GCST90200740 \| \| MR Egger \| 16 \| 0.06751318 \| 0.035228198 \| 0.075950983 \| -0.001534088 \| 0.136560448 \| 1.06984436 \| 0.998467089 \| 1.146324169 \| GCST90200749 \| \| Weighted median \| 16 \| 0.05746694 \| 0.017263947 \| 0.000872443 \| 0.023629605 \| 0.091304275 \| 1.059150255 \| 1.023910996 \| 1.095602319 \| GCST90200749 \| \| Inverse variance weighted \| 16 \| 0.034706476 \| 0.016173401 \| 0.03188108 \| 0.00300661 \| 0.066406342 \| 1.035315774 \| 1.003011135 \| 1.068660871 \| GCST90200749 \| \| Simple mode \| 16 \| -0.032047292 \| 0.046315805 \| 0.499552533 \| -0.12282627 \| 0.058731685 \| 0.96846078 \| 0.884417299 \| 1.060490657 \| GCST90200749 \| \| Weighted mode \| 16 \| 0.058804317 \| 0.017592221 \| 0.004452127 \| 0.024323563 \| 0.09328507 \| 1.060567685 \| 1.024621794 \| 1.097774634 \| GCST90200749 \| \| MR Egger \| 21 \| -0.027519436 \| 0.013308183 \| 0.052545572 \| -0.053603474 \| -0.001435398 \| 0.972855774 \| 0.947807862 \| 0.998565632 \| GCST90200794 \| \| Weighted median \| 21 \| -0.026569931 \| 0.009155619 \| 0.003707464 \| -0.044514944 \| -0.008624919 \| 0.973779944 \| 0.956461307 \| 0.991412169 \| GCST90200794 \| \| Inverse variance weighted \| 21 \| -0.018399102 \| 0.008366643 \| 0.027870658 \| -0.034797721 \| -0.002000482 \| 0.981769128 \| 0.965800757 \| 0.998001517 \| GCST90200794 \| \| Simple mode \| 21 \| 0.003836912 \| 0.032418344 \| 0.9069661 \| -0.059703043 \| 0.067376866 \| 1.003844282 \| 0.942044239 \| 1.069698536 \| GCST90200794 \| \| Weighted mode \| 21 \| -0.026956016 \| 0.009332357 \| 0.009085211 \| -0.045247435 \| -0.008664597 \| 0.973404055 \| 0.955760964 \| 0.991372832 \| GCST90200794 \| \| MR Egger \| 20 \| -0.04433247 \| 0.012950401 \| 0.003031276 \| -0.069715257 \| -0.018949684 \| 0.956635851 \| 0.93265935 \| 0.981228733 \| GCST90200795 \| \| Weighted median \| 20 \| -0.032501252 \| 0.00953209 \| 0.000650422 \| -0.051184148 \| -0.013818356 \| 0.968021238 \| 0.950103695 \| 0.986276679 \| GCST90200795 \| \| Inverse variance weighted \| 20 \| -0.027312098 \| 0.008651064 \| 0.001593578 \| -0.044268183 \| -0.010356012 \| 0.973057505 \| 0.956697353 \| 0.989697426 \| GCST90200795 \| \| Simple mode \| 20 \| -0.005170329 \| 0.026077792 \| 0.844943256 \| -0.056282801 \| 0.045942143 \| 0.994843015 \| 0.945271775 \| 1.047013833 \| GCST90200795 \| \| Weighted mode \| 20 \| -0.032516925 \| 0.009605703 \| 0.003106937 \| -0.051344104 \| -0.013689746 \| 0.968006066 \| 0.949951733 \| 0.986403532 \| GCST90200795 \| \| MR Egger \| 14 \| -0.000631512 \| 0.03286866 \| 0.984986798 \| -0.065054085 \| 0.063791061 \| 0.999368687 \| 0.937016783 \| 1.065869674 \| GCST90200804 \| \| Weighted median \| 14 \| 0.015987147 \| 0.01942927 \| 0.410599964 \| -0.022094222 \| 0.054068515 \| 1.016115625 \| 0.978148068 \| 1.055556921 \| GCST90200804 \| \| Inverse variance weighted \| 14 \| 0.031025369 \| 0.014365966 \| 0.030800249 \| 0.002868077 \| 0.059182662 \| 1.031511672 \| 1.002872194 \| 1.060969022 \| GCST90200804 \| \| Simple mode \| 14 \| 0.012808472 \| 0.033252862 \| 0.706329547 \| -0.052367137 \| 0.077984081 \| 1.012890852 \| 0.948980397 \| 1.081105449 \| GCST90200804 \| \| Weighted mode \| 14 \| 0.01370812 \| 0.031862832 \| 0.674079694 \| -0.04874303 \| 0.07615927 \| 1.013802507 \| 0.952425843 \| 1.079134434 \| GCST90200804 \| \| MR Egger \| 22 \| 0.004329462 \| 0.031511075 \| 0.89209242 \| -0.057432245 \| 0.066091169 \| 1.004338848 \| 0.944185862 \| 1.06832411 \| GCST90200812 \| \| Weighted median \| 22 \| -0.02695964 \| 0.018189635 \| 0.13830225 \| -0.062611324 \| 0.008692044 \| 0.973400527 \| 0.939308489 \| 1.00872993 \| GCST90200812 \| \| Inverse variance weighted \| 22 \| -0.030961818 \| 0.013239 \| 0.01935187 \| -0.056910258 \| -0.005013378 \| 0.96951259 \| 0.944678843 \| 0.994999168 \| GCST90200812 \| \| Simple mode \| 22 \| -0.027830409 \| 0.039583274 \| 0.489724688 \| -0.105413626 \| 0.049752808 \| 0.972553289 \| 0.899952202 \| 1.051011262 \| GCST90200812 \| \| Weighted mode \| 22 \| -0.030225267 \| 0.033247712 \| 0.373616935 \| -0.095390784 \| 0.034940249 \| 0.970226949 \| 0.909017636 \| 1.035557831 \| GCST90200812 \| \| MR Egger \| 16 \| 0.065420245 \| 0.03134595 \| 0.055650431 \| 0.003982183 \| 0.126858307 \| 1.067607586 \| 1.003990122 \| 1.135256148 \| GCST90200848 \| \| Weighted median \| 16 \| 0.035610192 \| 0.02073023 \| 0.085834835 \| -0.005021059 \| 0.076241442 \| 1.036251828 \| 0.994991526 \| 1.079223112 \| GCST90200848 \| \| Inverse variance weighted \| 16 \| 0.031513857 \| 0.015445055 \| 0.041312007 \| 0.001241549 \| 0.061786164 \| 1.032015676 \| 1.00124232 \| 1.063734856 \| GCST90200848 \| \| Simple mode \| 16 \| 0.06284559 \| 0.035853216 \| 0.100034021 \| -0.007426714 \| 0.133117894 \| 1.064862401 \| 0.992600796 \| 1.14238467 \| GCST90200848 \| \| Weighted mode \| 16 \| 0.058943434 \| 0.034407833 \| 0.107283629 \| -0.008495919 \| 0.126382787 \| 1.060715239 \| 0.991540069 \| 1.13471644 \| GCST90200848 \| \| MR Egger \| 20 \| 0.059610382 \| 0.032698876 \| 0.084955343 \| -0.004479416 \| 0.12370018 \| 1.061422917 \| 0.995530602 \| 1.131676521 \| GCST90200859 \| \| Weighted median \| 20 \| 0.035890554 \| 0.018163368 \| 0.048156402 \| 0.000290353 \| 0.071490754 \| 1.036542395 \| 1.000290395 \| 1.07410822 \| GCST90200859 \| \| Inverse variance weighted \| 20 \| 0.038637751 \| 0.013545487 \| 0.004338437 \| 0.012088597 \| 0.065186906 \| 1.039393896 \| 1.01216196 \| 1.067358501 \| GCST90200859 \| \| Simple mode \| 20 \| 0.031392302 \| 0.034123055 \| 0.369119993 \| -0.035488886 \| 0.09827349 \| 1.031890237 \| 0.96513346 \| 1.103264476 \| GCST90200859 \| \| Weighted mode \| 20 \| 0.032512692 \| 0.032112779 \| 0.324041803 \| -0.030428355 \| 0.095453739 \| 1.033047005 \| 0.970029928 \| 1.100157926 \| GCST90200859 \| \| MR Egger \| 15 \| 0.03576877 \| 0.036898044 \| 0.350039116 \| -0.036551397 \| 0.108088936 \| 1.036416168 \| 0.964108541 \| 1.114146829 \| GCST90200860 \| \| Weighted median \| 15 \| 0.033851691 \| 0.021275158 \| 0.111578758 \| -0.00784762 \| 0.075551001 \| 1.03443118 \| 0.992183092 \| 1.07847823 \| GCST90200860 \| \| Inverse variance weighted \| 15 \| 0.030016937 \| 0.015010246 \| 0.045525849 \| 0.000596854 \| 0.059437019 \| 1.030471987 \| 1.000597032 \| 1.061238921 \| GCST90200860 \| \| Simple mode \| 15 \| 0.060167648 \| 0.036675777 \| 0.12316199 \| -0.011716875 \| 0.132052172 \| 1.062014577 \| 0.9883515 \| 1.141167854 \| GCST90200860 \| \| Weighted mode \| 15 \| 0.056798046 \| 0.036451674 \| 0.141505179 \| -0.014647234 \| 0.128243327 \| 1.058442032 \| 0.985459515 \| 1.13682959 \| GCST90200860 \| \| MR Egger \| 21 \| 0.04455389 \| 0.030470746 \| 0.160034642 \| -0.015168772 \| 0.104276553 \| 1.045561321 \| 0.984945694 \| 1.10990736 \| GCST90200863 \| \| Weighted median \| 21 \| 0.034581731 \| 0.016248445 \| 0.033311372 \| 0.002734779 \| 0.066428682 \| 1.035186632 \| 1.002738522 \| 1.068684745 \| GCST90200863 \| \| Inverse variance weighted \| 21 \| 0.023469047 \| 0.011507004 \| 0.041395717 \| 0.000915319 \| 0.046022775 \| 1.023746612 \| 1.000915738 \| 1.047098259 \| GCST90200863 \| \| Simple mode \| 21 \| 0.031912705 \| 0.030661575 \| 0.310385538 \| -0.028183983 \| 0.092009393 \| 1.032427376 \| 0.972209481 \| 1.09637512 \| GCST90200863 \| \| Weighted mode \| 21 \| 0.031255551 \| 0.021751899 \| 0.166203761 \| -0.011378171 \| 0.073889273 \| 1.031749135 \| 0.988686316 \| 1.076687581 \| GCST90200863 \| \| MR Egger \| 14 \| -0.034258099 \| 0.036284288 \| 0.363711374 \| -0.105375304 \| 0.036859106 \| 0.966322066 \| 0.899986691 \| 1.037546827 \| GCST90200864 \| \| Weighted median \| 14 \| -0.038545762 \| 0.023884027 \| 0.106555203 \| -0.085358455 \| 0.008266932 \| 0.962187673 \| 0.918183098 \| 1.008301197 \| GCST90200864 \| \| Inverse variance weighted \| 14 \| -0.052291812 \| 0.016608344 \| 0.001640958 \| -0.084844167 \| -0.019739457 \| 0.949051881 \| 0.91865543 \| 0.98045409 \| GCST90200864 \| \| Simple mode \| 14 \| -0.020522336 \| 0.042227023 \| 0.635056492 \| -0.103287301 \| 0.06224263 \| 0.979686814 \| 0.901867829 \| 1.064220525 \| GCST90200864 \| \| Weighted mode \| 14 \| -0.020522336 \| 0.039164753 \| 0.609096691 \| -0.097285251 \| 0.05624058 \| 0.979686814 \| 0.907297162 \| 1.057852151 \| GCST90200864 \| \| MR Egger \| 19 \| 0.083052424 \| 0.037660319 \| 0.041489368 \| 0.009238198 \| 0.15686665 \| 1.086598771 \| 1.009281002 \| 1.169839606 \| GCST90200867 \| \| Weighted median \| 19 \| 0.022083607 \| 0.019778474 \| 0.264187808 \| -0.016682201 \| 0.060849416 \| 1.022329255 \| 0.983456176 \| 1.062738871 \| GCST90200867 \| \| Inverse variance weighted \| 19 \| 0.028873679 \| 0.013961628 \| 0.038633064 \| 0.001508889 \| 0.056238469 \| 1.029294565 \| 1.001510028 \| 1.057849919 \| GCST90200867 \| \| Simple mode \| 19 \| 0.013878802 \| 0.03567316 \| 0.701799956 \| -0.056040591 \| 0.083798195 \| 1.013975559 \| 0.945500756 \| 1.087409427 \| GCST90200867 \| \| Weighted mode \| 19 \| 0.013152074 \| 0.034803521 \| 0.709927995 \| -0.055062826 \| 0.081366974 \| 1.013238943 \| 0.946425686 \| 1.084768906 \| GCST90200867 \| \| MR Egger \| 27 \| -0.044280878 \| 0.027317463 \| 0.117569708 \| -0.097823104 \| 0.009261349 \| 0.956685208 \| 0.9068093 \| 1.009304368 \| GCST90200885 \| \| Weighted median \| 27 \| -0.036545976 \| 0.015748253 \| 0.020306451 \| -0.067412552 \| -0.0056794 \| 0.964113767 \| 0.934809464 \| 0.994336697 \| GCST90200885 \| \| Inverse variance weighted \| 27 \| -0.02757811 \| 0.011575411 \| 0.017196771 \| -0.050265915 \| -0.004890304 \| 0.972798695 \| 0.950976512 \| 0.995121634 \| GCST90200885 \| \| Simple mode \| 27 \| -0.045164305 \| 0.030142569 \| 0.146084882 \| -0.104243741 \| 0.013915131 \| 0.95584042 \| 0.901005659 \| 1.014012397 \| GCST90200885 \| \| Weighted mode \| 27 \| -0.045843656 \| 0.029303181 \| 0.129800854 \| -0.103277891 \| 0.011590579 \| 0.955191289 \| 0.901876316 \| 1.01165801 \| GCST90200885 \| \| MR Egger \| 20 \| -0.024391548 \| 0.026180457 \| 0.363835843 \| -0.075705243 \| 0.026922147 \| 0.975903522 \| 0.927089432 \| 1.027287822 \| GCST90200890 \| \| Weighted median \| 20 \| -0.032630283 \| 0.017999364 \| 0.069853792 \| -0.067909037 \| 0.00264847 \| 0.967896341 \| 0.93434546 \| 1.00265198 \| GCST90200890 \| \| Inverse variance weighted \| 20 \| -0.027362665 \| 0.013342282 \| 0.040284134 \| -0.053513537 \| -0.001211793 \| 0.973008301 \| 0.947893109 \| 0.998788941 \| GCST90200890 \| \| Simple mode \| 20 \| -0.037944717 \| 0.031812213 \| 0.247641975 \| -0.100296654 \| 0.02440722 \| 0.962766164 \| 0.904569034 \| 1.024707515 \| GCST90200890 \| \| Weighted mode \| 20 \| -0.035704689 \| 0.032053827 \| 0.279216373 \| -0.09853019 \| 0.027120812 \| 0.964925204 \| 0.906168335 \| 1.027491928 \| GCST90200890 \| \| MR Egger \| 17 \| -0.014270785 \| 0.037848357 \| 0.711416096 \| -0.088453563 \| 0.059911994 \| 0.98583056 \| 0.915345615 \| 1.061743103 \| GCST90200913 \| \| Weighted median \| 17 \| 0.036504145 \| 0.021247924 \| 0.08579479 \| -0.005141785 \| 0.078150076 \| 1.037178604 \| 0.994871411 \| 1.081284921 \| GCST90200913 \| \| Inverse variance weighted \| 17 \| 0.039489156 \| 0.017946492 \| 0.02777973 \| 0.004314032 \| 0.07466428 \| 1.040279218 \| 1.004323351 \| 1.077522344 \| GCST90200913 \| \| Simple mode \| 17 \| 0.032931989 \| 0.041941969 \| 0.443820007 \| -0.049274271 \| 0.115138249 \| 1.033480249 \| 0.95192001 \| 1.122028547 \| GCST90200913 \| \| Weighted mode \| 17 \| 0.031998837 \| 0.036999368 \| 0.39990347 \| -0.040519925 \| 0.104517599 \| 1.032516304 \| 0.96029003 \| 1.110174931 \| GCST90200913 \| \| MR Egger \| 21 \| -0.032129629 \| 0.030988649 \| 0.312836324 \| -0.092867381 \| 0.028608123 \| 0.968381044 \| 0.91131435 \| 1.029021266 \| GCST90200933 \| \| Weighted median \| 21 \| -0.012490931 \| 0.018096901 \| 0.490052789 \| -0.047960858 \| 0.022978995 \| 0.987586757 \| 0.953171096 \| 1.023245046 \| GCST90200933 \| \| Inverse variance weighted \| 21 \| -0.025210298 \| 0.01284764 \| 0.049733234 \| -0.050391673 \| -2.89E-05 \| 0.975104828 \| 0.950856927 \| 0.999971077 \| GCST90200933 \| \| Simple mode \| 21 \| -0.006687512 \| 0.035528643 \| 0.852594076 \| -0.076323653 \| 0.062948629 \| 0.9933348 \| 0.926516288 \| 1.064972129 \| GCST90200933 \| \| Weighted mode \| 21 \| -0.006687512 \| 0.03082744 \| 0.830457587 \| -0.067109295 \| 0.053734271 \| 0.9933348 \| 0.935092995 \| 1.055204166 \| GCST90200933 \| \| MR Egger \| 22 \| 0.012165636 \| 0.023869208 \| 0.615854491 \| -0.034618011 \| 0.058949283 \| 1.012239938 \| 0.965974337 \| 1.060721443 \| GCST90200935 \| \| Weighted median \| 22 \| -0.002566127 \| 0.0170646 \| 0.880466996 \| -0.036012744 \| 0.030880489 \| 0.997437162 \| 0.964628 \| 1.031362237 \| GCST90200935 \| \| Inverse variance weighted \| 22 \| -0.027312947 \| 0.011899949 \| 0.021720778 \| -0.050636847 \| -0.003989047 \| 0.973056678 \| 0.950623829 \| 0.996018898 \| GCST90200935 \| \| Simple mode \| 22 \| -0.003265082 \| 0.030728527 \| 0.91638795 \| -0.063492995 \| 0.056962831 \| 0.996740243 \| 0.938480693 \| 1.058616462 \| GCST90200935 \| \| Weighted mode \| 22 \| -0.000199722 \| 0.023111674 \| 0.993186645 \| -0.045498602 \| 0.045099158 \| 0.999800298 \| 0.955520938 \| 1.046131587 \| GCST90200935 \| \| MR Egger \| 24 \| 0.019935504 \| 0.014887732 \| 0.194224734 \| -0.00924445 \| 0.049115458 \| 1.020135544 \| 0.990798149 \| 1.050341615 \| GCST90200968 \| \| Weighted median \| 24 \| 0.028788111 \| 0.01147426 \| 0.012109761 \| 0.006298561 \| 0.051277661 \| 1.029206494 \| 1.006318439 \| 1.052615123 \| GCST90200968 \| \| Inverse variance weighted \| 24 \| 0.025706328 \| 0.009962212 \| 0.009869064 \| 0.006180392 \| 0.045232264 \| 1.026039585 \| 1.00619953 \| 1.046270843 \| GCST90200968 \| \| Simple mode \| 24 \| 0.030981537 \| 0.029160751 \| 0.299064888 \| -0.026173536 \| 0.08813661 \| 1.03146646 \| 0.974166022 \| 1.092137309 \| GCST90200968 \| \| Weighted mode \| 24 \| 0.029173298 \| 0.0122339 \| 0.025728161 \| 0.005194854 \| 0.053151743 \| 1.029603008 \| 1.005208371 \| 1.054589659 \| GCST90200968 \| \| MR Egger \| 20 \| 0.035639501 \| 0.0161598 \| 0.040664998 \| 0.003966293 \| 0.067312709 \| 1.036282201 \| 1.003974169 \| 1.069629909 \| GCST90200979 \| \| Weighted median \| 20 \| 0.037277678 \| 0.010286301 \| 0.000290068 \| 0.017116528 \| 0.057438828 \| 1.037981205 \| 1.017263855 \| 1.05912048 \| GCST90200979 \| \| Inverse variance weighted \| 20 \| 0.031746139 \| 0.009869316 \| 0.001296965 \| 0.01240228 \| 0.051089998 \| 1.032255422 \| 1.012479507 \| 1.052417604 \| GCST90200979 \| \| Simple mode \| 20 \| 0.031869544 \| 0.028349512 \| 0.274945579 \| -0.023695499 \| 0.087434587 \| 1.032382816 \| 0.976583035 \| 1.091370873 \| GCST90200979 \| \| Weighted mode \| 20 \| 0.036906853 \| 0.010599356 \| 0.002495246 \| 0.016132115 \| 0.057681592 \| 1.037596368 \| 1.01626294 \| 1.059377628 \| GCST90200979 \| \| MR Egger \| 19 \| -0.04518939 \| 0.023069874 \| 0.066748445 \| -0.090406343 \| 2.76E-05 \| 0.955816442 \| 0.913559891 \| 1.000027563 \| GCST90200990 \| \| Weighted median \| 19 \| -0.04798918 \| 0.014150988 \| 0.000695811 \| -0.075725118 \| -0.020253243 \| 0.9531441 \| 0.927071007 \| 0.979950476 \| GCST90200990 \| \| Inverse variance weighted \| 19 \| -0.028251036 \| 0.011141566 \| 0.011224107 \| -0.050088506 \| -0.006413566 \| 0.972144293 \| 0.951145239 \| 0.993606957 \| GCST90200990 \| \| Simple mode \| 19 \| -0.042961762 \| 0.029483318 \| 0.162298575 \| -0.100749066 \| 0.014825541 \| 0.957948019 \| 0.904159889 \| 1.014935984 \| GCST90200990 \| \| Weighted mode \| 19 \| -0.048908095 \| 0.01551212 \| 0.005503275 \| -0.079311851 \| -0.018504339 \| 0.952268644 \| 0.923751807 \| 0.981665815 \| GCST90200990 \| \| MR Egger \| 20 \| 0.126567544 \| 0.033918151 \| 0.001527428 \| 0.060087968 \| 0.193047121 \| 1.134926106 \| 1.061929958 \| 1.212939947 \| GCST90201007 \| \| Weighted median \| 20 \| 0.021290258 \| 0.019204333 \| 0.267595279 \| -0.016350236 \| 0.058930751 \| 1.021518512 \| 0.983782704 \| 1.060701786 \| GCST90201007 \| \| Inverse variance weighted \| 20 \| 0.034787072 \| 0.014606627 \| 0.017237839 \| 0.006158083 \| 0.063416062 \| 1.03539922 \| 1.006177083 \| 1.065470049 \| GCST90201007 \| \| Simple mode \| 20 \| 0.028860961 \| 0.040307042 \| 0.482682542 \| -0.050140841 \| 0.107862763 \| 1.029281474 \| 0.951095462 \| 1.113894867 \| GCST90201007 \| \| Weighted mode \| 20 \| 0.008791418 \| 0.032657446 \| 0.790676658 \| -0.055217176 \| 0.072800012 \| 1.008830176 \| 0.946279617 \| 1.075515425 \| GCST90201007 \| \| MR Egger \| 24 \| 0.020908844 \| 0.027734647 \| 0.458909362 \| -0.033451064 \| 0.075268753 \| 1.021128966 \| 0.967102236 \| 1.078173874 \| GCST90201009 \| \| Weighted median \| 24 \| 0.029090408 \| 0.017219607 \| 0.091147215 \| -0.004660023 \| 0.062840838 \| 1.029517666 \| 0.995350818 \| 1.064857341 \| GCST90201009 \| \| Inverse variance weighted \| 24 \| 0.032811004 \| 0.011764054 \| 0.005285637 \| 0.009753459 \| 0.05586855 \| 1.033355221 \| 1.009801179 \| 1.057458671 \| GCST90201009 \| \| Simple mode \| 24 \| 0.027437438 \| 0.03576822 \| 0.450831122 \| -0.042668273 \| 0.09754315 \| 1.027817311 \| 0.958229208 \| 1.102459011 \| GCST90201009 \| \| Weighted mode \| 24 \| 0.025193421 \| 0.028631855 \| 0.388010254 \| -0.030925015 \| 0.081311857 \| 1.025513457 \| 0.969548272 \| 1.084709118 \| GCST90201009 \| \| MR Egger \| 26 \| 0.037738256 \| 0.017857412 \| 0.045161058 \| 0.002737729 \| 0.072738784 \| 1.038459387 \| 1.00274148 \| 1.075449576 \| GCST90201013 \| \| Weighted median \| 26 \| 0.037193001 \| 0.01213155 \| 0.002170828 \| 0.013415163 \| 0.060970839 \| 1.037893316 \| 1.01350555 \| 1.062867919 \| GCST90201013 \| \| Inverse variance weighted \| 26 \| 0.026187094 \| 0.01194234 \| 0.028322216 \| 0.002780107 \| 0.049594081 \| 1.026532989 \| 1.002783975 \| 1.050844452 \| GCST90201013 \| \| Simple mode \| 26 \| 0.035875234 \| 0.025780812 \| 0.176318626 \| -0.014655157 \| 0.086405624 \| 1.036526515 \| 0.985451707 \| 1.09024847 \| GCST90201013 \| \| Weighted mode \| 26 \| 0.038309581 \| 0.012495571 \| 0.005151704 \| 0.013818262 \| 0.062800901 \| 1.039052854 \| 1.013914175 \| 1.064814814 \| GCST90201013 \| |
| --- | --- | --- | --- | --- | --- | --- | --- | --- | --- | --- | --- | --- | --- | --- | --- | --- | --- | --- | --- | --- | --- | --- | --- | --- | --- | --- | --- | --- | --- | --- | --- | --- | --- | --- | --- | --- | --- | --- | --- | --- | --- | --- | --- | --- | --- | --- | --- | --- | --- | --- | --- | --- | --- | --- | --- | --- | --- | --- | --- | --- | --- | --- | --- | --- | --- | --- | --- | --- | --- | --- | --- | --- | --- | --- | --- | --- | --- | --- | --- | --- | --- | --- | --- | --- | --- | --- | --- | --- | --- | --- | --- | --- | --- | --- | --- | --- | --- | --- | --- | --- | --- | --- | --- | --- | --- | --- | --- | --- | --- | --- | --- | --- | --- | --- | --- | --- | --- | --- | --- | --- | --- | --- | --- | --- | --- | --- | --- | --- | --- | --- | --- | --- | --- | --- | --- | --- | --- | --- | --- | --- | --- | --- | --- | --- | --- | --- | --- | --- | --- | --- | --- | --- | --- | --- | --- | --- | --- | --- | --- | --- | --- | --- | --- | --- | --- | --- | --- | --- | --- | --- | --- | --- | --- | --- | --- | --- | --- | --- | --- | --- | --- | --- | --- | --- | --- | --- | --- | --- | --- | --- | --- | --- | --- | --- | --- | --- | --- | --- | --- | --- | --- | --- | --- | --- | --- | --- | --- | --- | --- | --- | --- | --- | --- | --- | --- | --- | --- | --- | --- | --- | --- | --- | --- | --- | --- | --- | --- | --- | --- | --- | --- | --- | --- | --- | --- | --- | --- | --- | --- | --- | --- | --- | --- | --- | --- | --- | --- | --- | --- | --- | --- | --- | --- | --- | --- | --- | --- | --- | --- | --- | --- | --- | --- | --- | --- | --- | --- | --- | --- | --- | --- | --- | --- | --- | --- | --- | --- | --- | --- | --- | --- | --- | --- | --- | --- | --- | --- | --- | --- | --- | --- | --- | --- | --- | --- | --- | --- | --- | --- | --- | --- | --- | --- | --- | --- | --- | --- | --- | --- | --- | --- | --- | --- | --- | --- | --- | --- | --- | --- | --- | --- | --- | --- | --- | --- | --- | --- | --- | --- | --- | --- | --- | --- | --- | --- | --- | --- | --- | --- | --- | --- | --- | --- | --- | --- | --- | --- | --- | --- | --- | --- | --- | --- | --- | --- | --- | --- | --- | --- | --- | --- | --- | --- | --- | --- | --- | --- | --- | --- | --- | --- | --- | --- | --- | --- | --- | --- | --- | --- | --- | --- | --- | --- | --- | --- | --- | --- | --- | --- | --- | --- | --- | --- | --- | --- | --- | --- | --- | --- | --- | --- | --- | --- | --- | --- | --- | --- | --- | --- | --- | --- | --- | --- | --- | --- | --- | --- | --- | --- | --- | --- | --- | --- | --- | --- | --- | --- | --- | --- | --- | --- | --- | --- | --- | --- | --- | --- | --- | --- | --- | --- | --- | --- | --- | --- | --- | --- | --- | --- | --- | --- | --- | --- | --- | --- | --- | --- | --- | --- | --- | --- | --- | --- | --- | --- | --- | --- | --- | --- | --- | --- | --- | --- | --- | --- | --- | --- | --- | --- | --- | --- | --- | --- | --- | --- | --- | --- | --- | --- | --- | --- | --- | --- | --- | --- | --- | --- | --- | --- | --- | --- | --- | --- | --- | --- | --- | --- | --- | --- | --- | --- | --- | --- | --- | --- | --- | --- | --- | --- | --- | --- | --- | --- | --- | --- | --- | --- | --- | --- | --- | --- | --- | --- | --- | --- | --- | --- | --- | --- | --- | --- | --- | --- | --- | --- | --- | --- | --- | --- | --- | --- | --- | --- | --- | --- | --- | --- | --- | --- | --- | --- | --- | --- | --- | --- | --- | --- | --- | --- | --- | --- | --- | --- | --- | --- | --- | --- | --- | --- | --- | --- | --- | --- | --- | --- | --- | --- | --- | --- | --- | --- | --- | --- | --- | --- | --- | --- | --- | --- | --- | --- | --- | --- | --- | --- | --- | --- | --- | --- | --- | --- | --- | --- | --- | --- | --- | --- | --- | --- | --- | --- | --- | --- | --- | --- | --- | --- | --- | --- | --- | --- | --- | --- | --- | --- | --- | --- | --- | --- | --- | --- | --- | --- | --- | --- | --- | --- | --- | --- | --- | --- | --- | --- | --- | --- | --- | --- | --- | --- | --- | --- | --- | --- | --- | --- | --- | --- | --- | --- | --- | --- | --- | --- | --- | --- | --- | --- | --- | --- | --- | --- | --- | --- | --- | --- | --- | --- | --- | --- | --- | --- | --- | --- | --- | --- | --- | --- | --- | --- | --- | --- | --- | --- | --- | --- | --- | --- | --- | --- | --- | --- | --- | --- | --- | --- | --- | --- | --- | --- | --- | --- | --- | --- | --- | --- | --- | --- | --- | --- | --- | --- | --- | --- | --- | --- | --- | --- | --- | --- | --- | --- | --- | --- | --- | --- | --- | --- | --- | --- | --- | --- | --- | --- | --- | --- | --- | --- | --- | --- | --- | --- | --- | --- | --- | --- | --- | --- | --- | --- | --- | --- | --- | --- | --- | --- | --- | --- | --- | --- | --- | --- | --- | --- | --- | --- | --- | --- | --- | --- | --- | --- | --- | --- | --- | --- | --- | --- | --- | --- | --- | --- | --- | --- | --- | --- | --- | --- | --- | --- | --- | --- | --- | --- | --- | --- | --- | --- | --- | --- | --- | --- | --- | --- | --- | --- | --- | --- | --- | --- | --- | --- | --- | --- | --- | --- | --- | --- | --- | --- | --- | --- | --- | --- | --- | --- | --- | --- | --- | --- | --- | --- | --- | --- | --- | --- | --- | --- | --- | --- | --- | --- | --- | --- | --- | --- | --- | --- | --- | --- | --- | --- | --- | --- | --- | --- | --- | --- | --- | --- | --- | --- | --- | --- | --- | --- | --- | --- | --- | --- | --- | --- | --- | --- | --- | --- | --- | --- | --- | --- | --- | --- | --- | --- | --- | --- | --- | --- | --- | --- | --- | --- | --- | --- | --- | --- | --- | --- | --- | --- | --- | --- | --- | --- | --- | --- | --- | --- | --- | --- | --- | --- | --- | --- | --- | --- | --- | --- | --- | --- | --- | --- | --- | --- | --- | --- | --- | --- | --- | --- | --- | --- | --- | --- | --- | --- | --- | --- | --- | --- | --- | --- | --- | --- | --- | --- | --- | --- | --- | --- | --- | --- | --- | --- | --- | --- | --- | --- | --- | --- | --- | --- | --- | --- | --- | --- | --- | --- | --- | --- | --- | --- | --- | --- | --- | --- | --- | --- | --- | --- | --- | --- | --- | --- | --- | --- | --- | --- | --- | --- | --- | --- | --- | --- | --- | --- | --- | --- | --- | --- | --- | --- | --- | --- | --- | --- | --- | --- | --- | --- | --- | --- | --- | --- | --- | --- | --- | --- | --- | --- | --- | --- | --- | --- | --- | --- | --- | --- | --- | --- | --- | --- | --- | --- | --- | --- | --- | --- | --- | --- | --- | --- | --- | --- | --- | --- | --- | --- | --- | --- | --- | --- | --- | --- | --- | --- | --- | --- | --- | --- | --- | --- | --- | --- | --- | --- | --- | --- | --- | --- | --- | --- | --- | --- | --- | --- | --- | --- | --- | --- | --- | --- | --- | --- | --- | --- | --- | --- | --- | --- | --- | --- | --- | --- | --- | --- | --- | --- | --- | --- | --- | --- | --- | --- | --- | --- | --- | --- | --- | --- | --- | --- | --- | --- | --- | --- | --- | --- | --- | --- | --- | --- | --- | --- | --- | --- | --- | --- | --- | --- | --- | --- | --- | --- | --- | --- | --- | --- | --- | --- | --- | --- | --- | --- | --- | --- | --- | --- | --- | --- | --- | --- | --- | --- | --- | --- | --- | --- | --- | --- | --- | --- | --- | --- | --- | --- | --- | --- | --- | --- | --- | --- | --- | --- | --- | --- | --- | --- | --- | --- | --- | --- | --- | --- | --- | --- | --- | --- | --- | --- | --- | --- | --- | --- | --- | --- | --- | --- | --- | --- | --- | --- | --- | --- | --- | --- | --- | --- | --- | --- | --- | --- | --- | --- | --- | --- | --- | --- | --- | --- | --- | --- | --- | --- | --- | --- | --- | --- | --- | --- | --- | --- | --- | --- | --- | --- | --- | --- | --- | --- | --- | --- | --- | --- | --- | --- | --- | --- | --- | --- | --- | --- | --- | --- | --- | --- | --- | --- | --- | --- | --- | --- | --- | --- | --- | --- | --- | --- | --- | --- | --- | --- | --- | --- | --- | --- | --- | --- | --- | --- | --- | --- | --- | --- | --- | --- | --- | --- | --- | --- | --- | --- | --- | --- | --- | --- | --- | --- | --- | --- | --- | --- | --- | --- | --- | --- | --- | --- | --- | --- | --- | --- | --- | --- | --- | --- | --- | --- | --- | --- | --- | --- | --- | --- | --- | --- | --- | --- | --- | --- | --- | --- | --- | --- | --- | --- | --- | --- | --- | --- | --- | --- | --- | --- | --- | --- | --- | --- | --- | --- | --- | --- | --- | --- | --- | --- | --- | --- | --- | --- | --- | --- | --- | --- | --- | --- | --- | --- | --- | --- | --- | --- | --- | --- | --- | --- | --- | --- | --- | --- | --- | --- | --- | --- | --- | --- | --- | --- | --- | --- | --- | --- | --- | --- | --- | --- | --- | --- | --- | --- | --- | --- | --- | --- | --- | --- | --- | --- | --- | --- | --- | --- | --- | --- | --- | --- | --- | --- | --- | --- | --- | --- | --- | --- | --- | --- | --- | --- | --- | --- | --- | --- | --- | --- | --- | --- | --- | --- | --- | --- | --- | --- | --- | --- | --- | --- | --- | --- | --- | --- | --- | --- | --- | --- | --- | --- | --- | --- | --- | --- | --- | --- | --- | --- | --- | --- | --- | --- | --- | --- | --- | --- | --- | --- | --- | --- | --- | --- | --- | --- | --- | --- | --- | --- | --- | --- | --- | --- | --- | --- | --- | --- | --- | --- | --- | --- | --- | --- | --- | --- | --- | --- | --- | --- | --- | --- | --- | --- | --- | --- | --- | --- | --- | --- | --- | --- | --- | --- | --- | --- | --- | --- | --- | --- | --- | --- | --- | --- | --- | --- | --- | --- | --- | --- | --- | --- | --- | --- | --- | --- | --- | --- | --- | --- | --- | --- | --- | --- | --- | --- | --- | --- | --- | --- | --- | --- | --- | --- | --- | --- | --- | --- | --- | --- | --- | --- | --- | --- | --- | --- | --- | --- | --- | --- | --- | --- | --- | --- | --- | --- | --- | --- | --- | --- | --- | --- | --- | --- | --- | --- | --- | --- | --- | --- | --- | --- | --- | --- | --- | --- | --- | --- | --- | --- | --- | --- | --- | --- | --- | --- | --- | --- | --- | --- | --- | --- | --- | --- | --- | --- | --- | --- | --- | --- | --- | --- | --- | --- | --- | --- | --- | --- | --- | --- | --- | --- | --- | --- | --- | --- | --- | --- | --- | --- | --- | --- | --- | --- | --- | --- | --- | --- | --- | --- | --- | --- | --- | --- | --- | --- | --- | --- | --- | --- | --- | --- | --- | --- | --- | --- | --- | --- | --- | --- | --- | --- | --- | --- | --- | --- | --- | --- | --- | --- | --- | --- | --- | --- | --- | --- | --- | --- | --- | --- | --- | --- | --- | --- | --- | --- | --- | --- | --- | --- | --- | --- | --- | --- | --- | --- | --- | --- | --- | --- | --- | --- | --- | --- | --- | --- | --- | --- | --- | --- | --- | --- | --- | --- | --- | --- | --- | --- | --- | --- | --- | --- | --- | --- | --- | --- | --- | --- | --- | --- | --- | --- | --- | --- | --- | --- | --- | --- | --- | --- | --- | --- | --- | --- | --- | --- | --- | --- | --- | --- | --- | --- | --- | --- | --- | --- | --- | --- | --- | --- | --- | --- | --- | --- | --- | --- | --- | --- | --- | --- | --- | --- | --- | --- | --- | --- | --- | --- | --- | --- | --- | --- | --- | --- | --- | --- | --- | --- | --- | --- | --- | --- | --- | --- | --- | --- | --- | --- | --- | --- | --- | --- | --- | --- | --- | --- | --- | --- | --- | --- | --- | --- | --- | --- | --- | --- | --- | --- | --- | --- | --- | --- | --- | --- | --- | --- | --- | --- | --- | --- | --- | --- | --- | --- | --- | --- | --- | --- | --- | --- | --- | --- | --- | --- | --- | --- | --- | --- | --- | --- | --- | --- | --- | --- | --- | --- | --- | --- | --- | --- | --- | --- | --- | --- | --- | --- | --- | --- | --- | --- | --- | --- | --- | --- | --- | --- | --- | --- | --- | --- | --- | --- | --- | --- | --- | --- | --- | --- | --- | --- | --- | --- | --- | --- | --- | --- | --- | --- | --- | --- | --- | --- | --- | --- | --- | --- | --- | --- | --- | --- | --- | --- | --- | --- | --- | --- | --- | --- | --- | --- | --- | --- | --- | --- | --- | --- | --- | --- | --- | --- | --- | --- | --- | --- | --- | --- | --- | --- | --- | --- | --- | --- | --- | --- | --- | --- | --- | --- | --- | --- | --- | --- | --- | --- | --- | --- | --- | --- | --- | --- | --- | --- | --- | --- | --- | --- | --- | --- | --- | --- | --- | --- | --- | --- | --- | --- | --- | --- | --- | --- | --- | --- | --- | --- | --- | --- | --- | --- | --- | --- | --- | --- | --- | --- | --- | --- | --- | --- | --- | --- | --- | --- | --- | --- | --- | --- | --- | --- | --- | --- | --- | --- | --- | --- | --- | --- | --- | --- | --- | --- | --- | --- | --- | --- | --- | --- | --- | --- | --- | --- | --- | --- | --- | --- | --- | --- | --- | --- | --- | --- | --- | --- | --- | --- | --- | --- | --- | --- | --- | --- | --- | --- | --- | --- | --- | --- | --- | --- | --- | --- | --- | --- | --- | --- | --- | --- | --- | --- | --- | --- | --- | --- | --- | --- | --- | --- | --- | --- | --- | --- | --- | --- | --- | --- | --- | --- | --- | --- | --- | --- | --- | --- | --- | --- | --- | --- | --- | --- | --- | --- | --- | --- | --- | --- | --- | --- | --- | --- | --- | --- | --- | --- | --- | --- | --- | --- | --- | --- | --- | --- | --- | --- | --- | --- | --- | --- | --- | --- | --- | --- | --- | --- | --- | --- | --- | --- | --- | --- | --- | --- | --- | --- | --- | --- | --- | --- | --- | --- | --- | --- | --- | --- | --- | --- | --- | --- | --- | --- | --- | --- | --- | --- | --- | --- | --- | --- | --- | --- | --- | --- | --- | --- | --- | --- | --- | --- | --- | --- | --- | --- | --- | --- | --- | --- | --- | --- | --- | --- | --- | --- | --- | --- | --- | --- | --- | --- | --- | --- | --- | --- | --- | --- | --- | --- | --- | --- | --- | --- | --- | --- | --- | --- | --- | --- | --- | --- | --- | --- | --- | --- | --- | --- | --- | --- | --- | --- | --- | --- | --- | --- | --- | --- | --- | --- | --- | --- | --- | --- | --- | --- | --- | --- | --- | --- | --- | --- | --- | --- | --- | --- | --- | --- | --- | --- | --- | --- | --- | --- | --- | --- | --- | --- | --- | --- | --- | --- | --- | --- | --- | --- | --- | --- | --- | --- | --- | --- | --- | --- | --- | --- | --- | --- | --- | --- | --- | --- | --- | --- | --- | --- | --- | --- | --- | --- | --- | --- | --- | --- | --- | --- | --- | --- | --- | --- | --- | --- | --- | --- | --- | --- | --- | --- | --- | --- | --- | --- | --- | --- | --- | --- | --- | --- | --- | --- | --- | --- | --- | --- | --- | --- | --- | --- | --- | --- | --- | --- | --- | --- | --- | --- | --- | --- | --- | --- | --- | --- | --- | --- | --- | --- | --- | --- | --- | --- | --- | --- | --- | --- | --- | --- | --- | --- | --- | --- | --- | --- | --- | --- | --- | --- | --- | --- | --- | --- | --- | --- | --- | --- | --- | --- | --- | --- | --- | --- | --- | --- | --- | --- | --- | --- | --- | --- | --- | --- | --- | --- | --- | --- | --- | --- | --- | --- | --- | --- | --- | --- | --- | --- | --- | --- | --- | --- | --- | --- | --- | --- | --- | --- | --- | --- | --- | --- | --- | --- | --- | --- | --- | --- | --- | --- | --- | --- | --- | --- | --- | --- | --- | --- | --- | --- | --- | --- | --- | --- | --- | --- | --- | --- | --- | --- | --- | --- | --- | --- | --- | --- | --- | --- | --- | --- | --- | --- | --- | --- | --- | --- | --- | --- | --- | --- | --- | --- | --- | --- | --- | --- | --- | --- | --- | --- | --- | --- | --- | --- | --- | --- | --- | --- | --- | --- | --- | --- | --- | --- | --- | --- | --- | --- | --- | --- | --- | --- | --- | --- | --- | --- | --- | --- | --- | --- | --- | --- | --- | --- | --- | --- | --- | --- | --- | --- | --- | --- | --- | --- | --- | --- | --- | --- | --- | --- | --- | --- | --- | --- | --- | --- | --- | --- | --- | --- | --- | --- | --- | --- | --- | --- | --- | --- | --- | --- | --- | --- | --- | --- | --- | --- | --- | --- | --- | --- | --- | --- | --- | --- | --- | --- | --- | --- | --- | --- | --- | --- | --- | --- | --- | --- | --- | --- | --- | --- | --- | --- | --- | --- | --- | --- | --- | --- | --- | --- | --- | --- | --- | --- | --- | --- | --- | --- | --- | --- | --- | --- | --- | --- | --- | --- | --- | --- | --- | --- | --- | --- | --- | --- | --- | --- | --- | --- | --- | --- | --- | --- | --- | --- | --- | --- | --- | --- | --- | --- | --- | --- | --- | --- | --- | --- | --- | --- | --- | --- | --- | --- | --- | --- | --- | --- | --- | --- | --- | --- | --- | --- | --- | --- | --- | --- | --- | --- | --- | --- | --- | --- | --- | --- | --- | --- | --- | --- | --- | --- | --- | --- | --- | --- | --- | --- | --- | --- | --- | --- | --- | --- | --- | --- | --- | --- | --- | --- | --- | --- | --- | --- | --- | --- | --- | --- | --- | --- | --- | --- | --- | --- | --- | --- | --- | --- | --- | --- | --- | --- | --- | --- | --- | --- | --- | --- | --- | --- | --- | --- | --- | --- | --- | --- | --- | --- | --- | --- | --- | --- | --- | --- | --- | --- | --- | --- | --- | --- | --- | --- | --- | --- | --- | --- | --- | --- | --- | --- | --- | --- | --- | --- | --- | --- | --- | --- | --- | --- | --- | --- | --- | --- | --- | --- | --- | --- | --- | --- | --- | --- | --- | --- | --- | --- | --- | --- | --- | --- | --- | --- | --- | --- | --- | --- | --- | --- | --- | --- | --- | --- | --- | --- | --- | --- | --- | --- | --- | --- | --- | --- | --- | --- | --- | --- | --- | --- | --- | --- | --- | --- | --- | --- | --- | --- | --- | --- | --- | --- | --- | --- | --- | --- | --- | --- | --- | --- | --- | --- | --- | --- | --- | --- | --- | --- | --- | --- | --- | --- | --- | --- | --- | --- | --- | --- | --- | --- | --- | --- | --- | --- | --- | --- | --- | --- | --- | --- | --- | --- | --- | --- | --- | --- | --- | --- | --- | --- | --- | --- | --- | --- | --- | --- | --- | --- | --- | --- | --- | --- | --- | --- | --- | --- | --- | --- | --- | --- | --- | --- | --- | --- | --- | --- | --- | --- | --- | --- | --- | --- | --- | --- | --- | --- | --- | --- | --- | --- | --- | --- | --- | --- | --- | --- | --- | --- | --- | --- | --- | --- | --- | --- | --- | --- | --- | --- | --- | --- | --- | --- | --- | --- | --- | --- | --- | --- | --- | --- | --- | --- | --- | --- | --- | --- | --- | --- | --- | --- | --- | --- | --- | --- | --- | --- | --- | --- | --- | --- | --- | --- | --- | --- | --- | --- | --- | --- | --- | --- | --- | --- | --- | --- | --- | --- | --- | --- | --- | --- | --- | --- | --- | --- | --- | --- | --- | --- | --- | --- | --- | --- | --- | --- | --- | --- | --- | --- | --- | --- | --- | --- | --- | --- | --- | --- | --- | --- | --- | --- | --- | --- | --- | --- | --- | --- | --- | --- | --- | --- | --- | --- | --- | --- | --- | --- | --- | --- | --- | --- | --- | --- | --- | --- | --- | --- | --- | --- | --- | --- | --- | --- | --- | --- | --- | --- | --- | --- | --- | --- | --- | --- | --- | --- | --- | --- | --- | --- | --- | --- | --- | --- | --- | --- | --- | --- | --- | --- | --- | --- | --- | --- | --- | --- | --- | --- | --- | --- | --- | --- | --- | --- | --- | --- | --- | --- | --- | --- | --- | --- | --- | --- | --- | --- | --- | --- | --- | --- | --- | --- | --- | --- | --- | --- | --- | --- | --- | --- | --- | --- | --- | --- | --- | --- | --- | --- | --- | --- | --- | --- | --- | --- | --- | --- | --- | --- | --- | --- | --- | --- | --- | --- | --- | --- | --- | --- | --- | --- | --- | --- | --- | --- | --- | --- | --- | --- | --- | --- | --- | --- | --- | --- | --- | --- | --- | --- | --- | --- | --- | --- | --- | --- | --- | --- | --- | --- | --- | --- | --- | --- | --- | --- | --- | --- | --- | --- | --- | --- | --- | --- | --- | --- | --- | --- | --- | --- | --- | --- | --- | --- | --- | --- | --- | --- | --- | --- | --- | --- | --- | --- | --- | --- | --- | --- | --- | --- | --- | --- | --- | --- | --- | --- | --- | --- | --- | --- | --- | --- | --- | --- | --- | --- | --- | --- | --- | --- | --- | --- | --- | --- | --- | --- | --- | --- | --- | --- | --- | --- | --- | --- | --- | --- | --- | --- | --- | --- | --- | --- | --- | --- | --- | --- | --- | --- | --- | --- | --- | --- | --- | --- | --- | --- | --- | --- | --- | --- | --- | --- | --- | --- | --- | --- | --- | --- | --- | --- | --- | --- | --- | --- | --- | --- | --- | --- | --- | --- | --- | --- | --- | --- | --- | --- | --- | --- | --- | --- | --- | --- | --- | --- | --- | --- | --- | --- | --- | --- | --- | --- | --- | --- | --- | --- | --- | --- | --- | --- | --- | --- | --- | --- | --- | --- | --- | --- | --- | --- | --- | --- | --- | --- | --- | --- | --- | --- | --- | --- | --- | --- | --- | --- | --- | --- | --- | --- | --- | --- | --- | --- | --- | --- | --- | --- | --- | --- | --- | --- | --- | --- | --- | --- | --- | --- | --- | --- | --- | --- | --- | --- | --- | --- | --- | --- | --- | --- | --- | --- | --- | --- | --- | --- | --- | --- | --- | --- | --- | --- | --- | --- | --- | --- | --- | --- | --- | --- | --- | --- | --- | --- | --- | --- | --- | --- | --- | --- | --- | --- | --- | --- | --- | --- | --- | --- | --- | --- | --- | --- | --- | --- | --- | --- | --- | --- | --- | --- | --- | --- | --- | --- | --- | --- | --- | --- | --- | --- | --- | --- | --- | --- | --- | --- | --- | --- | --- | --- | --- | --- | --- | --- | --- | --- | --- | --- | --- | --- | --- | --- | --- | --- | --- | --- | --- | --- | --- | --- | --- | --- | --- | --- | --- | --- | --- | --- | --- | --- | --- | --- | --- | --- | --- | --- | --- | --- | --- | --- | --- | --- | --- | --- | --- | --- | --- | --- | --- | --- | --- | --- | --- | --- | --- | --- | --- | --- | --- | --- | --- | --- | --- | --- | --- | --- | --- | --- | --- | --- | --- | --- | --- | --- | --- | --- | --- | --- | --- | --- | --- | --- | --- | --- | --- | --- | --- | --- | --- | --- | --- | --- | --- | --- | --- | --- | --- | --- | --- | --- | --- | --- | --- | --- | --- | --- | --- | --- | --- | --- | --- | --- | --- | --- | --- | --- | --- | --- | --- | --- | --- | --- | --- | --- | --- | --- | --- | --- | --- | --- | --- | --- | --- | --- | --- | --- | --- | --- | --- | --- | --- | --- | --- | --- | --- | --- | --- | --- | --- | --- | --- | --- | --- | --- | --- | --- | --- | --- | --- | --- | --- | --- | --- | --- | --- | --- | --- | --- | --- | --- | --- | --- | --- | --- | --- | --- | --- | --- | --- | --- | --- | --- | --- | --- | --- | --- | --- | --- | --- | --- | --- | --- | --- | --- | --- | --- | --- | --- | --- | --- | --- | --- | --- | --- | --- | --- | --- | --- | --- | --- | --- | --- | --- | --- | --- | --- | --- | --- | --- | --- | --- | --- | --- | --- | --- | --- | --- | --- | --- | --- | --- | --- | --- | --- | --- | --- | --- | --- | --- | --- | --- | --- | --- | --- | --- | --- | --- | --- | --- | --- | --- | --- | --- | --- | --- | --- | --- | --- | --- | --- | --- | --- | --- | --- | --- | --- | --- | --- | --- | --- | --- | --- | --- | --- | --- | --- | --- | --- | --- | --- | --- | --- | --- | --- | --- | --- | --- | --- | --- | --- | --- | --- | --- | --- | --- | --- | --- | --- | --- | --- | --- | --- | --- | --- | --- | --- | --- | --- | --- | --- | --- | --- | --- | --- | --- | --- | --- | --- | --- | --- | --- | --- | --- | --- | --- | --- | --- | --- | --- | --- | --- | --- | --- | --- | --- | --- | --- | --- | --- | --- | --- | --- | --- | --- | --- | --- | --- | --- | --- | --- | --- | --- | --- | --- | --- | --- | --- | --- | --- | --- | --- | --- | --- | --- | --- | --- | --- | --- | --- | --- | --- | --- | --- | --- | --- | --- | --- | --- | --- | --- | --- | --- | --- | --- | --- | --- | --- | --- | --- | --- | --- | --- | --- | --- | --- | --- | --- | --- | --- | --- | --- | --- | --- | --- | --- | --- | --- | --- | --- | --- | --- | --- | --- | --- | --- | --- | --- | --- | --- | --- | --- | --- | --- | --- | --- | --- | --- | --- | --- | --- | --- | --- | --- | --- | --- | --- | --- | --- | --- | --- | --- | --- | --- | --- | --- | --- | --- | --- | --- | --- | --- | --- | --- | --- | --- | --- | --- | --- | --- | --- | --- | --- | --- | --- | --- | --- | --- | --- | --- | --- | --- | --- | --- | --- | --- | --- | --- | --- | --- | --- | --- | --- | --- | --- | --- | --- | --- | --- | --- | --- | --- | --- | --- | --- | --- | --- | --- | --- | --- | --- | --- | --- | --- | --- | --- | --- | --- | --- | --- | --- | --- | --- | --- | --- | --- | --- | --- | --- | --- | --- | --- | --- | --- | --- | --- | --- | --- | --- | --- | --- | --- | --- | --- | --- | --- | --- | --- | --- | --- | --- | --- | --- | --- | --- | --- | --- | --- | --- | --- | --- | --- | --- | --- | --- | --- | --- | --- | --- | --- | --- | --- | --- | --- | --- | --- | --- | --- | --- | --- | --- | --- | --- | --- | --- | --- | --- | --- | --- | --- | --- | --- | --- | --- | --- | --- | --- | --- | --- | --- | --- | --- | --- | --- | --- | --- | --- | --- | --- | --- | --- | --- | --- | --- | --- | --- | --- | --- | --- | --- | --- | --- | --- | --- | --- | --- | --- | --- | --- | --- | --- | --- | --- | --- | --- | --- | --- | --- | --- | --- | --- | --- | --- | --- | --- | --- | --- | --- | --- | --- | --- | --- | --- | --- | --- | --- | --- | --- | --- | --- | --- | --- | --- | --- | --- | --- | --- | --- | --- | --- | --- | --- | --- | --- | --- | --- | --- | --- | --- | --- | --- | --- | --- | --- | --- | --- | --- | --- | --- | --- | --- | --- | --- | --- | --- | --- | --- | --- | --- | --- | --- | --- | --- | --- | --- | --- | --- | --- | --- | --- | --- | --- | --- | --- | --- | --- | --- | --- | --- | --- | --- | --- | --- | --- | --- | --- | --- | --- | --- | --- | --- | --- | --- | --- | --- | --- | --- | --- | --- | --- | --- | --- | --- | --- | --- | --- | --- | --- | --- | --- | --- | --- | --- | --- | --- | --- | --- | --- | --- | --- | --- | --- | --- | --- | --- | --- | --- | --- | --- | --- | --- | --- | --- | --- | --- | --- | --- | --- | --- | --- | --- | --- | --- | --- | --- | --- | --- | --- | --- | --- | --- | --- | --- | --- | --- | --- | --- | --- | --- | --- | --- | --- | --- | --- | --- | --- | --- | --- | --- | --- | --- | --- | --- | --- | --- | --- | --- | --- | --- | --- | --- | --- | --- | --- | --- | --- | --- | --- | --- | --- | --- | --- | --- | --- | --- | --- | --- | --- | --- | --- | --- | --- | --- | --- | --- | --- | --- | --- | --- | --- | --- | --- | --- | --- | --- | --- | --- | --- | --- | --- | --- | --- | --- | --- | --- | --- | --- | --- | --- | --- | --- | --- | --- | --- | --- | --- | --- | --- | --- | --- | --- | --- | --- | --- | --- | --- | --- | --- | --- | --- | --- | --- | --- | --- | --- | --- | --- | --- | --- | --- | --- | --- | --- | --- | --- | --- | --- | --- | --- | --- | --- | --- | --- | --- | --- | --- | --- | --- | --- | --- | --- | --- | --- | --- | --- | --- | --- | --- | --- | --- | --- | --- | --- | --- | --- | --- | --- | --- | --- | --- | --- | --- | --- | --- | --- | --- | --- | --- | --- | --- | --- | --- | --- | --- | --- | --- | --- | --- | --- | --- | --- | --- | --- | --- | --- | --- | --- | --- | --- | --- | --- | --- | --- | --- | --- | --- | --- | --- | --- | --- | --- | --- | --- | --- | --- | --- | --- | --- | --- | --- | --- | --- | --- | --- | --- | --- | --- | --- | --- | --- | --- | --- | --- | --- | --- | --- | --- | --- | --- | --- | --- | --- | --- | --- | --- | --- | --- | --- | --- | --- | --- | --- | --- | --- | --- | --- | --- | --- | --- | --- | --- | --- | --- | --- | --- | --- | --- | --- | --- | --- | --- | --- | --- | --- | --- | --- | --- | --- | --- | --- | --- | --- | --- | --- | --- | --- | --- | --- | --- | --- | --- | --- | --- | --- | --- | --- | --- | --- | --- | --- | --- | --- | --- | --- | --- | --- | --- | --- | --- | --- | --- | --- | --- | --- | --- | --- | --- | --- | --- | --- | --- | --- | --- | --- | --- | --- | --- | --- | --- | --- | --- | --- | --- | --- | --- | --- | --- | --- | --- | --- | --- | --- | --- | --- | --- | --- | --- | --- | --- | --- | --- | --- | --- | --- | --- | --- | --- | --- | --- | --- | --- | --- | --- | --- | --- | --- | --- | --- | --- | --- | --- | --- | --- | --- | --- | --- | --- | --- | --- | --- | --- | --- | --- | --- | --- | --- | --- | --- | --- | --- | --- | --- | --- | --- | --- | --- | --- | --- | --- | --- | --- | --- | --- | --- | --- | --- | --- | --- | --- | --- | --- | --- | --- | --- | --- | --- | --- | --- | --- | --- | --- | --- | --- | --- | --- | --- | --- | --- | --- | --- | --- | --- | --- | --- | --- | --- | --- | --- | --- | --- | --- | --- | --- | --- | --- | --- | --- | --- | --- | --- | --- | --- | --- | --- | --- | --- | --- | --- | --- | --- | --- | --- | --- | --- | --- | --- | --- | --- | --- | --- | --- | --- | --- | --- | --- | --- | --- | --- | --- | --- | --- | --- | --- | --- | --- | --- | --- | --- | --- | --- | --- | --- | --- | --- | --- | --- | --- | --- | --- | --- | --- | --- | --- | --- | --- | --- | --- | --- | --- | --- | --- | --- | --- | --- | --- | --- | --- | --- | --- | --- | --- | --- | --- | --- | --- | --- | --- | --- | --- | --- | --- | --- | --- | --- | --- | --- | --- | --- | --- | --- | --- | --- | --- | --- | --- | --- | --- | --- | --- | --- | --- | --- | --- | --- | --- | --- | --- | --- | --- | --- | --- | --- | --- | --- | --- | --- | --- | --- | --- | --- | --- | --- | --- | --- | --- | --- | --- | --- | --- | --- | --- | --- | --- | --- | --- | --- | --- | --- | --- | --- | --- | --- | --- | --- | --- | --- | --- | --- | --- | --- | --- | --- | --- | --- | --- | --- | --- | --- | --- | --- | --- | --- | --- | --- | --- | --- | --- | --- | --- | --- | --- | --- | --- | --- | --- | --- | --- | --- | --- | --- | --- | --- | --- | --- | --- | --- | --- | --- | --- | --- | --- | --- | --- | --- | --- | --- | --- | --- | --- | --- | --- | --- | --- | --- | --- | --- | --- | --- | --- | --- | --- | --- | --- | --- | --- | --- | --- | --- | --- | --- | --- | --- | --- | --- | --- | --- | --- | --- | --- | --- | --- | --- | --- | --- | --- | --- | --- | --- | --- | --- | --- | --- | --- | --- | --- | --- | --- | --- | --- | --- | --- | --- | --- | --- | --- | --- | --- | --- | --- | --- | --- | --- | --- | --- | --- | --- | --- | --- | --- | --- | --- | --- | --- | --- | --- | --- | --- | --- | --- | --- | --- | --- | --- | --- | --- | --- | --- | --- | --- | --- | --- | --- | --- | --- | --- | --- | --- | --- | --- | --- | --- | --- | --- | --- | --- | --- | --- | --- | --- | --- | --- | --- | --- | --- | --- | --- | --- | --- | --- | --- | --- | --- | --- | --- | --- | --- | --- | --- | --- | --- | --- | --- | --- | --- | --- | --- | --- | --- | --- | --- | --- | --- | --- | --- | --- | --- | --- | --- | --- | --- | --- | --- | --- | --- | --- | --- | --- | --- | --- | --- | --- | --- | --- | --- | --- | --- | --- | --- | --- | --- | --- | --- | --- | --- | --- | --- | --- | --- | --- | --- | --- | --- | --- | --- | --- | --- | --- | --- | --- | --- | --- | --- | --- | --- | --- | --- | --- | --- | --- | --- | --- | --- | --- | --- | --- | --- | --- | --- | --- | --- | --- | --- | --- | --- | --- | --- | --- | --- | --- | --- | --- | --- | --- | --- | --- | --- | --- | --- | --- | --- | --- | --- | --- | --- | --- | --- | --- | --- | --- | --- | --- | --- | --- | --- | --- | --- | --- | --- | --- | --- | --- | --- | --- | --- | --- | --- | --- | --- | --- | --- | --- | --- | --- | --- | --- | --- | --- | --- | --- | --- | --- | --- | --- | --- | --- | --- | --- | --- | --- | --- | --- | --- | --- | --- | --- | --- | --- | --- | --- | --- | --- | --- | --- | --- | --- | --- | --- | --- | --- | --- | --- | --- | --- | --- | --- | --- | --- | --- | --- | --- | --- | --- | --- | --- | --- | --- | --- | --- | --- | --- | --- | --- | --- | --- | --- | --- | --- | --- | --- | --- | --- | --- | --- | --- | --- | --- | --- | --- | --- | --- | --- | --- | --- | --- | --- | --- | --- | --- | --- | --- | --- | --- | --- | --- | --- | --- | --- | --- | --- | --- | --- | --- | --- | --- | --- | --- | --- | --- | --- | --- | --- | --- | --- | --- | --- | --- | --- | --- | --- | --- | --- | --- | --- | --- | --- | --- | --- | --- | --- | --- | --- | --- | --- | --- | --- | --- | --- | --- | --- | --- | --- | --- | --- | --- | --- | --- | --- | --- | --- |
|  |
|  |
|  |
|  |
|  |
|  |
|  |
| 2.Supplementary Table 2: Five MR Models estimated the causal relationship between 65 metabolites, or metabolite ratios, and MDD risk and tested for heterogeneity and horizontal pleiotropy.   \| CLASS \| Metabolite name and number \| Method \| SNP(n) \| pval \| OR \| 95%CI \| Heterogeneity \|  \| Pleiotropy \|  \| \| --- \| --- \| --- \| --- \| --- \| --- \| --- \| --- \| --- \| --- \| --- \| \| Qvalue \| P \| intercept \| P \| \| Lipids \| 1-stearoyl-2-arachidonoyl-GPI (18:0/20:4) levels \| MR Egger \| 19 \| 0.220 \| 1.03 \| 0.99-1.07 \| 14.36 \| 0.641 \|  \|  \| \|  \| GCST90199649 \| Inverse variance weighted \| 19 \| 0.026 \| 1.02 \| 1-1.04 \| 14.46 \| 0.698 \| 0.00 \| 0.756 \| \|  \|  \| Weighted median \| 19 \| 0.064 \| 1.03 \| 1-1.05 \|  \|  \|  \|  \| \|  \|  \| Simple mode \| 19 \| 0.814 \| 1.01 \| 0.96-1.06 \|  \|  \|  \|  \| \|  \|  \| Weighted mode \| 19 \| 0.015 \| 1.05 \| 1.01-1.08 \|  \|  \|  \|  \| \|  \|  \|  \|  \|  \|  \|  \|  \|  \|  \|  \| \| Lipids \| Alpha-hydroxyisocaproate levels \| MR Egger \| 22 \| 0.820 \| 0.99 \| 0.91-1.08 \| 33.89 \| 0.027 \|  \|  \| \|  \| GCST90199658 \| Inverse variance weighted \| 22 \| 0.042 \| 0.97 \| 0.93-1 \| 34.53 \| 0.032 \| 0.00 \| 0.548 \| \|  \|  \| Weighted median \| 22 \| 0.145 \| 0.97 \| 0.94-1.01 \|  \|  \|  \|  \| \|  \|  \| Simple mode \| 22 \| 0.409 \| 0.97 \| 0.91-1.04 \|  \|  \|  \|  \| \|  \|  \| Weighted mode \| 22 \| 0.399 \| 0.97 \| 0.92-1.03 \|  \|  \|  \|  \| \|  \|  \|  \|  \|  \|  \|  \|  \|  \|  \|  \| \| Lipids \| 2-linoleoylglycerol (18:2) levels \| MR Egger \| 19 \| 0.096 \| 0.95 \| 0.9-1.01 \| 6.81 \| 0.986 \|  \|  \| \|  \| GCST90199685 \| Inverse variance weighted \| 19 \| 0.003 \| 0.96 \| 0.94-0.99 \| 7.10 \| 0.989 \| 0.00 \| 0.598 \| \|  \|  \| Weighted median \| 19 \| 0.011 \| 0.96 \| 0.93-0.99 \|  \|  \|  \|  \| \|  \|  \| Simple mode \| 19 \| 0.149 \| 0.96 \| 0.91-1.01 \|  \|  \|  \|  \| \|  \|  \| Weighted mode \| 19 \| 0.146 \| 0.96 \| 0.91-1.01 \|  \|  \|  \|  \| \|  \|  \|  \|  \|  \|  \|  \|  \|  \|  \|  \| \| Lipids \| Docosatrienoate (22:3n3) levels \| MR Egger \| 21 \| 0.096 \| 0.96 \| 0.92-1 \| 21.25 \| 0.323 \|  \|  \| \|  \| GCST90199710 \| Inverse variance weighted \| 21 \| 0.003 \| 0.97 \| 0.95-0.99 \| 21.48 \| 0.370 \| 0.00 \| 0.657 \| \|  \|  \| Weighted median \| 21 \| 0.002 \| 0.95 \| 0.93-0.98 \|  \|  \|  \|  \| \|  \|  \| Simple mode \| 21 \| 0.377 \| 0.98 \| 0.93-1.03 \|  \|  \|  \|  \| \|  \|  \| Weighted mode \| 21 \| 0.010 \| 0.95 \| 0.92-0.99 \|  \|  \|  \|  \| \|  \|  \|  \|  \|  \|  \|  \|  \|  \|  \|  \| \| Lipids \| 1-linoleoyl-gpc (18:2) levels \| MR Egger \| 22 \| 0.088 \| 0.95 \| 0.89-1.01 \| 23.20 \| 0.279 \|  \|  \| \|  \| GCST90199742 \| Inverse variance weighted \| 22 \| 0.005 \| 0.96 \| 0.94-0.99 \| 23.63 \| 0.311 \| 0.00 \| 0.549 \| \|  \|  \| Weighted median \| 22 \| 0.231 \| 0.98 \| 0.94-1.01 \|  \|  \|  \|  \| \|  \|  \| Simple mode \| 22 \| 0.613 \| 0.98 \| 0.91-1.06 \|  \|  \|  \|  \| \|  \|  \| Weighted mode \| 22 \| 0.648 \| 0.98 \| 0.9-1.07 \|  \|  \|  \|  \| \|  \|  \|  \|  \|  \|  \|  \|  \|  \|  \|  \| \| Lipids \| 1-stearoyl-GPE (18:0) levels \| MR Egger \| 36 \| 0.422 \| 1.02 \| 0.97-1.08 \| 41.79 \| 0.168 \|  \|  \| \|  \| GCST90199772 \| Inverse variance weighted \| 36 \| 0.003 \| 1.03 \| 1.01-1.05 \| 41.92 \| 0.196 \| 0.00 \| 0.754 \| \|  \|  \| Weighted median \| 36 \| 0.063 \| 1.03 \| 1-1.06 \|  \|  \|  \|  \| \|  \|  \| Simple mode \| 36 \| 0.142 \| 1.05 \| 0.99-1.11 \|  \|  \|  \|  \| \|  \|  \| Weighted mode \| 36 \| 0.146 \| 1.03 \| 0.99-1.08 \|  \|  \|  \|  \| \|  \|  \|  \|  \|  \|  \|  \|  \|  \|  \|  \| \| Lipids \| Malonylcarnitine levels \| MR Egger \| 20 \| 0.472 \| 1.02 \| 0.96-1.09 \| 21.48 \| 0.256 \|  \|  \| \|  \| GCST90199776 \| Inverse variance weighted \| 20 \| 0.035 \| 1.03 \| 1-1.05 \| 21.48 \| 0.311 \| 0.00 \| 0.957 \| \|  \|  \| Weighted median \| 20 \| 0.148 \| 1.02 \| 0.99-1.06 \|  \|  \|  \|  \| \|  \|  \| Simple mode \| 20 \| 0.678 \| 1.01 \| 0.95-1.08 \|  \|  \|  \|  \| \|  \|  \| Weighted mode \| 20 \| 0.725 \| 1.01 \| 0.95-1.08 \|  \|  \|  \|  \| \|  \|  \|  \|  \|  \|  \|  \|  \|  \|  \|  \| \| Lipids \| 2-hydroxy-3-methylvalerate levels \| MR Egger \| 28 \| 0.011 \| 0.92 \| 0.87-0.98 \| 31.32 \| 0.217 \|  \|  \| \|  \| GCST90199786 \| Inverse variance weighted \| 28 \| 0.034 \| 0.97 \| 0.95-1 \| 36.17 \| 0.112 \| 0.01 \| 0.055 \| \|  \|  \| Weighted median \| 28 \| 0.773 \| 1.00 \| 0.96-1.03 \|  \|  \|  \|  \| \|  \|  \| Simple mode \| 28 \| 0.076 \| 0.94 \| 0.88-1 \|  \|  \|  \|  \| \|  \|  \| Weighted mode \| 28 \| 0.696 \| 0.99 \| 0.95-1.04 \|  \|  \|  \|  \| \|  \|  \|  \|  \|  \|  \|  \|  \|  \|  \|  \| \| Lipids \| 1-arachidonoyl-gpc (20:4n6) levels \| MR Egger \| 24 \| 0.043 \| 1.03 \| 1-1.06 \| 37.47 \| 0.021 \|  \|  \| \|  \| GCST90199788 \| Inverse variance weighted \| 24 \| 0.041 \| 1.02 \| 1-1.04 \| 39.39 \| 0.018 \| 0.00 \| 0.300 \| \|  \|  \| Weighted median \| 24 \| 0.001 \| 1.03 \| 1.01-1.04 \|  \|  \|  \|  \| \|  \|  \| Simple mode \| 24 \| 0.828 \| 1.01 \| 0.94-1.08 \|  \|  \|  \|  \| \|  \|  \| Weighted mode \| 24 \| 0.008 \| 1.03 \| 1.01-1.05 \|  \|  \|  \|  \| \|  \|  \|  \|  \|  \|  \|  \|  \|  \|  \|  \| \| Lipids \| Pregnanediol-3-glucuronide levels \| MR Egger \| 30 \| 0.037 \| 0.96 \| 0.92-1 \| 30.21 \| 0.353 \|  \|  \| \|  \| GCST90199917 \| Inverse variance weighted \| 30 \| 0.002 \| 0.97 \| 0.96-0.99 \| 31.17 \| 0.357 \| 0.00 \| 0.353 \| \|  \|  \| Weighted median \| 30 \| 0.062 \| 0.98 \| 0.95-1 \|  \|  \|  \|  \| \|  \|  \| Simple mode \| 30 \| 0.299 \| 0.98 \| 0.93-1.02 \|  \|  \|  \|  \| \|  \|  \| Weighted mode \| 30 \| 0.072 \| 0.97 \| 0.94-1 \|  \|  \|  \|  \| \|  \|  \|  \|  \|  \|  \|  \|  \|  \|  \|  \| \| Lipids \| 1-stearoyl-2-linoleoyl-gpc (18:0/18:2) levels \| MR Egger \| 19 \| 0.359 \| 0.97 \| 0.9-1.04 \| 29.75 \| 0.028 \|  \|  \| \|  \| GCST90200037 \| Inverse variance weighted \| 19 \| 0.037 \| 0.97 \| 0.94-1 \| 29.76 \| 0.040 \| 0.00 \| 0.951 \| \|  \|  \| Weighted median \| 19 \| 0.022 \| 0.96 \| 0.93-0.99 \|  \|  \|  \|  \| \|  \|  \| Simple mode \| 19 \| 0.588 \| 0.98 \| 0.91-1.05 \|  \|  \|  \|  \| \|  \|  \| Weighted mode \| 19 \| 0.006 \| 0.95 \| 0.91-0.98 \|  \|  \|  \|  \| \|  \|  \|  \|  \|  \|  \|  \|  \|  \|  \|  \| \| Lipids \| 1-palmitoyl-2-docosahexaenoyl-gpc (16:0/22:6) levels \| MR Egger \| 21 \| 0.091 \| 1.07 \| 0.99-1.14 \| 21.53 \| 0.308 \|  \|  \| \|  \| GCST90200043 \| Inverse variance weighted \| 21 \| 0.029 \| 1.03 \| 1-1.05 \| 22.93 \| 0.292 \| -0.01 \| 0.280 \| \|  \|  \| Weighted median \| 21 \| 0.315 \| 1.02 \| 0.98-1.05 \|  \|  \|  \|  \| \|  \|  \| Simple mode \| 21 \| 0.672 \| 1.01 \| 0.95-1.08 \|  \|  \|  \|  \| \|  \|  \| Weighted mode \| 21 \| 0.742 \| 1.01 \| 0.95-1.07 \|  \|  \|  \|  \| \|  \|  \|  \|  \|  \|  \|  \|  \|  \|  \|  \| \| Lipids \| 1-palmitoyl-2-arachidonoyl-GPE (16:0/20:4) levels \| MR Egger \| 17 \| 0.645 \| 1.01 \| 0.97-1.05 \| 22.26 \| 0.101 \|  \|  \| \|  \| GCST90200054 \| Inverse variance weighted \| 17 \| 0.025 \| 1.03 \| 1-1.05 \| 23.40 \| 0.103 \| 0.00 \| 0.395 \| \|  \|  \| Weighted median \| 17 \| 0.291 \| 1.01 \| 0.99-1.04 \|  \|  \|  \|  \| \|  \|  \| Simple mode \| 17 \| 0.449 \| 1.02 \| 0.97-1.09 \|  \|  \|  \|  \| \|  \|  \| Weighted mode \| 17 \| 0.355 \| 1.01 \| 0.99-1.04 \|  \|  \|  \|  \| \|  \|  \|  \|  \|  \|  \|  \|  \|  \|  \|  \| \| Lipids \| 1-(1-enyl-palmitoyl)-2-palmitoleoyl-GPC (P-16:0/16:1) levels \| MR Egger \| 31 \| 0.146 \| 1.03 \| 0.99-1.07 \| 38.52 \| 0.111 \|  \|  \| \|  \| GCST90200070 \| Inverse variance weighted \| 31 \| 0.038 \| 1.02 \| 1-1.04 \| 38.92 \| 0.128 \| 0.00 \| 0.588 \| \|  \|  \| Weighted median \| 31 \| 0.135 \| 1.02 \| 0.99-1.04 \|  \|  \|  \|  \| \|  \|  \| Simple mode \| 31 \| 0.778 \| 1.01 \| 0.95-1.06 \|  \|  \|  \|  \| \|  \|  \| Weighted mode \| 31 \| 0.190 \| 1.02 \| 0.99-1.04 \|  \|  \|  \|  \| \|  \|  \|  \|  \|  \|  \|  \|  \|  \|  \|  \| \| Lipids \| 1-oleoyl-2-linoleoyl-GPE (18:1/18:2) levels \| MR Egger \| 25 \| 0.037 \| 0.97 \| 0.94-1 \| 25.82 \| 0.310 \|  \|  \| \|  \| GCST90200082 \| Inverse variance weighted \| 25 \| 0.021 \| 0.98 \| 0.97-1 \| 27.23 \| 0.294 \| 0.00 \| 0.274 \| \|  \|  \| Weighted median \| 25 \| 0.001 \| 0.96 \| 0.94-0.99 \|  \|  \|  \|  \| \|  \|  \| Simple mode \| 25 \| 0.330 \| 0.97 \| 0.93-1.02 \|  \|  \|  \|  \| \|  \|  \| Weighted mode \| 25 \| 0.013 \| 0.97 \| 0.95-0.99 \|  \|  \|  \|  \| \|  \|  \|  \|  \|  \|  \|  \|  \|  \|  \|  \| \| Lipids \| 1-linoleoyl-2-linolenoyl-GPC (18:2/18:3) levels \| MR Egger \| 16 \| 0.046 \| 0.94 \| 0.89-0.99 \| 15.91 \| 0.319 \|  \|  \| \|  \| GCST90200095 \| Inverse variance weighted \| 16 \| 0.017 \| 0.97 \| 0.94-0.99 \| 17.62 \| 0.283 \| 0.00 \| 0.240 \| \|  \|  \| Weighted median \| 16 \| 0.001 \| 0.94 \| 0.91-0.97 \|  \|  \|  \|  \| \|  \|  \| Simple mode \| 16 \| 0.406 \| 0.96 \| 0.89-1.05 \|  \|  \|  \|  \| \|  \|  \| Weighted mode \| 16 \| 0.005 \| 0.94 \| 0.91-0.98 \|  \|  \|  \|  \| \|  \|  \|  \|  \|  \|  \|  \|  \|  \|  \|  \| \| Lipids \| Linoleoyl-arachidonoyl-glycerol (18:2/20:4) [1] levels \| MR Egger \| 15 \| 0.077 \| 1.04 \| 1-1.08 \| 16.44 \| 0.226 \|  \|  \| \|  \| GCST90200103 \| Inverse variance weighted \| 15 \| 0.013 \| 1.03 \| 1.01-1.05 \| 17.11 \| 0.250 \| 0.00 \| 0.479 \| \|  \|  \| Weighted median \| 15 \| 0.001 \| 1.05 \| 1.02-1.08 \|  \|  \|  \|  \| \|  \|  \| Simple mode \| 15 \| 0.138 \| 1.04 \| 0.99-1.1 \|  \|  \|  \|  \| \|  \|  \| Weighted mode \| 15 \| 0.006 \| 1.05 \| 1.02-1.08 \|  \|  \|  \|  \| \|  \|  \|  \|  \|  \|  \|  \|  \|  \|  \|  \| \| Lipids \| Glycosyl-N-tricosanoyl-sphingadienine (d18:2/23:0) levels \| MR Egger \| 22 \| 0.563 \| 0.98 \| 0.94-1.04 \| 20.76 \| 0.411 \|  \|  \| \|  \| GCST90200114 \| Inverse variance weighted \| 22 \| 0.007 \| 0.97 \| 0.95-0.99 \| 21.14 \| 0.450 \| 0.00 \| 0.553 \| \|  \|  \| Weighted median \| 22 \| 0.055 \| 0.97 \| 0.94-1 \|  \|  \|  \|  \| \|  \|  \| Simple mode \| 22 \| 0.727 \| 0.99 \| 0.94-1.04 \|  \|  \|  \|  \| \|  \|  \| Weighted mode \| 22 \| 0.106 \| 0.97 \| 0.93-1.01 \|  \|  \|  \|  \| \|  \|  \|  \|  \|  \|  \|  \|  \|  \|  \|  \| \| Lipids \| Taurodeoxycholate levels \| MR Egger \| 19 \| 0.977 \| 1.00 \| 0.94-1.06 \| 14.42 \| 0.637 \|  \|  \| \|  \| GCST90200334 \| Inverse variance weighted \| 19 \| 0.032 \| 0.97 \| 0.95-1 \| 15.54 \| 0.625 \| 0.00 \| 0.305 \| \|  \|  \| Weighted median \| 19 \| 0.476 \| 0.99 \| 0.95-1.02 \|  \|  \|  \|  \| \|  \|  \| Simple mode \| 19 \| 0.842 \| 0.99 \| 0.93-1.06 \|  \|  \|  \|  \| \|  \|  \| Weighted mode \| 19 \| 0.842 \| 0.99 \| 0.93-1.06 \|  \|  \|  \|  \| \|  \|  \|  \|  \|  \|  \|  \|  \|  \|  \|  \| \| Lipids \| Methylsuccinate levels \| MR Egger \| 18 \| 0.004 \| 1.05 \| 1.02-1.09 \| 11.42 \| 0.783 \|  \|  \| \|  \| GCST90200352 \| Inverse variance weighted \| 18 \| 0.007 \| 1.03 \| 1.01-1.05 \| 15.91 \| 0.53 \| -0.01 \| 0.05 \| \|  \|  \| Weighted median \| 18 \| 0.001 \| 1.04 \| 1.02-1.07 \|  \|  \|  \|  \| \|  \|  \| Simple mode \| 18 \| 0.636 \| 1.02 \| 0.95-1.08 \|  \|  \|  \|  \| \|  \|  \| Weighted mode \| 18 \| 0.003 \| 1.04 \| 1.02-1.07 \|  \|  \|  \|  \| \|  \|  \|  \|  \|  \|  \|  \|  \|  \|  \|  \| \| Lipids \| Cholesterol levels \| MR Egger \| 18 \| 0.167 \| 0.96 \| 0.91-1.01 \| 10.01 \| 0.866 \|  \|  \| \|  \| GCST90200368 \| Inverse variance weighted \| 18 \| 0.011 \| 0.96 \| 0.94-0.99 \| 10.06 \| 0.901 \| 0 \| 0.829 \| \|  \|  \| Weighted median \| 18 \| 0.034 \| 0.96 \| 0.92-1 \|  \|  \|  \|  \| \|  \|  \| Simple mode \| 18 \| 0.144 \| 0.95 \| 0.89-1.02 \|  \|  \|  \|  \| \|  \|  \| Weighted mode \| 18 \| 0.183 \| 0.95 \| 0.89-1.02 \|  \|  \|  \|  \| \|  \|  \|  \|  \|  \|  \|  \|  \|  \|  \|  \| \| Lipids \| 1-palmitoyl-2-arachidonoyl-gpc (16:0/20:4n6) levels \| MR Egger \| 28 \| 0.115 \| 1.02 \| 1-1.05 \| 46.92 \| 0.007 \|  \|  \| \|  \| GCST90200692 \| Inverse variance weighted \| 28 \| 0.016 \| 1.02 \| 1-1.04 \| 46.93 \| 0.01 \| 0 \| 0.955 \| \|  \|  \| Weighted median \| 28 \| 0.001 \| 1.03 \| 1.01-1.04 \|  \|  \|  \|  \| \|  \|  \| Simple mode \| 28 \| 0.524 \| 1.02 \| 0.96-1.08 \|  \|  \|  \|  \| \|  \|  \| Weighted mode \| 28 \| 0.005 \| 1.02 \| 1.01-1.04 \|  \|  \|  \|  \| \|  \|  \|  \|  \|  \|  \|  \|  \|  \|  \|  \| \| Amino acids \| N-acetylglutamate levels \| MR Egger \| 19 \| 0.977 \| 1.00 \| 0.94-1.07 \| 17.56 \| 0.417 \|  \|  \| \|  \| GCST90199637 \| Inverse variance weighted \| 19 \| 0.007 \| 0.96 \| 0.94-0.99 \| 18.99 \| 0.392 \| 0.00 \| 0.256 \| \|  \|  \| Weighted median \| 19 \| 0.250 \| 0.98 \| 0.94-1.02 \|  \|  \|  \|  \| \|  \|  \| Simple mode \| 19 \| 0.586 \| 0.98 \| 0.92-1.05 \|  \|  \|  \|  \| \|  \|  \| Weighted mode \| 19 \| 0.549 \| 0.98 \| 0.92-1.04 \|  \|  \|  \|  \| \|  \|  \|  \|  \|  \|  \|  \|  \|  \|  \|  \| \| Amino acids \| N-acetylhistidine levels \| MR Egger \| 20 \| 0.039 \| 0.98 \| 0.96-1 \| 18.02 \| 0.454 \|  \|  \| \|  \| GCST90199735 \| Inverse variance weighted \| 20 \| 0.024 \| 0.98 \| 0.97-1 \| 18.95 \| 0.460 \| 0.00 \| 0.349 \| \|  \|  \| Weighted median \| 20 \| 0.025 \| 0.98 \| 0.96-1 \|  \|  \|  \|  \| \|  \|  \| Simple mode \| 20 \| 0.841 \| 1.00 \| 0.96-1.04 \|  \|  \|  \|  \| \|  \|  \| Weighted mode \| 20 \| 0.038 \| 0.98 \| 0.97-1 \|  \|  \|  \|  \| \|  \|  \|  \|  \|  \|  \|  \|  \|  \|  \|  \| \| Amino acids \| N6-acetyllysine levels \| MR Egger \| 17 \| 0.031 \| 0.96 \| 0.92-0.99 \| 24.80 \| 0.053 \|  \|  \| \|  \| GCST90199826 \| Inverse variance weighted \| 17 \| 0.002 \| 0.96 \| 0.94-0.99 \| 25.08 \| 0.068 \| 0.00 \| 0.690 \| \|  \|  \| Weighted median \| 17 \| 0.002 \| 0.96 \| 0.94-0.99 \|  \|  \|  \|  \| \|  \|  \| Simple mode \| 17 \| 0.031 \| 0.94 \| 0.89-0.99 \|  \|  \|  \|  \| \|  \|  \| Weighted mode \| 17 \| 0.005 \| 0.96 \| 0.94-0.98 \|  \|  \|  \|  \| \|  \|  \|  \|  \|  \|  \|  \|  \|  \|  \|  \| \| Amino acids \| 6-oxopiperidine-2-carboxylate levels \| MR Egger \| 22 \| 0.075 \| 0.97 \| 0.93-1 \| 27.22 \| 0.129 \|  \|  \| \|  \| GCST90199949 \| Inverse variance weighted \| 22 \| 0.003 \| 0.97 \| 0.95-0.99 \| 27.22 \| 0.164 \| 0.00 \| 0.967 \| \|  \|  \| Weighted median \| 22 \| 0.009 \| 0.97 \| 0.94-0.99 \|  \|  \|  \|  \| \|  \|  \| Simple mode \| 22 \| 0.301 \| 0.97 \| 0.92-1.03 \|  \|  \|  \|  \| \|  \|  \| Weighted mode \| 22 \| 0.029 \| 0.97 \| 0.94-0.99 \|  \|  \|  \|  \| \|  \|  \|  \|  \|  \|  \|  \|  \|  \|  \|  \| \| Amino acids \| 5-oxoproline levels \| MR Egger \| 22 \| 0.014 \| 0.97 \| 0.95-0.99 \| 18.29 \| 0.568 \|  \|  \| \|  \| GCST90200280 \| Inverse variance weighted \| 22 \| 0.013 \| 0.98 \| 0.96-1 \| 20.07 \| 0.517 \| 0.00 \| 0.198 \| \|  \|  \| Weighted median \| 22 \| 0.010 \| 0.98 \| 0.96-0.99 \|  \|  \|  \|  \| \|  \|  \| Simple mode \| 22 \| 0.251 \| 0.97 \| 0.92-1.02 \|  \|  \|  \|  \| \|  \|  \| Weighted mode \| 22 \| 0.018 \| 0.97 \| 0.96-0.99 \|  \|  \|  \|  \| \|  \|  \|  \|  \|  \|  \|  \|  \|  \|  \|  \| \| Amino acids \| Gamma-glutamyltyrosine levels \| MR Egger \| 25 \| 0.492 \| 1.02 \| 0.97-1.07 \| 13.91 \| 0.930 \|  \|  \| \|  \| GCST90200295 \| Inverse variance weighted \| 25 \| 0.040 \| 1.02 \| 1-1.05 \| 14.00 \| 0.947 \| 0.00 \| 0.761 \| \|  \|  \| Weighted median \| 25 \| 0.126 \| 1.03 \| 0.99-1.06 \|  \|  \|  \|  \| \|  \|  \| Simple mode \| 25 \| 0.392 \| 1.03 \| 0.97-1.09 \|  \|  \|  \|  \| \|  \|  \| Weighted mode \| 25 \| 0.300 \| 1.03 \| 0.98-1.08 \|  \|  \|  \|  \| \|  \|  \|  \|  \|  \|  \|  \|  \|  \|  \|  \| \| Amino acids \| Glutamine levels \| MR Egger \| 24 \| 0.045 \| 1.05 \| 1-1.09 \| 22.3 \| 0.442 \|  \|  \| \|  \| GCST90200419 \| Inverse variance weighted \| 24 \| 0.005 \| 1.03 \| 1.01-1.05 \| 23.11 \| 0.454 \| 0 \| 0.379 \| \|  \|  \| Weighted median \| 24 \| 0.006 \| 1.04 \| 1.01-1.08 \|  \|  \|  \|  \| \|  \|  \| Simple mode \| 24 \| 0.131 \| 1.04 \| 0.99-1.09 \|  \|  \|  \|  \| \|  \|  \| Weighted mode \| 24 \| 0.018 \| 1.05 \| 1.01-1.08 \|  \|  \|  \|  \| \|  \|  \|  \|  \|  \|  \|  \|  \|  \|  \|  \| \| Keto acids \| 2-oxoarginine levels \| MR Egger \| 20 \| 0.699 \| 0.98 \| 0.91-1.07 \| 21.38 \| 0.261 \|  \|  \| \|  \| GCST90199903 \| Inverse variance weighted \| 20 \| 0.015 \| 0.97 \| 0.94-0.99 \| 21.60 \| 0.305 \| 0.00 \| 0.668 \| \|  \|  \| Weighted median \| 20 \| 0.258 \| 0.98 \| 0.94-1.02 \|  \|  \|  \|  \| \|  \|  \| Simple mode \| 20 \| 0.101 \| 0.93 \| 0.86-1.01 \|  \|  \|  \|  \| \|  \|  \| Weighted mode \| 20 \| 0.126 \| 0.95 \| 0.89-1.01 \|  \|  \|  \|  \| \|  \|  \|  \|  \|  \|  \|  \|  \|  \|  \|  \| \| Keto acids \| Alpha-ketobutyrate levels \| MR Egger \| 14 \| 0.224 \| 0.96 \| 0.89-1.02 \| 7.51 \| 0.822 \|  \|  \| \|  \| GCST90200438 \| Inverse variance weighted \| 14 \| 0.034 \| 0.97 \| 0.94-1 \| 7.62 \| 0.867 \| 0 \| 0.746 \| \|  \|  \| Weighted median \| 14 \| 0.353 \| 0.98 \| 0.94-1.02 \|  \|  \|  \|  \| \|  \|  \| Simple mode \| 14 \| 0.669 \| 0.98 \| 0.92-1.06 \|  \|  \|  \|  \| \|  \|  \| Weighted mode \| 14 \| 0.687 \| 0.98 \| 0.92-1.06 \|  \|  \|  \|  \| \|  \|  \|  \|  \|  \|  \|  \|  \|  \|  \|  \| \| Quinic acid \| Quinate levels \| MR Egger \| 16 \| 0.359 \| 0.97 \| 0.92-1.03 \| 12.45 \| 0.570 \|  \|  \| \|  \| GCST90199645 \| Inverse variance weighted \| 16 \| 0.007 \| 0.96 \| 0.93-0.99 \| 12.67 \| 0.628 \| 0.00 \| 0.649 \| \|  \|  \| Weighted median \| 16 \| 0.034 \| 0.96 \| 0.92-1 \|  \|  \|  \|  \| \|  \|  \| Simple mode \| 16 \| 0.220 \| 0.95 \| 0.89-1.03 \|  \|  \|  \|  \| \|  \|  \| Weighted mode \| 16 \| 0.207 \| 0.95 \| 0.89-1.02 \|  \|  \|  \|  \| \|  \|  \|  \|  \|  \|  \|  \|  \|  \|  \|  \| \| Carboxylic acids \| N-acetyl-beta-alanine levels \| MR Egger \| 23 \| 0.059 \| 1.04 \| 1-1.09 \| 14.03 \| 0.868 \|  \|  \| \|  \| GCST90199866 \| Inverse variance weighted \| 23 \| 0.003 \| 1.03 \| 1.01-1.06 \| 14.31 \| 0.890 \| 0.00 \| 0.606 \| \|  \|  \| Weighted median \| 23 \| 0.007 \| 1.05 \| 1.01-1.08 \|  \|  \|  \|  \| \|  \|  \| Simple mode \| 23 \| 0.256 \| 1.04 \| 0.98-1.1 \|  \|  \|  \|  \| \|  \|  \| Weighted mode \| 23 \| 0.039 \| 1.05 \| 1-1.09 \|  \|  \|  \|  \| \|  \|  \|  \|  \|  \|  \|  \|  \|  \|  \|  \| \| Naphthalenes \| 2-naphthol sulfate levels \| MR Egger \| 20 \| 0.061 \| 0.94 \| 0.89-1 \| 14.18 \| 0.717 \|  \|  \| \|  \| GCST90200213 \| Inverse variance weighted \| 20 \| 0.045 \| 0.97 \| 0.95-1 \| 15.65 \| 0.680 \| 0.00 \| 0.241 \| \|  \|  \| Weighted median \| 20 \| 0.104 \| 0.97 \| 0.93-1.01 \|  \|  \|  \|  \| \|  \|  \| Simple mode \| 20 \| 0.347 \| 0.96 \| 0.9-1.04 \|  \|  \|  \|  \| \|  \|  \| Weighted mode \| 20 \| 0.392 \| 0.97 \| 0.91-1.04 \|  \|  \|  \|  \| \|  \|  \|  \|  \|  \|  \|  \|  \|  \|  \|  \| \| Purine nucleotides \| Adenosine 5'-diphosphate (ADP) levels \| MR Egger \| 22 \| 0.106 \| 0.96 \| 0.91-1.01 \| 22.69 \| 0.304 \|  \|  \| \|  \| GCST90200355 \| Inverse variance weighted \| 22 \| 0.007 \| 0.97 \| 0.95-0.99 \| 23.26 \| 0.33 \| 0 \| 0.487 \| \|  \|  \| Weighted median \| 22 \| 0.018 \| 0.97 \| 0.94-0.99 \|  \|  \|  \|  \| \|  \|  \| Simple mode \| 22 \| 0.171 \| 0.96 \| 0.91-1.02 \|  \|  \|  \|  \| \|  \|  \| Weighted mode \| 22 \| 0.137 \| 0.96 \| 0.91-1.01 \|  \|  \|  \|  \| \|  \|  \|  \|  \|  \|  \|  \|  \|  \|  \|  \| \| Ratio \| Adenosine 5'-diphosphate (ADP) to Adenosine 5'-monophosphate (AMP) ratio \| MR Egger \| 23 \| 0.411 \| 0.98 \| 0.94-1.03 \| 14.22 \| 0.86 \|  \|  \| \|  \| GCST90200728 \| Inverse variance weighted \| 23 \| 0.015 \| 0.98 \| 0.96-1 \| 14.24 \| 0.893 \| 0 \| 0.888 \| \|  \|  \| Weighted median \| 23 \| 0.111 \| 0.98 \| 0.96-1 \|  \|  \|  \|  \| \|  \|  \| Simple mode \| 23 \| 0.576 \| 0.99 \| 0.94-1.03 \|  \|  \|  \|  \| \|  \|  \| Weighted mode \| 23 \| 0.560 \| 0.99 \| 0.94-1.03 \|  \|  \|  \|  \| \|  \|  \|  \|  \|  \|  \|  \|  \|  \|  \|  \| \| Ratio \| Arachidonate (20:4n6) to oleate to vaccenate (18:1) ratio \| MR Egger \| 18 \| 0.071 \| 1.03 \| 1-1.07 \| 25.37 \| 0.063 \|  \|  \| \|  \| GCST90200740 \| Inverse variance weighted \| 18 \| 0.004 \| 1.03 \| 1.01-1.05 \| 25.47 \| 0.085 \| 0 \| 0.811 \| \|  \|  \| Weighted median \| 18 \| 0.001 \| 1.04 \| 1.02-1.06 \|  \|  \|  \|  \| \|  \|  \| Simple mode \| 18 \| 0.897 \| 1 \| 0.95-1.06 \|  \|  \|  \|  \| \|  \|  \| Weighted mode \| 18 \| 0.005 \| 1.03 \| 1.01-1.05 \|  \|  \|  \|  \| \|  \|  \|  \|  \|  \|  \|  \|  \|  \|  \|  \| \| Ratio \| Oleoyl-linoleoyl-glycerol (18:1 to 18:2) [2] to linoleoyl-arachidonoyl-glycerol (18:2 to 20:4) [2] ratio \| MR Egger \| 20 \| 0.003 \| 0.96 \| 0.93-0.98 \| 20.07 \| 0.329 \|  \|  \| \|  \| GCST90200795 \| Inverse variance weighted \| 20 \| 0.002 \| 0.97 \| 0.96-0.99 \| 23.31 \| 0.224 \| 0 \| 0.105 \| \|  \|  \| Weighted median \| 20 \| 0.001 \| 0.97 \| 0.95-0.99 \|  \|  \|  \|  \| \|  \|  \| Simple mode \| 20 \| 0.845 \| 0.99 \| 0.95-1.05 \|  \|  \|  \|  \| \|  \|  \| Weighted mode \| 20 \| 0.003 \| 0.97 \| 0.95-0.99 \|  \|  \|  \|  \| \|  \|  \|  \|  \|  \|  \|  \|  \|  \|  \|  \| \| Ratio \| Adenosine 5'-monophosphate (AMP) to glutamine ratio \| MR Egger \| 16 \| 0.056 \| 1.07 \| 1-1.14 \| 8.18 \| 0.88 \|  \|  \| \|  \| GCST90200848 \| Inverse variance weighted \| 16 \| 0.041 \| 1.03 \| 1-1.06 \| 9.73 \| 0.837 \| 0 \| 0.234 \| \|  \|  \| Weighted median \| 16 \| 0.086 \| 1.04 \| 0.99-1.08 \|  \|  \|  \|  \| \|  \|  \| Simple mode \| 16 \| 0.100 \| 1.06 \| 0.99-1.14 \|  \|  \|  \|  \| \|  \|  \| Weighted mode \| 16 \| 0.107 \| 1.06 \| 0.99-1.13 \|  \|  \|  \|  \| \|  \|  \|  \|  \|  \|  \|  \|  \|  \|  \|  \| \| Ratio \| Adenosine 5'-monophosphate (AMP) to asparagine ratio \| MR Egger \| 20 \| 0.085 \| 1.06 \| 1-1.13 \| 9.33 \| 0.952 \|  \|  \| \|  \| GCST90200859 \| Inverse variance weighted \| 20 \| 0.004 \| 1.04 \| 1.01-1.07 \| 9.83 \| 0.957 \| 0 \| 0.49 \| \|  \|  \| Weighted median \| 20 \| 0.048 \| 1.04 \| 1-1.07 \|  \|  \|  \|  \| \|  \|  \| Simple mode \| 20 \| 0.369 \| 1.03 \| 0.97-1.1 \|  \|  \|  \|  \| \|  \|  \| Weighted mode \| 20 \| 0.324 \| 1.03 \| 0.97-1.1 \|  \|  \|  \|  \| \|  \|  \|  \|  \|  \|  \|  \|  \|  \|  \|  \| \| Ratio \| Adenosine 5'-monophosphate (AMP) to serine ratio \| MR Egger \| 15 \| 0.350 \| 1.04 \| 0.96-1.11 \| 11.82 \| 0.543 \|  \|  \| \|  \| GCST90200860 \| Inverse variance weighted \| 15 \| 0.046 \| 1.03 \| 1-1.06 \| 11.85 \| 0.619 \| 0 \| 0.867 \| \|  \|  \| Weighted median \| 15 \| 0.112 \| 1.03 \| 0.99-1.08 \|  \|  \|  \|  \| \|  \|  \| Simple mode \| 15 \| 0.123 \| 1.06 \| 0.99-1.14 \|  \|  \|  \|  \| \|  \|  \| Weighted mode \| 15 \| 0.142 \| 1.06 \| 0.99-1.14 \|  \|  \|  \|  \| \|  \|  \|  \|  \|  \|  \|  \|  \|  \|  \|  \| \| Ratio \| Phosphate to serine ratio \| MR Egger \| 21 \| 0.160 \| 1.05 \| 0.98-1.11 \| 14.85 \| 0.732 \|  \|  \| \|  \| GCST90200863 \| Inverse variance weighted \| 21 \| 0.041 \| 1.02 \| 1-1.05 \| 15.41 \| 0.753 \| 0 \| 0.464 \| \|  \|  \| Weighted median \| 21 \| 0.033 \| 1.04 \| 1-1.07 \|  \|  \|  \|  \| \|  \|  \| Simple mode \| 21 \| 0.310 \| 1.03 \| 0.97-1.1 \|  \|  \|  \|  \| \|  \|  \| Weighted mode \| 21 \| 0.166 \| 1.03 \| 0.99-1.08 \|  \|  \|  \|  \| \|  \|  \|  \|  \|  \|  \|  \|  \|  \|  \|  \| \| Ratio \| Methionine to phosphate ratio \| MR Egger \| 14 \| 0.364 \| 0.97 \| 0.9-1.04 \| 11.95 \| 0.449 \|  \|  \| \|  \| GCST90200864 \| Inverse variance weighted \| 14 \| 0.002 \| 0.95 \| 0.92-0.98 \| 12.27 \| 0.506 \| 0 \| 0.586 \| \|  \|  \| Weighted median \| 14 \| 0.107 \| 0.96 \| 0.92-1.01 \|  \|  \|  \|  \| \|  \|  \| Simple mode \| 14 \| 0.635 \| 0.98 \| 0.9-1.06 \|  \|  \|  \|  \| \|  \|  \| Weighted mode \| 14 \| 0.609 \| 0.98 \| 0.91-1.06 \|  \|  \|  \|  \| \|  \|  \|  \|  \|  \|  \|  \|  \|  \|  \|  \| \| Ratio \| Adenosine 5'-monophosphate (AMP) to isoleucine ratio \| MR Egger \| 19 \| 0.041 \| 1.09 \| 1.01-1.17 \| 13.9 \| 0.674 \|  \|  \| \|  \| GCST90200867 \| Inverse variance weighted \| 19 \| 0.039 \| 1.03 \| 1-1.06 \| 16.3 \| 0.572 \| -0.01 \| 0.14 \| \|  \|  \| Weighted median \| 19 \| 0.264 \| 1.02 \| 0.98-1.06 \|  \|  \|  \|  \| \|  \|  \| Simple mode \| 19 \| 0.702 \| 1.01 \| 0.95-1.09 \|  \|  \|  \|  \| \|  \|  \| Weighted mode \| 19 \| 0.710 \| 1.01 \| 0.95-1.08 \|  \|  \|  \|  \| \|  \|  \|  \|  \|  \|  \|  \|  \|  \|  \|  \| \| Ratio \| Phenylpyruvate to citrate ratio \| MR Egger \| 27 \| 0.118 \| 0.96 \| 0.91-1.01 \| 17.16 \| 0.876 \|  \|  \| \|  \| GCST90200885 \| Inverse variance weighted \| 27 \| 0.017 \| 0.97 \| 0.95-1 \| 17.61 \| 0.889 \| 0 \| 0.506 \| \|  \|  \| Weighted median \| 27 \| 0.020 \| 0.96 \| 0.93-0.99 \|  \|  \|  \|  \| \|  \|  \| Simple mode \| 27 \| 0.146 \| 0.96 \| 0.9-1.01 \|  \|  \|  \|  \| \|  \|  \| Weighted mode \| 27 \| 0.130 \| 0.96 \| 0.9-1.01 \|  \|  \|  \|  \| \|  \|  \|  \|  \|  \|  \|  \|  \|  \|  \|  \| \| Ratio \| Cortisol to taurocholate ratio \| MR Egger \| 20 \| 0.364 \| 0.98 \| 0.93-1.03 \| 20.07 \| 0.329 \|  \|  \| \|  \| GCST90200890 \| Inverse variance weighted \| 20 \| 0.040 \| 0.97 \| 0.95-1 \| 20.09 \| 0.389 \| 0 \| 0.896 \| \|  \|  \| Weighted median \| 20 \| 0.070 \| 0.97 \| 0.93-1 \|  \|  \|  \|  \| \|  \|  \| Simple mode \| 20 \| 0.248 \| 0.96 \| 0.9-1.02 \|  \|  \|  \|  \| \|  \|  \| Weighted mode \| 20 \| 0.279 \| 0.96 \| 0.91-1.03 \|  \|  \|  \|  \| \|  \|  \|  \|  \|  \|  \|  \|  \|  \|  \|  \| \| Ratio \| Alpha-ketoglutarate to proline ratio \| MR Egger \| 21 \| 0.313 \| 0.97 \| 0.91-1.03 \| 18.23 \| 0.507 \|  \|  \| \|  \| GCST90200933 \| Inverse variance weighted \| 21 \| 0.050 \| 0.98 \| 0.95-1 \| 18.29 \| 0.568 \| 0 \| 0.809 \| \|  \|  \| Weighted median \| 21 \| 0.490 \| 0.99 \| 0.95-1.02 \|  \|  \|  \|  \| \|  \|  \| Simple mode \| 21 \| 0.853 \| 0.99 \| 0.93-1.06 \|  \|  \|  \|  \| \|  \|  \| Weighted mode \| 21 \| 0.830 \| 0.99 \| 0.94-1.06 \|  \|  \|  \|  \| \|  \|  \|  \|  \|  \|  \|  \|  \|  \|  \|  \| \| Ratio \| Phosphate to 5-oxoproline ratio \| MR Egger \| 24 \| 0.194 \| 1.02 \| 0.99-1.05 \| 29.15 \| 0.141 \|  \|  \| \|  \| GCST90200968 \| Inverse variance weighted \| 24 \| 0.010 \| 1.03 \| 1.01-1.05 \| 29.52 \| 0.164 \| 0 \| 0.602 \| \|  \|  \| Weighted median \| 24 \| 0.012 \| 1.03 \| 1.01-1.05 \|  \|  \|  \|  \| \|  \|  \| Simple mode \| 24 \| 0.299 \| 1.03 \| 0.97-1.09 \|  \|  \|  \|  \| \|  \|  \| Weighted mode \| 24 \| 0.026 \| 1.03 \| 1.01-1.05 \|  \|  \|  \|  \| \|  \|  \|  \|  \|  \|  \|  \|  \|  \|  \|  \| \| Ratio \| Arachidonate (20:4n6) to linoleate (18:2n6) ratio \| MR Egger \| 20 \| 0.041 \| 1.04 \| 1-1.07 \| 24.95 \| 0.126 \|  \|  \| \|  \| GCST90200979 \| Inverse variance weighted \| 20 \| 0.001 \| 1.03 \| 1.01-1.05 \| 25.08 \| 0.158 \| 0 \| 0.761 \| \|  \|  \| Weighted median \| 20 \| 0.000 \| 1.04 \| 1.02-1.06 \|  \|  \|  \|  \| \|  \|  \| Simple mode \| 20 \| 0.275 \| 1.03 \| 0.98-1.09 \|  \|  \|  \|  \| \|  \|  \| Weighted mode \| 20 \| 0.002 \| 1.04 \| 1.02-1.06 \|  \|  \|  \|  \| \|  \|  \|  \|  \|  \|  \|  \|  \|  \|  \|  \| \| Ratio \| Benzoate to linoleoyl-arachidonoyl-glycerol (18:2 to 20:4) [2] ratio \| MR Egger \| 19 \| 0.067 \| 0.96 \| 0.91-1 \| 23.01 \| 0.149 \|  \|  \| \|  \| GCST90200990 \| Inverse variance weighted \| 19 \| 0.011 \| 0.97 \| 0.95-0.99 \| 23.96 \| 0.156 \| 0 \| 0.412 \| \|  \|  \| Weighted median \| 19 \| 0.001 \| 0.95 \| 0.93-0.98 \|  \|  \|  \|  \| \|  \|  \| Simple mode \| 19 \| 0.162 \| 0.96 \| 0.9-1.01 \|  \|  \|  \|  \| \|  \|  \| Weighted mode \| 19 \| 0.006 \| 0.95 \| 0.92-0.98 \|  \|  \|  \|  \| \|  \|  \|  \|  \|  \|  \|  \|  \|  \|  \|  \| \| Ratio \| Threonine to pyruvate ratio \| MR Egger \| 24 \| 0.459 \| 1.02 \| 0.97-1.08 \| 22.88 \| 0.408 \|  \|  \| \|  \| GCST90201009 \| Inverse variance weighted \| 24 \| 0.005 \| 1.03 \| 1.01-1.06 \| 23.12 \| 0.454 \| 0 \| 0.639 \| \|  \|  \| Weighted median \| 24 \| 0.091 \| 1.03 \| 1-1.06 \|  \|  \|  \|  \| \|  \|  \| Simple mode \| 24 \| 0.451 \| 1.03 \| 0.96-1.1 \|  \|  \|  \|  \| \|  \|  \| Weighted mode \| 24 \| 0.388 \| 1.03 \| 0.97-1.08 \|  \|  \|  \|  \| \|  \|  \|  \|  \|  \|  \|  \|  \|  \|  \|  \| \| Ratio \| Androsterone glucuronide to etiocholanolone glucuronide ratio \| MR Egger \| 26 \| 0.045 \| 1.04 \| 1-1.08 \| 52.33 \| 0.001 \|  \|  \| \|  \| GCST90201013 \| Inverse variance weighted \| 26 \| 0.028 \| 1.03 \| 1-1.05 \| 53.99 \| 0.001 \| 0 \| 0.391 \| \|  \|  \| Weighted median \| 26 \| 0.002 \| 1.04 \| 1.01-1.06 \|  \|  \|  \|  \| \|  \|  \| Simple mode \| 26 \| 0.176 \| 1.04 \| 0.99-1.09 \|  \|  \|  \|  \| \|  \|  \| Weighted mode \| 26 \| 0.005 \| 1.04 \| 1.01-1.06 \|  \|  \|  \|  \| \|  \|  \|  \|  \|  \|  \|  \|  \|  \|  \|  \| \| Unknown \| X-11470 levels \| MR Egger \| 30 \| 0.177 \| 1.02 \| 0.99-1.05 \| 41.08 \| 0.053 \|  \|  \| \|  \| GCST90200470 \| Inverse variance weighted \| 30 \| 0.019 \| 1.02 \| 1-1.04 \| 41.12 \| 0.067 \| 0 \| 0.867 \| \|  \|  \| Weighted median \| 30 \| 0.288 \| 1.02 \| 0.99-1.04 \|  \|  \|  \|  \| \|  \|  \| Simple mode \| 30 \| 0.088 \| 1.05 \| 0.99-1.1 \|  \|  \|  \|  \| \|  \|  \| Weighted mode \| 30 \| 0.523 \| 1.01 \| 0.98-1.05 \|  \|  \|  \|  \| \|  \|  \|  \|  \|  \|  \|  \|  \|  \|  \|  \| \| Unknown \| X-11444 levels \| MR Egger \| 28 \| 0.013 \| 1.04 \| 1.01-1.07 \| 32.99 \| 0.162 \|  \|  \| \|  \| GCST90200474 \| Inverse variance weighted \| 28 \| 0.002 \| 1.03 \| 1.01-1.05 \| 34.08 \| 0.164 \| 0 \| 0.361 \| \|  \|  \| Weighted median \| 28 \| 0.089 \| 1.02 \| 1-1.05 \|  \|  \|  \|  \| \|  \|  \| Simple mode \| 28 \| 0.358 \| 1.03 \| 0.97-1.09 \|  \|  \|  \|  \| \|  \|  \| Weighted mode \| 28 \| 0.028 \| 1.04 \| 1.01-1.07 \|  \|  \|  \|  \| \|  \|  \|  \|  \|  \|  \|  \|  \|  \|  \|  \| \| Unknown \| X-12410 levels \| MR Egger \| 21 \| 0.201 \| 0.97 \| 0.92-1.02 \| 28.18 \| 0.08 \|  \|  \| \|  \| GCST90200480 \| Inverse variance weighted \| 21 \| 0.013 \| 0.97 \| 0.95-0.99 \| 28.19 \| 0.105 \| 0 \| 0.919 \| \|  \|  \| Weighted median \| 21 \| 0.022 \| 0.96 \| 0.93-0.99 \|  \|  \|  \|  \| \|  \|  \| Simple mode \| 21 \| 0.888 \| 1 \| 0.94-1.06 \|  \|  \|  \|  \| \|  \|  \| Weighted mode \| 21 \| 0.082 \| 0.97 \| 0.93-1 \|  \|  \|  \|  \| \|  \|  \|  \|  \|  \|  \|  \|  \|  \|  \|  \| \| Unknown \| X-12740 levels \| MR Egger \| 12 \| 0.394 \| 1.04 \| 0.96-1.12 \| 9.6 \| 0.476 \|  \|  \| \|  \| GCST90200497 \| Inverse variance weighted \| 12 \| 0.001 \| 1.05 \| 1.02-1.08 \| 9.71 \| 0.556 \| 0 \| 0.74 \| \|  \|  \| Weighted median \| 12 \| 0.021 \| 1.05 \| 1.01-1.1 \|  \|  \|  \|  \| \|  \|  \| Simple mode \| 12 \| 0.576 \| 1.02 \| 0.95-1.1 \|  \|  \|  \|  \| \|  \|  \| Weighted mode \| 12 \| 0.696 \| 1.01 \| 0.95-1.09 \|  \|  \|  \|  \| \|  \|  \|  \|  \|  \|  \|  \|  \|  \|  \|  \| \| Unknown \| X-13728 levels \| MR Egger \| 14 \| 0.459 \| 1.02 \| 0.96-1.09 \| 9.63 \| 0.649 \|  \|  \| \|  \| GCST90200522 \| Inverse variance weighted \| 14 \| 0.034 \| 1.03 \| 1-1.07 \| 9.71 \| 0.717 \| 0 \| 0.778 \| \|  \|  \| Weighted median \| 14 \| 0.062 \| 1.04 \| 1-1.08 \|  \|  \|  \|  \| \|  \|  \| Simple mode \| 14 \| 0.554 \| 1.02 \| 0.95-1.09 \|  \|  \|  \|  \| \|  \|  \| Weighted mode \| 14 \| 0.386 \| 1.03 \| 0.96-1.1 \|  \|  \|  \|  \| \|  \|  \|  \|  \|  \|  \|  \|  \|  \|  \|  \| \| Unknown \| X-18901 levels \| MR Egger \| 27 \| 0.111 \| 1.04 \| 0.99-1.08 \| 13.77 \| 0.966 \|  \|  \| \|  \| GCST90200559 \| Inverse variance weighted \| 27 \| 0.003 \| 1.04 \| 1.01-1.06 \| 13.77 \| 0.976 \| 0 \| 0.979 \| \|  \|  \| Weighted median \| 27 \| 0.051 \| 1.03 \| 1-1.06 \|  \|  \|  \|  \| \|  \|  \| Simple mode \| 27 \| 0.370 \| 1.03 \| 0.97-1.09 \|  \|  \|  \|  \| \|  \|  \| Weighted mode \| 27 \| 0.363 \| 1.03 \| 0.97-1.09 \|  \|  \|  \|  \| \|  \|  \|  \|  \|  \|  \|  \|  \|  \|  \|  \| \| Unknown \| X-18935 levels \| MR Egger \| 16 \| 0.939 \| 1 \| 0.94-1.07 \| 11.07 \| 0.681 \|  \|  \| \|  \| GCST90200573 \| Inverse variance weighted \| 16 \| 0.030 \| 1.03 \| 1-1.05 \| 11.6 \| 0.709 \| 0 \| 0.477 \| \|  \|  \| Weighted median \| 16 \| 0.370 \| 1.02 \| 0.98-1.05 \|  \|  \|  \|  \| \|  \|  \| Simple mode \| 16 \| 0.878 \| 1 \| 0.95-1.07 \|  \|  \|  \|  \| \|  \|  \| Weighted mode \| 16 \| 0.795 \| 1.01 \| 0.95-1.06 \|  \|  \|  \|  \| \|  \|  \|  \|  \|  \|  \|  \|  \|  \|  \|  \| \| Unknown \| X-21283 levels \| MR Egger \| 15 \| 0.046 \| 0.97 \| 0.95-1 \| 9.68 \| 0.72 \|  \|  \| \|  \| GCST90200575 \| Inverse variance weighted \| 15 \| 0.015 \| 0.98 \| 0.96-1 \| 10.19 \| 0.748 \| 0 \| 0.488 \| \|  \|  \| Weighted median \| 15 \| 0.013 \| 0.98 \| 0.96-0.99 \|  \|  \|  \|  \| \|  \|  \| Simple mode \| 15 \| 0.767 \| 0.99 \| 0.94-1.04 \|  \|  \|  \|  \| \|  \|  \| Weighted mode \| 15 \| 0.021 \| 0.97 \| 0.96-0.99 \|  \|  \|  \|  \| \|  \|  \|  \|  \|  \|  \|  \|  \|  \|  \|  \| \| Unknown \| X-24556 levels \| MR Egger \| 24 \| 0.800 \| 0.99 \| 0.95-1.04 \| 25.37 \| 0.28 \|  \|  \| \|  \| GCST90200628 \| Inverse variance weighted \| 24 \| 0.003 \| 0.97 \| 0.95-0.99 \| 26.86 \| 0.262 \| 0 \| 0.269 \| \|  \|  \| Weighted median \| 24 \| 0.077 \| 0.97 \| 0.94-1 \|  \|  \|  \|  \| \|  \|  \| Simple mode \| 24 \| 0.164 \| 0.96 \| 0.91-1.01 \|  \|  \|  \|  \| \|  \|  \| Weighted mode \| 24 \| 0.134 \| 0.97 \| 0.94-1.01 \|  \|  \|  \|  \| \|  \|  \|  \|  \|  \|  \|  \|  \|  \|  \|  \| \| Unknown \| X-24307 levels \| MR Egger \| 13 \| 0.509 \| 1.03 \| 0.95-1.12 \| 8.14 \| 0.701 \|  \|  \| \|  \| GCST90200632 \| Inverse variance weighted \| 13 \| 0.027 \| 1.04 \| 1-1.07 \| 8.18 \| 0.771 \| 0 \| 0.837 \| \|  \|  \| Weighted median \| 13 \| 0.443 \| 1.02 \| 0.98-1.06 \|  \|  \|  \|  \| \|  \|  \| Simple mode \| 13 \| 0.799 \| 1.01 \| 0.94-1.08 \|  \|  \|  \|  \| \|  \|  \| Weighted mode \| 13 \| 0.784 \| 1.01 \| 0.94-1.08 \|  \|  \|  \|  \| \|  \|  \|  \|  \|  \|  \|  \|  \|  \|  \|  \| \| Unknown \| X-24951 levels \| MR Egger \| 19 \| 0.151 \| 1.06 \| 0.98-1.15 \| 25.65 \| 0.081 \|  \|  \| \|  \| GCST90200643 \| Inverse variance weighted \| 19 \| 0.006 \| 1.05 \| 1.01-1.08 \| 25.82 \| 0.104 \| 0 \| 0.743 \| \|  \|  \| Weighted median \| 19 \| 0.062 \| 1.04 \| 1-1.09 \|  \|  \|  \|  \| \|  \|  \| Simple mode \| 19 \| 0.577 \| 1.03 \| 0.94-1.12 \|  \|  \|  \|  \| \|  \|  \| Weighted mode \| 19 \| 0.630 \| 1.02 \| 0.94-1.12 \|  \|  \|  \|  \| \|  \|  \|  \|  \|  \|  \|  \|  \|  \|  \|  \| \| Unknown \| X-24565 levels \| MR Egger \| 28 \| 0.177 \| 0.98 \| 0.95-1.01 \| 18.39 \| 0.861 \|  \|  \| \|  \| GCST90200645 \| Inverse variance weighted \| 28 \| 0.006 \| 0.98 \| 0.96-0.99 \| 18.39 \| 0.891 \| 0 \| 0.98 \| \|  \|  \| Weighted median \| 28 \| 0.063 \| 0.98 \| 0.96-1 \|  \|  \|  \|  \| \|  \|  \| Simple mode \| 28 \| 0.372 \| 0.98 \| 0.94-1.02 \|  \|  \|  \|  \| \|  \|  \| Weighted mode \| 28 \| 0.347 \| 0.98 \| 0.94-1.02 \|  \|  \|  \|  \| \|  \|  \|  \|  \|  \|  \|  \|  \|  \|  \|  \| \| Unknown \| X-25422 levels \| MR Egger \| 20 \| 0.105 \| 1.05 \| 0.99-1.11 \| 22.61 \| 0.206 \|  \|  \| \|  \| GCST90200661 \| Inverse variance weighted \| 20 \| 0.001 \| 1.04 \| 1.02-1.07 \| 22.72 \| 0.25 \| 0 \| 0.777 \| \|  \|  \| Weighted median \| 20 \| 0.016 \| 1.04 \| 1.01-1.07 \|  \|  \|  \|  \| \|  \|  \| Simple mode \| 20 \| 0.191 \| 1.04 \| 0.98-1.1 \|  \|  \|  \|  \| \|  \|  \| Weighted mode \| 20 \| 0.029 \| 1.04 \| 1.01-1.07 \|  \|  \|  \|  \| |

3.Supplementary Table 3: Leave-One-Out plots of 96 metabolites

1.GCST90199637


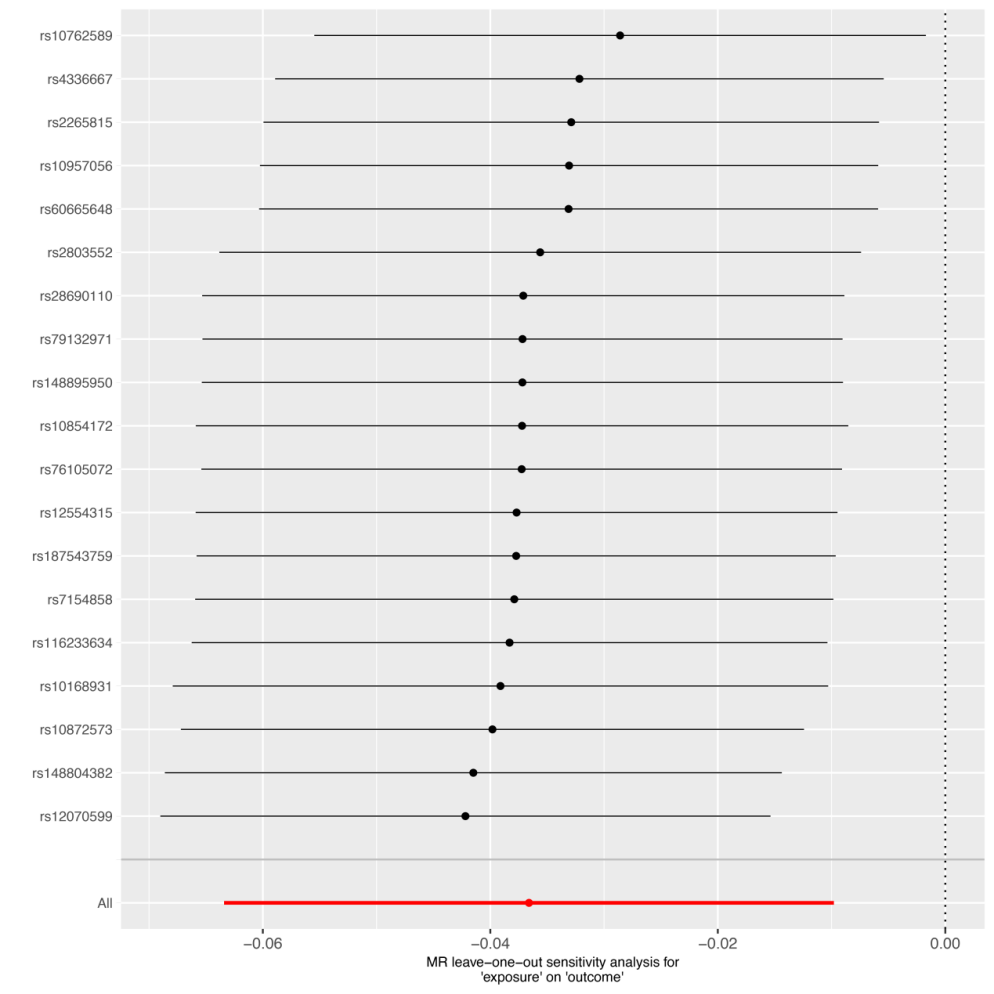


2.GCST90199645


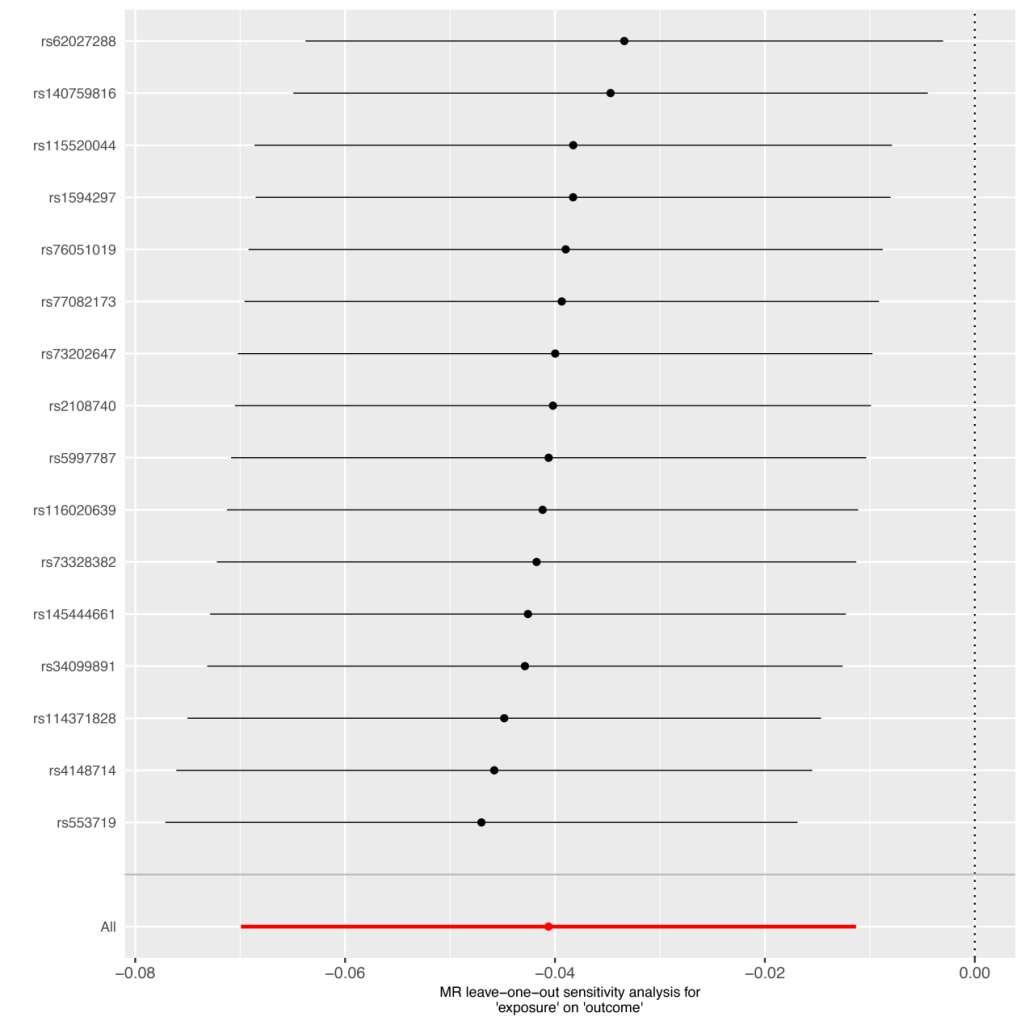


3.GCST90199649


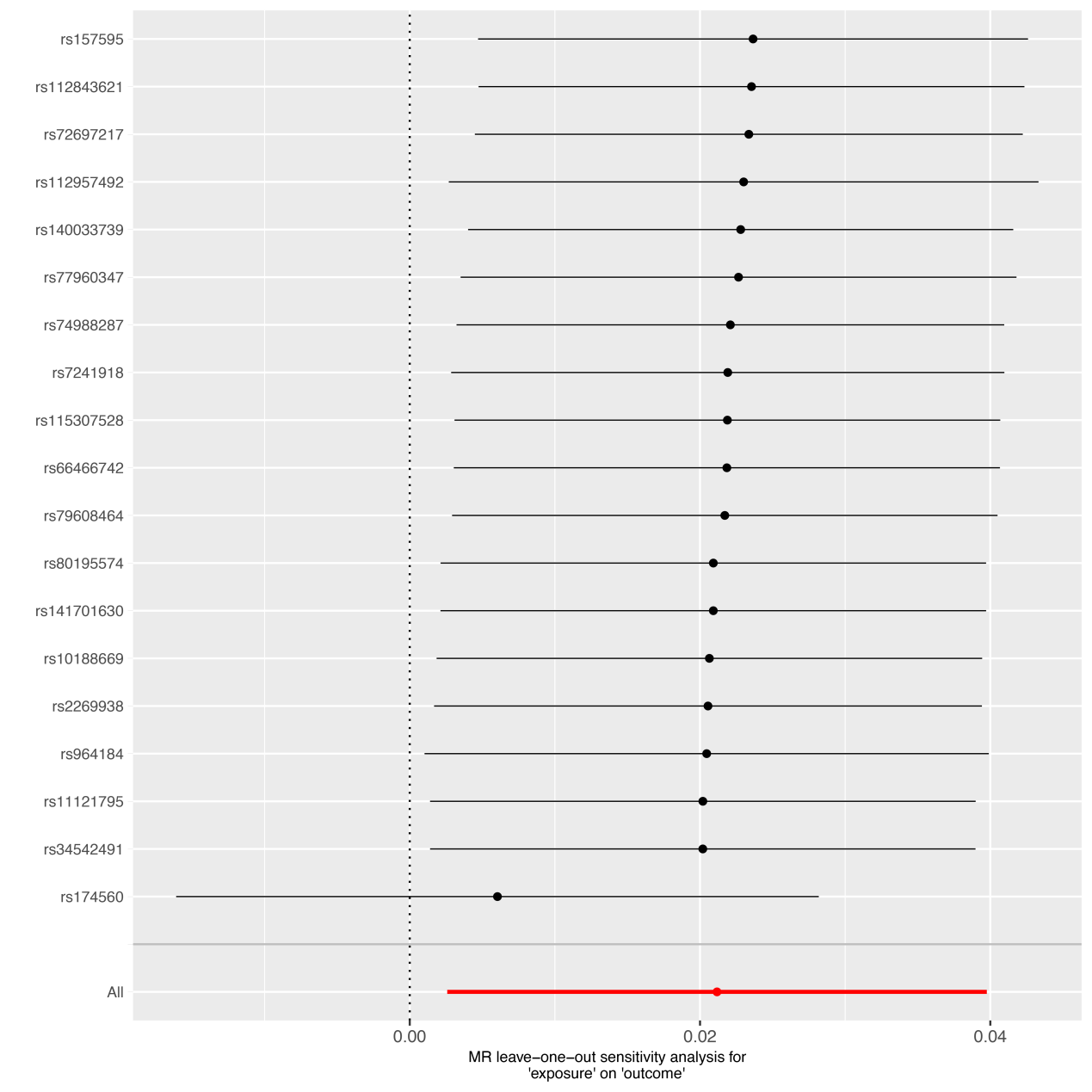


4.GCST90199658


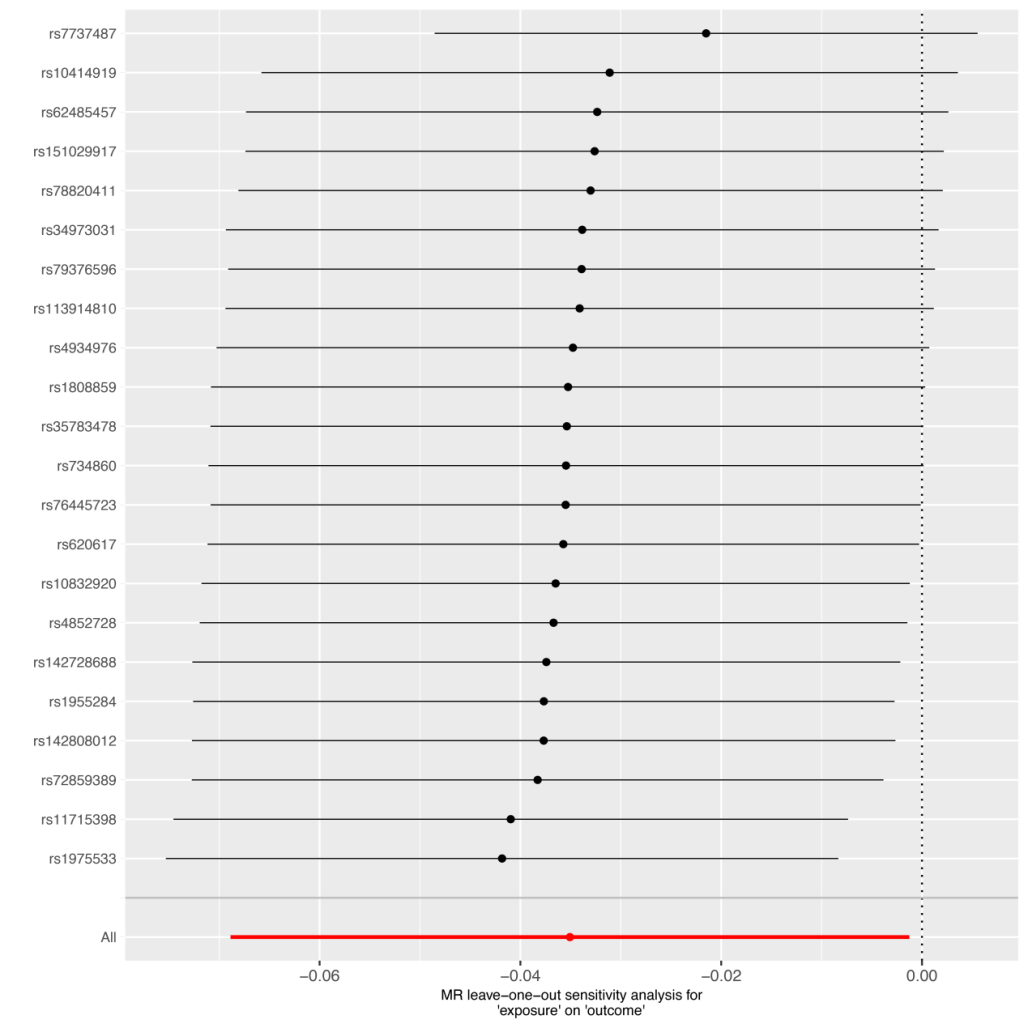


5.GCST90199685


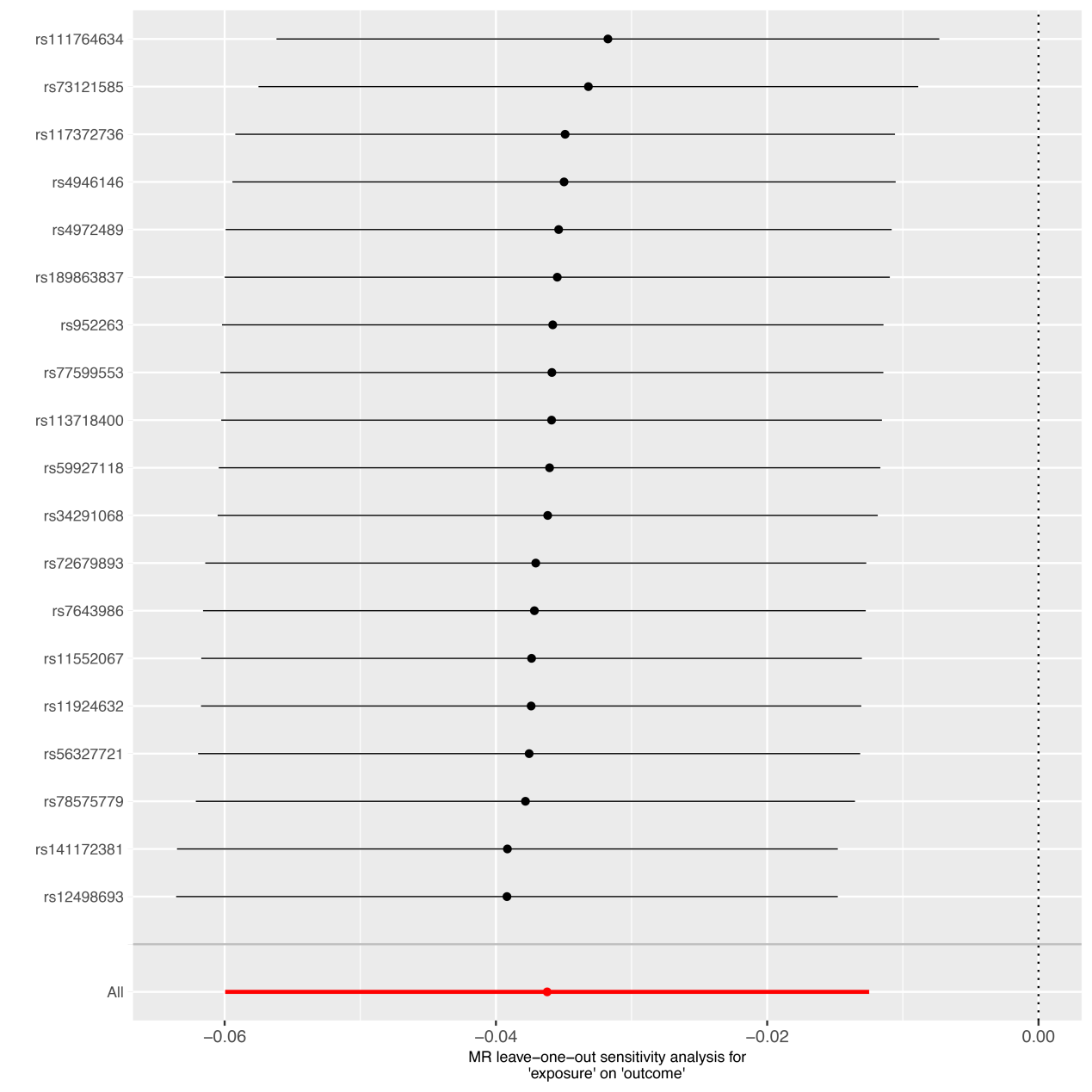


1. GCST90199697


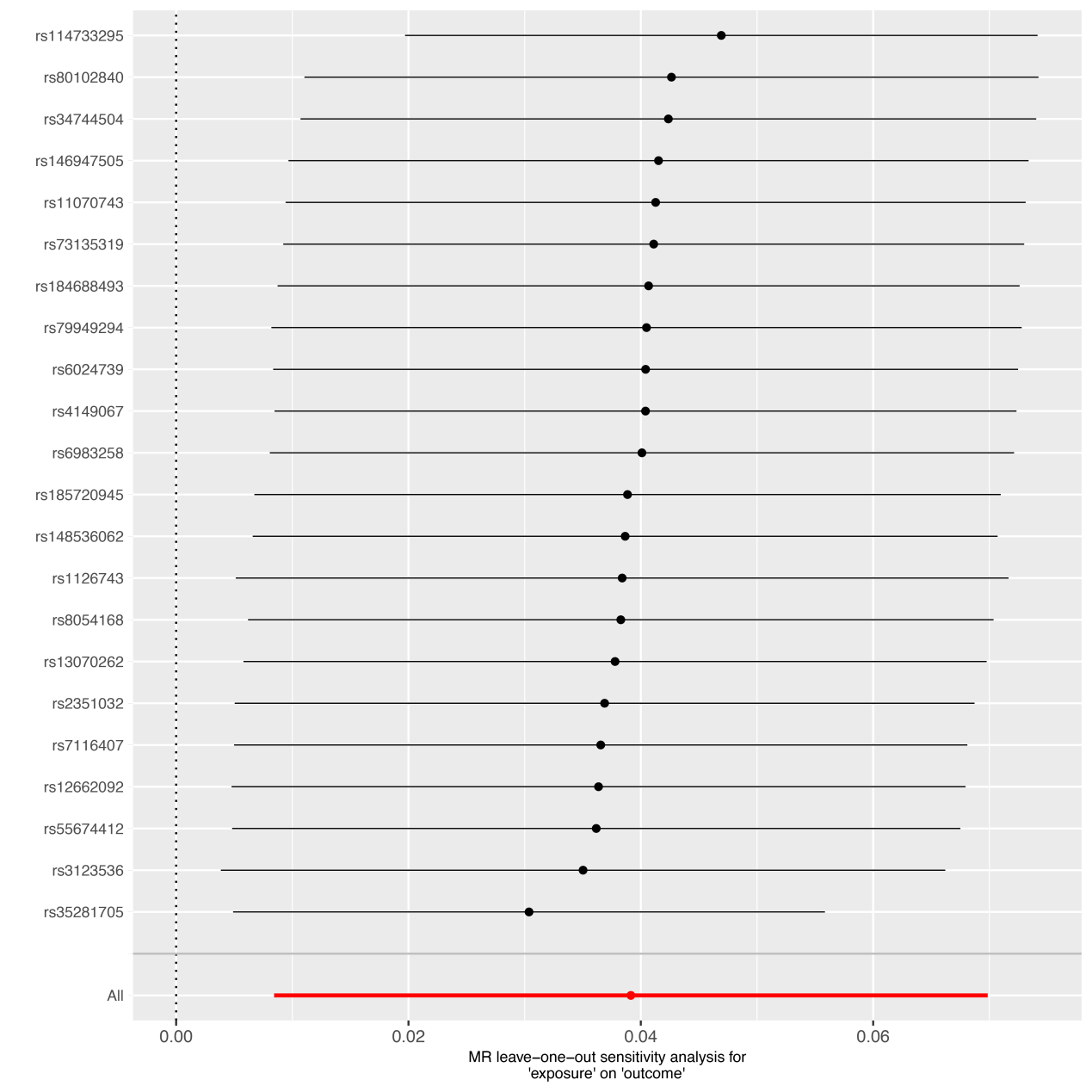


7.GCST90199706


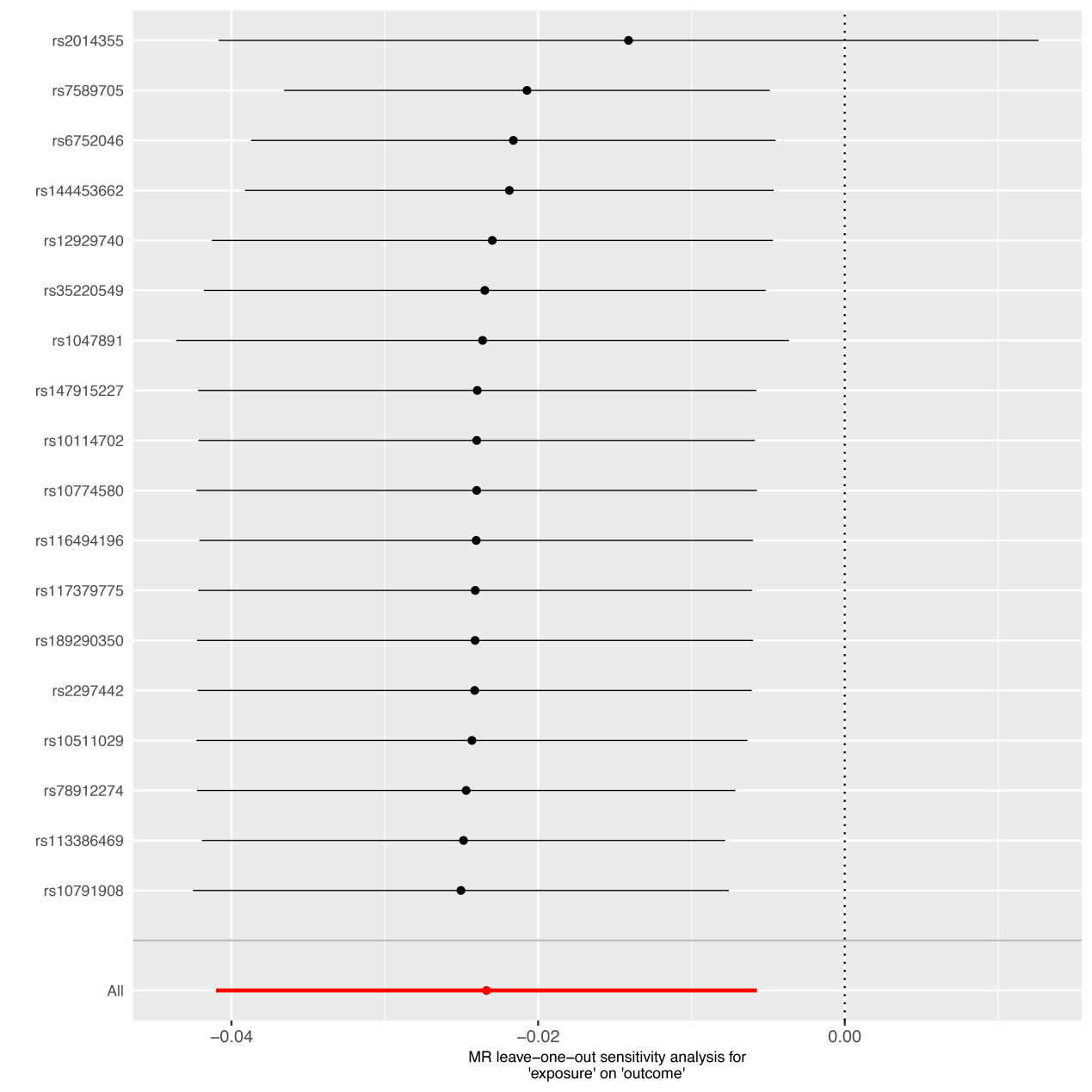


8.GCST90199710


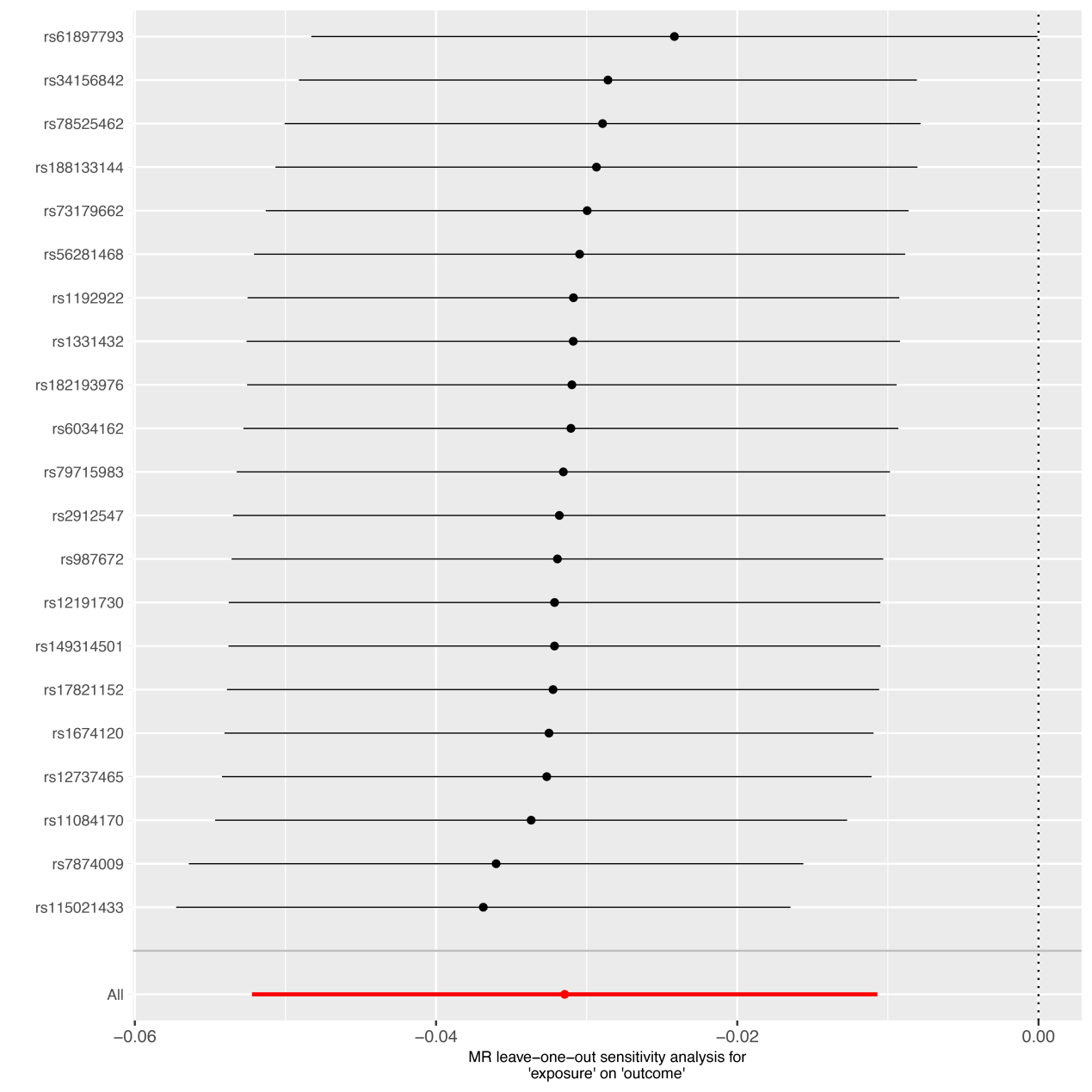


1. GCST90199712


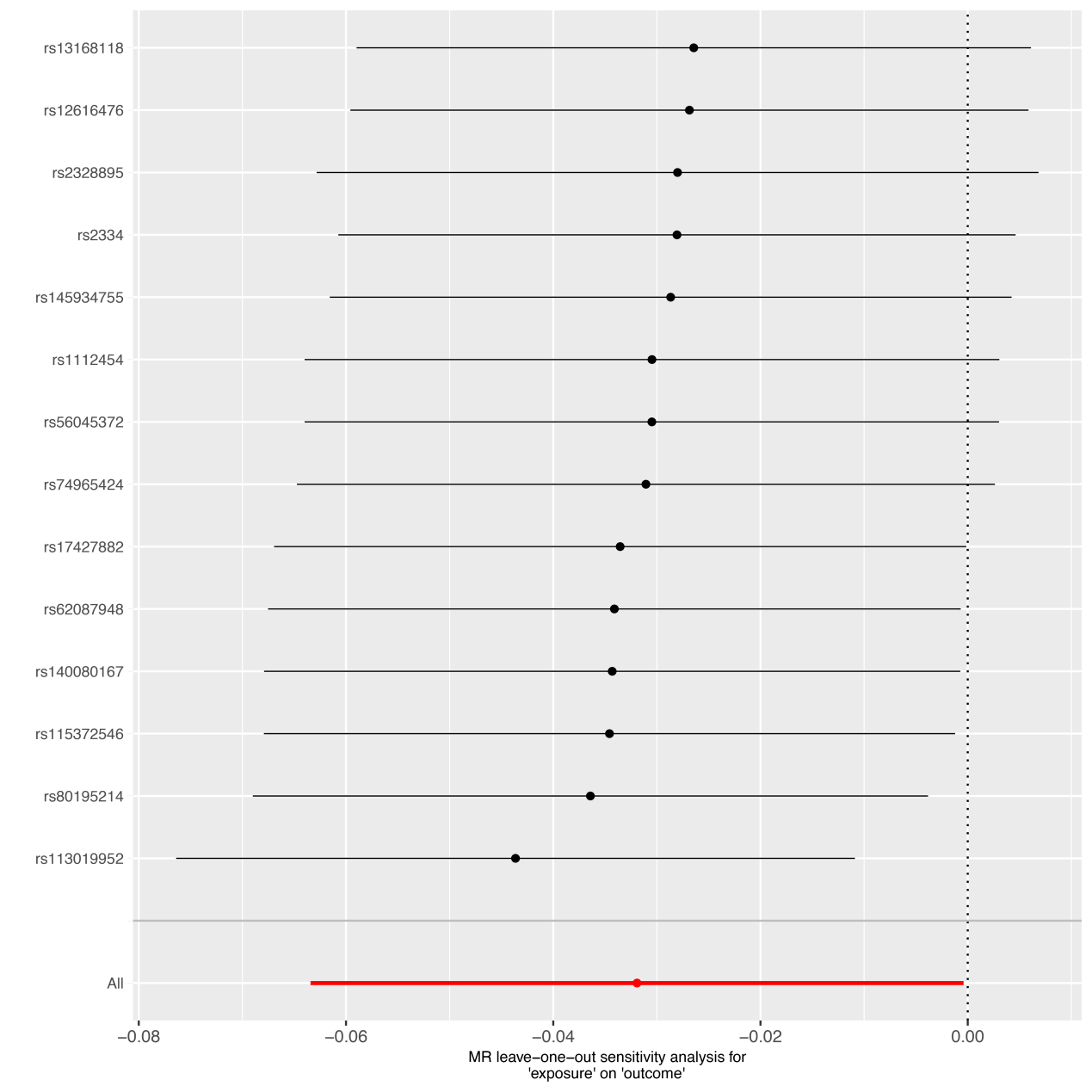


1. GCST90199735


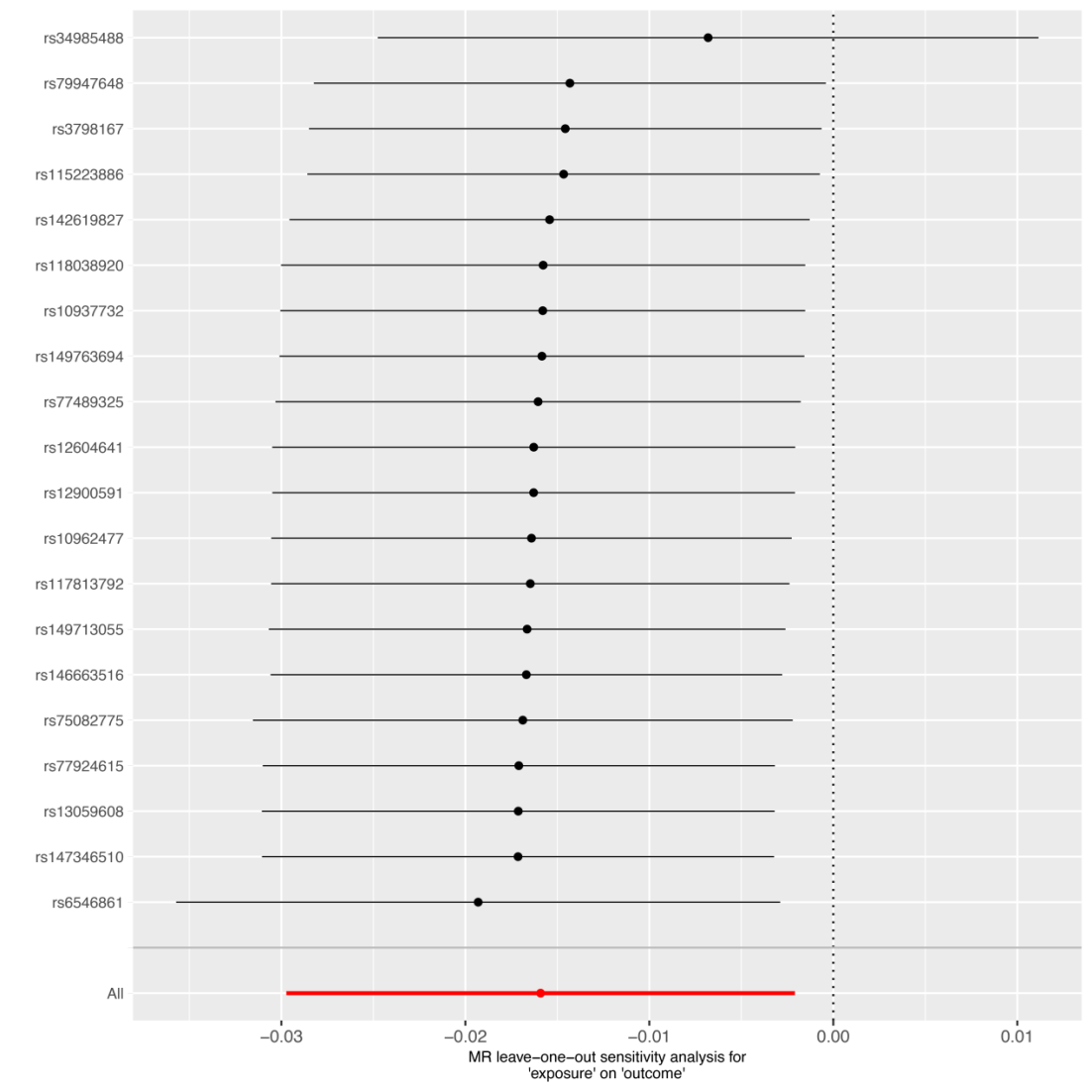


1. GCST90199742


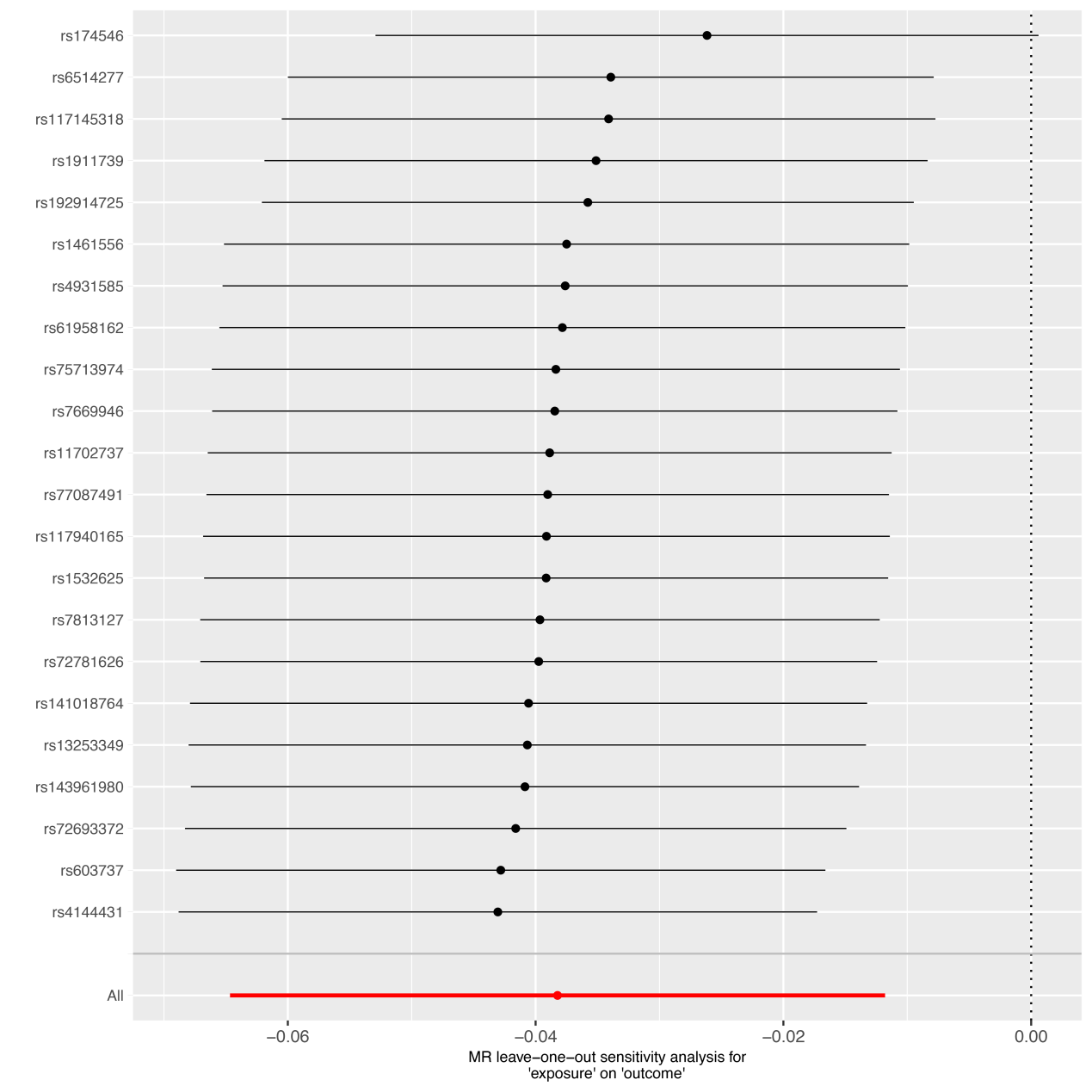


1. GCST90199770


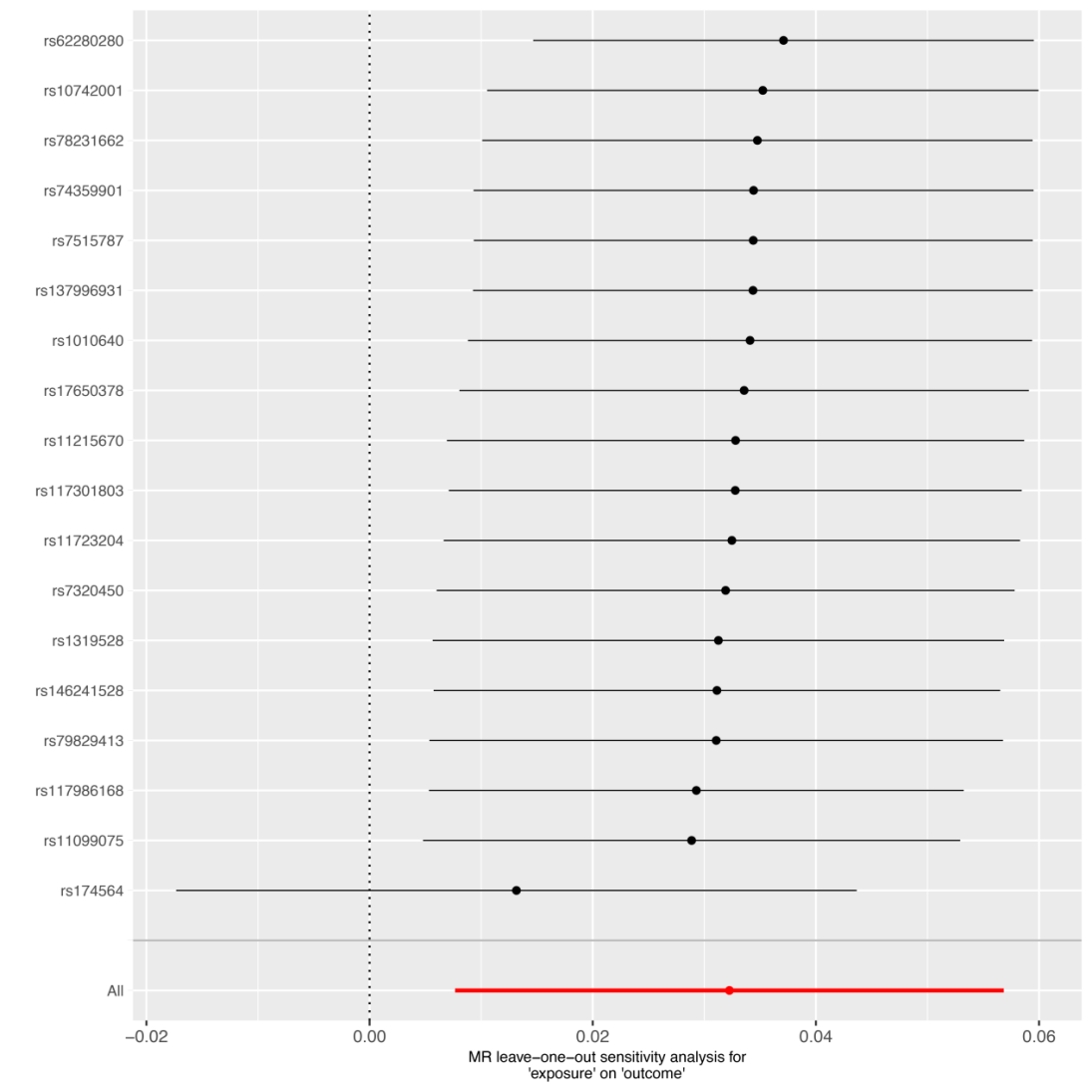


1. GCST90199772


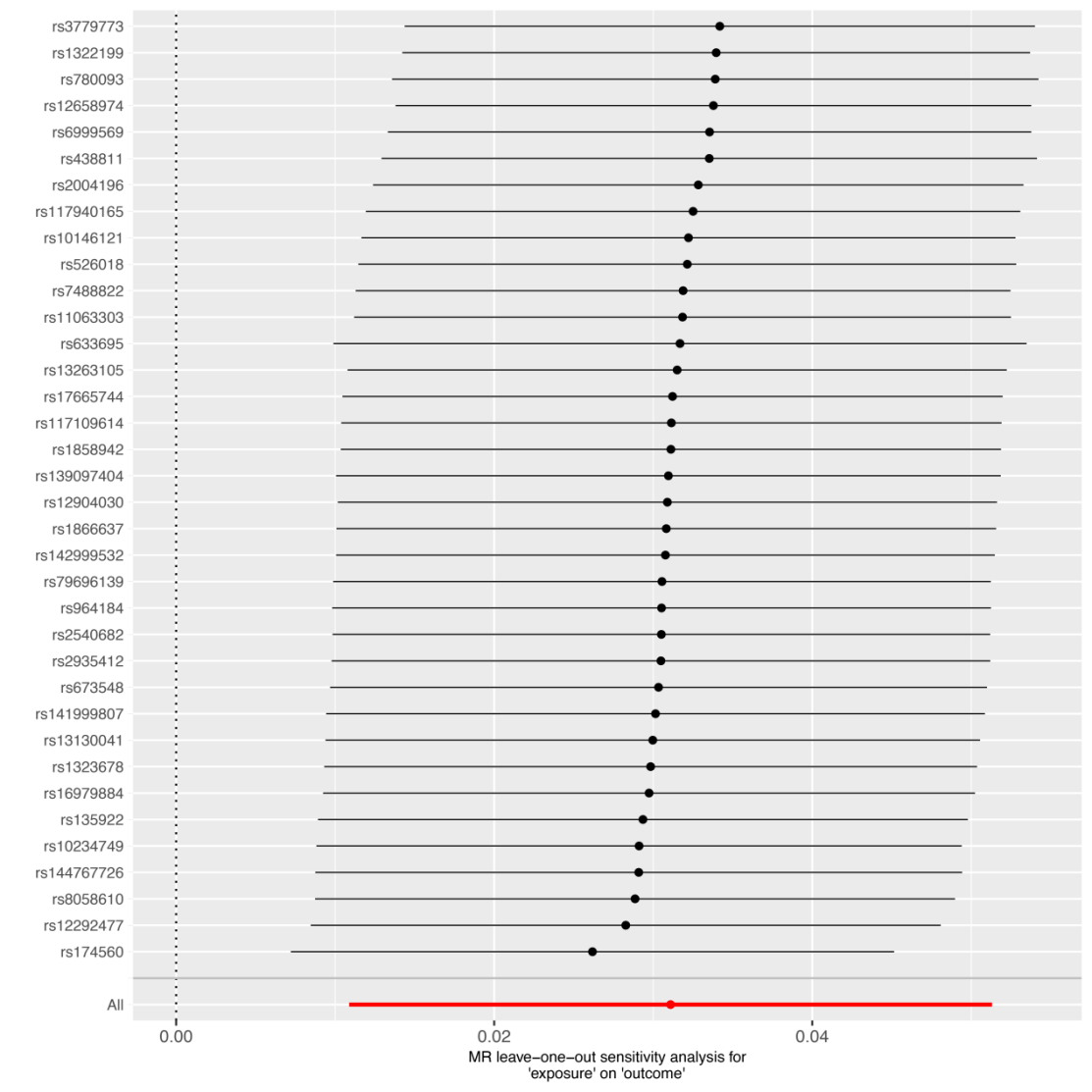


1. GCST9019977


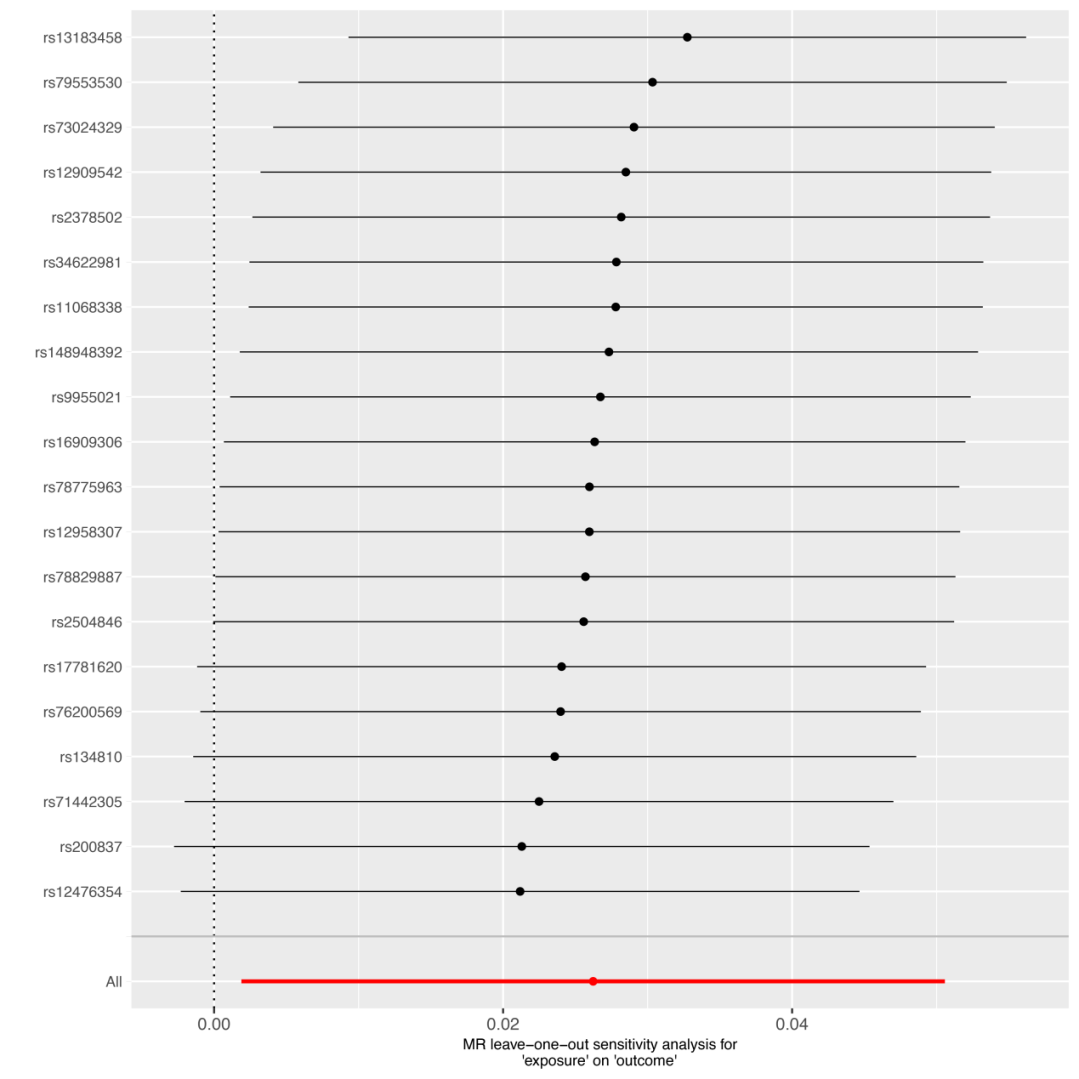


1. GCST90199786


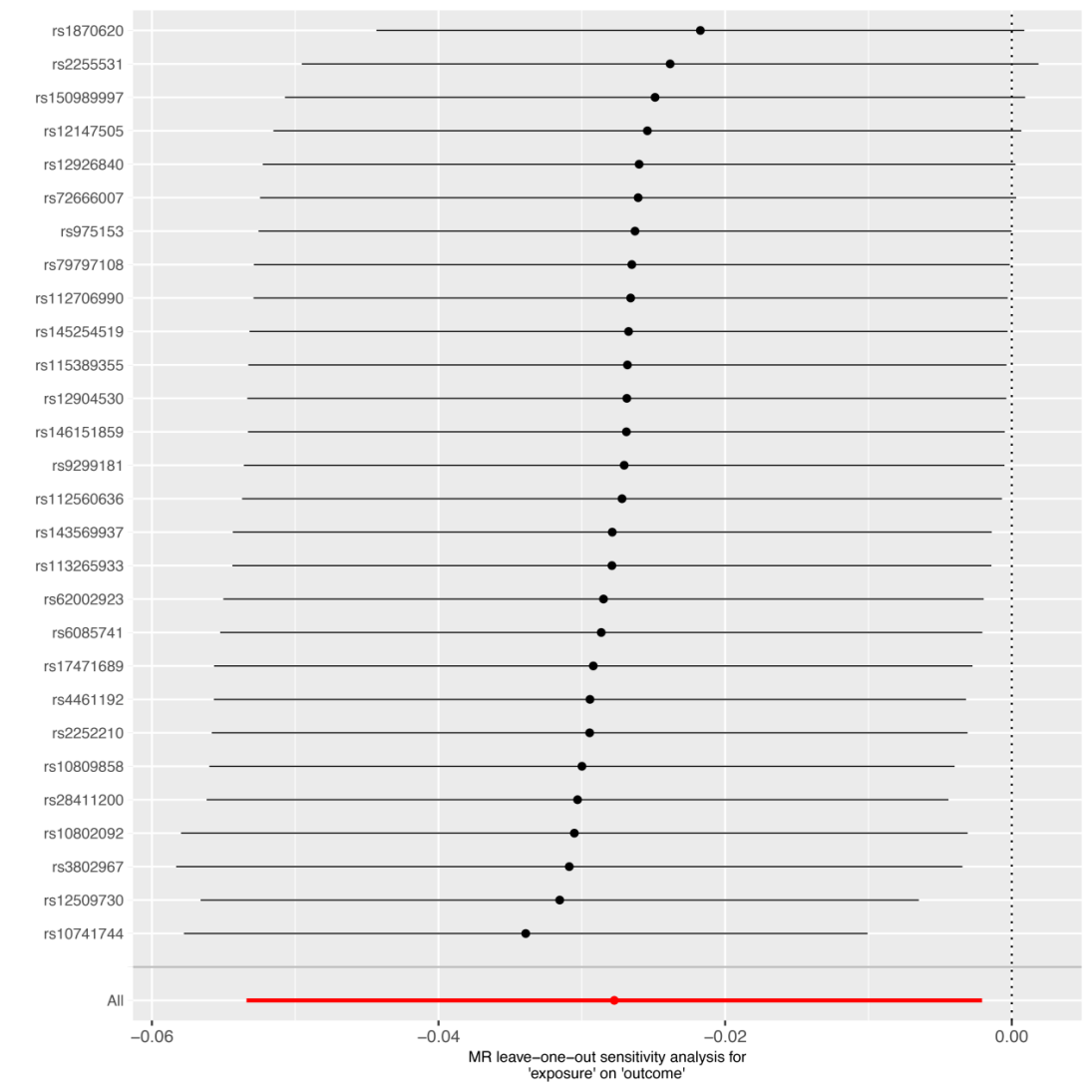


16.GCST90199788


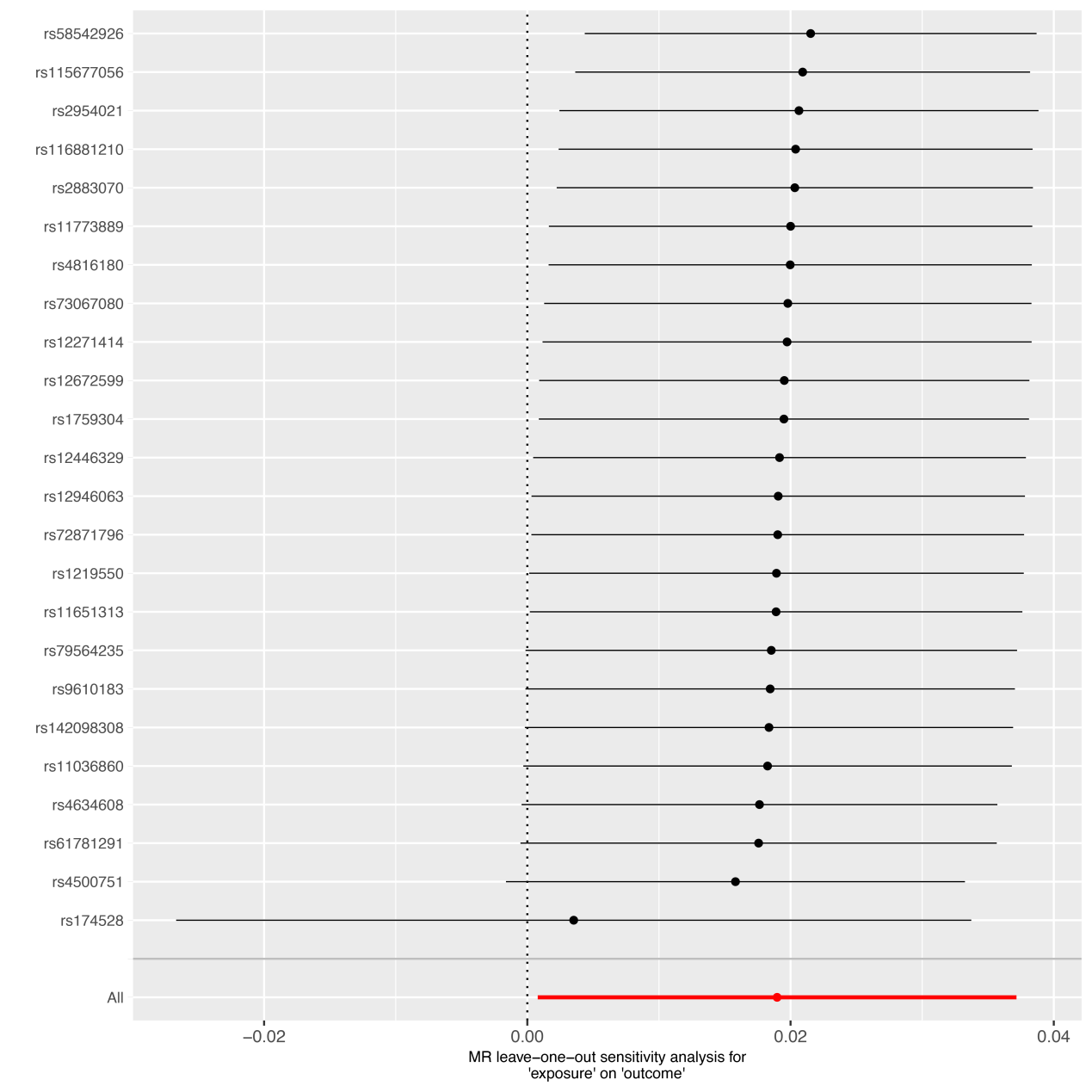


17.GCST90199826


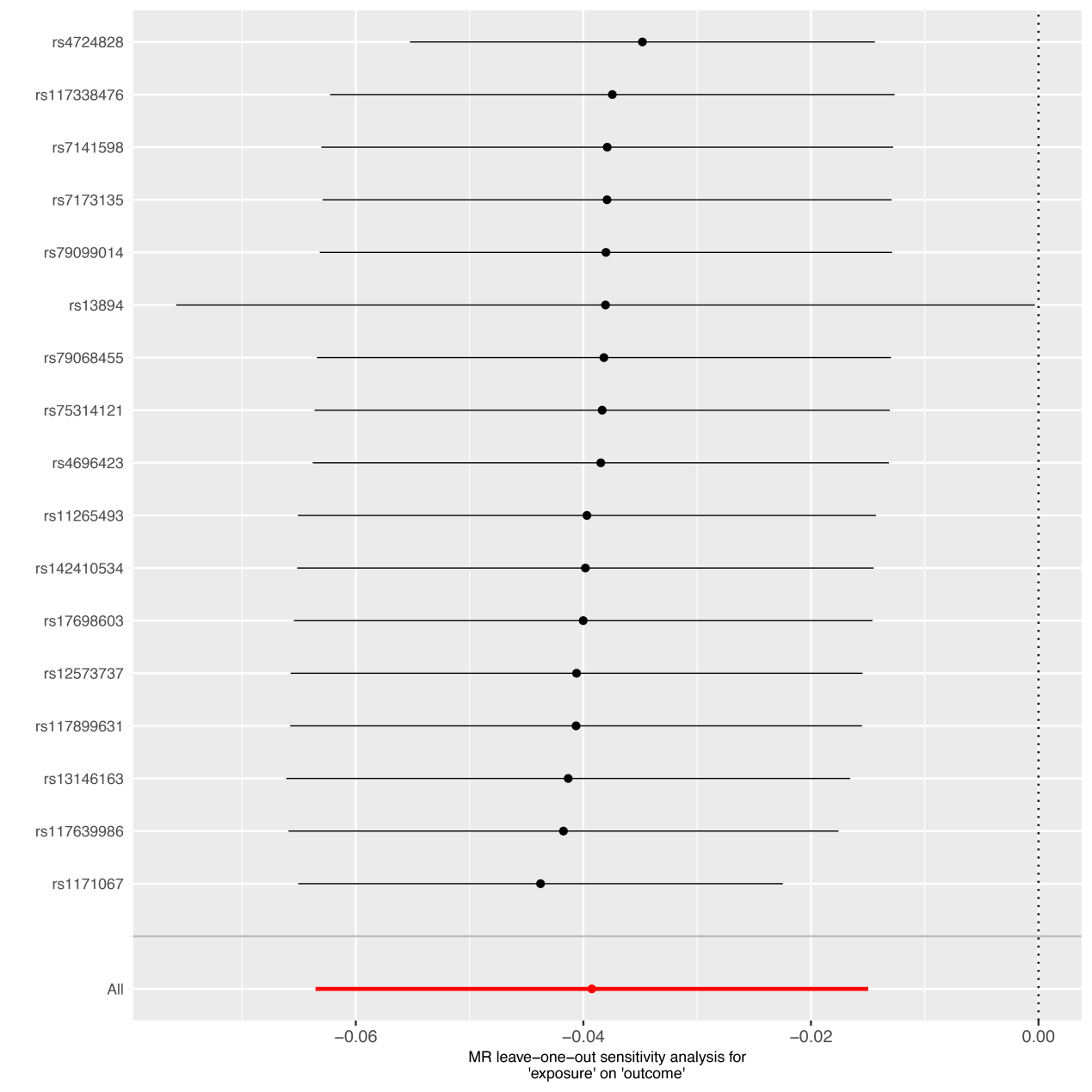


18.GCST90199832


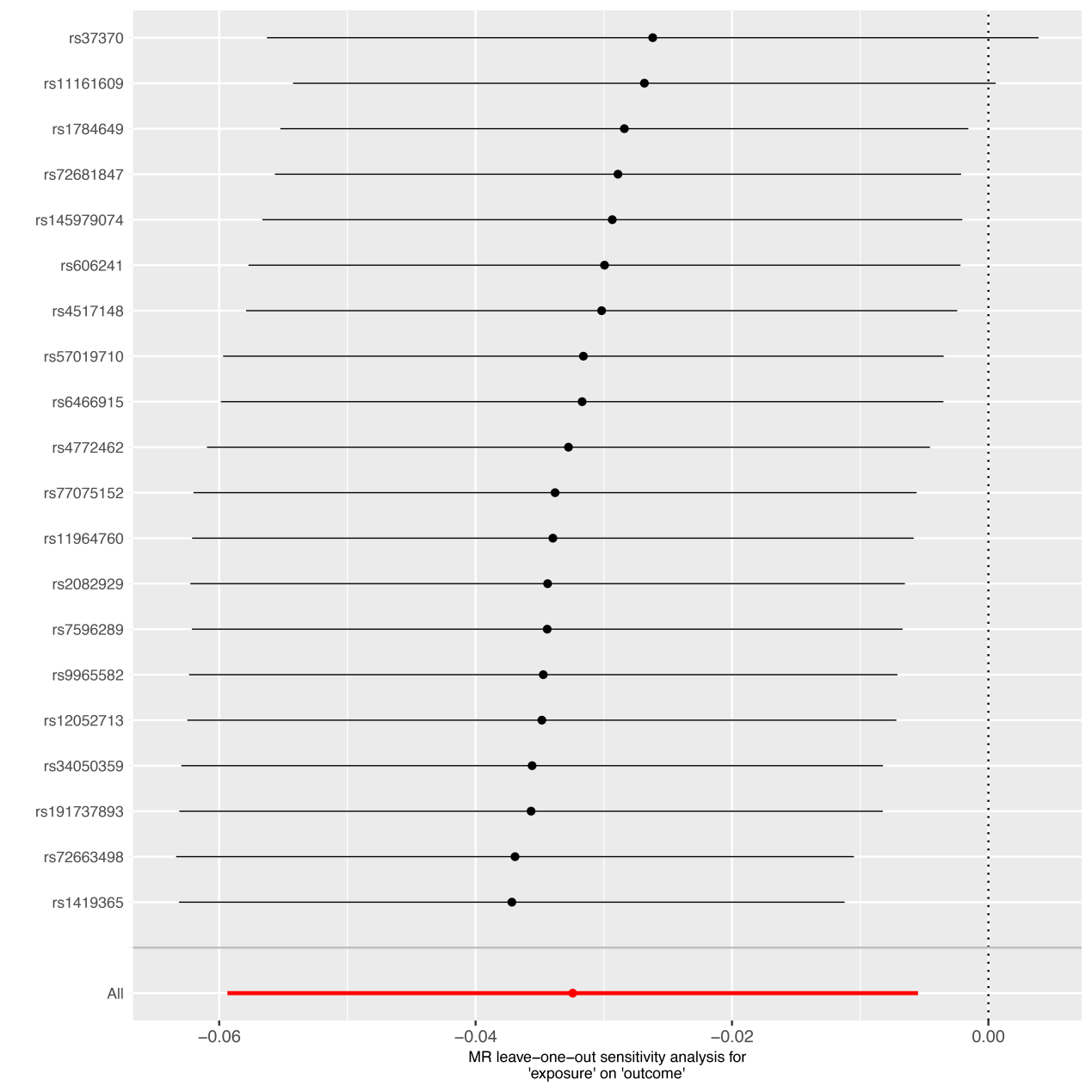


19.GCST90199866


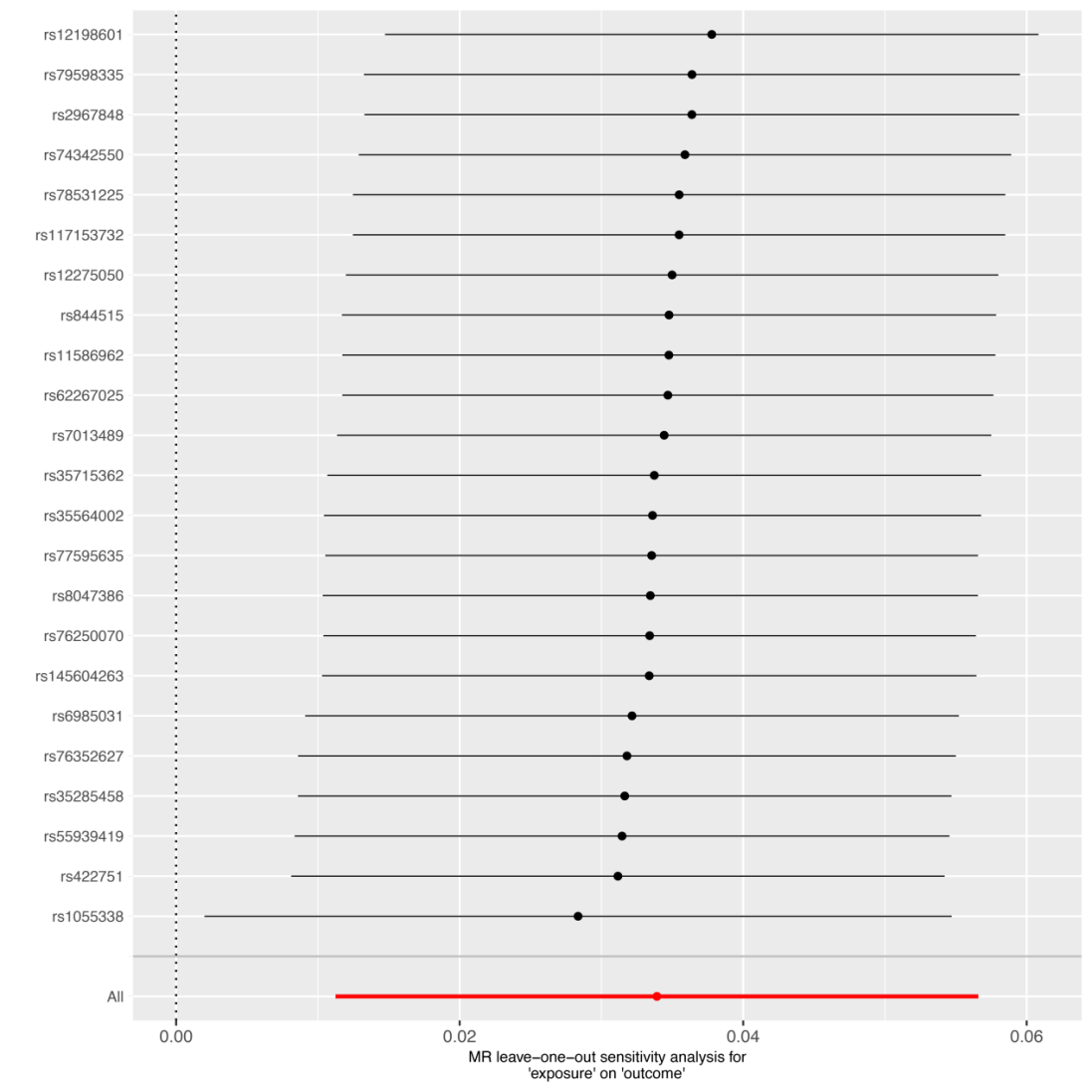


20.GCST90199895


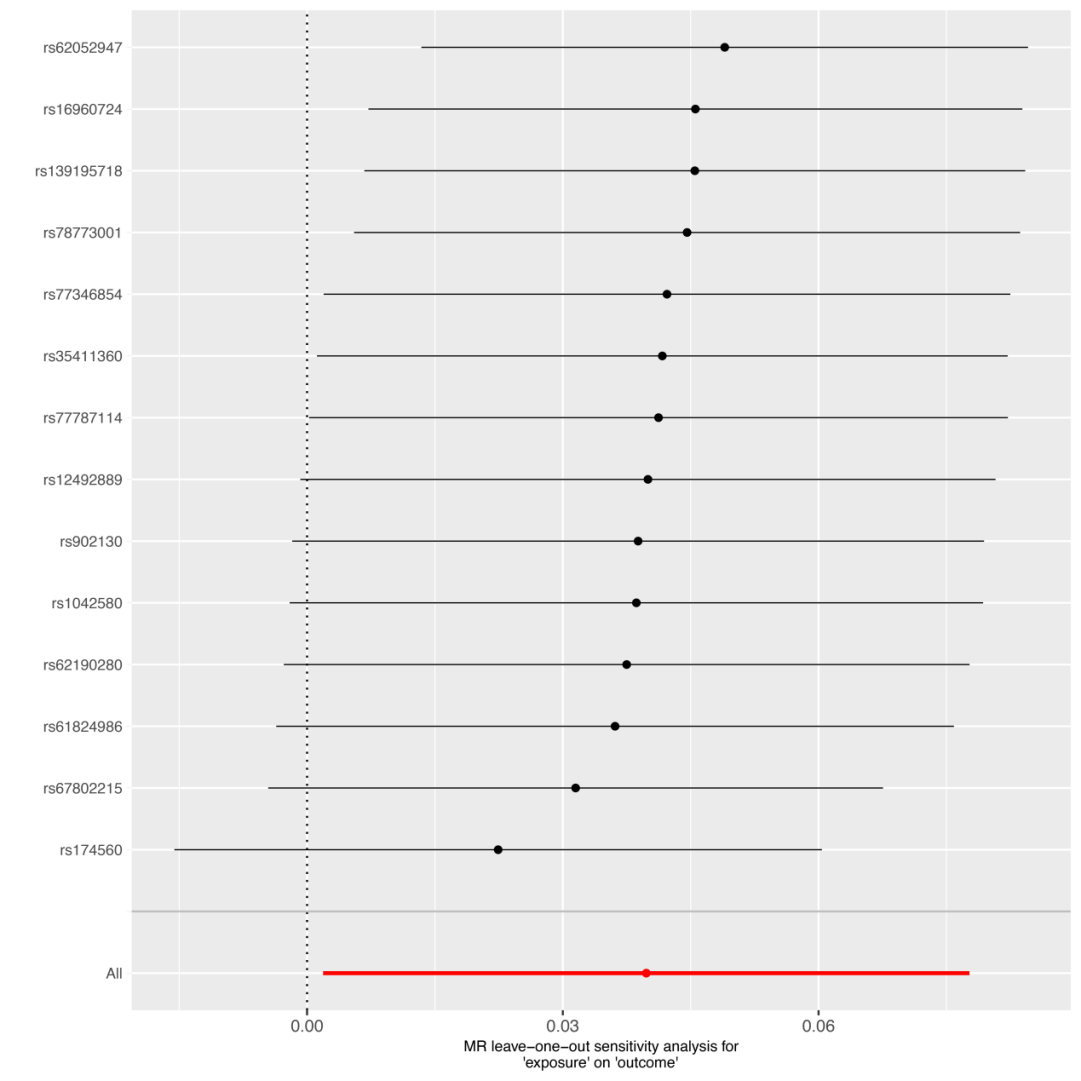


21.GCST90199903


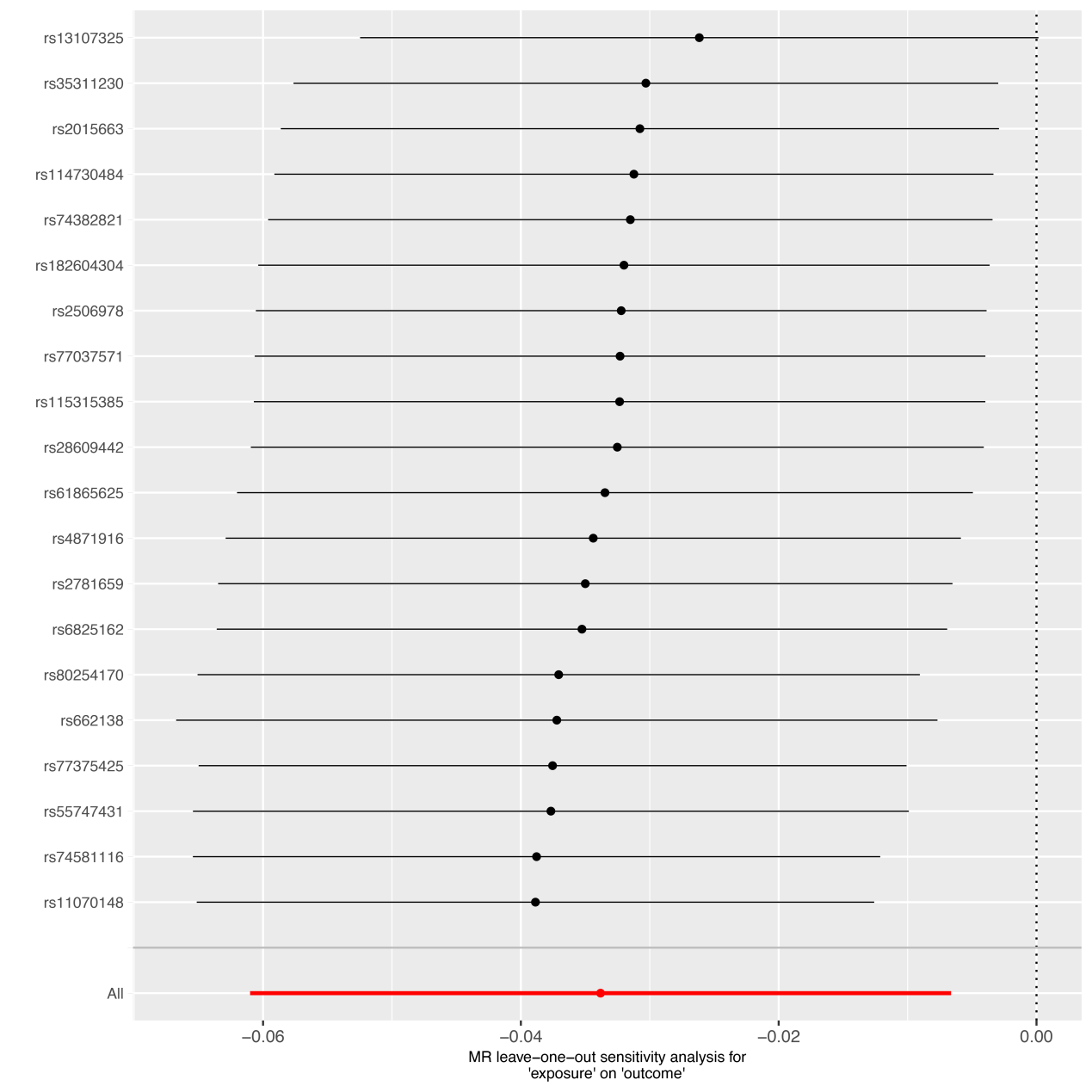


22.GCST90199917


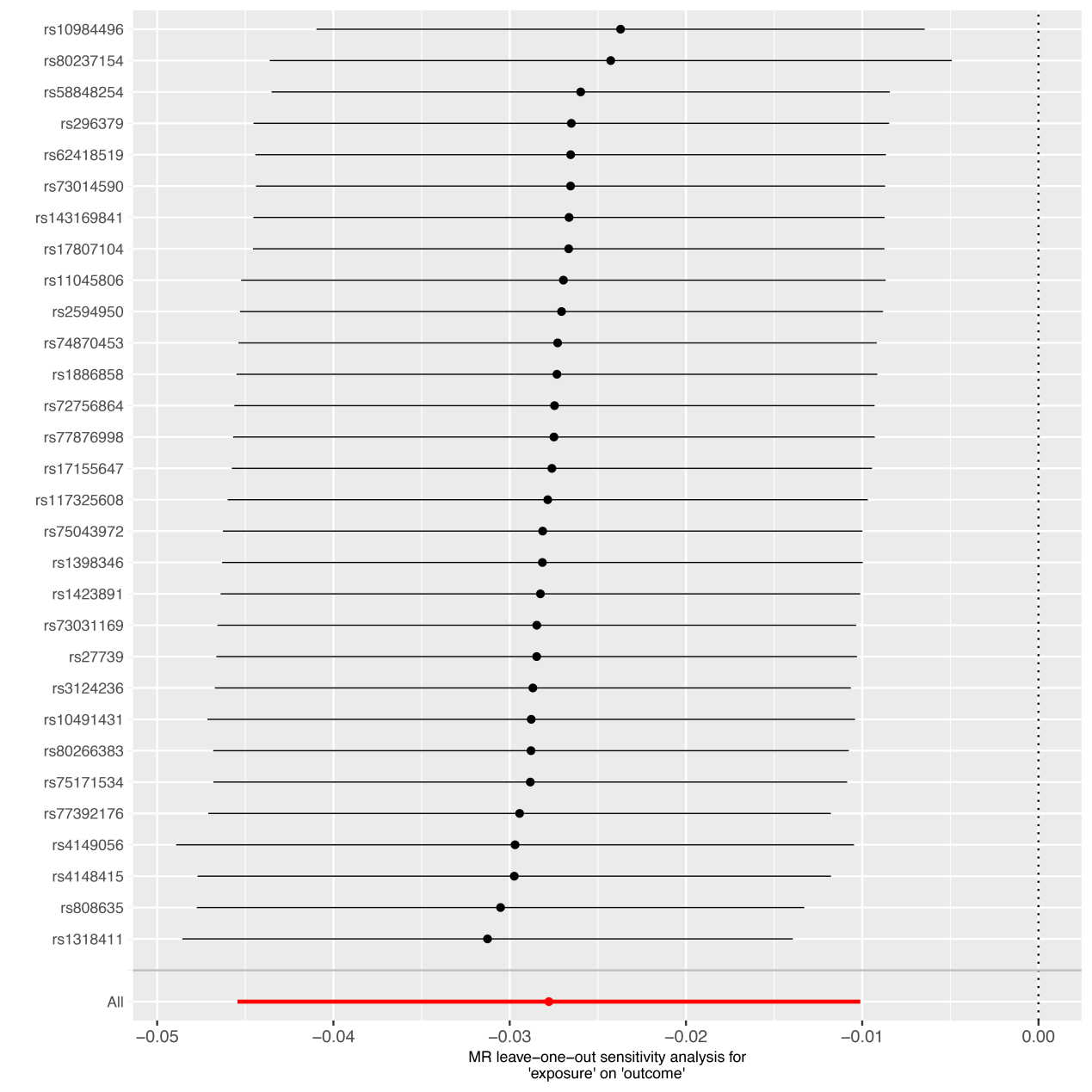


23.GCST90199949


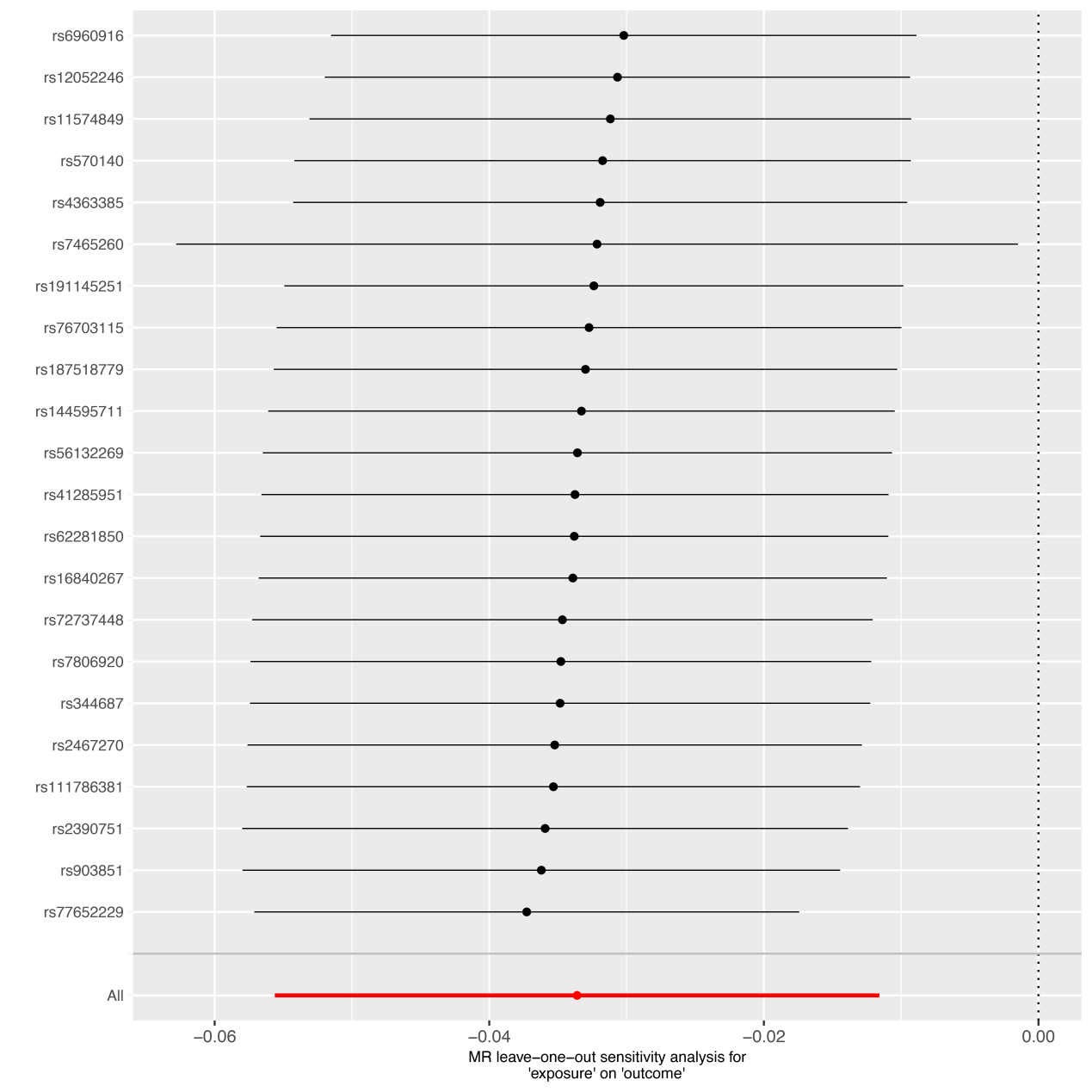


24.GCST90199990


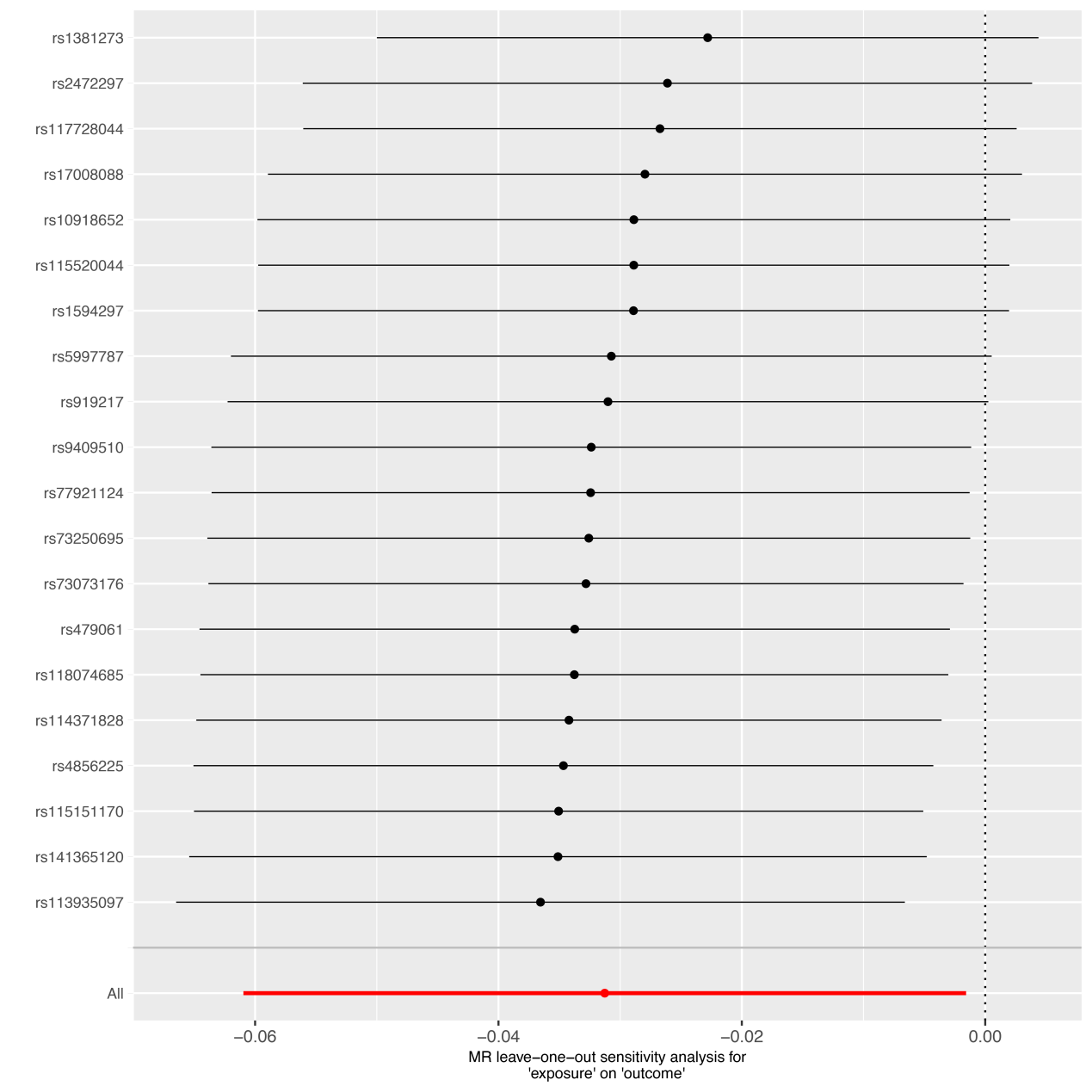


25.GCST90200037


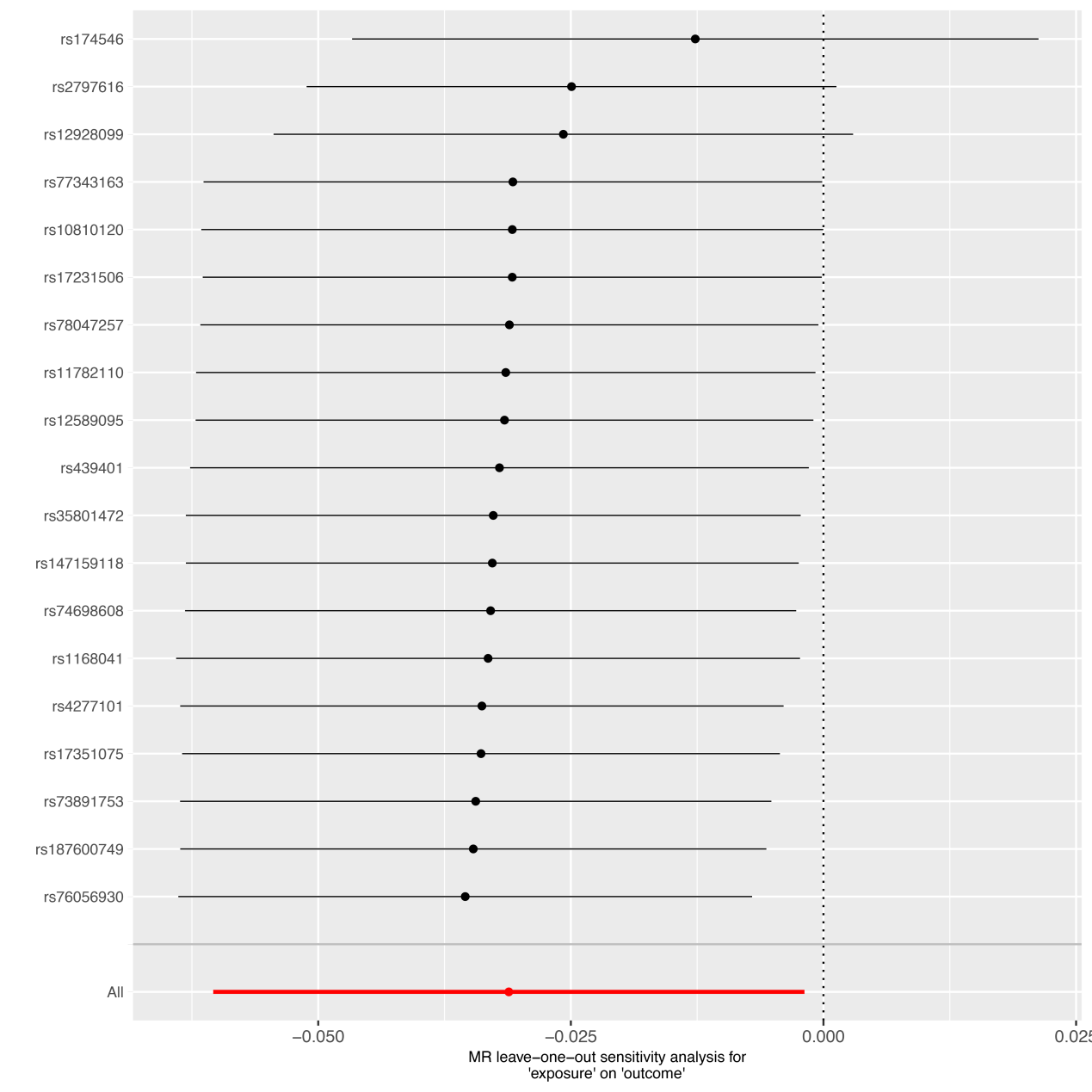


26.GCST90200043


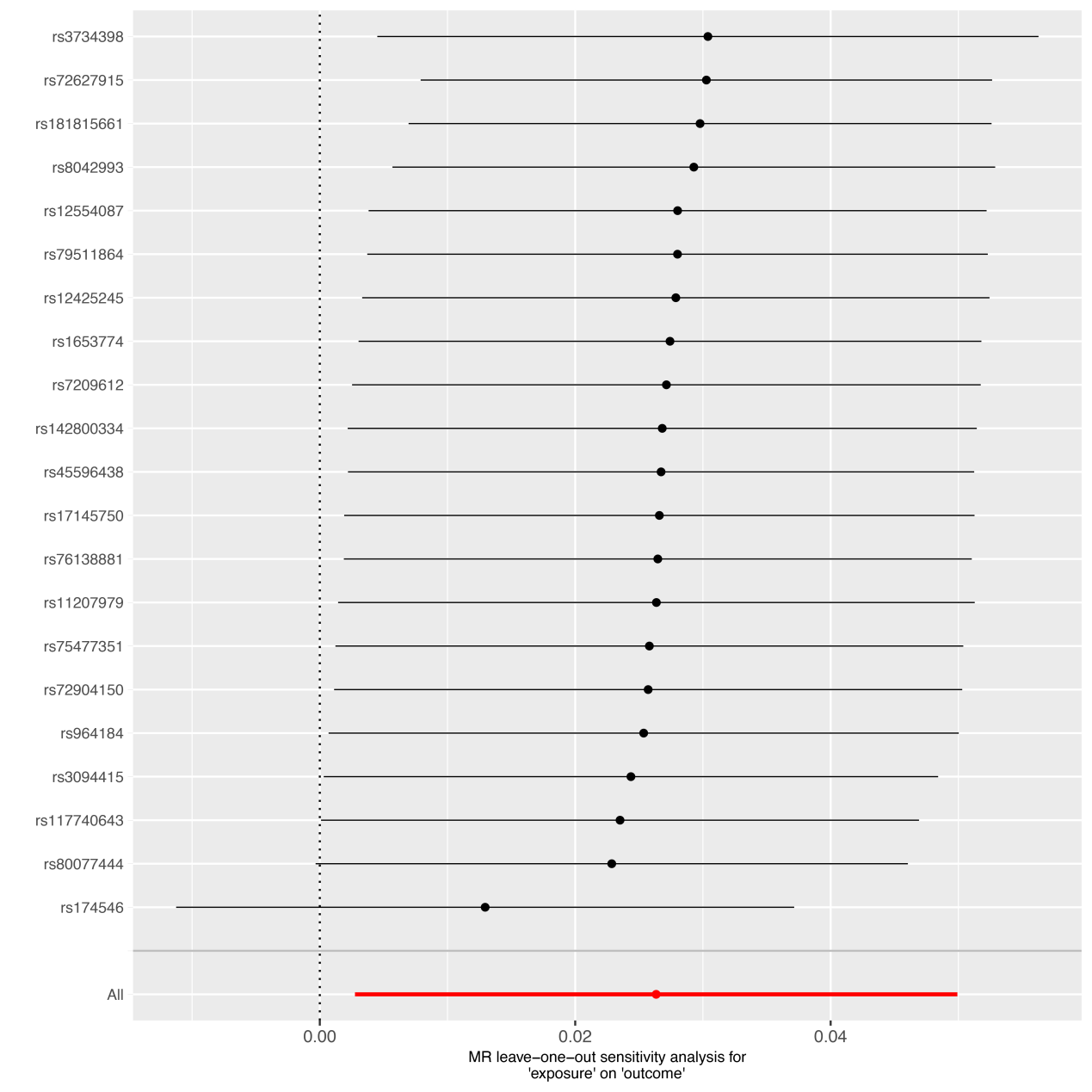


1. GCST90200052


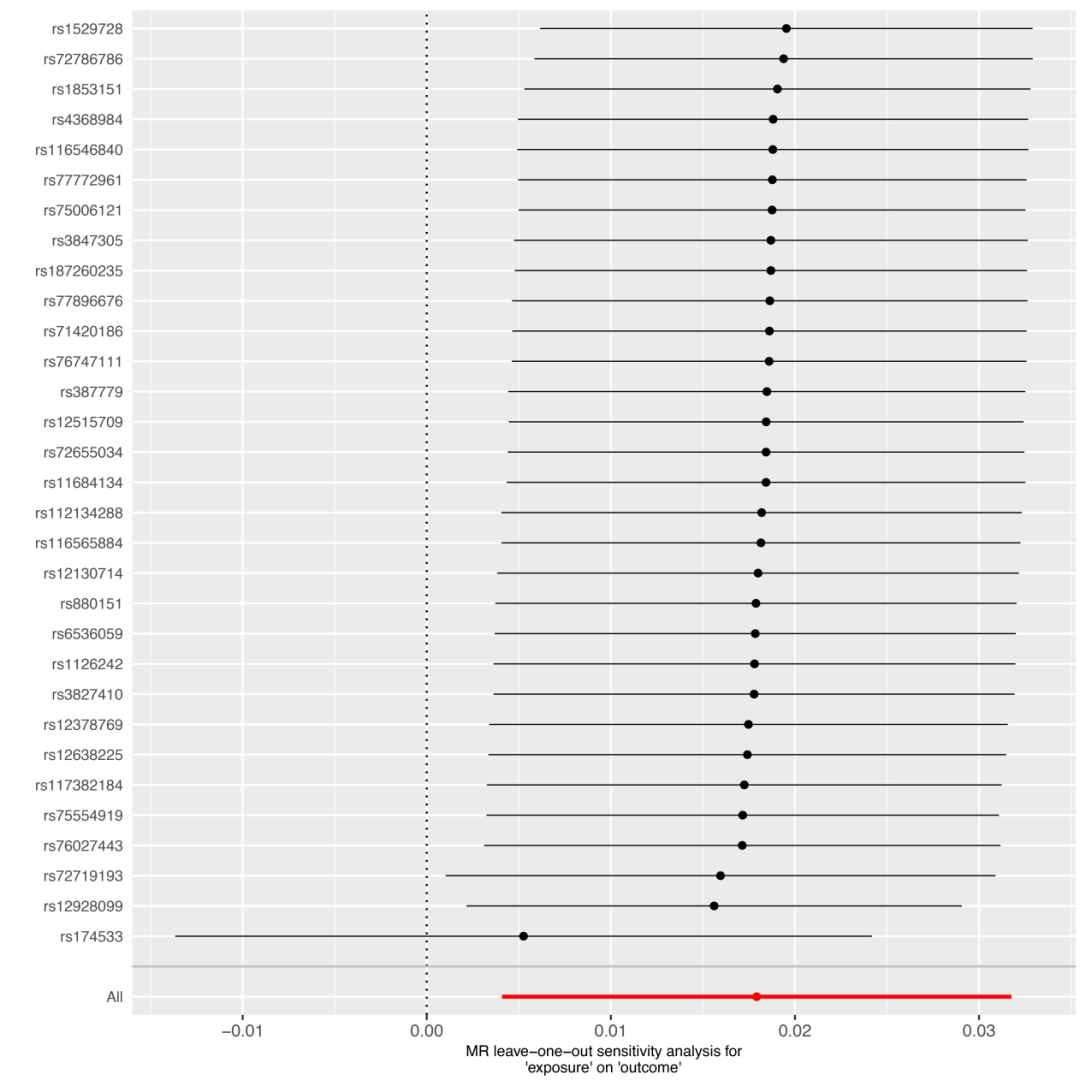


28.GCST90200054


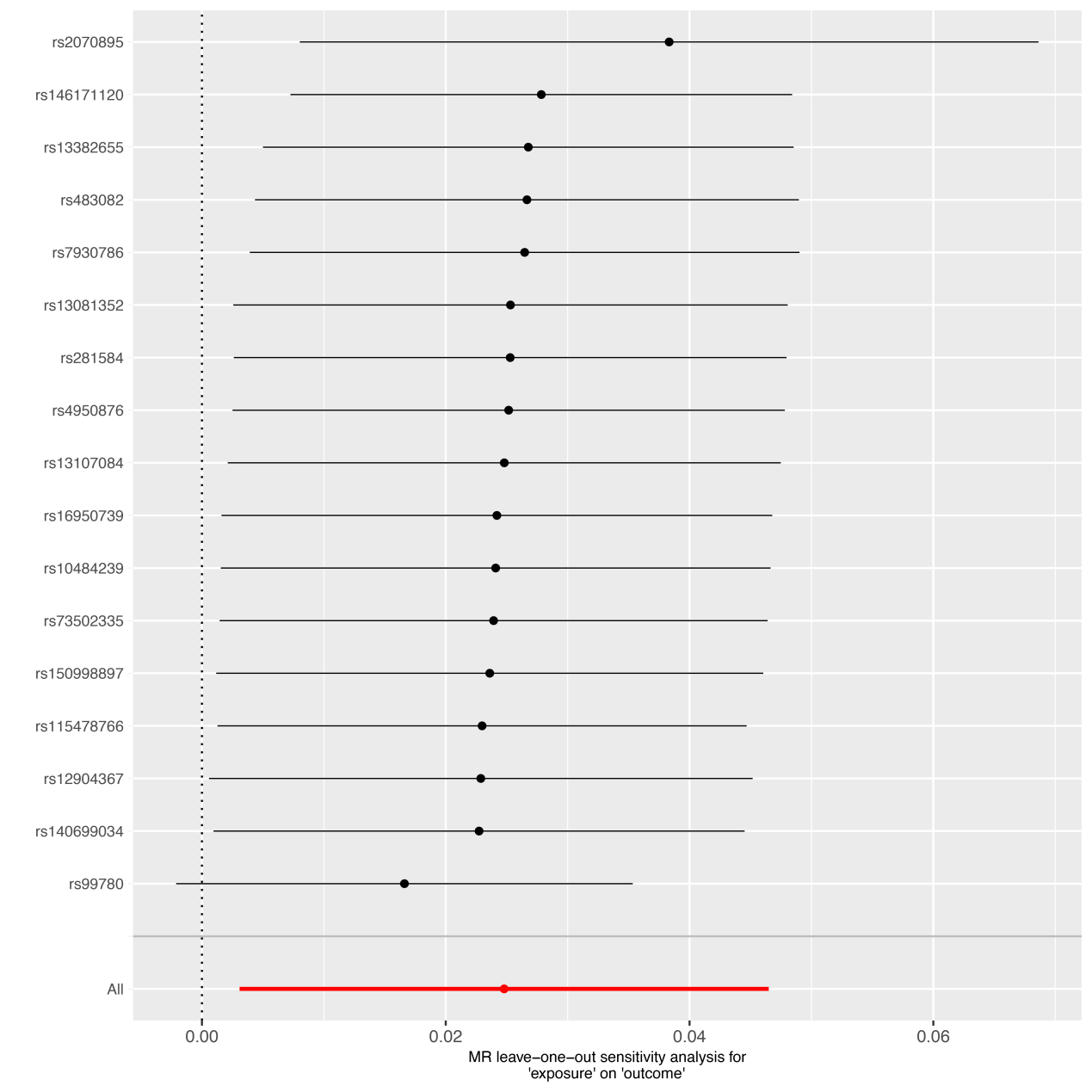


29.GCST90200058


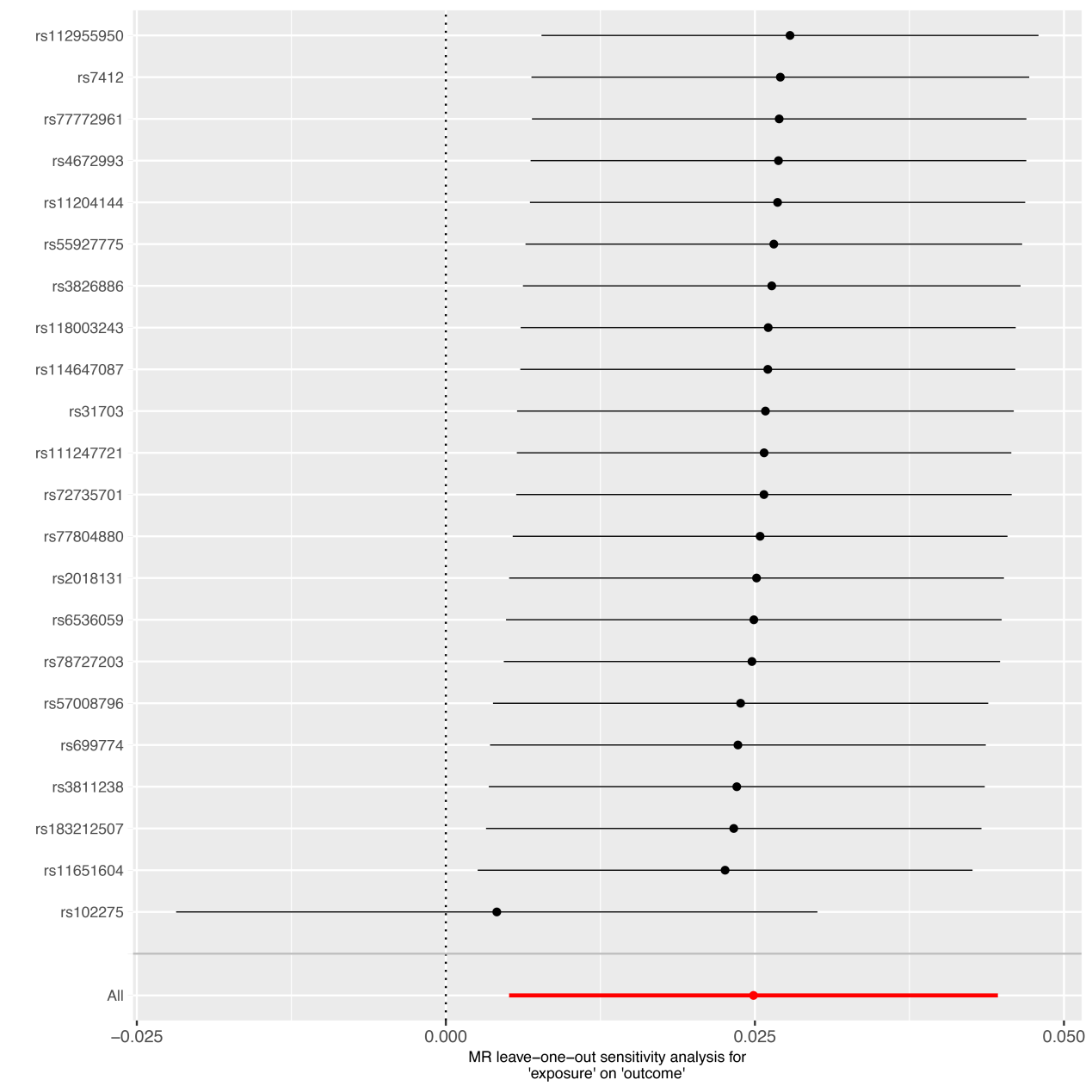


30.GCST90200062


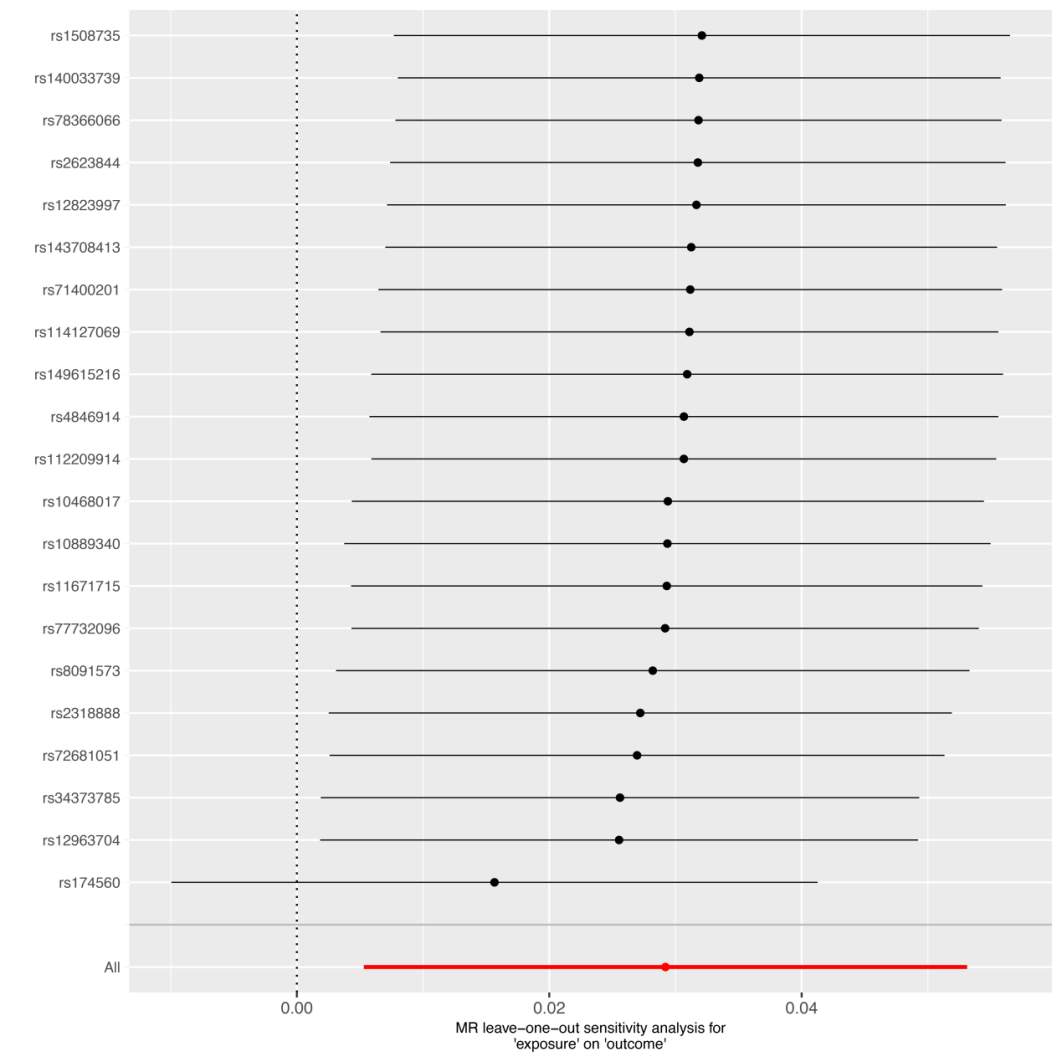


31.GCST90200070


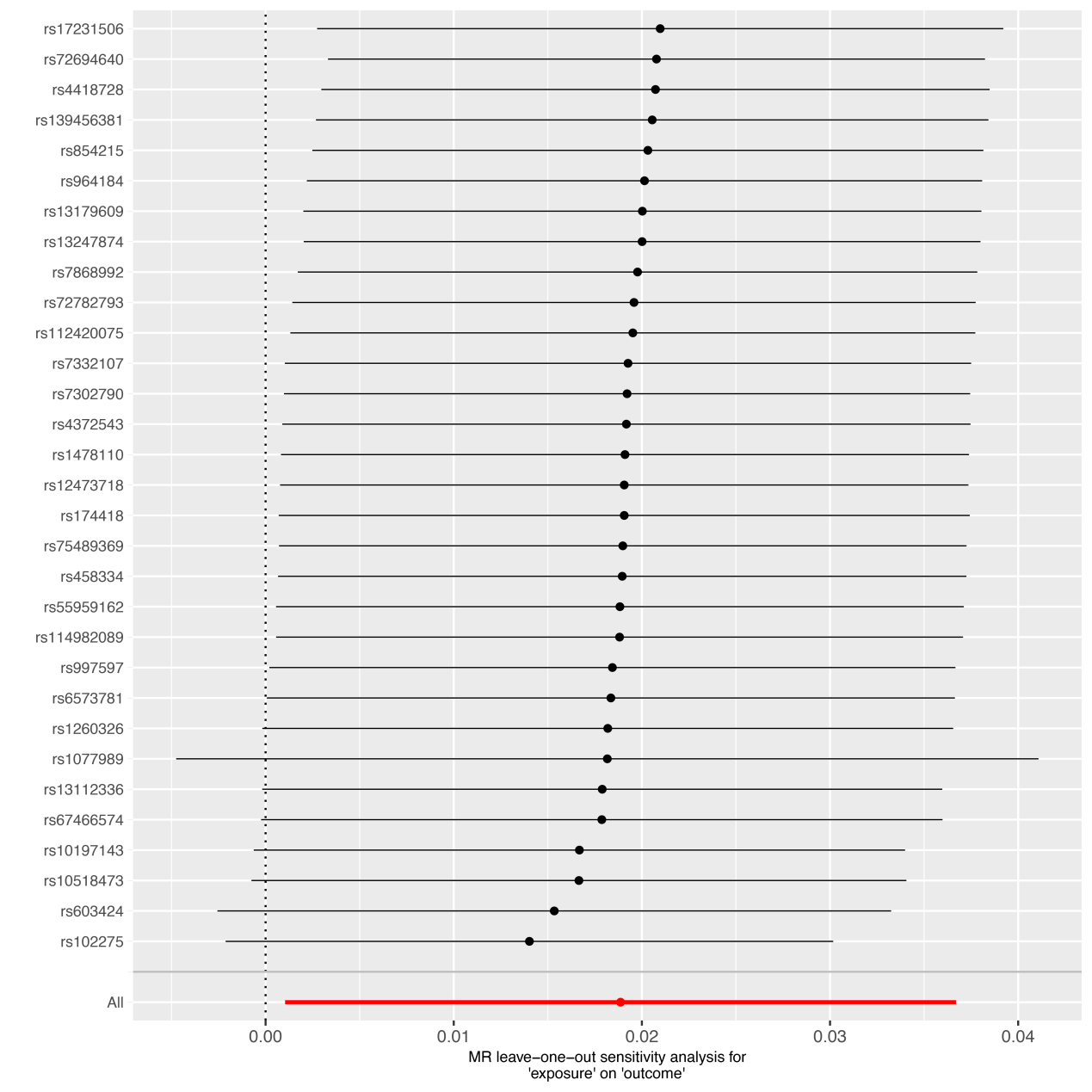


32.GCST90200082


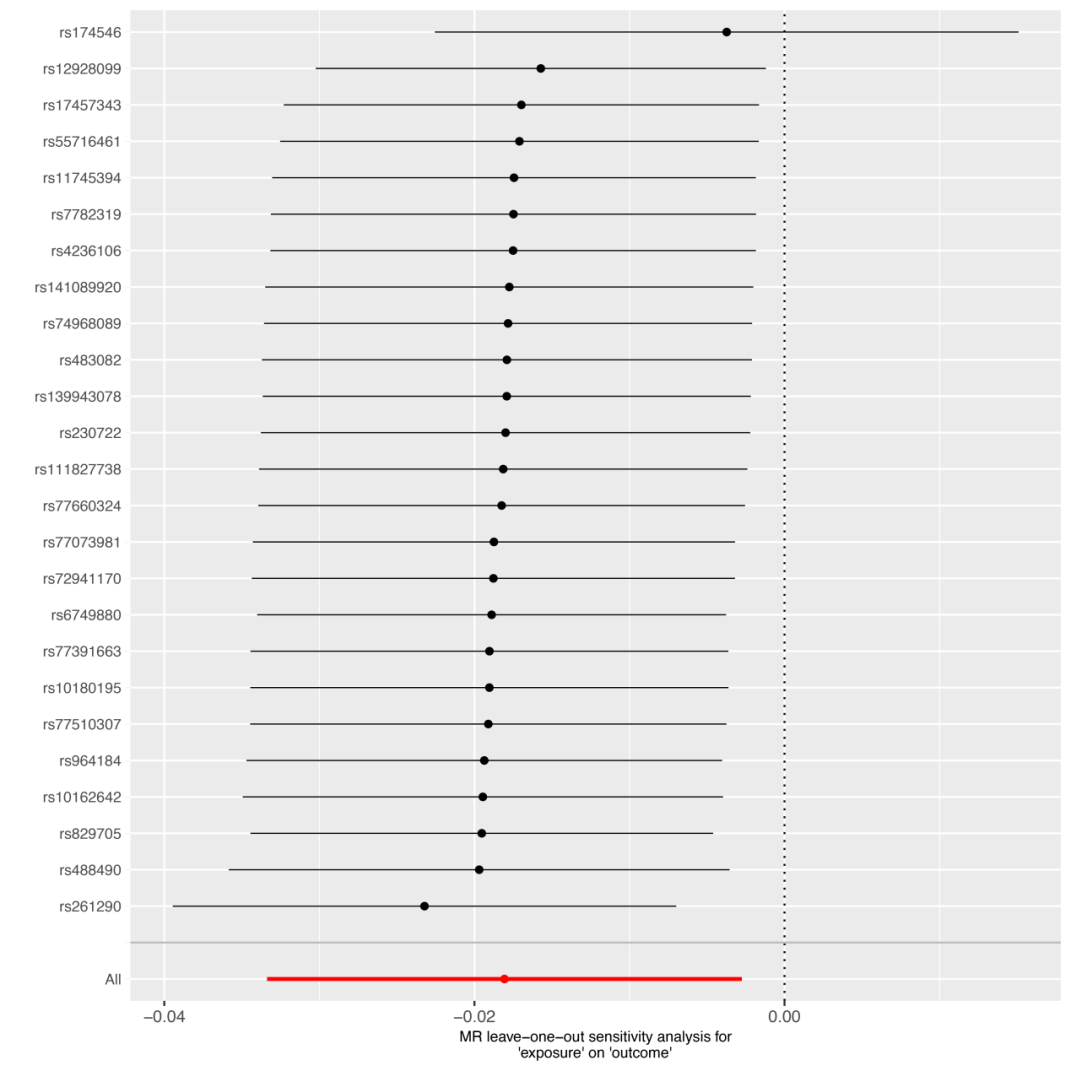


33.GCST90200095


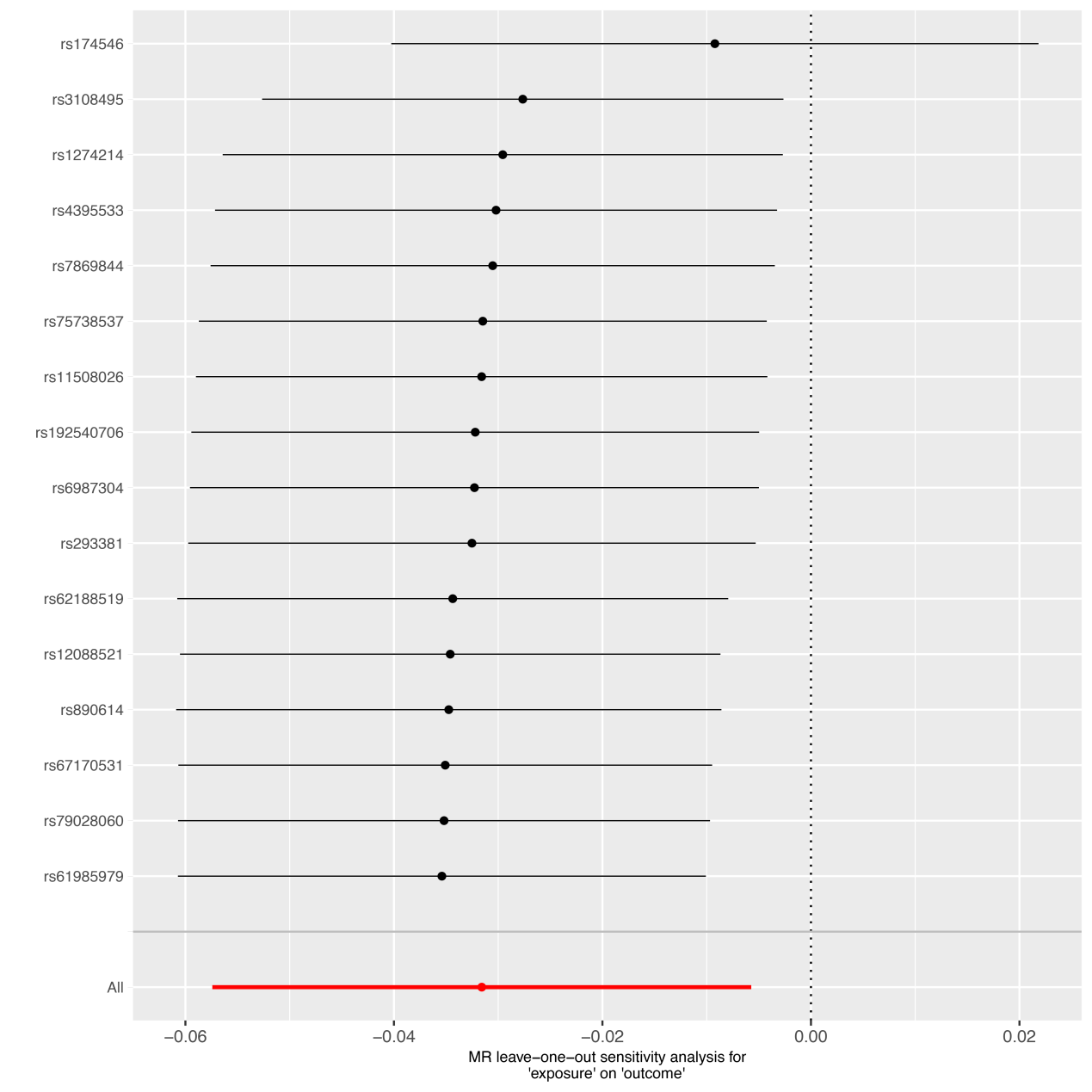


34.GCST90200103


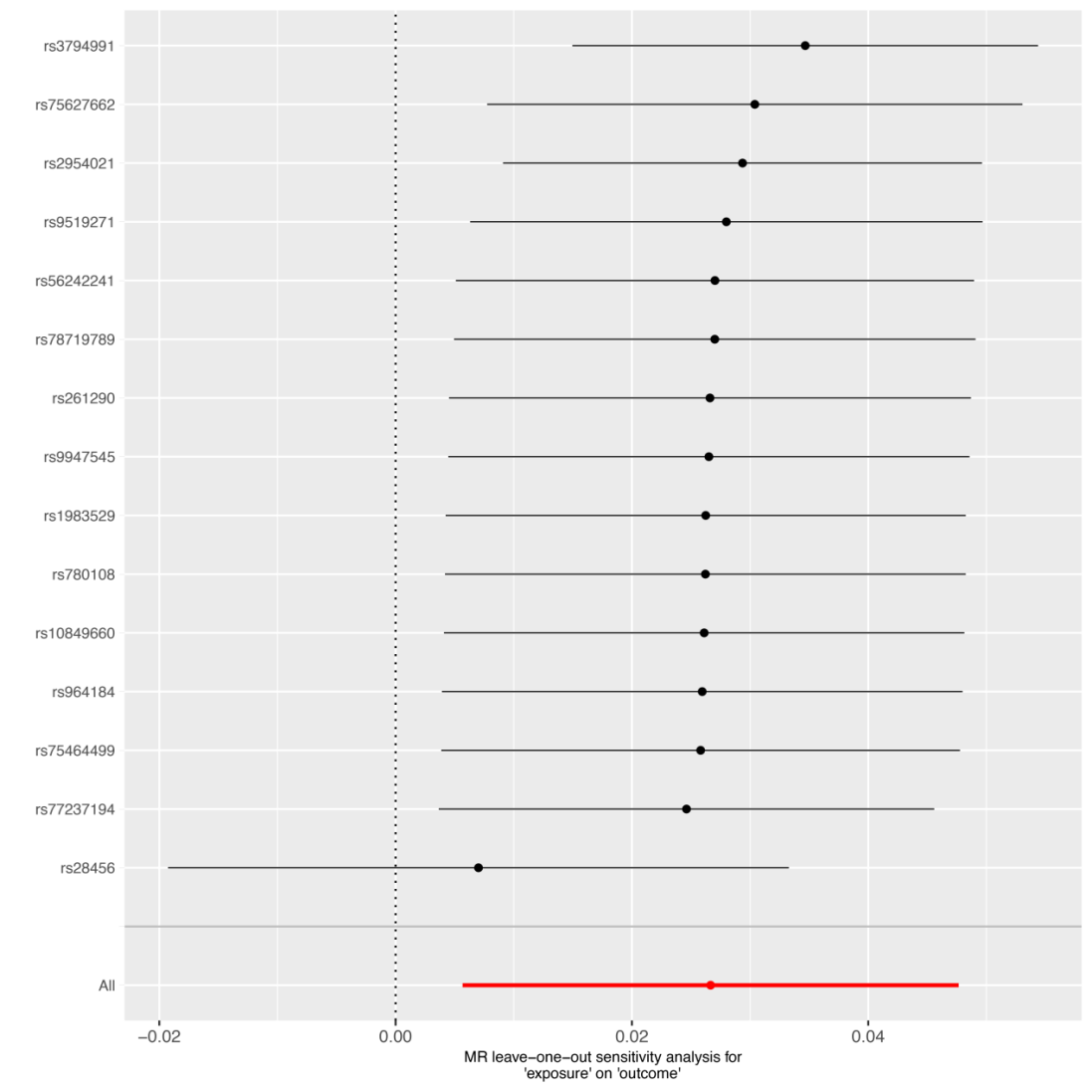


35.GCST90200114


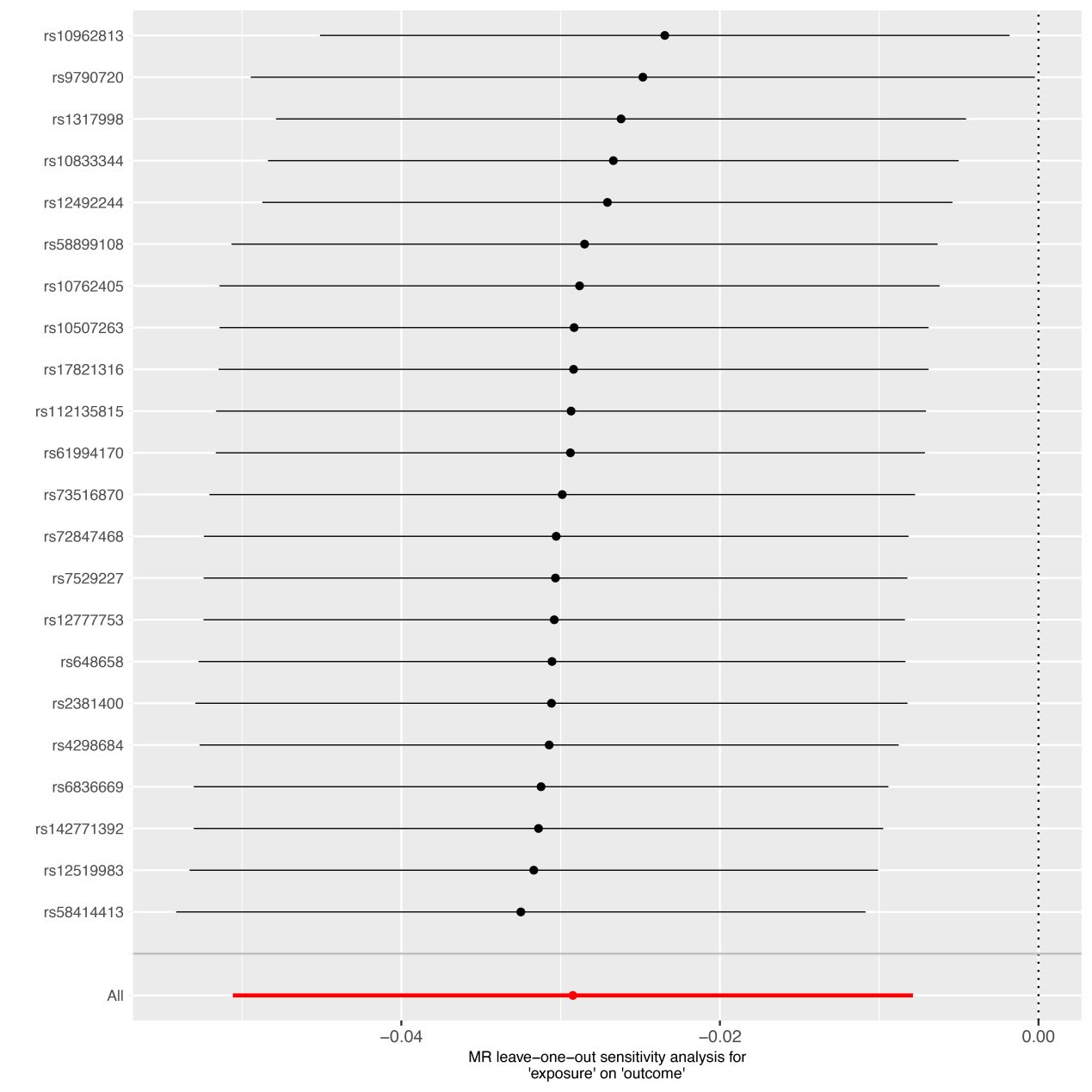


36.GCST90200136


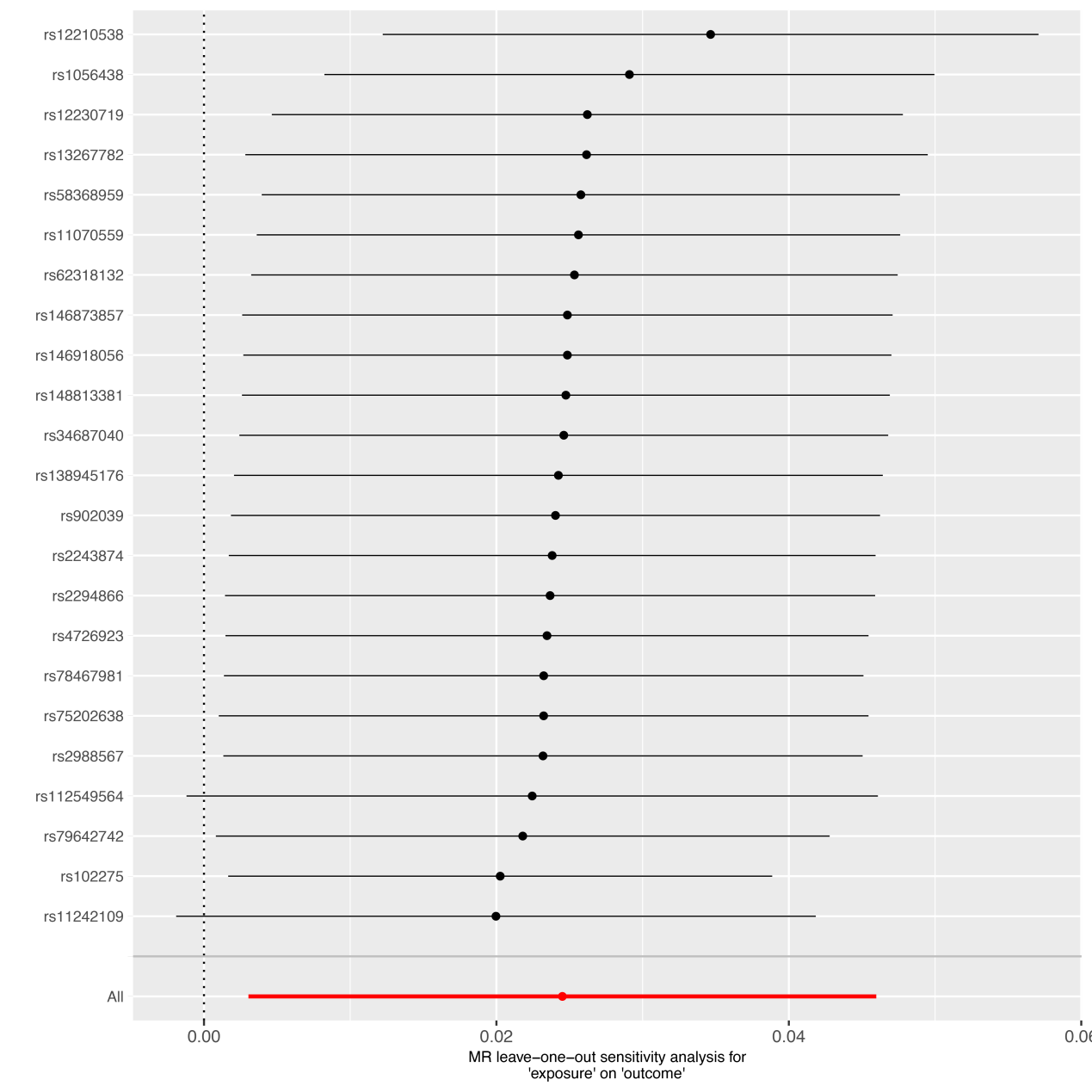


37.GCST90200186


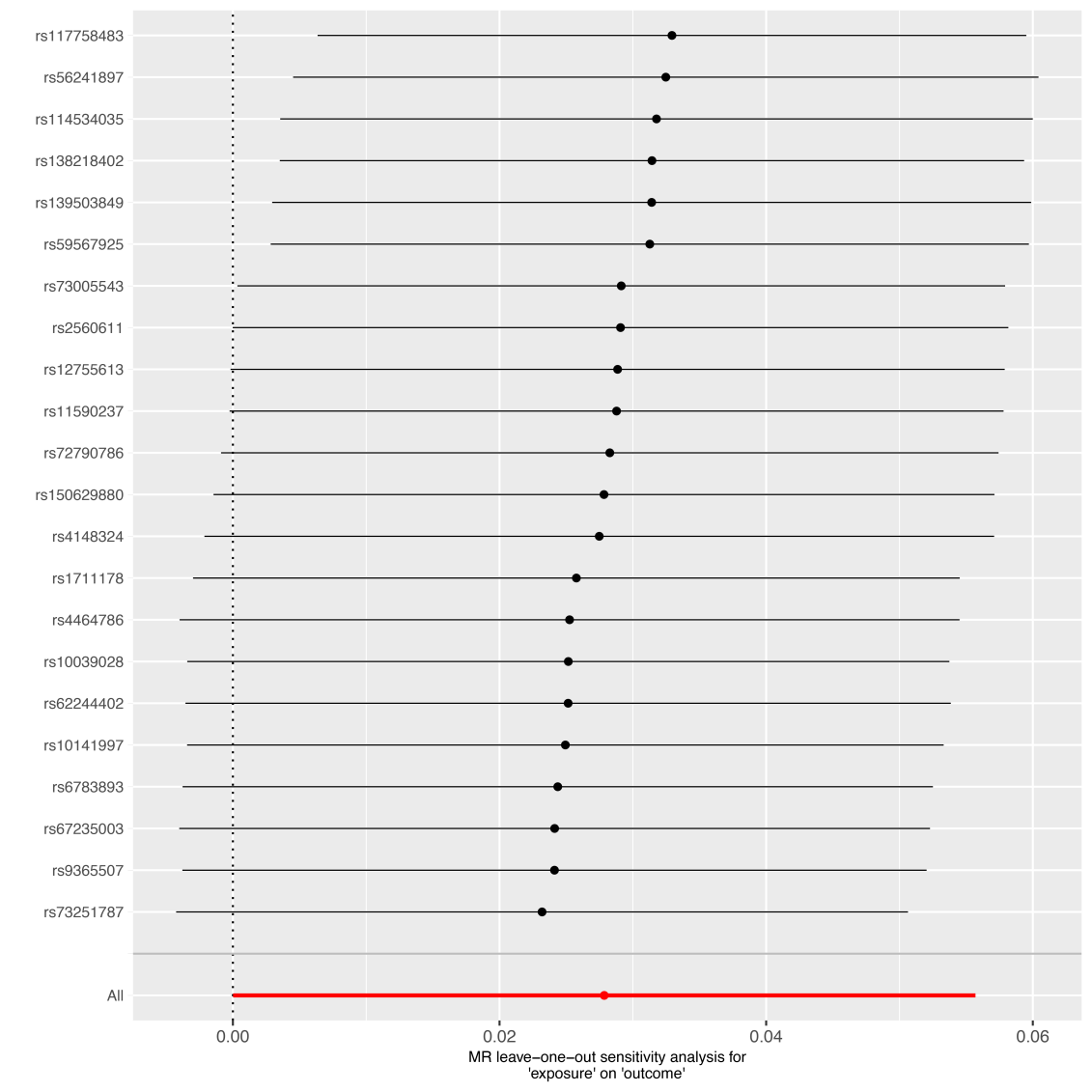


38.GCST90200213


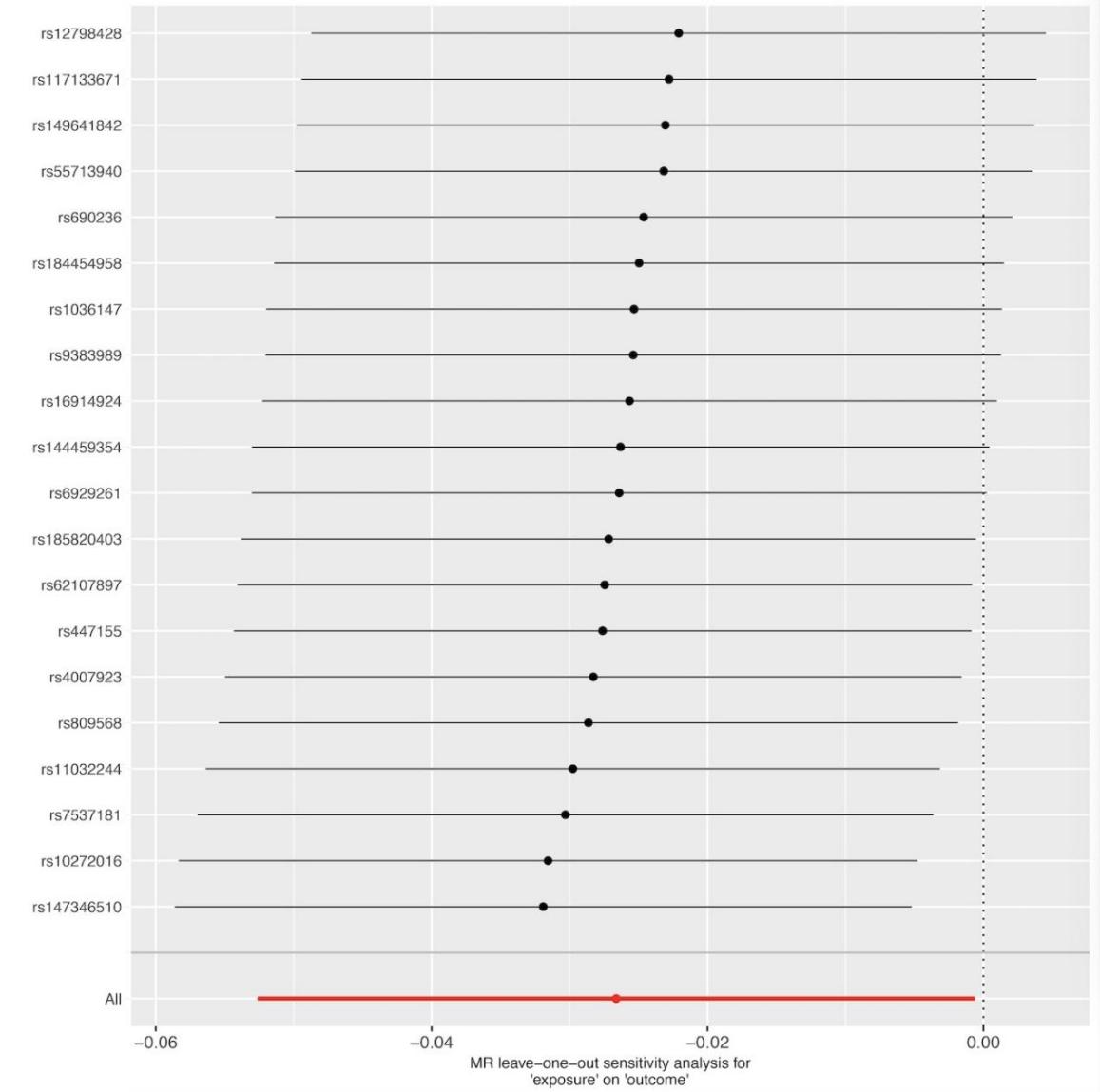


39.GCST90200268


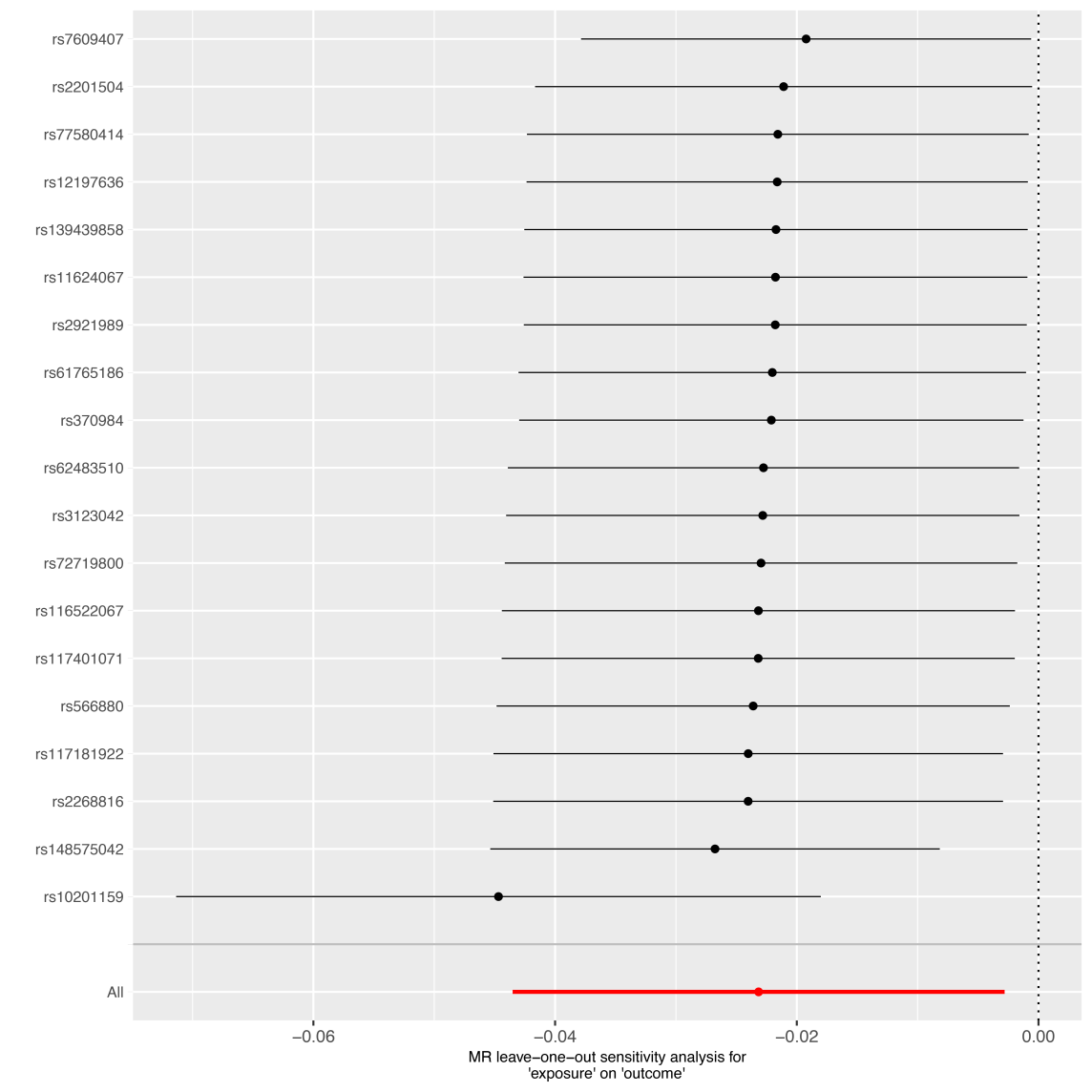


40.GCST90200280


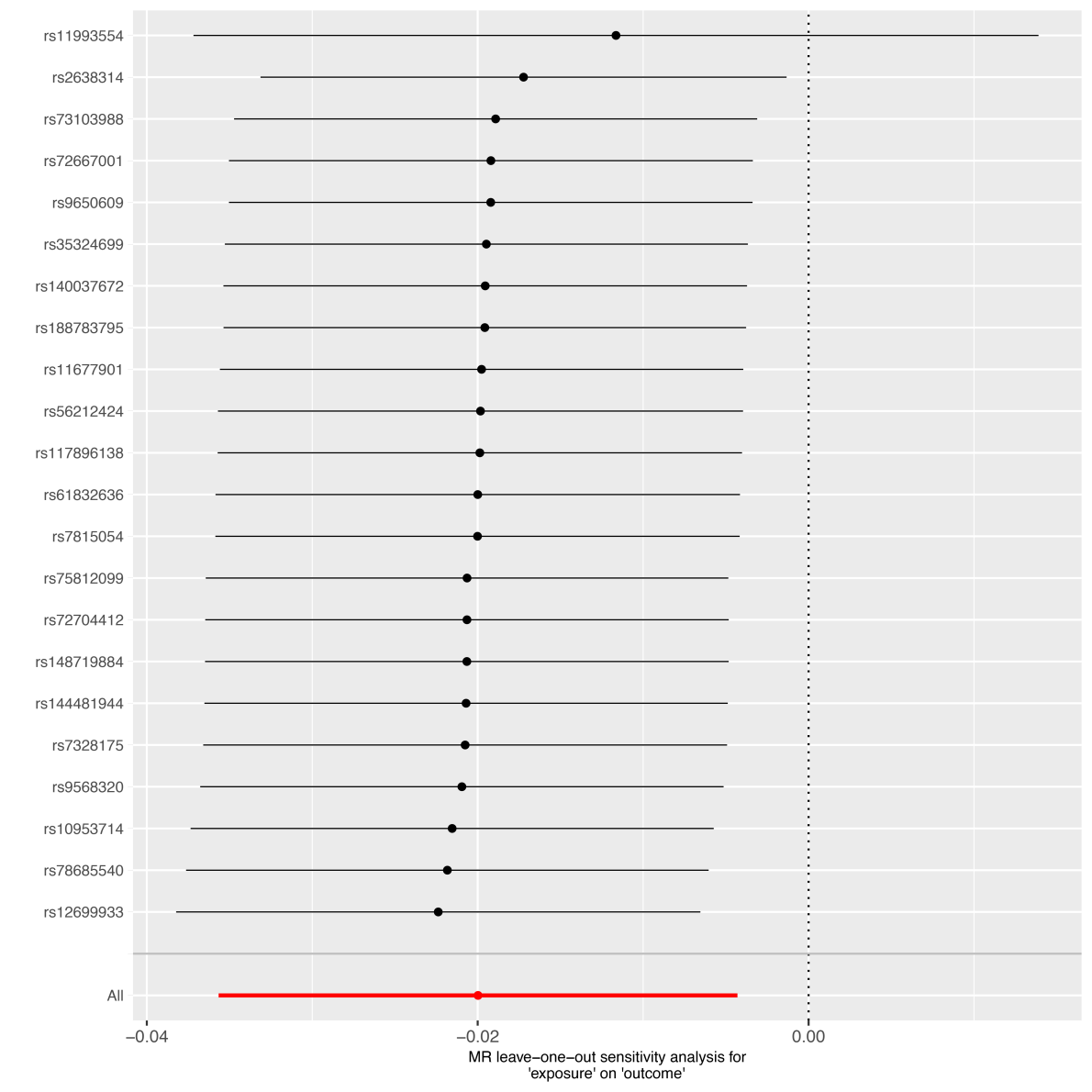


1. GCST90200295


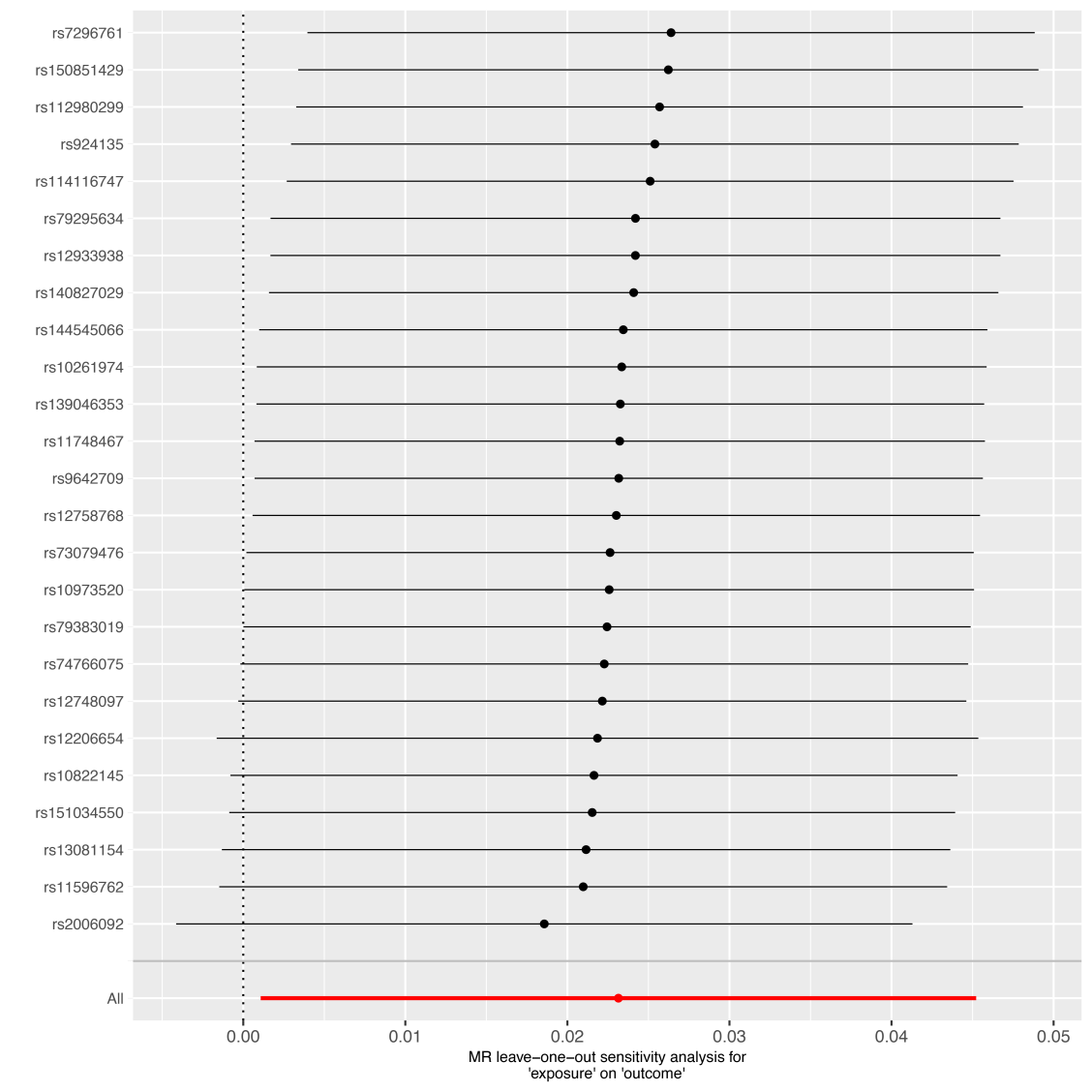


42.GCST90200323


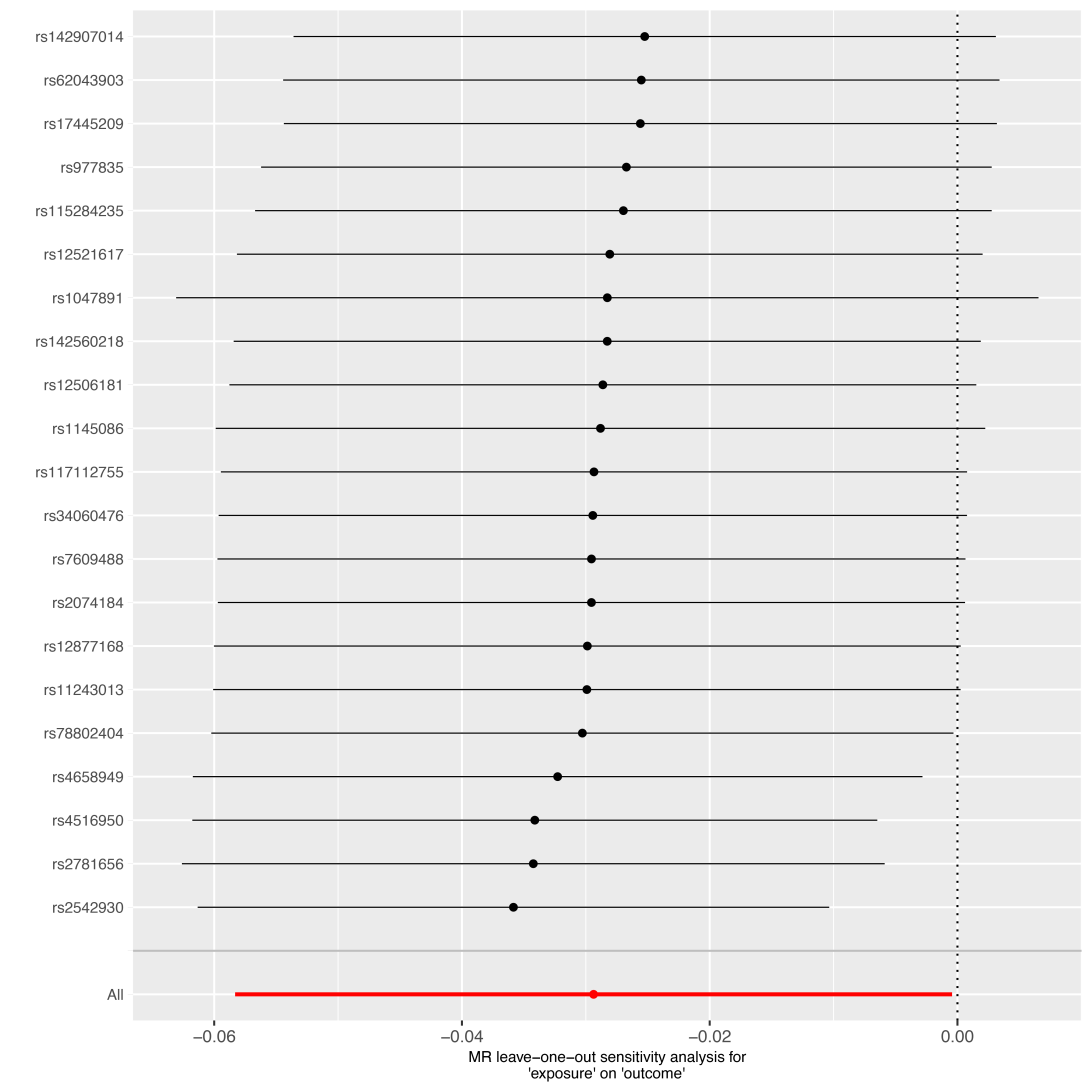


43.GCST90200334


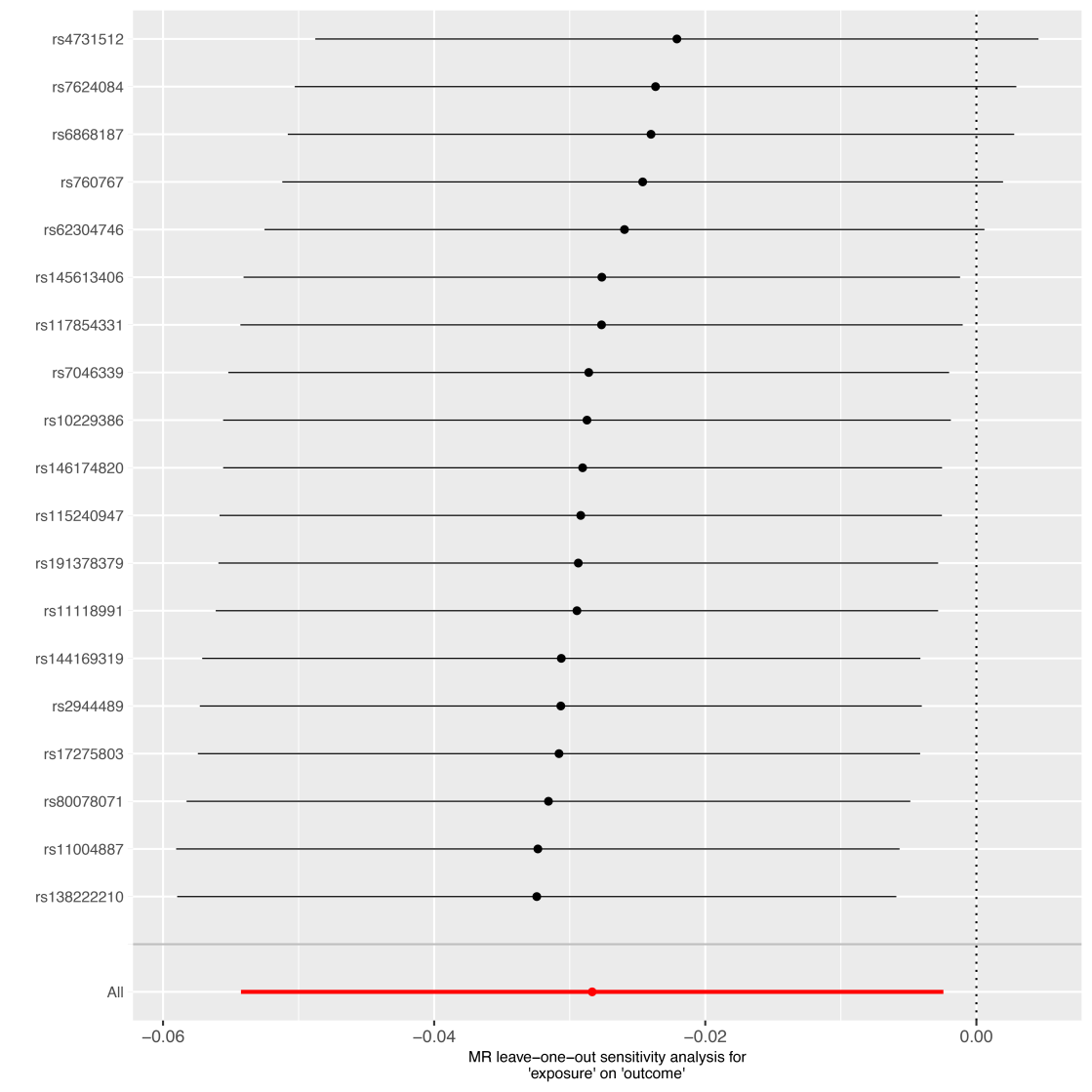


44.GCST90200352


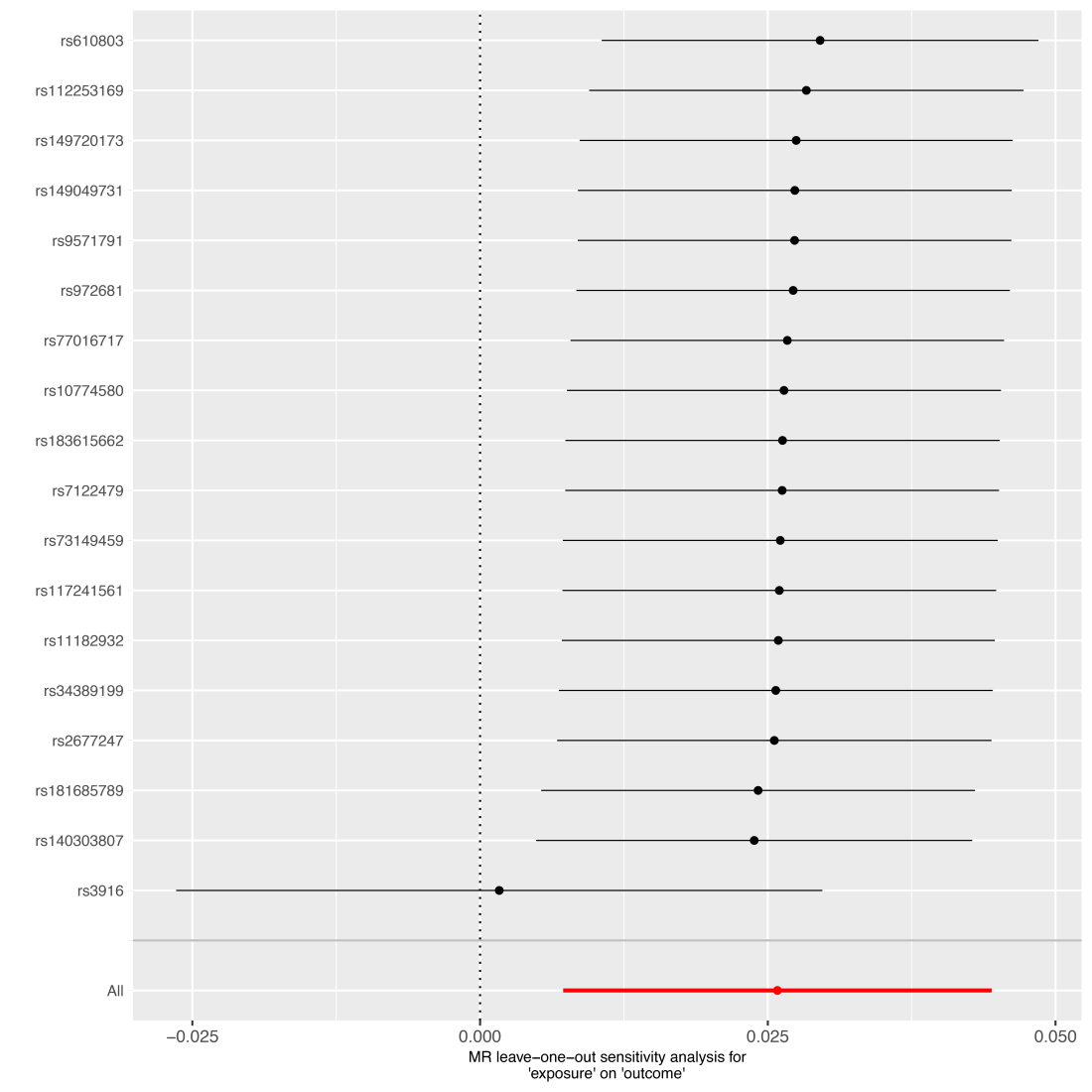


1. GCST90200355


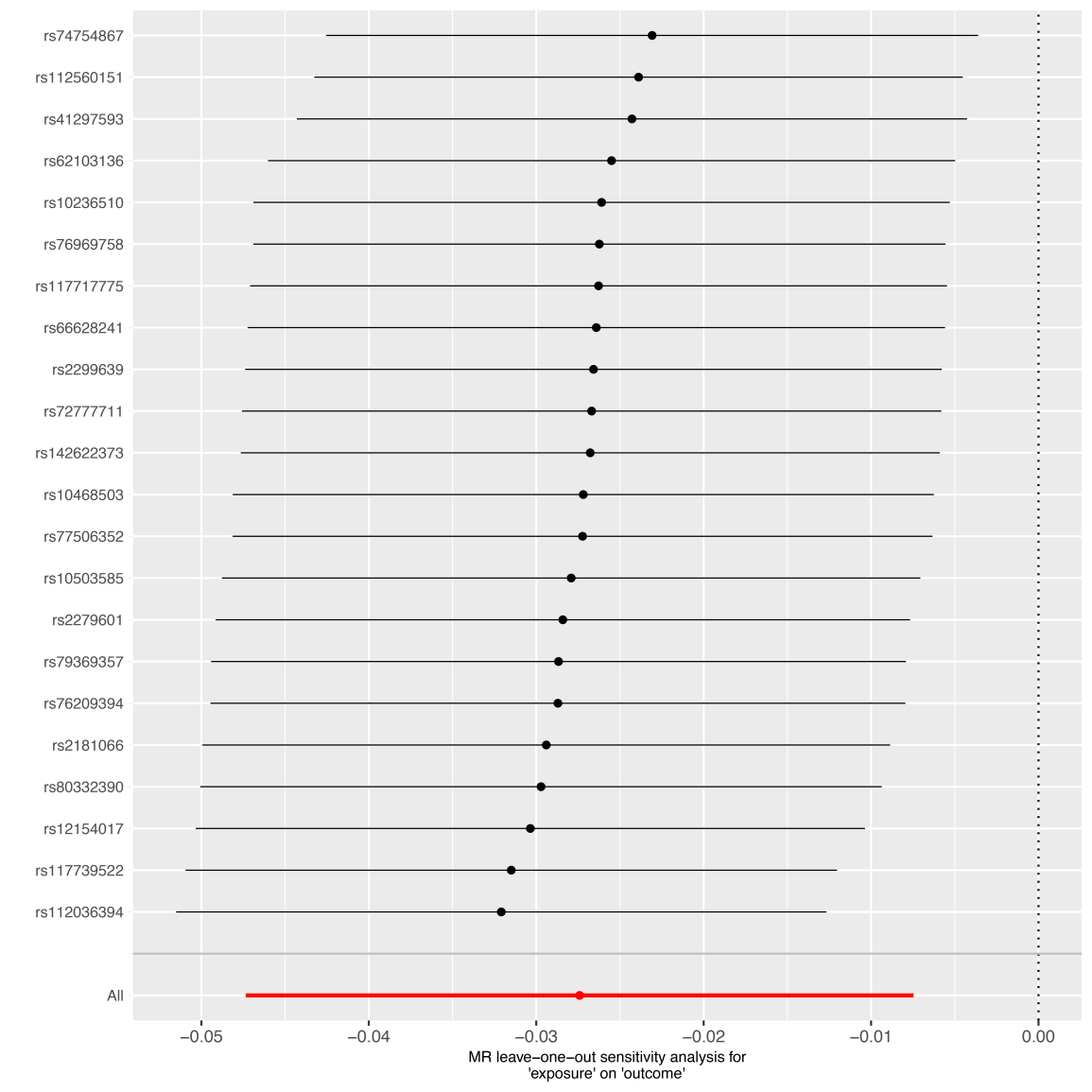


1. GCST90200358


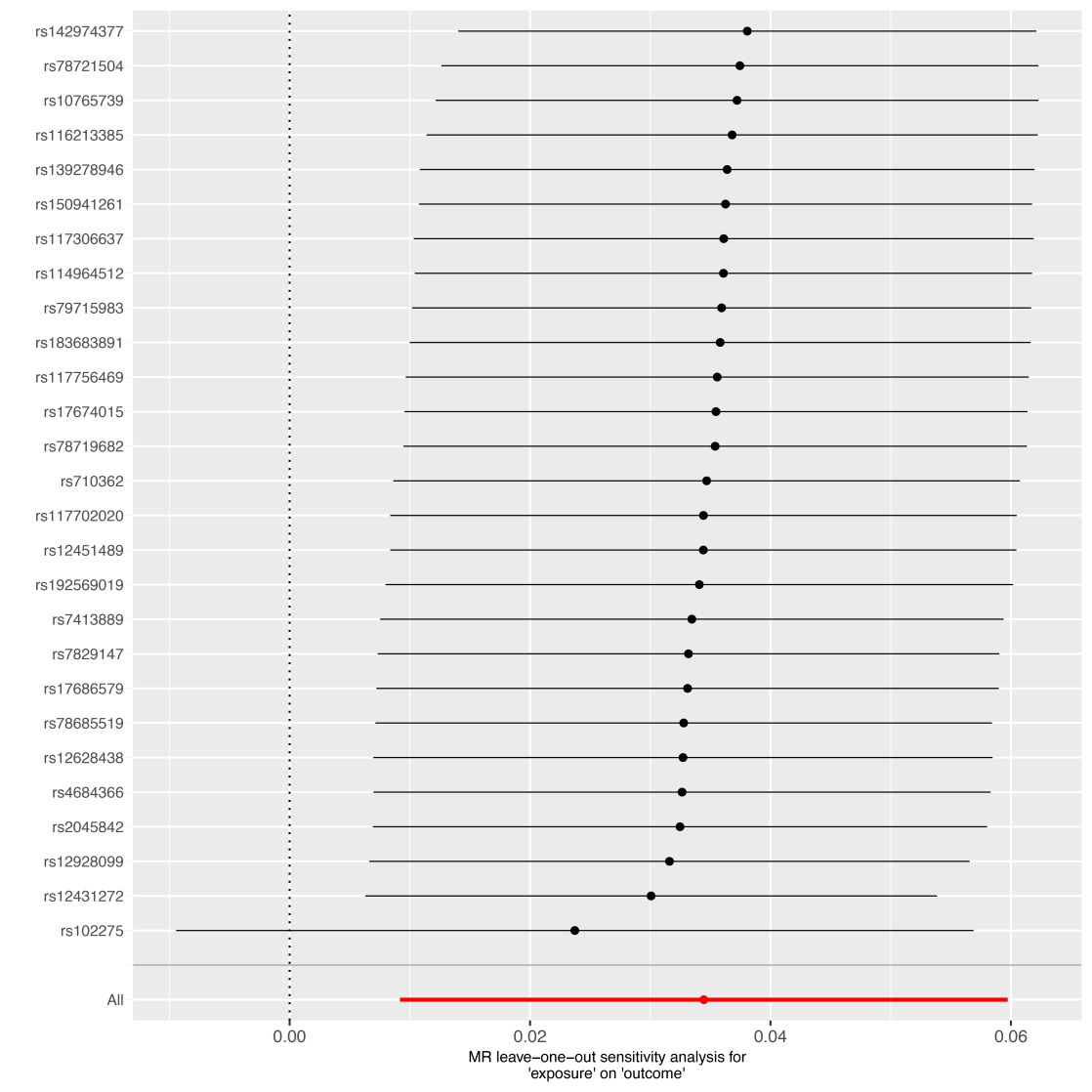


1. GCST90200368


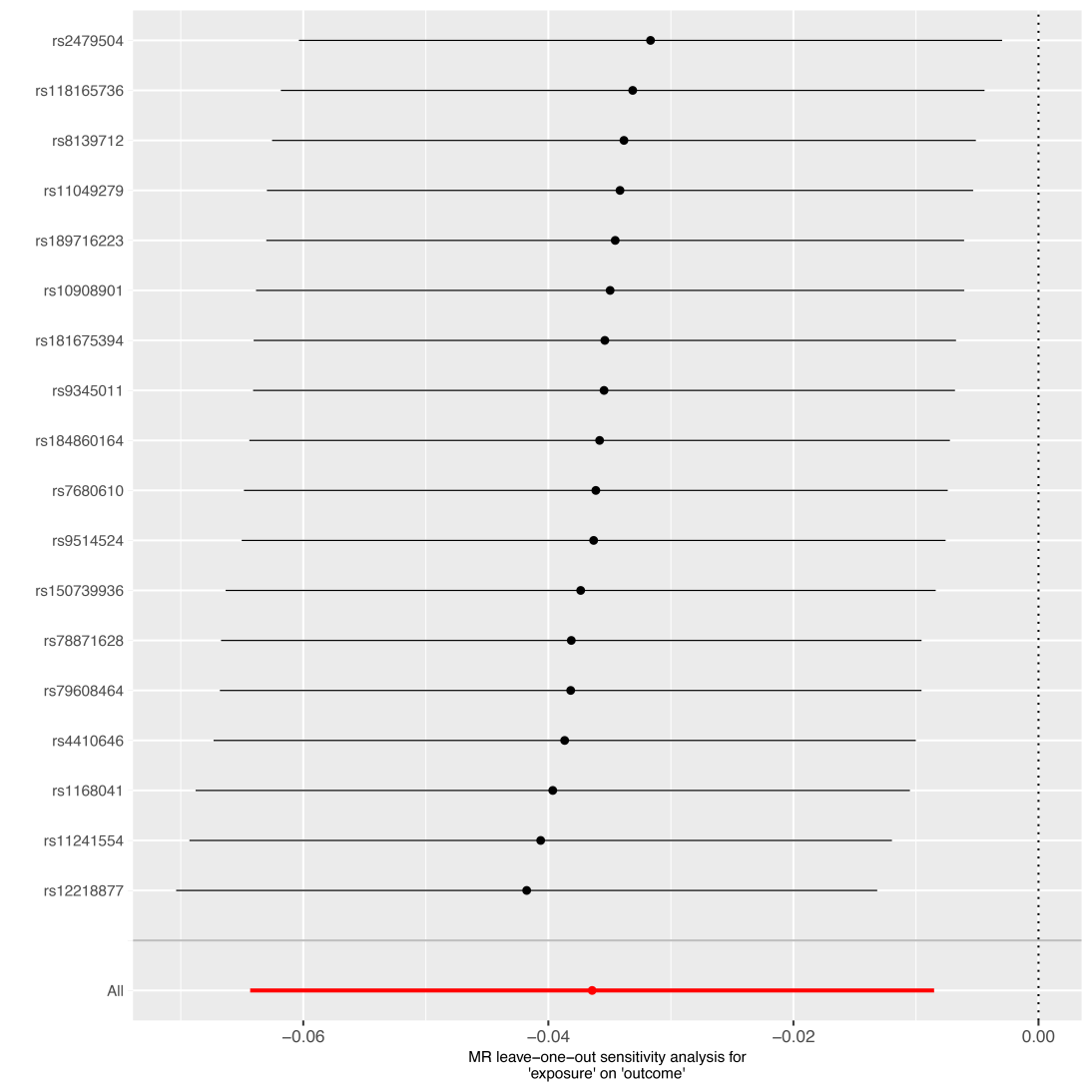


48.GCST90200419


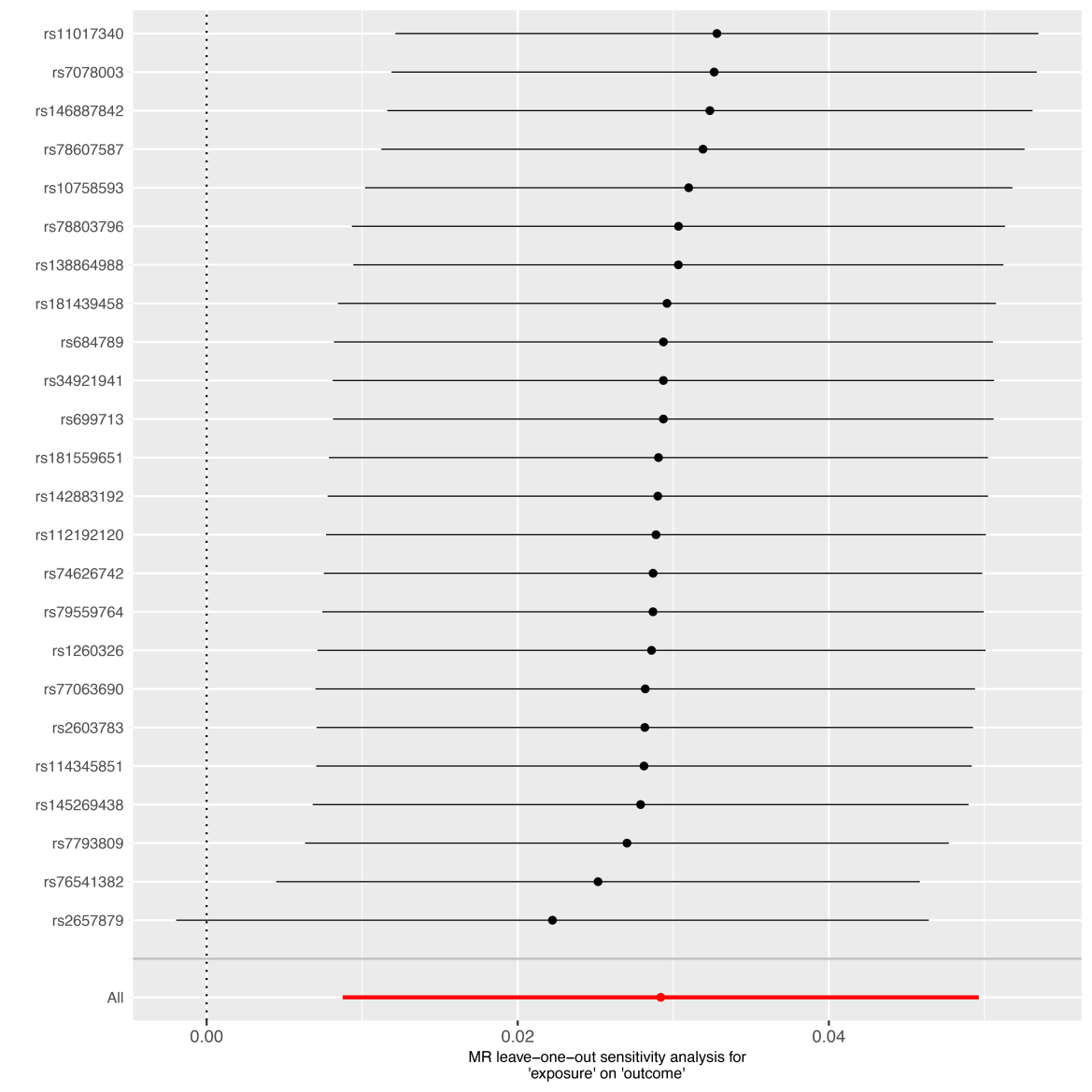


49.GCST90200438


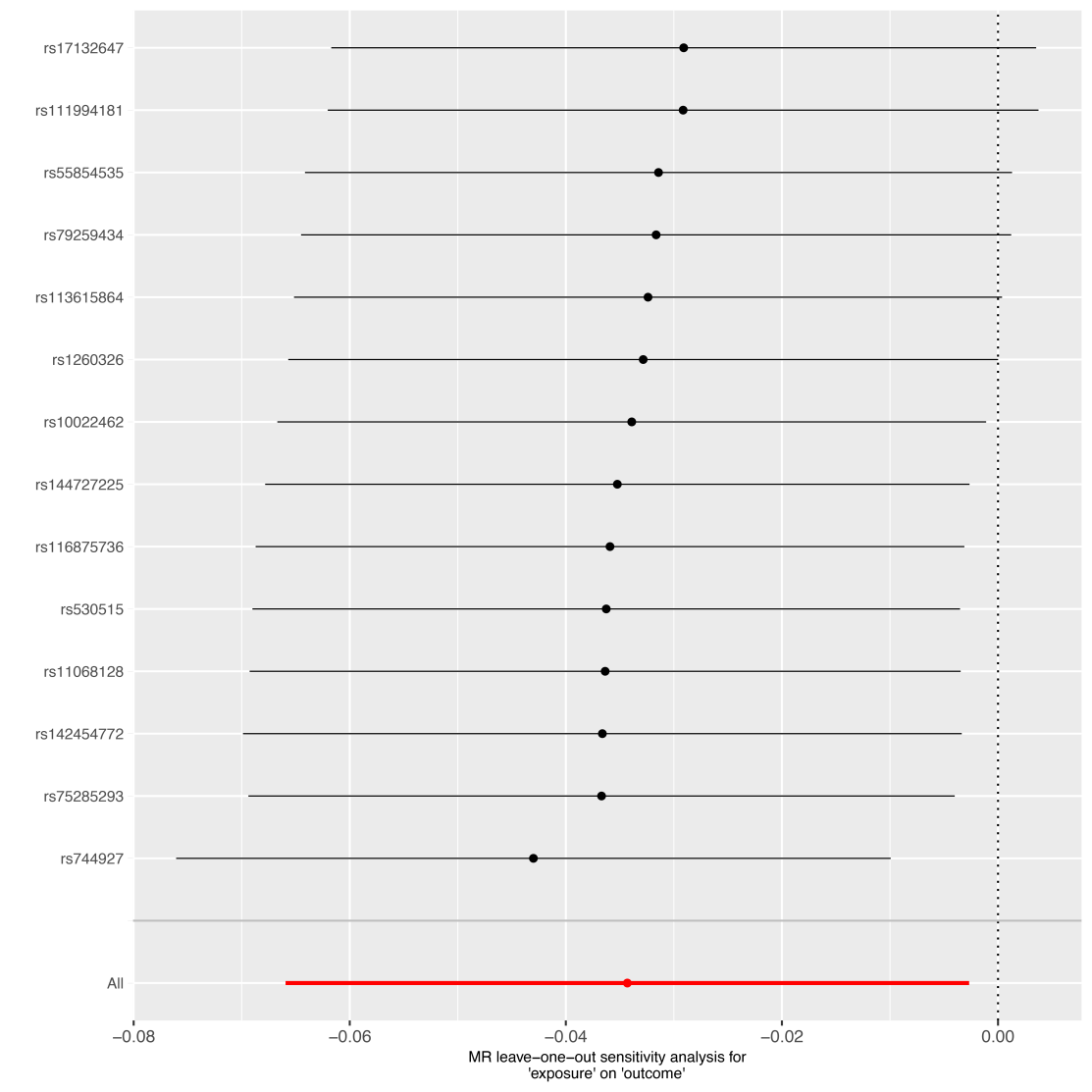


50.GCST90200447


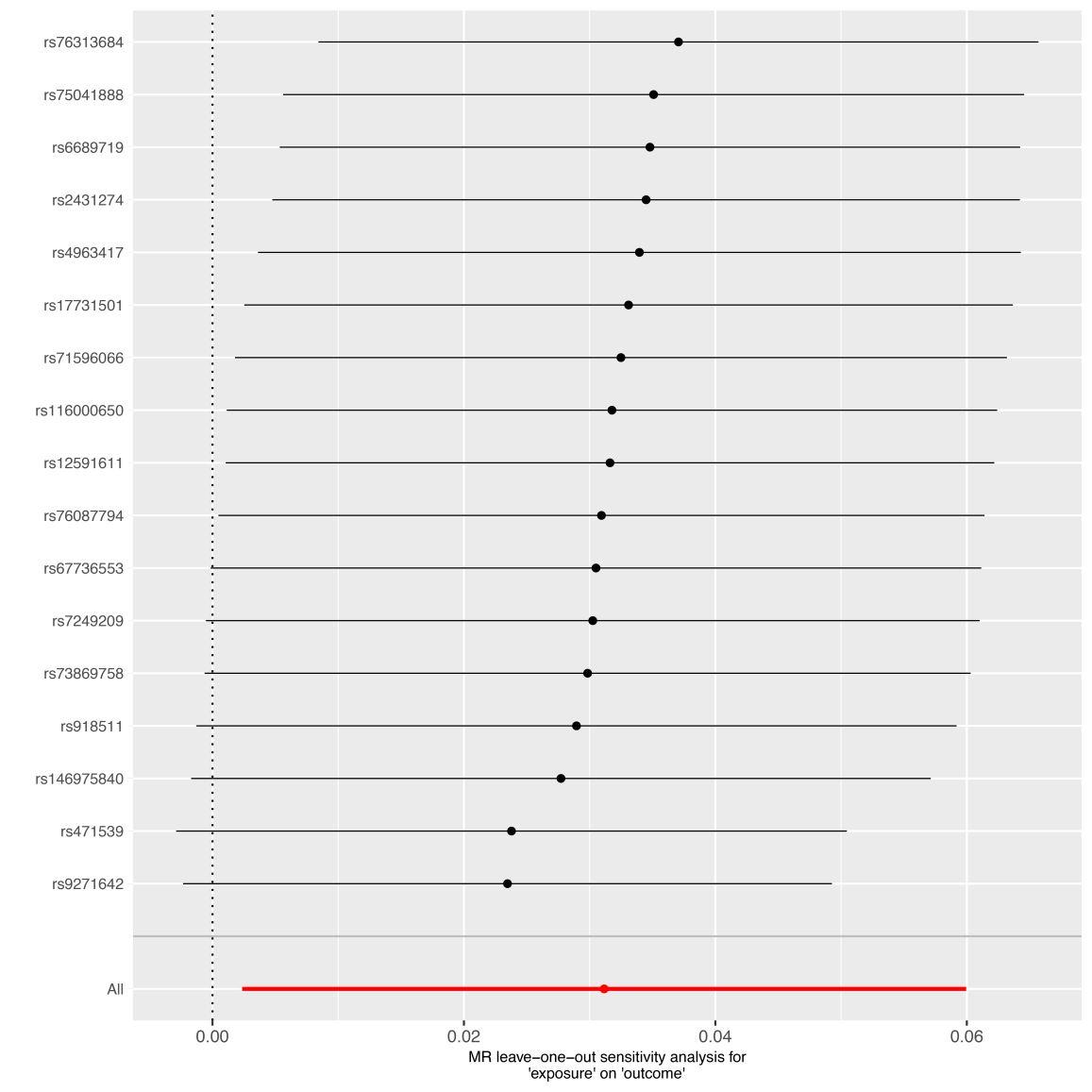


51.GCST90200464


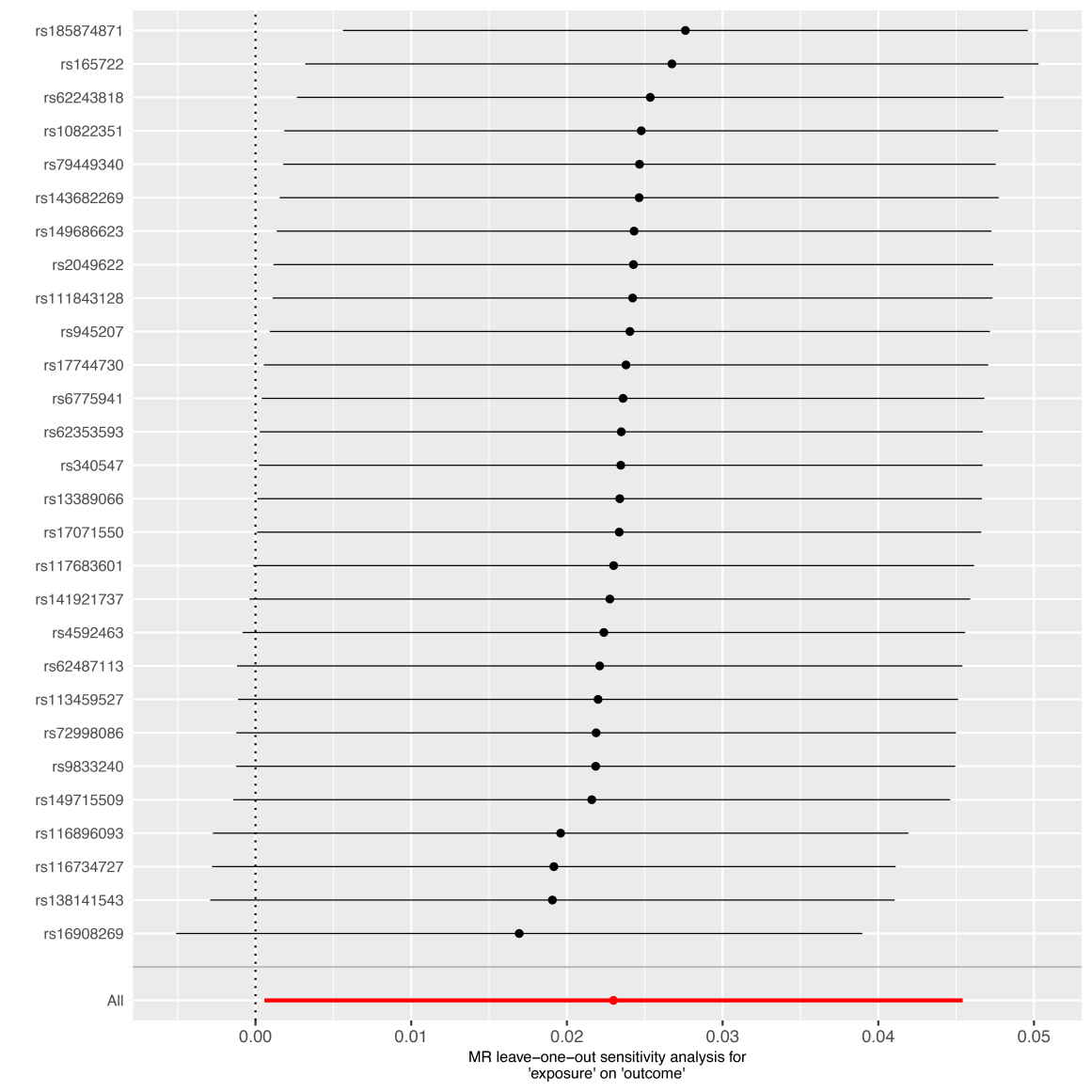


52.GCST90200470


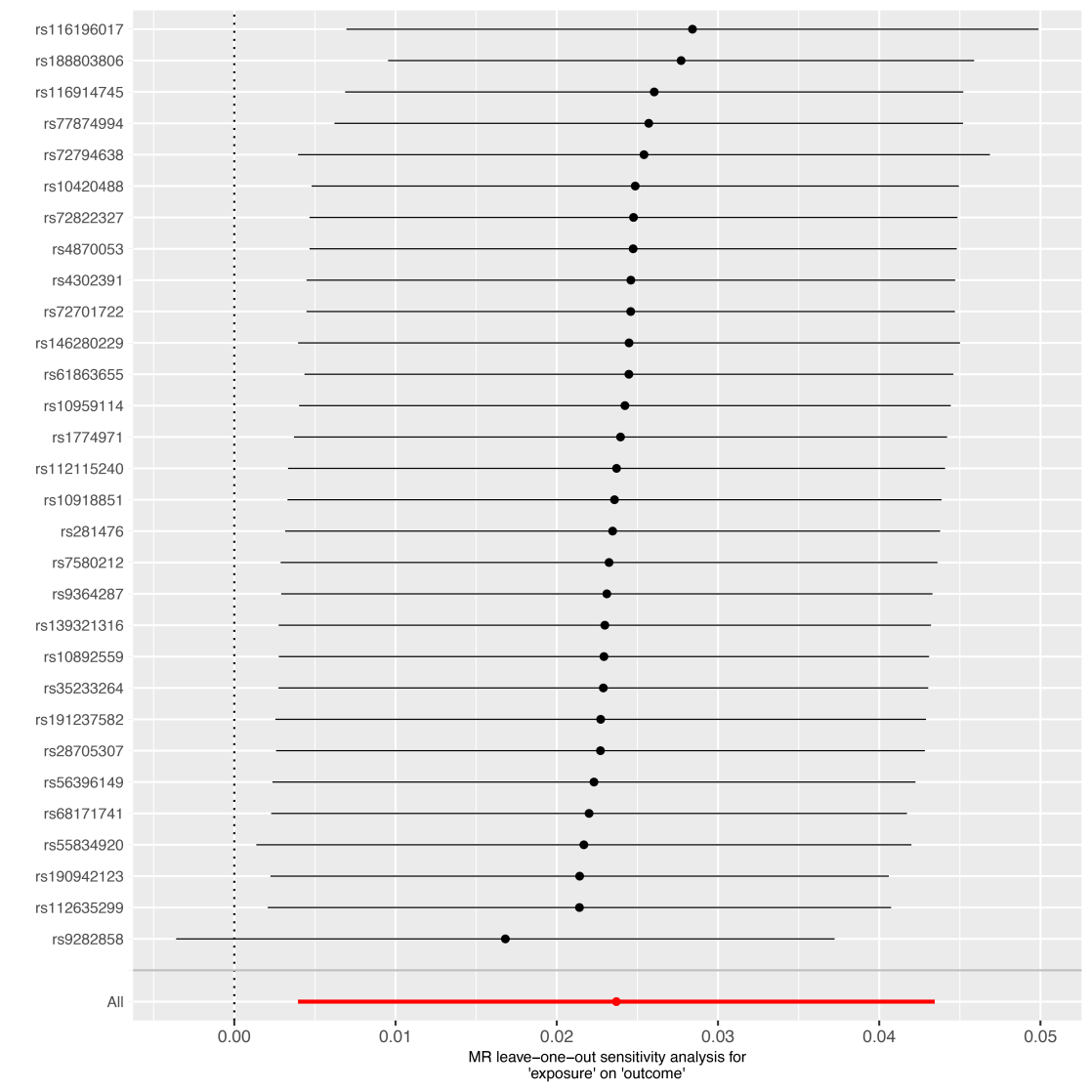


53.GCST90200474


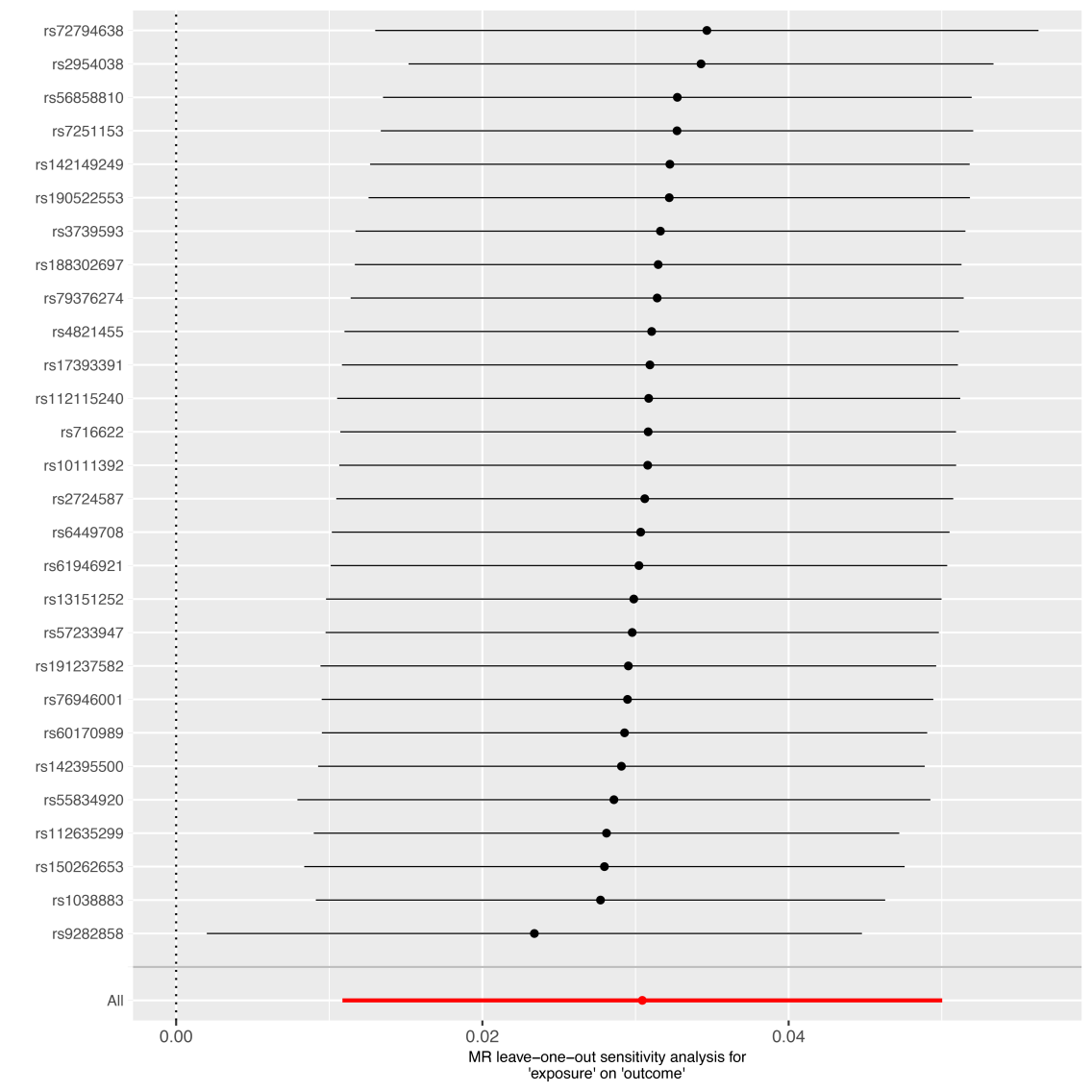


54.GCST90200480


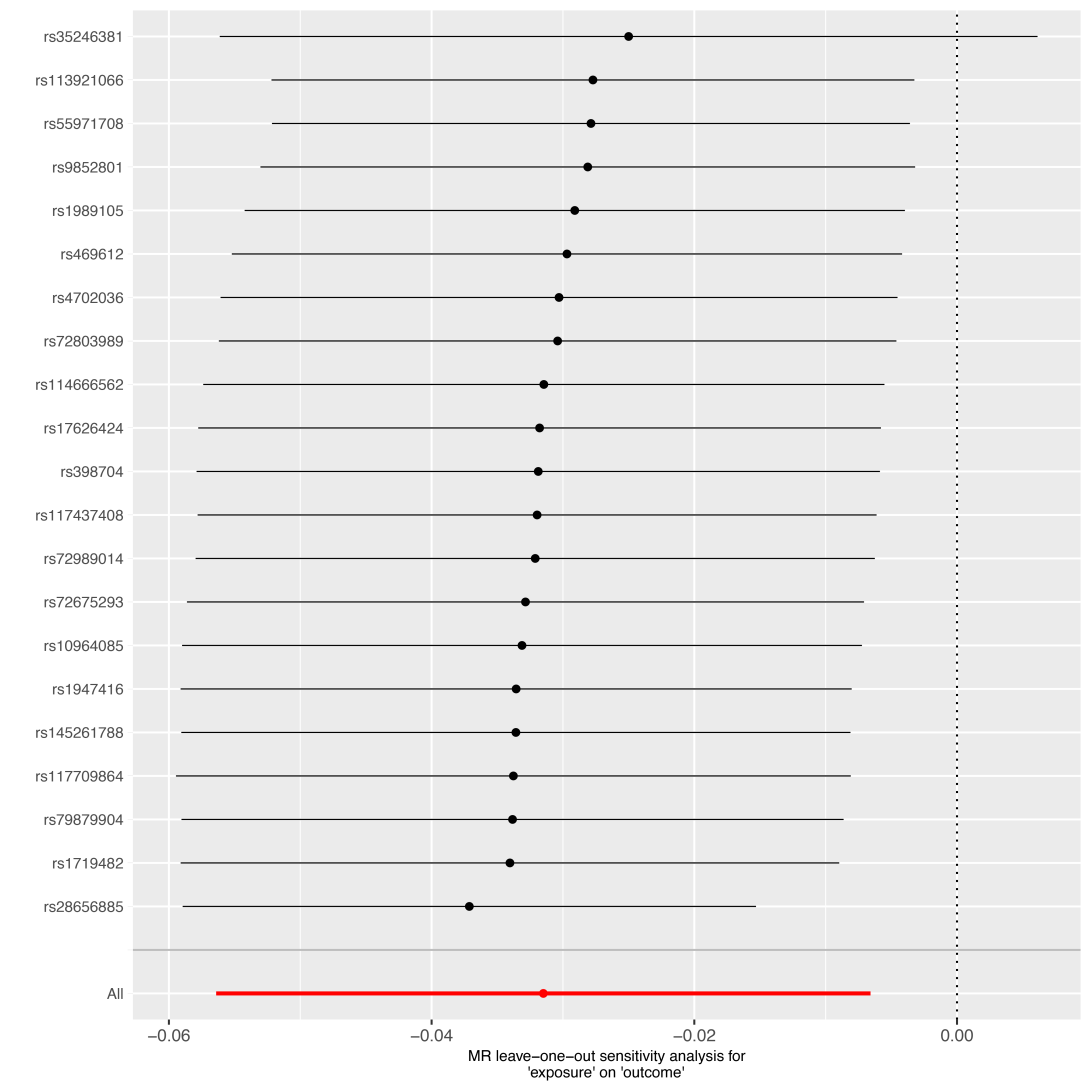


55.GCST90200497


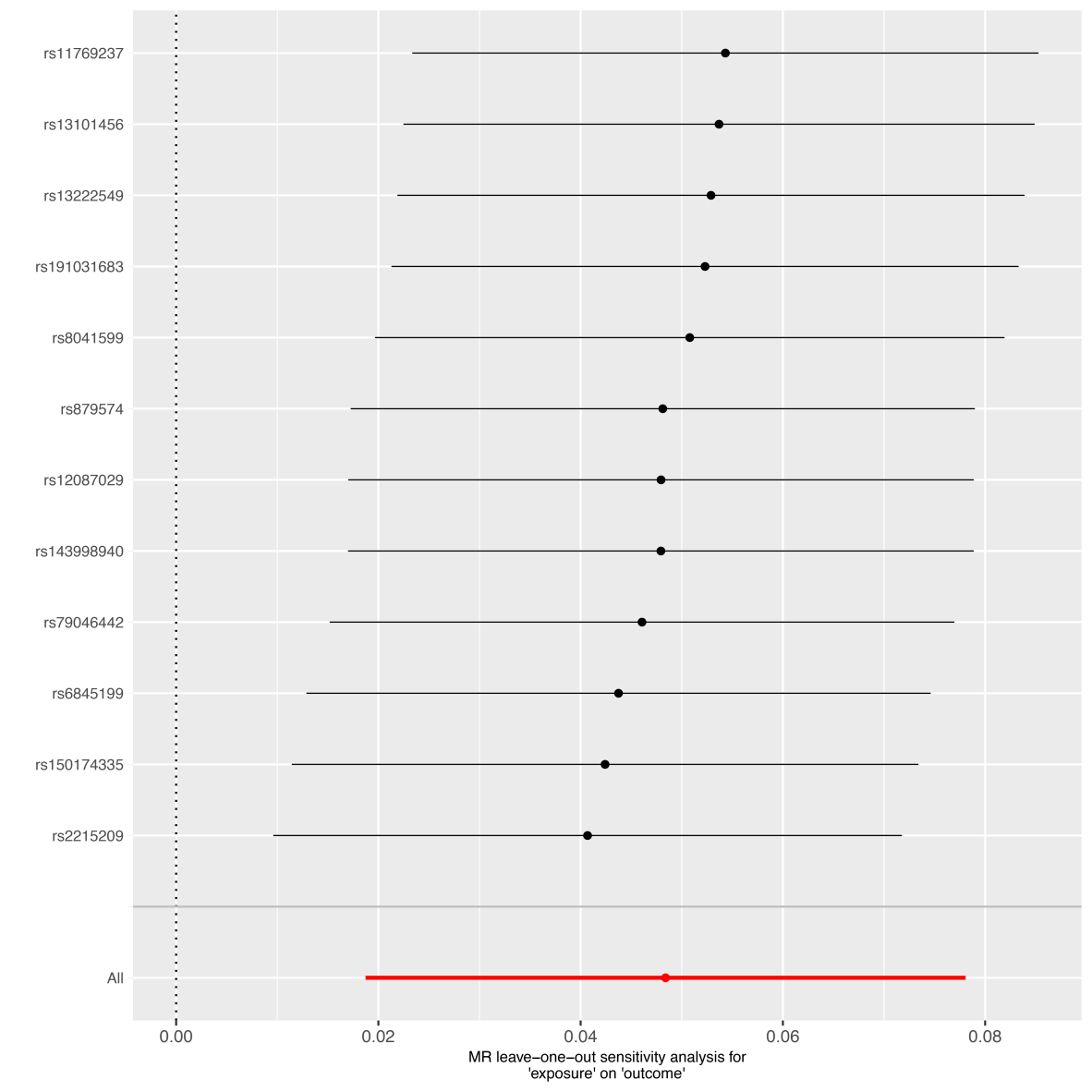


56.GCST90200522


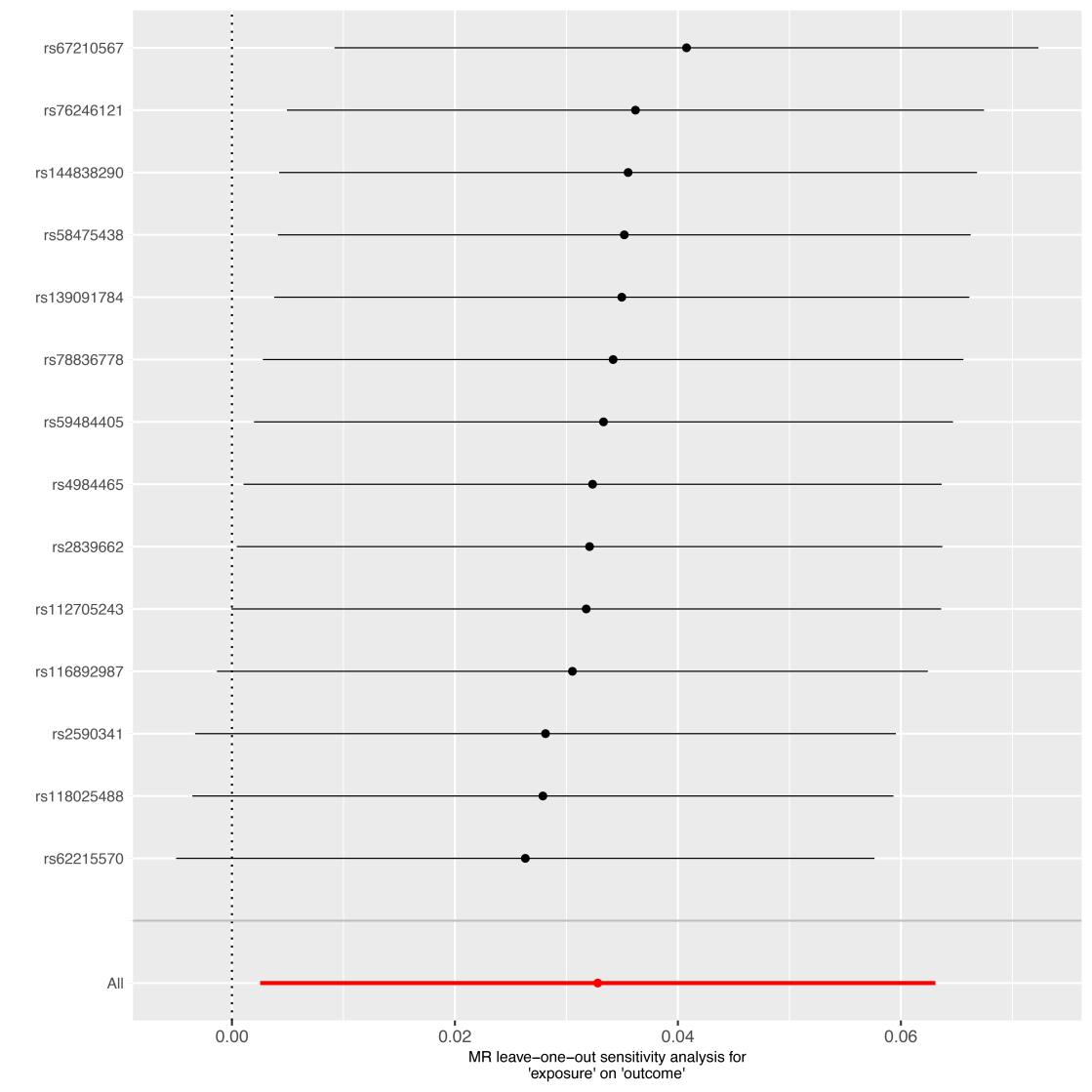


57.GCST90200544


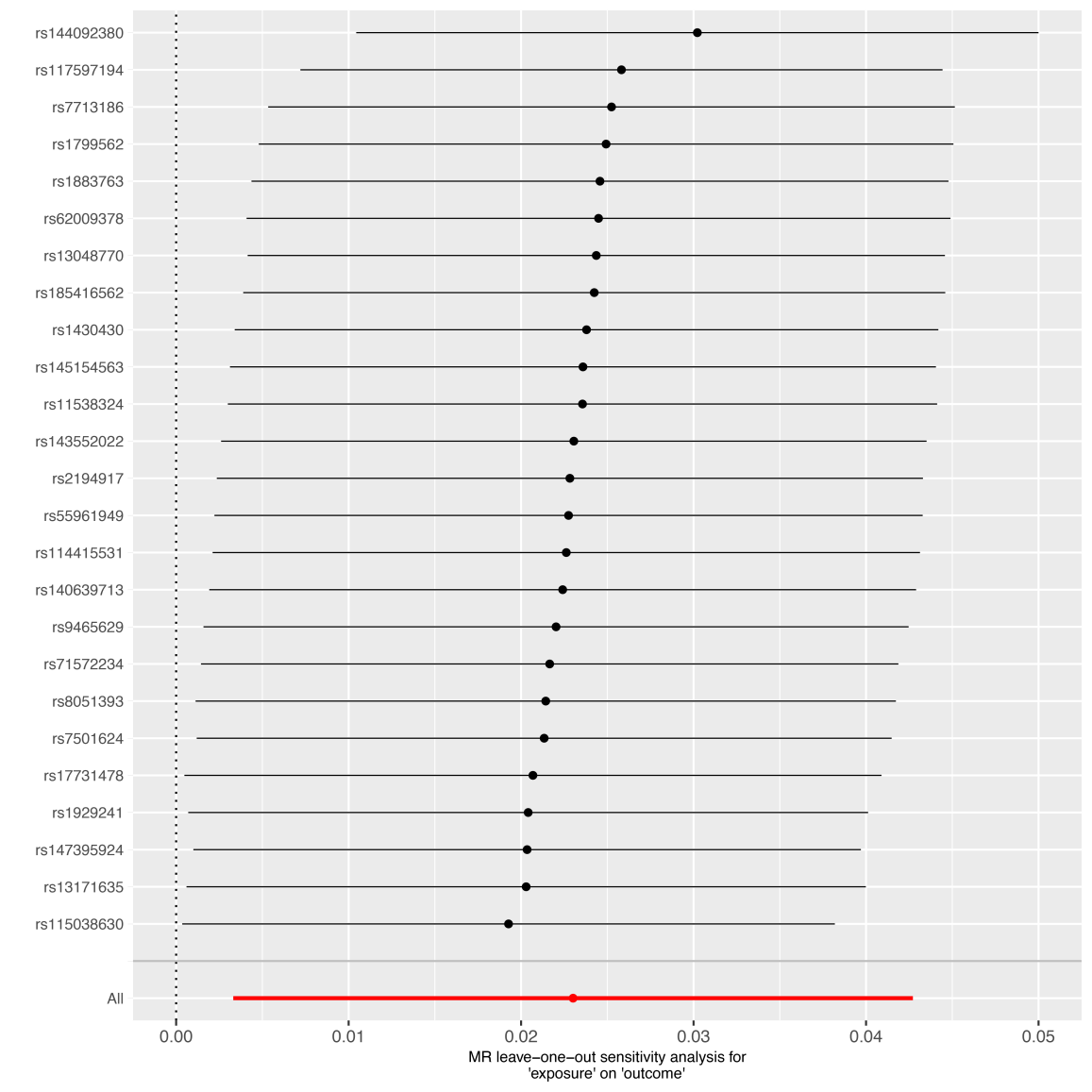


58.GCST90200559


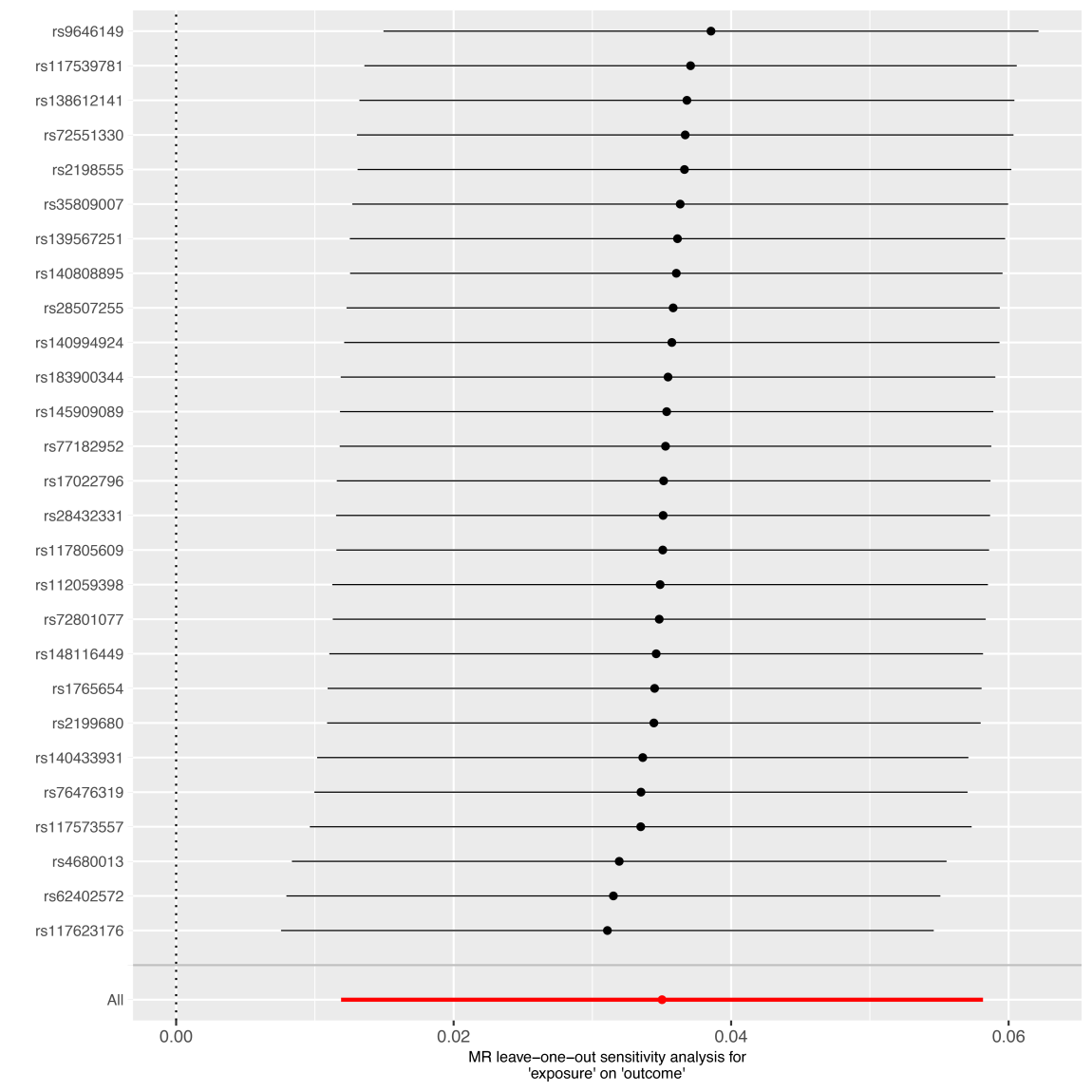


59.GCST90200562


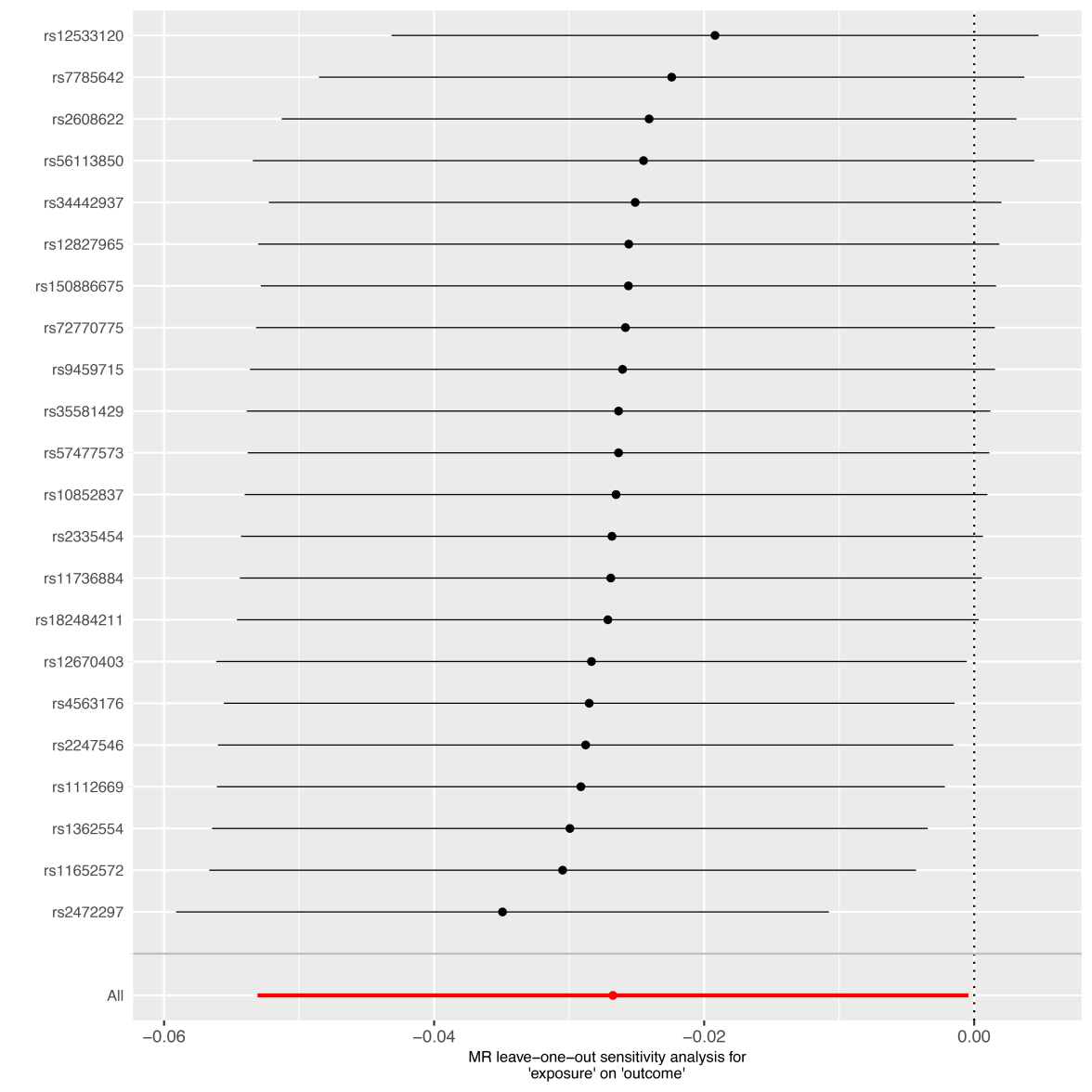


60.GCST90200570


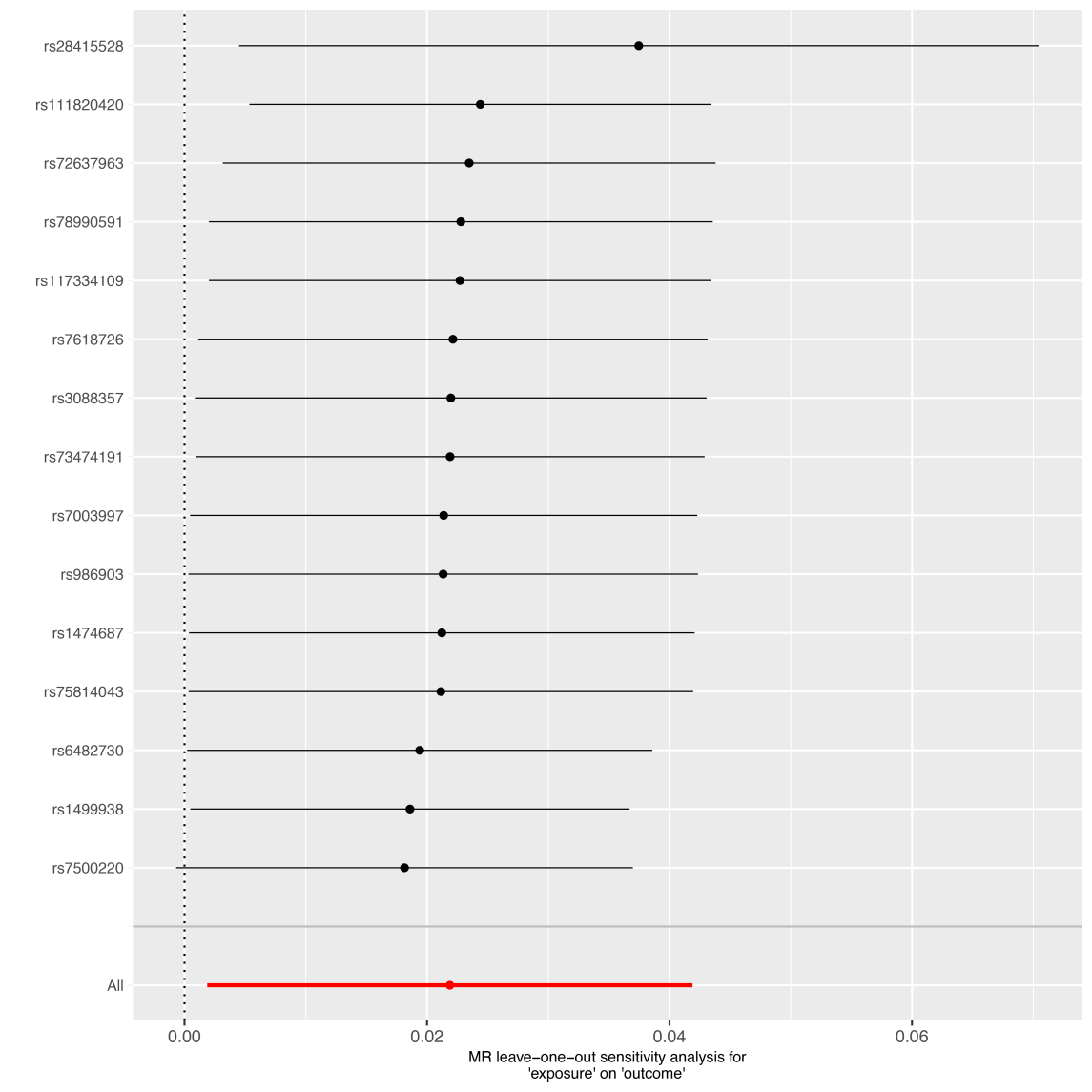


61.GCST90200573


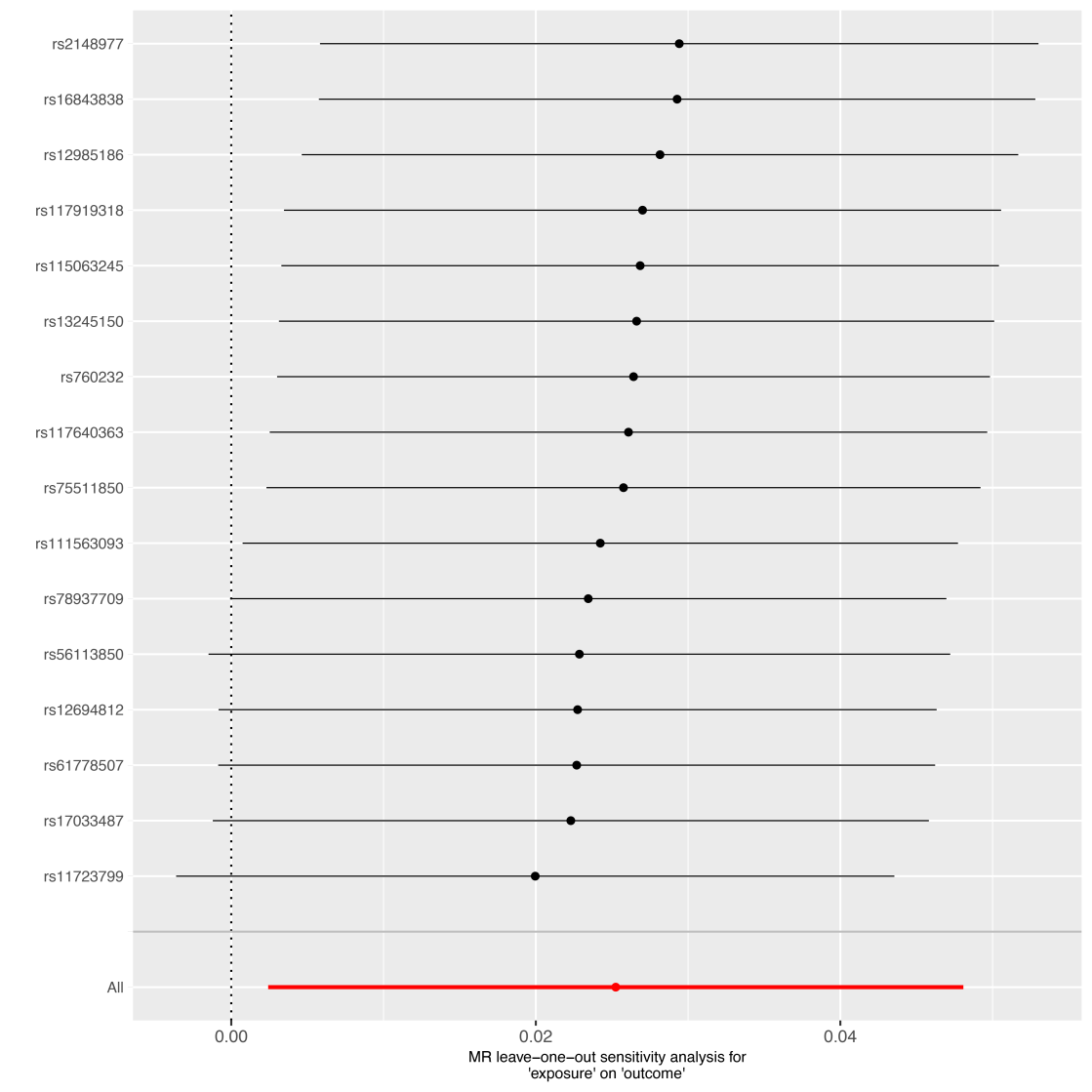


62.GCST90200575


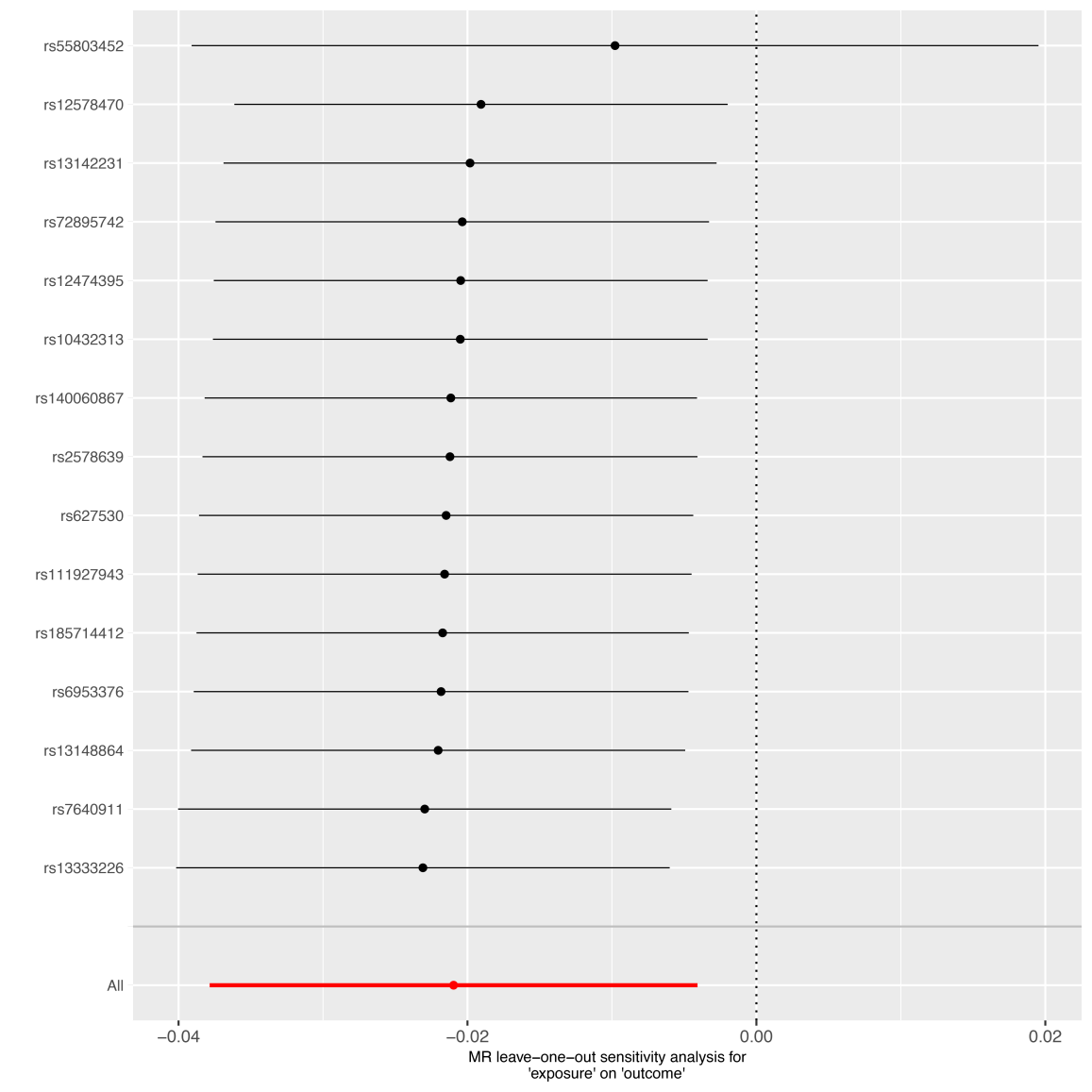


63.GCST90200628


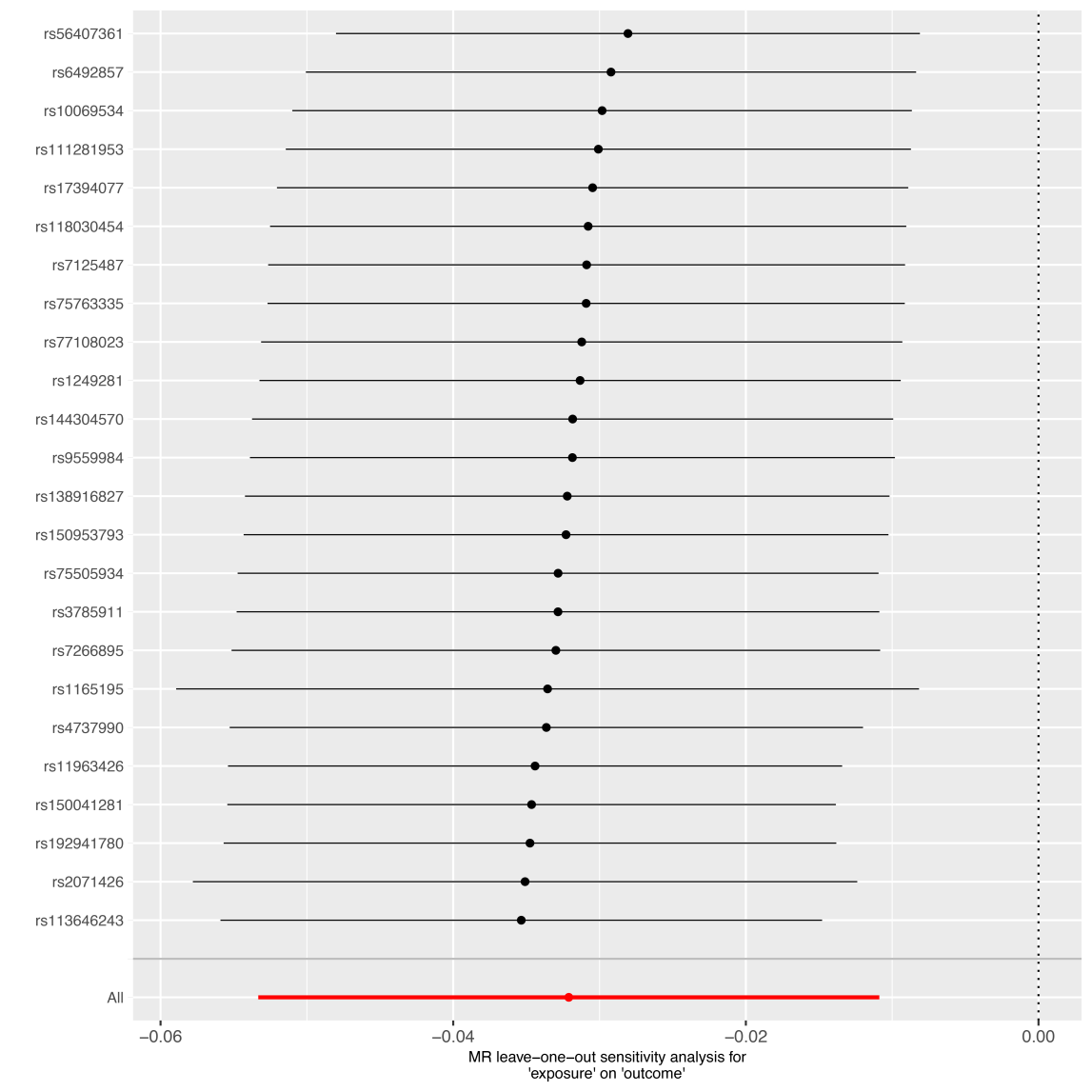


64.GCST90200632


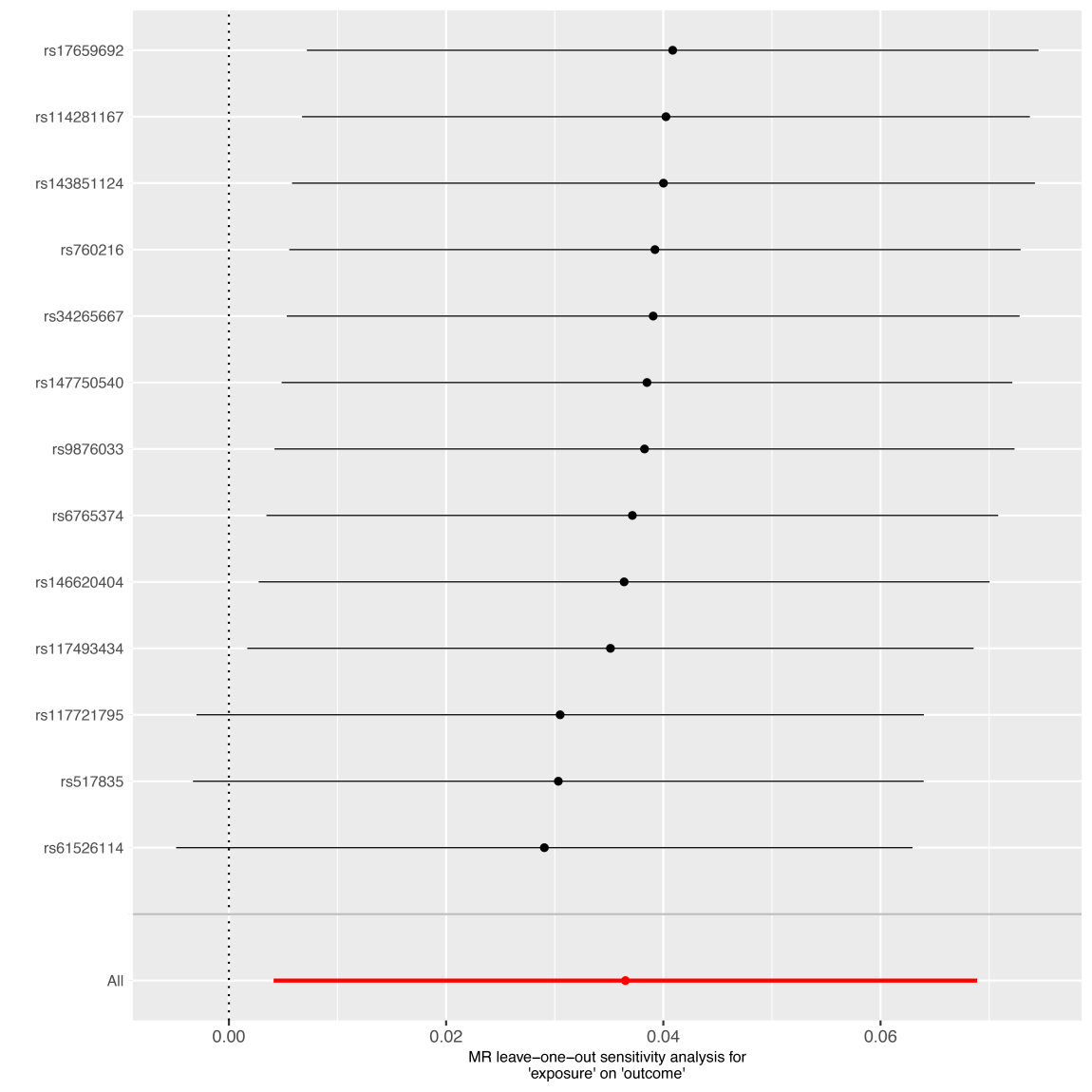


65.GCST90200643


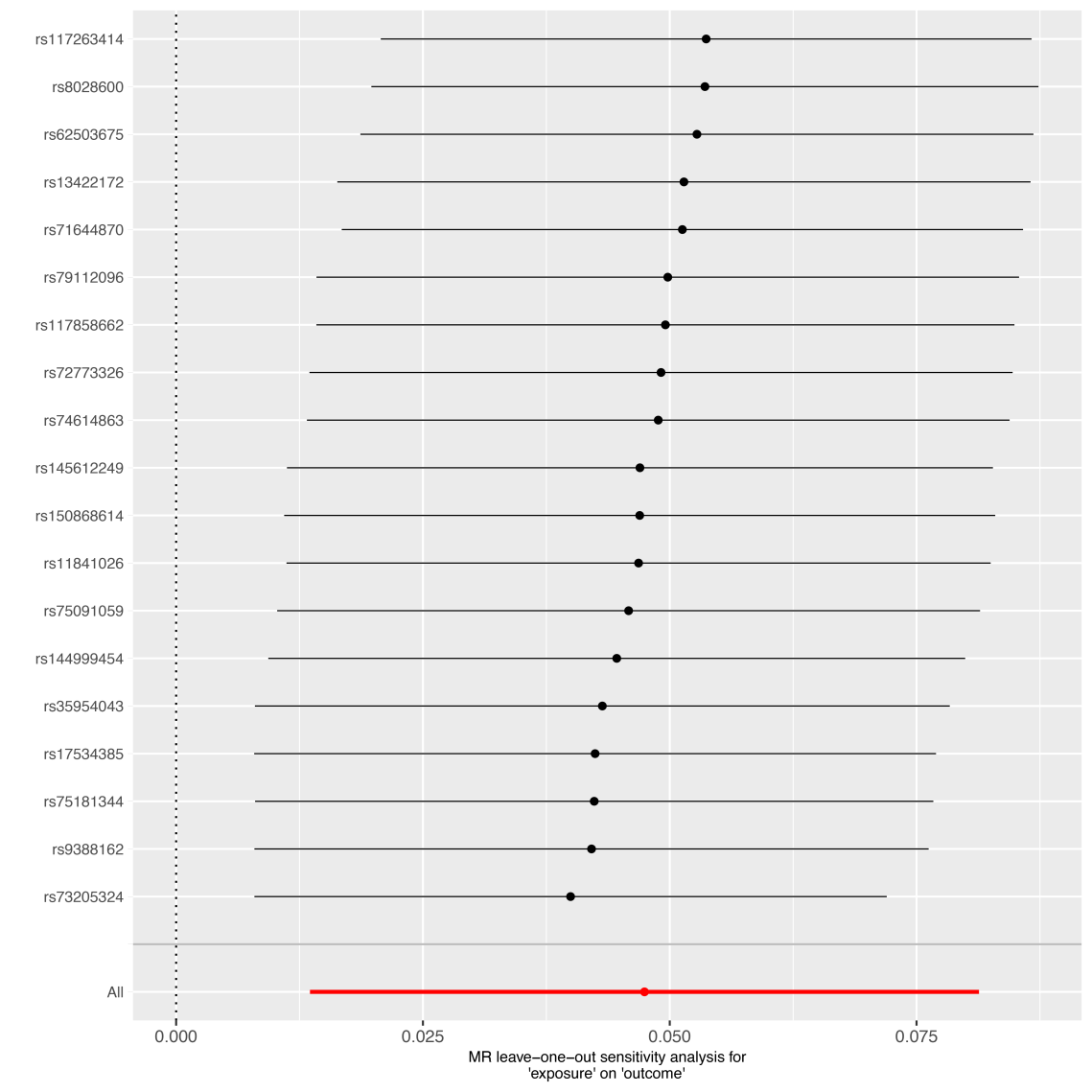


66.GCST90200644


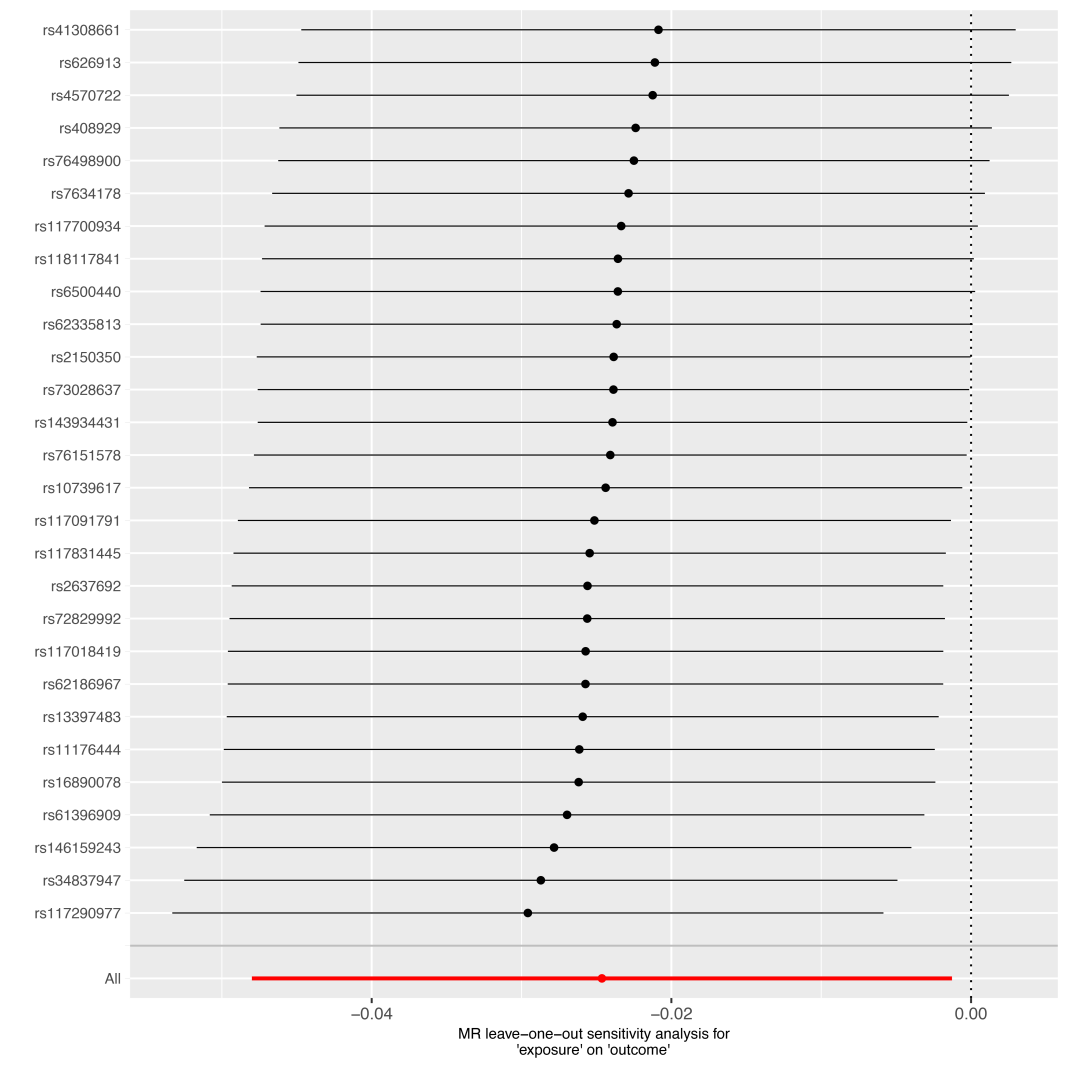


67.GCST90200645


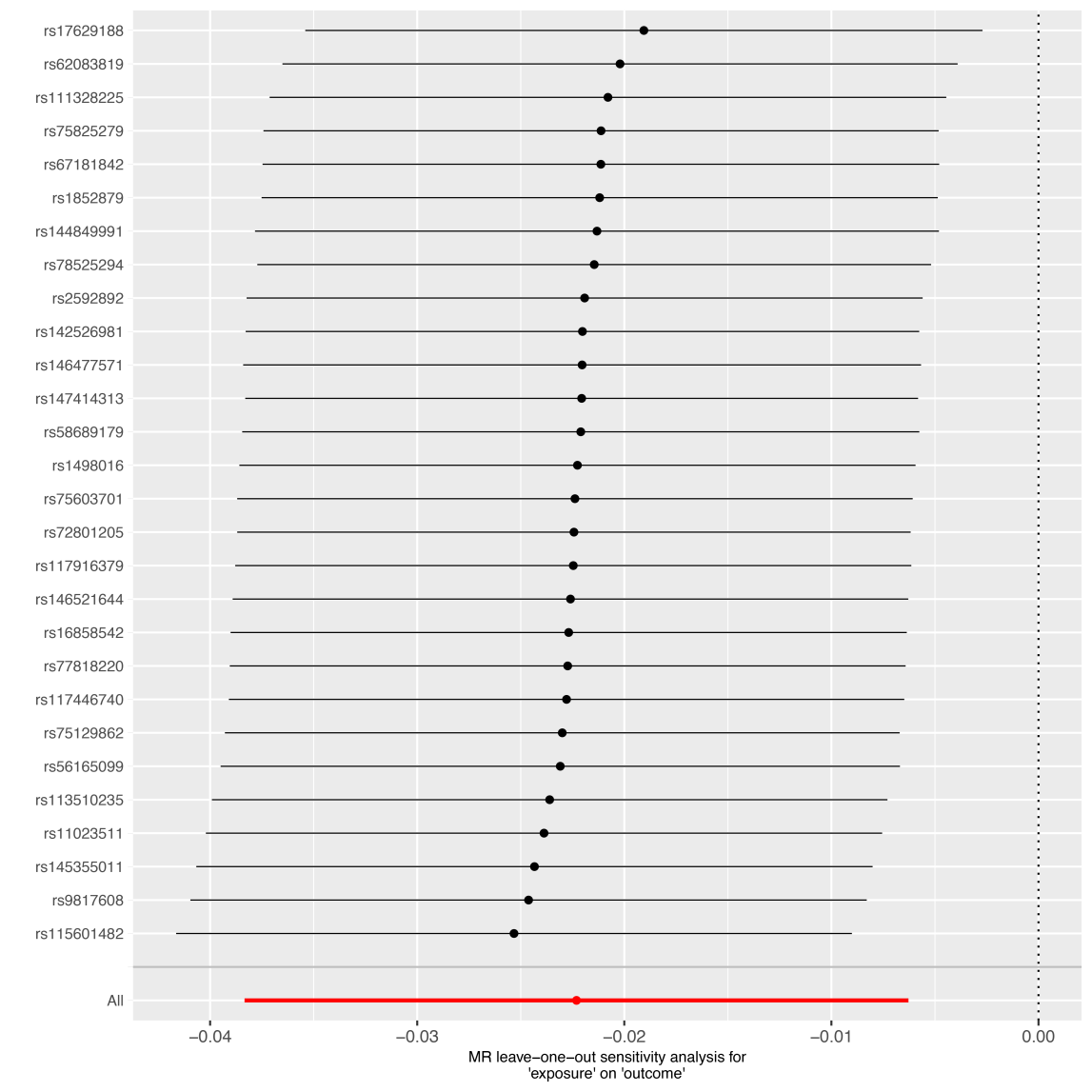


68.GCST90200651


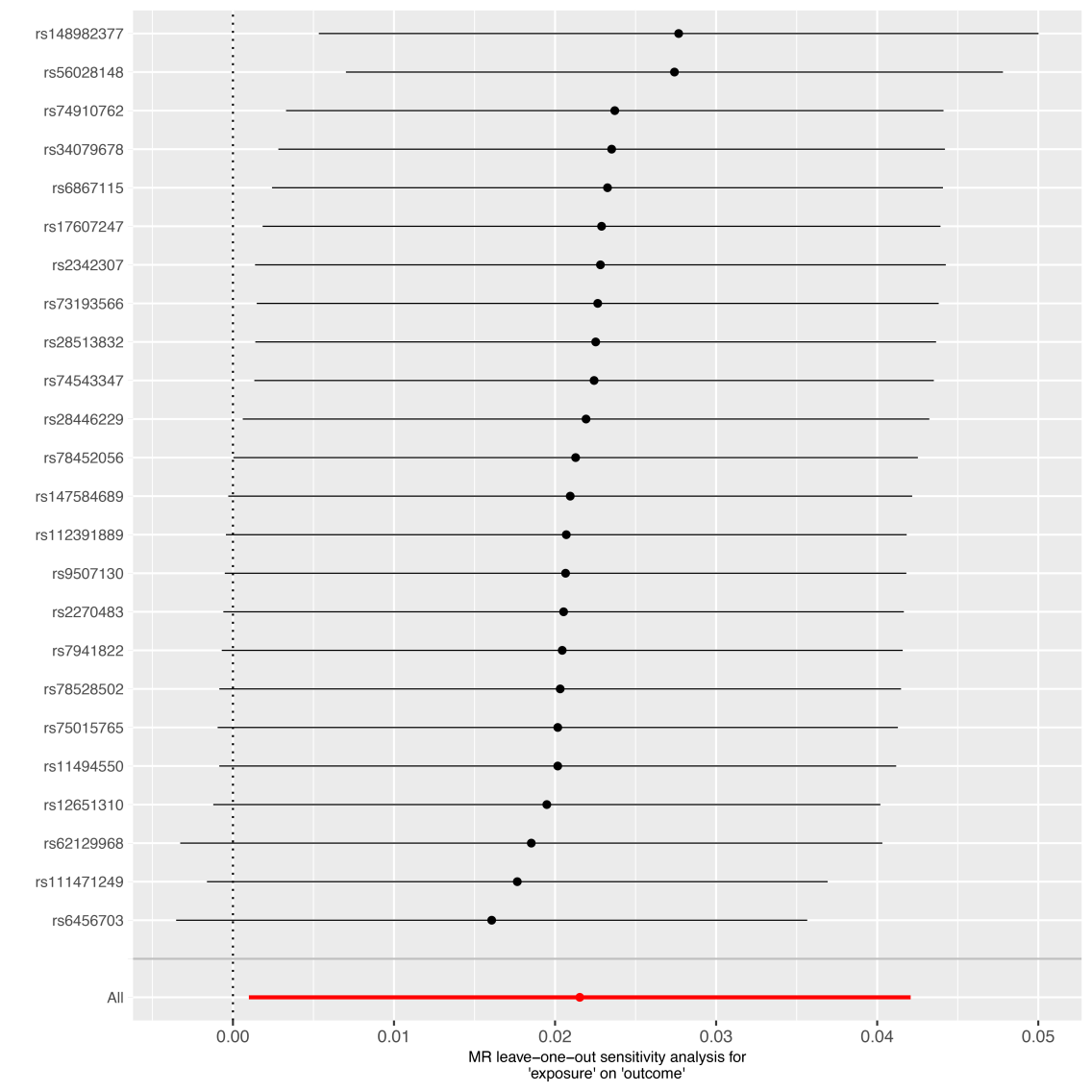


69.GCST90200661


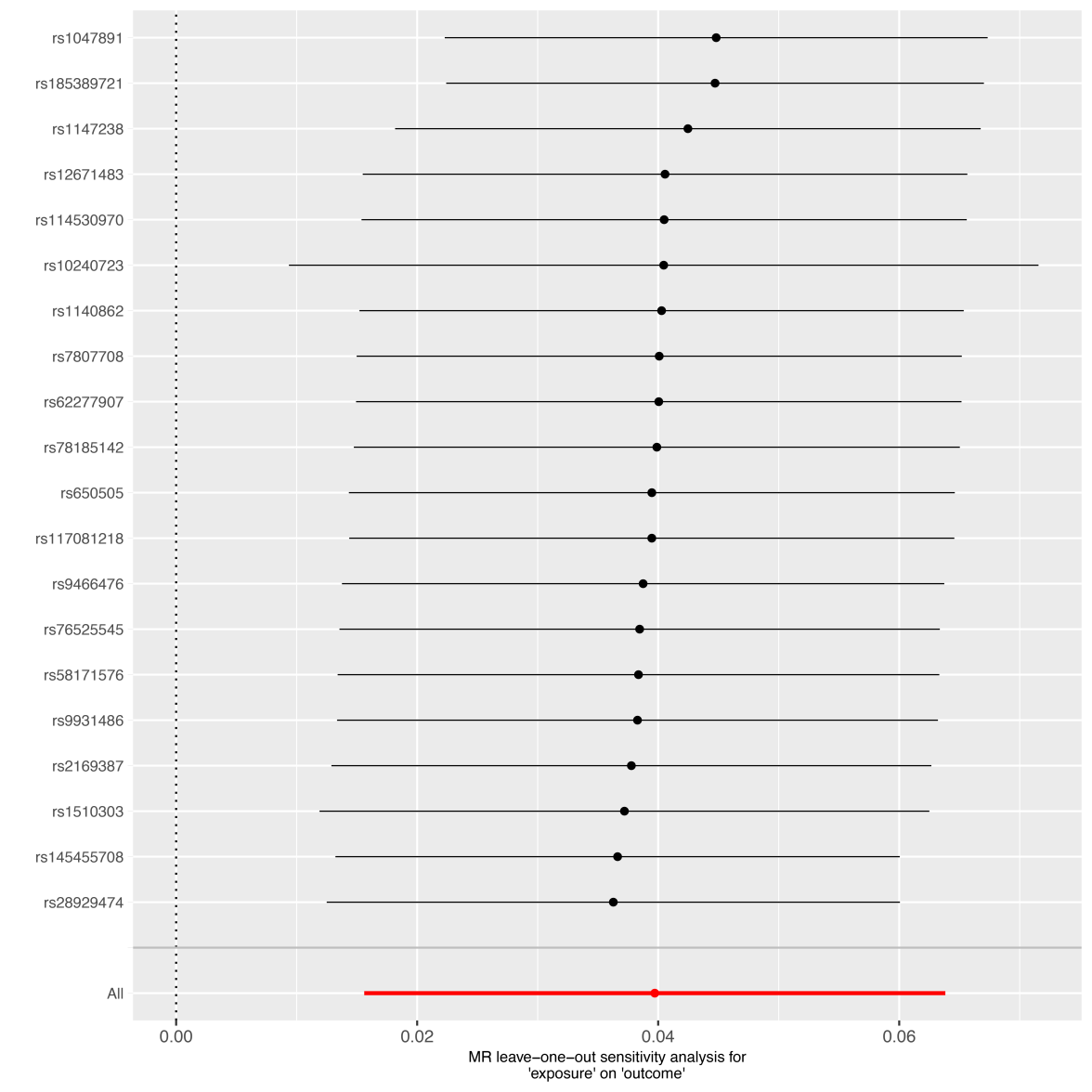


1. GCST90200685


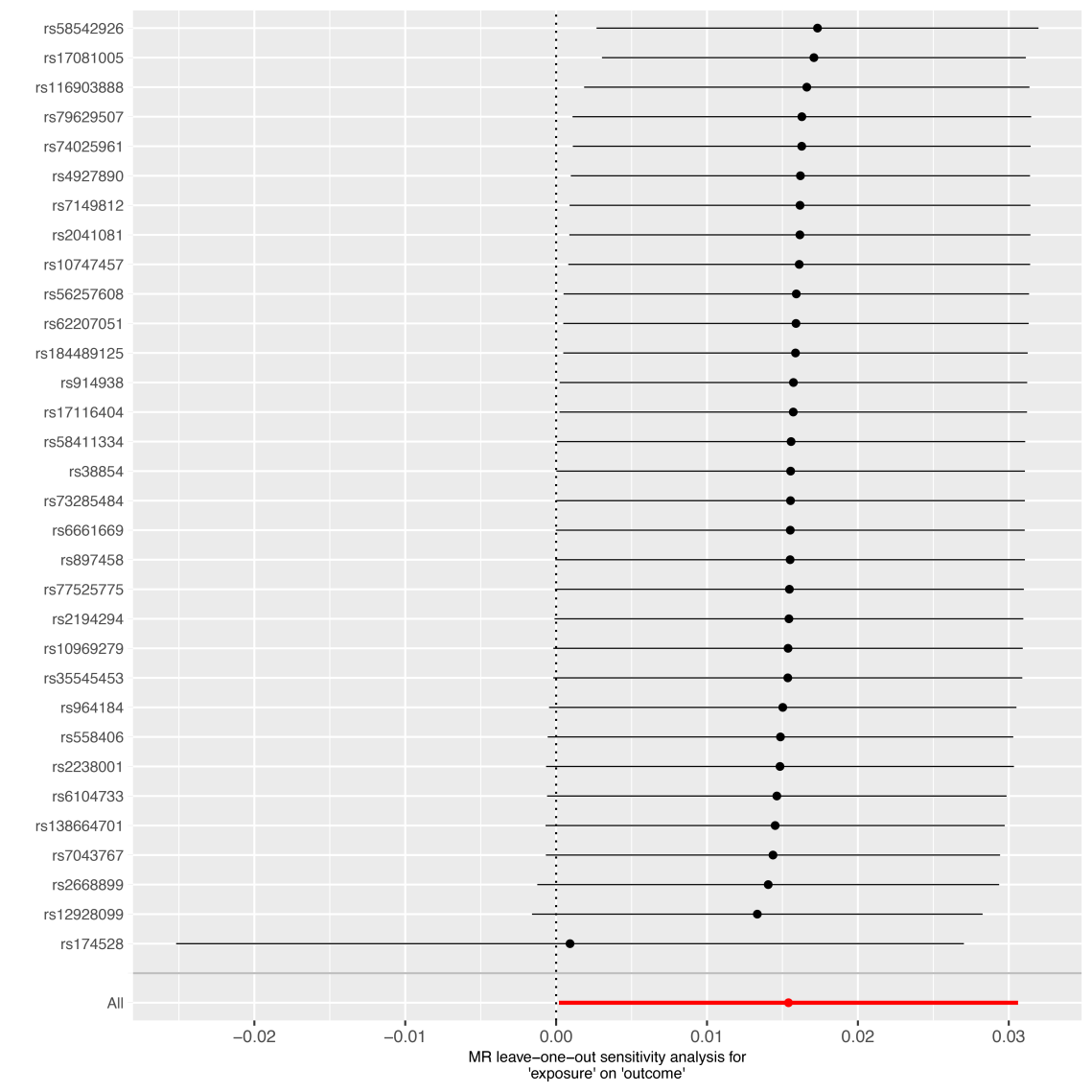


71.GCST90200692


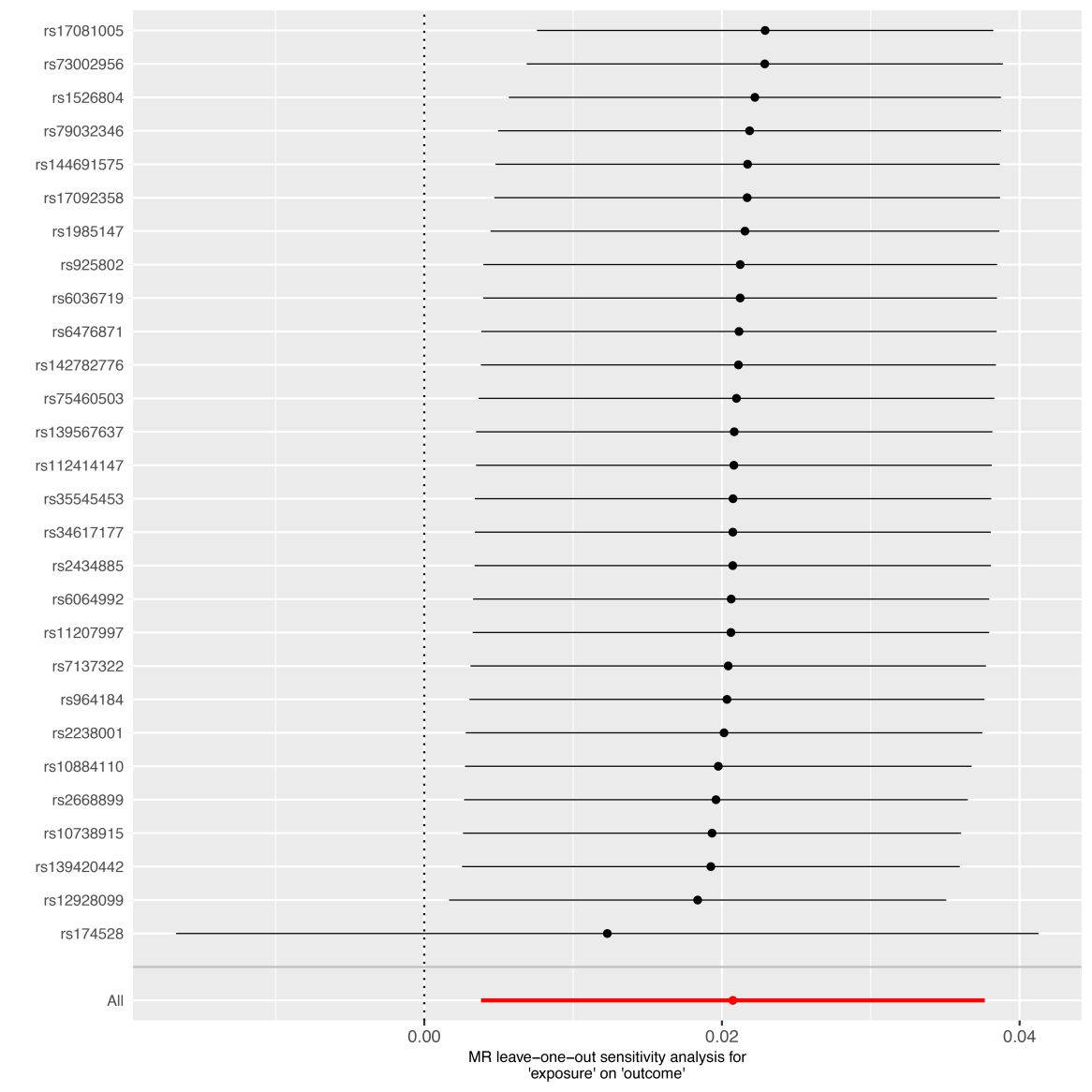


72.GCST90200705


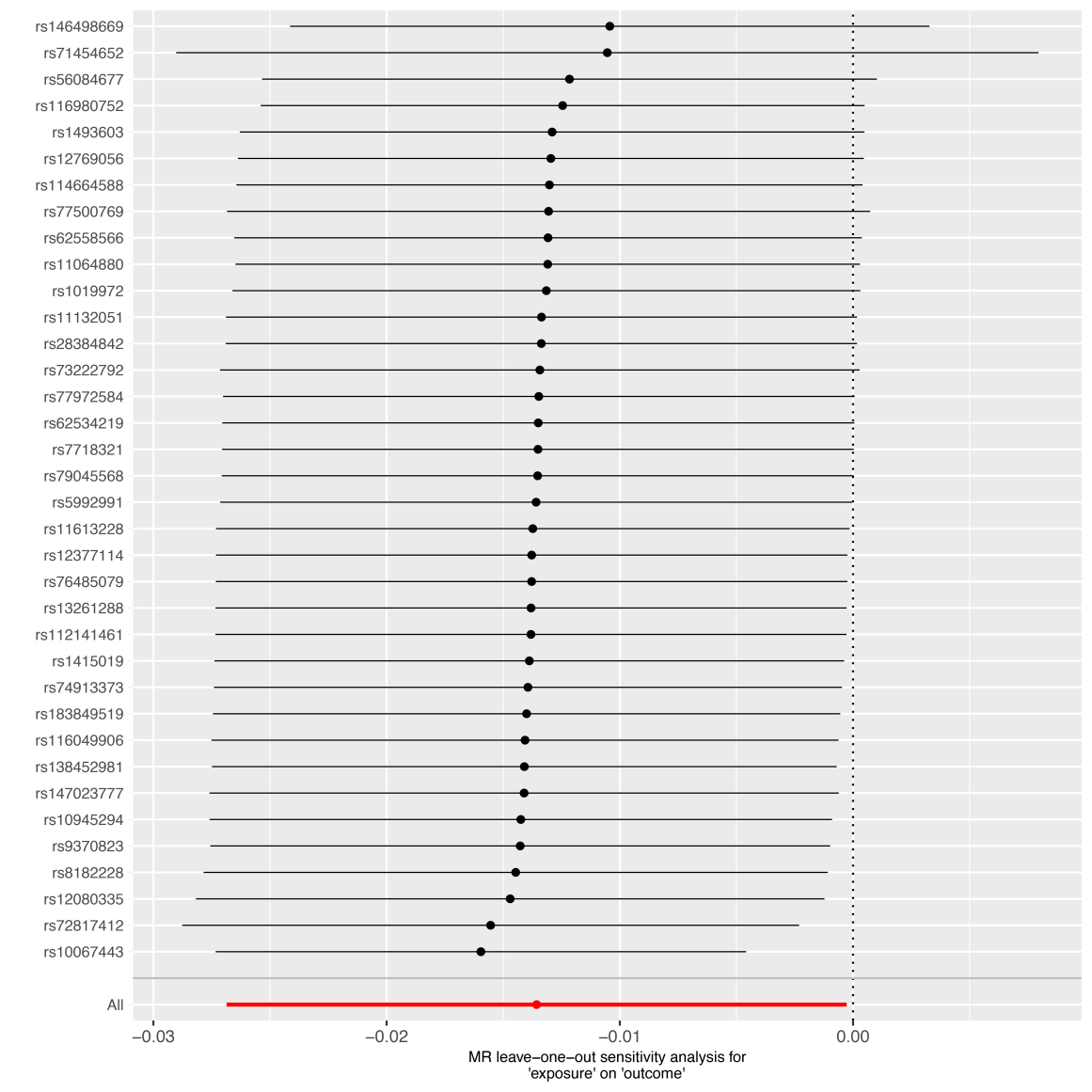


73.GCST90200728


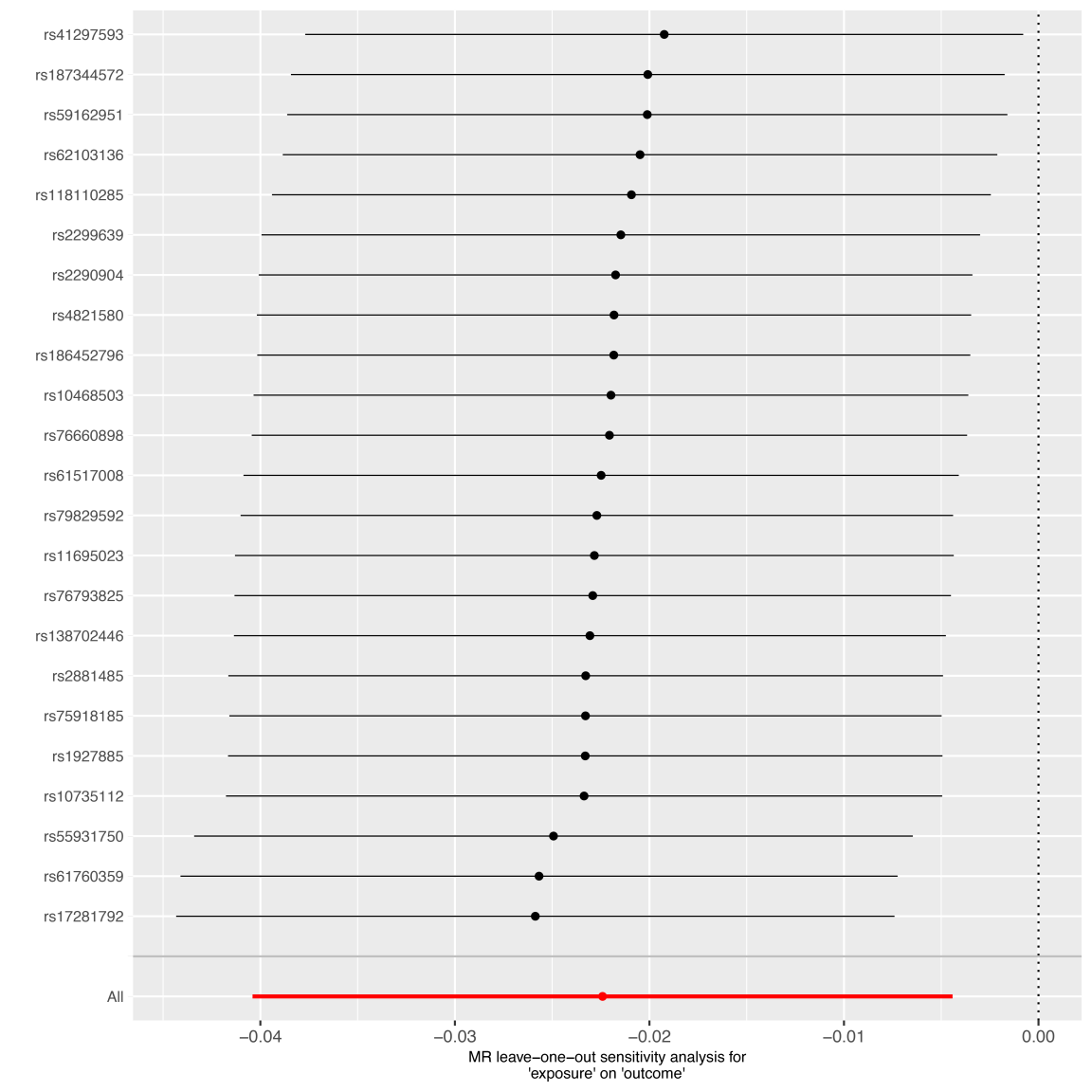


74.GCST90200740


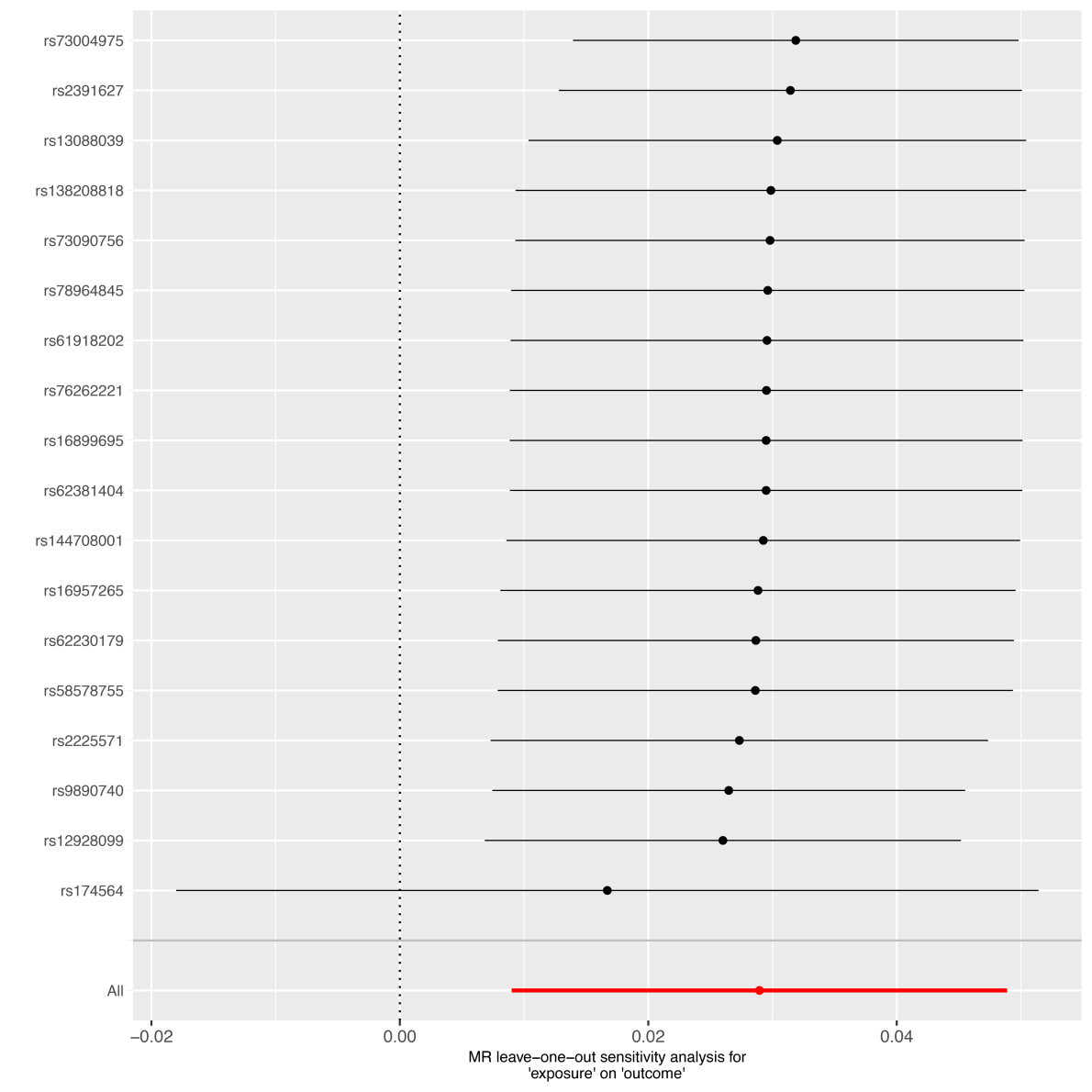


75.GCST90200749


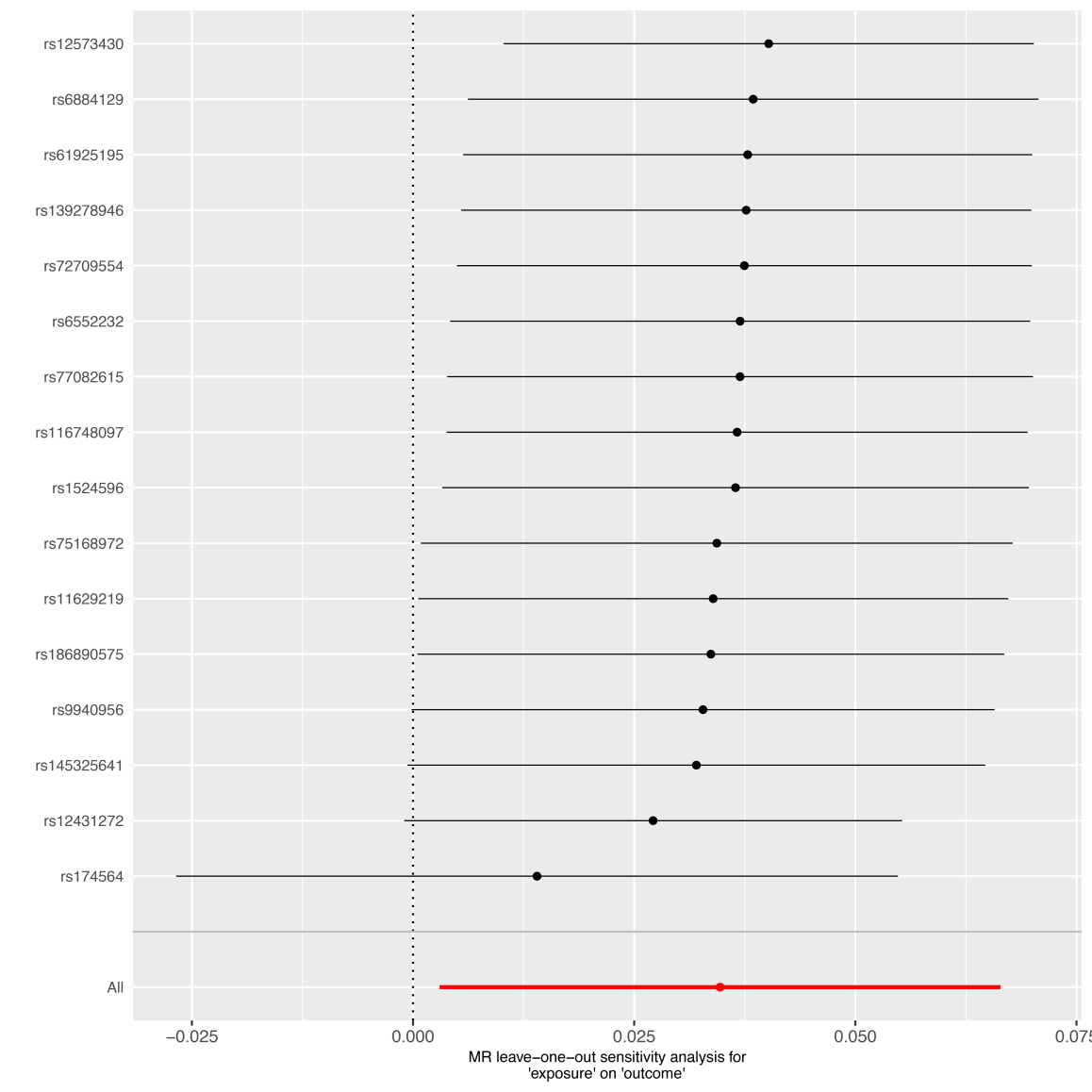


76.GCST90200794


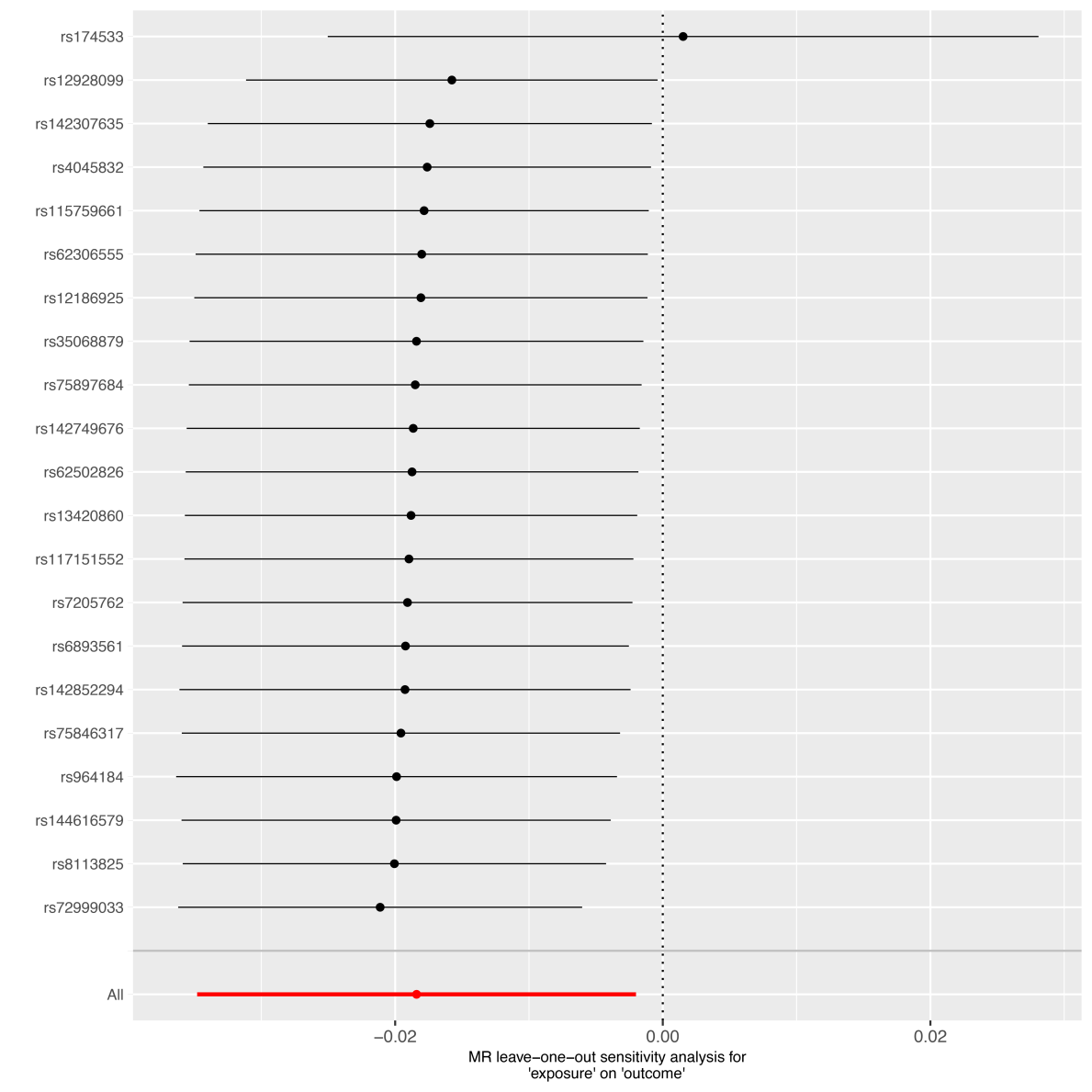


77.GCST90200795


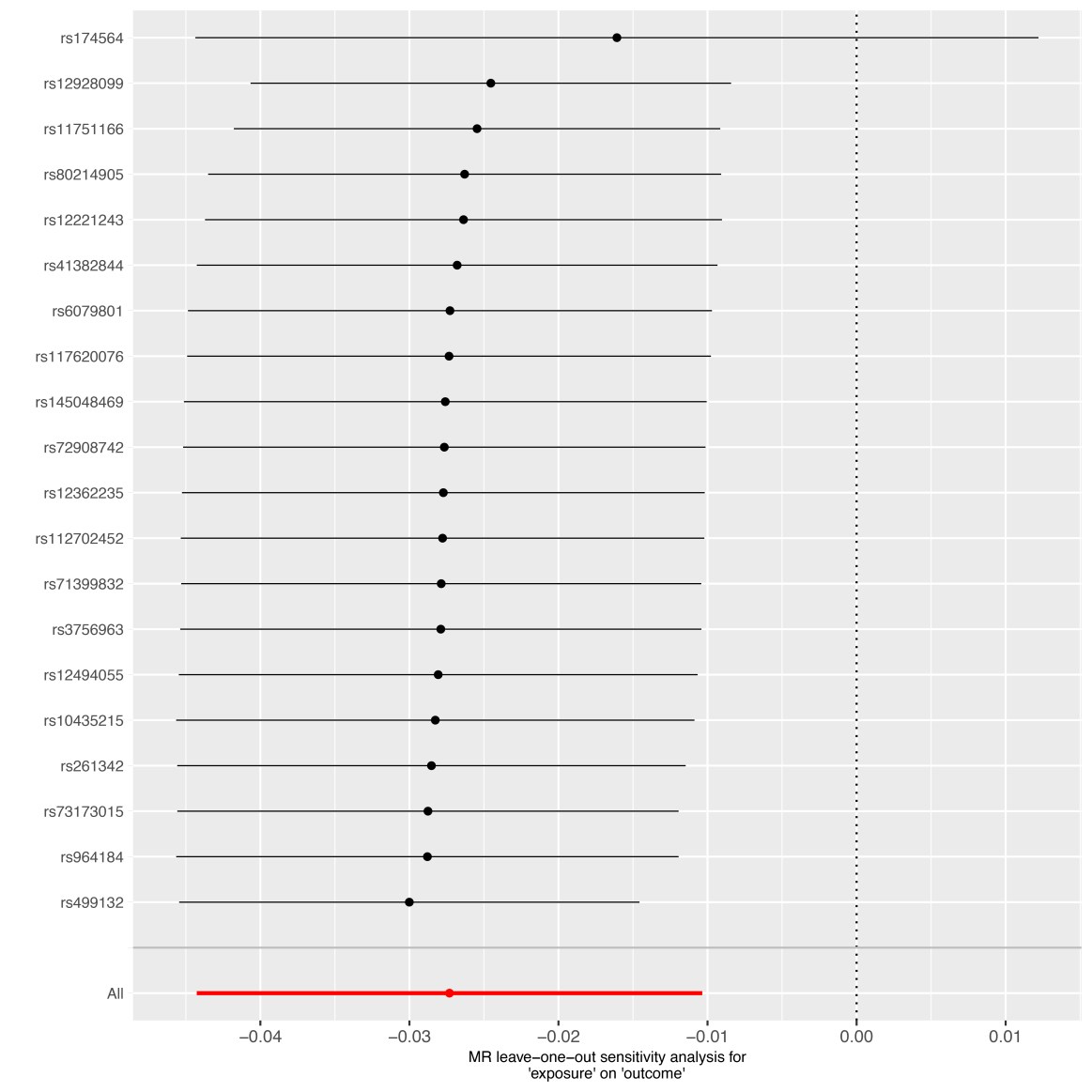


78.GCST90200804


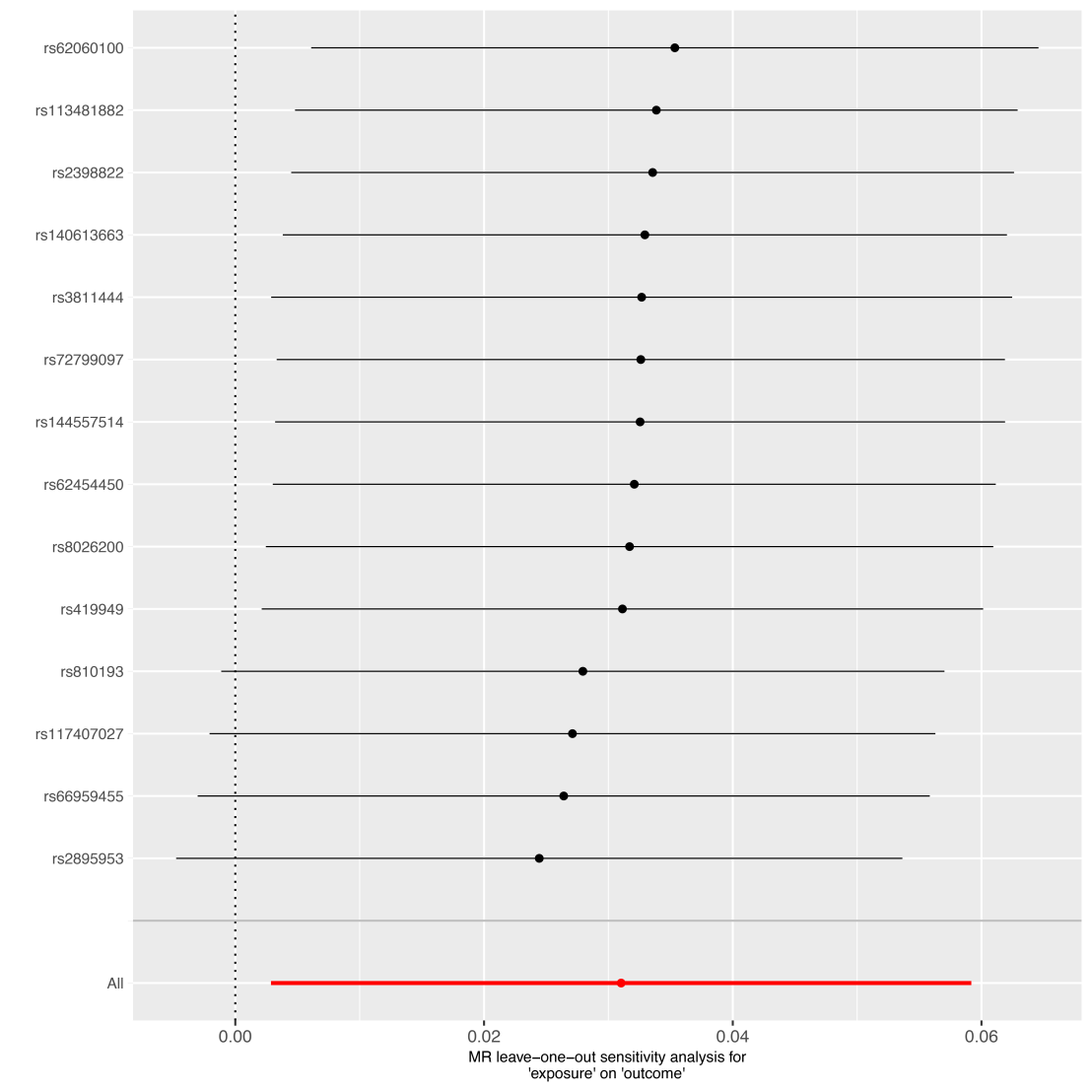


79.GCST90200812


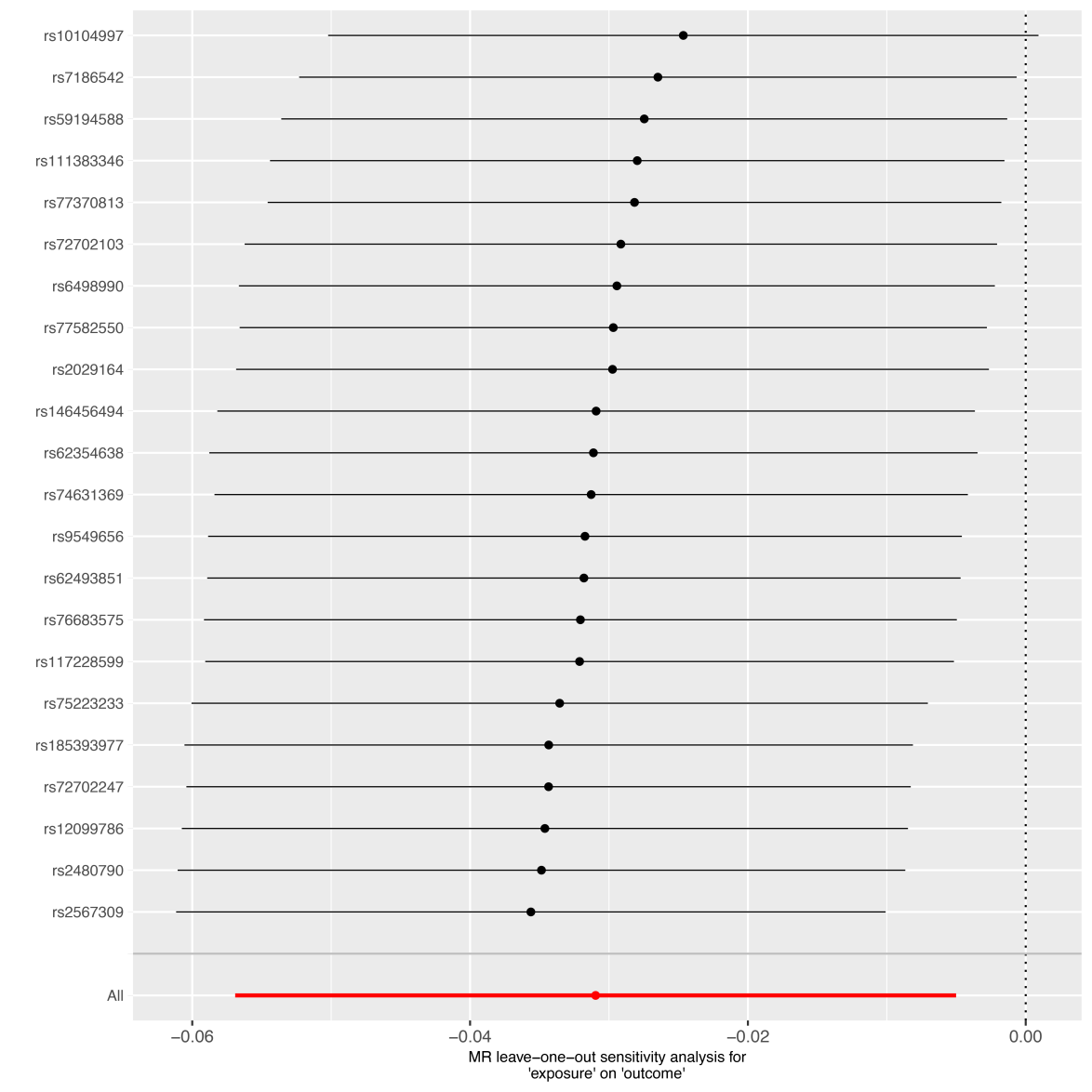


80.GCST90200848


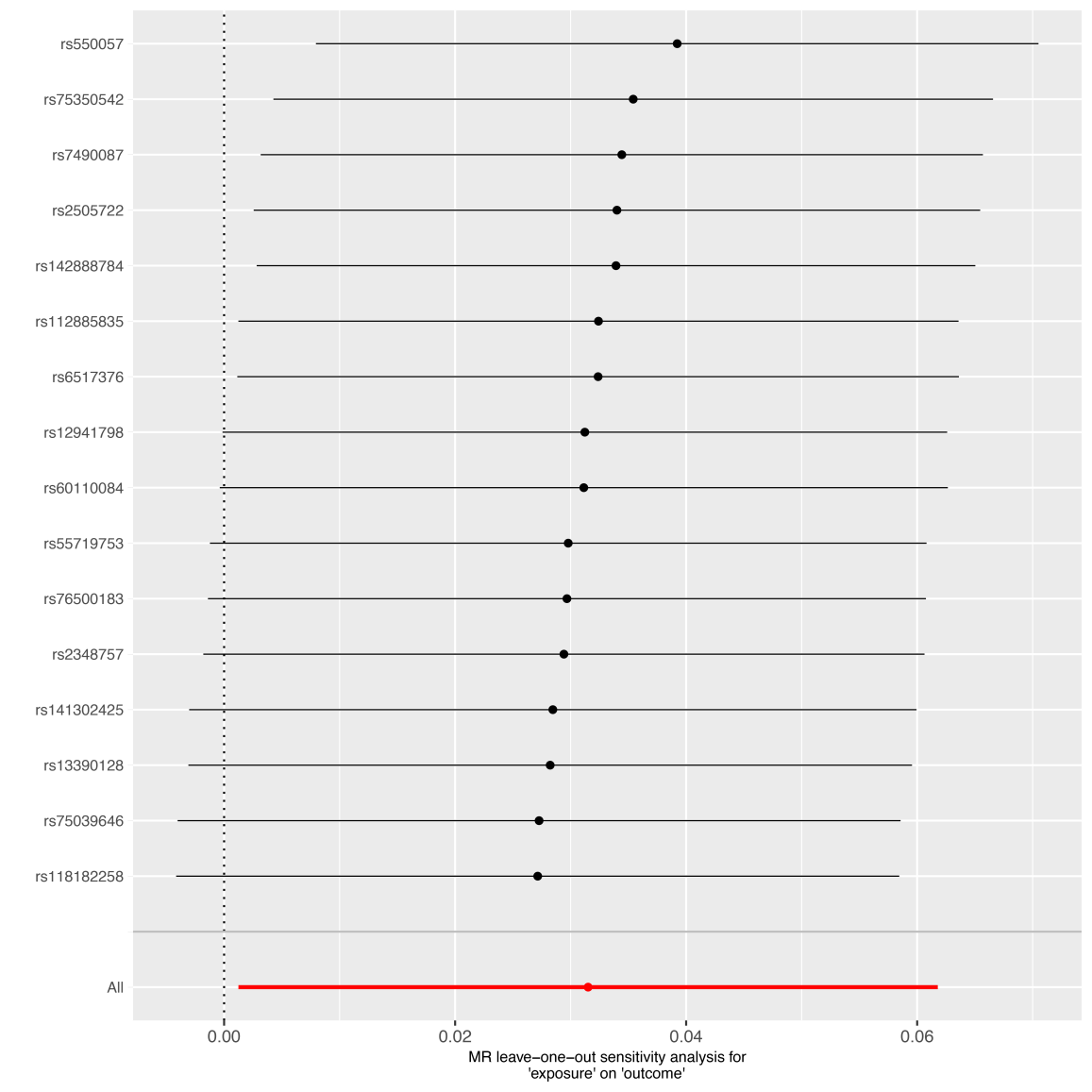


81.GCST90200859


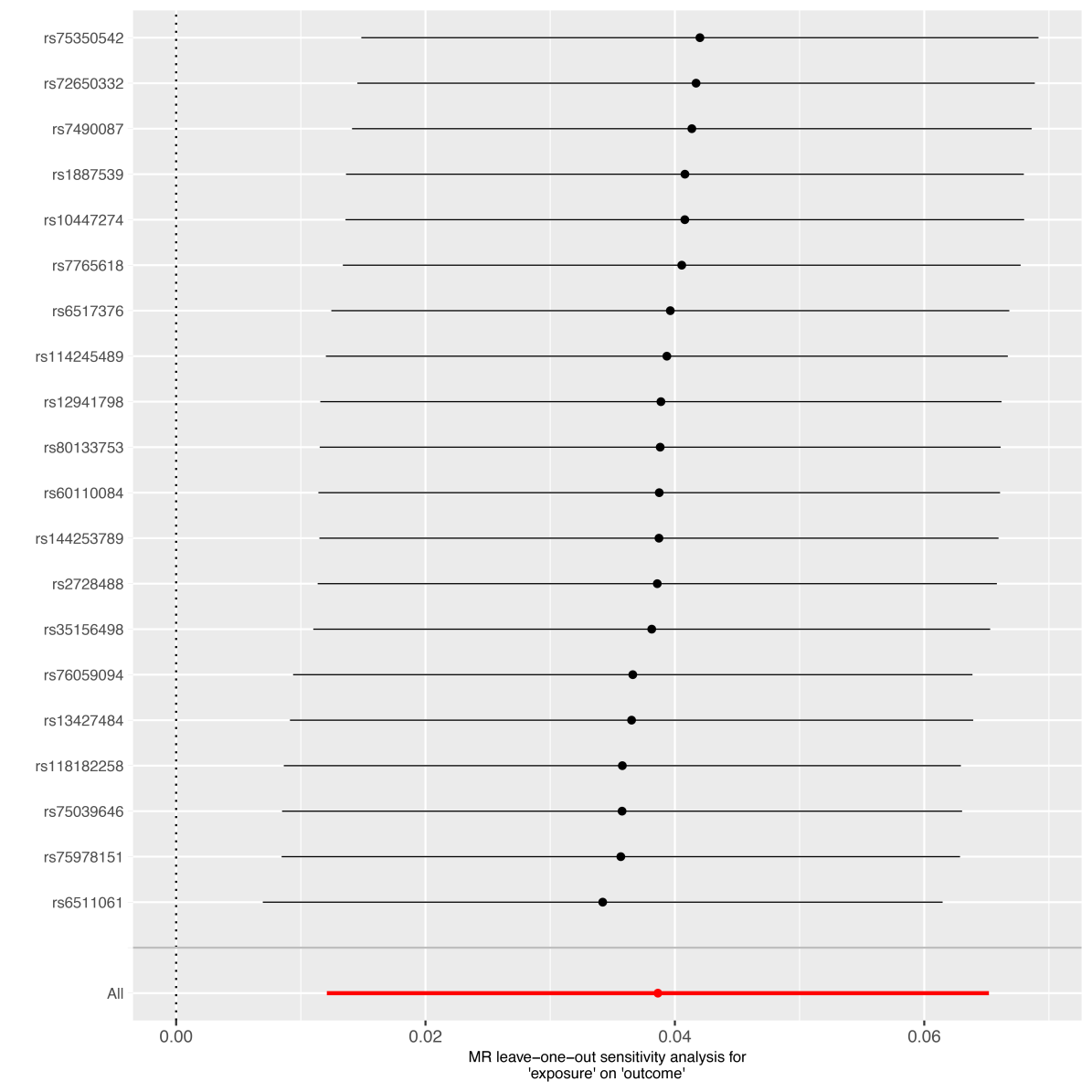


82.GCST90200860


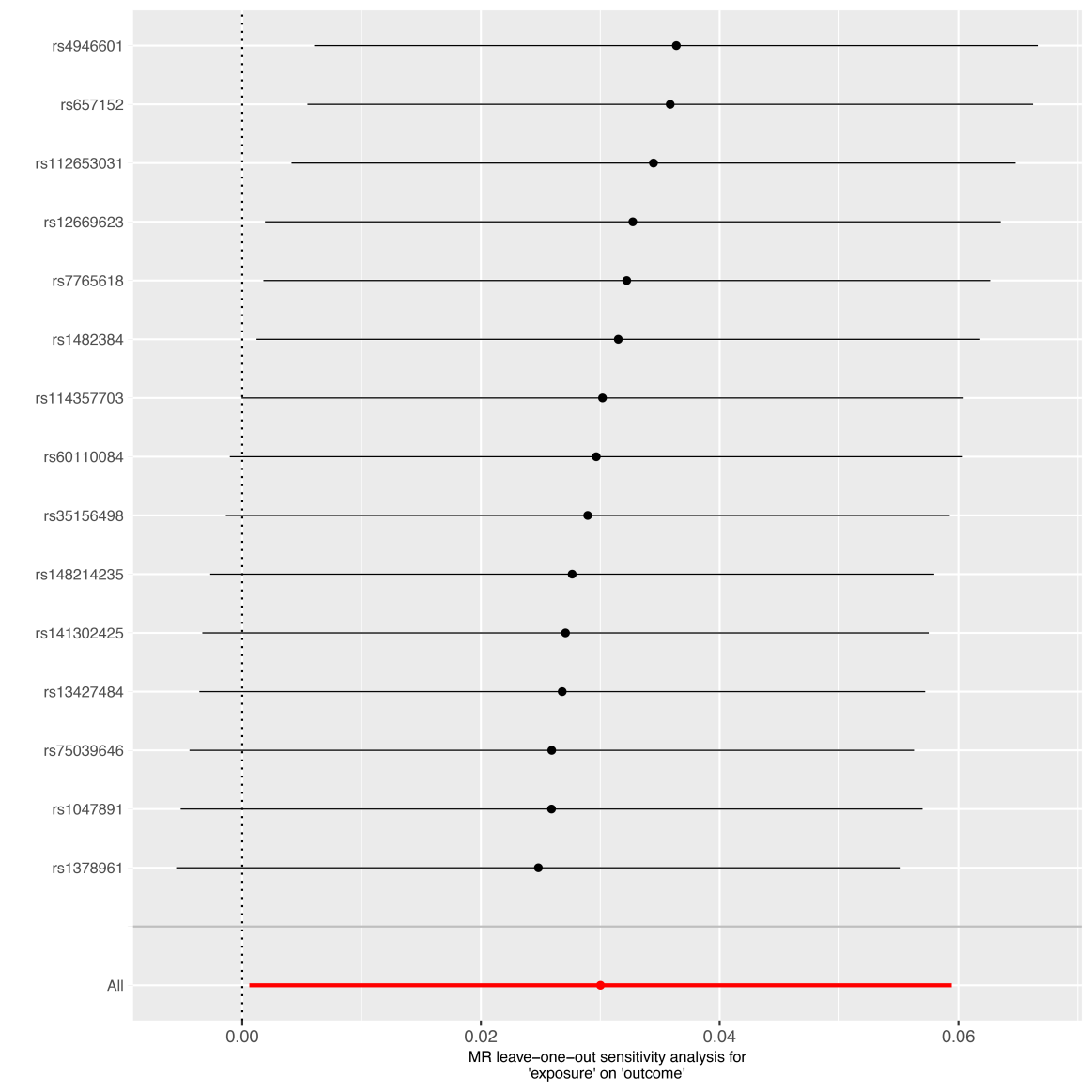


83.GCST90200863


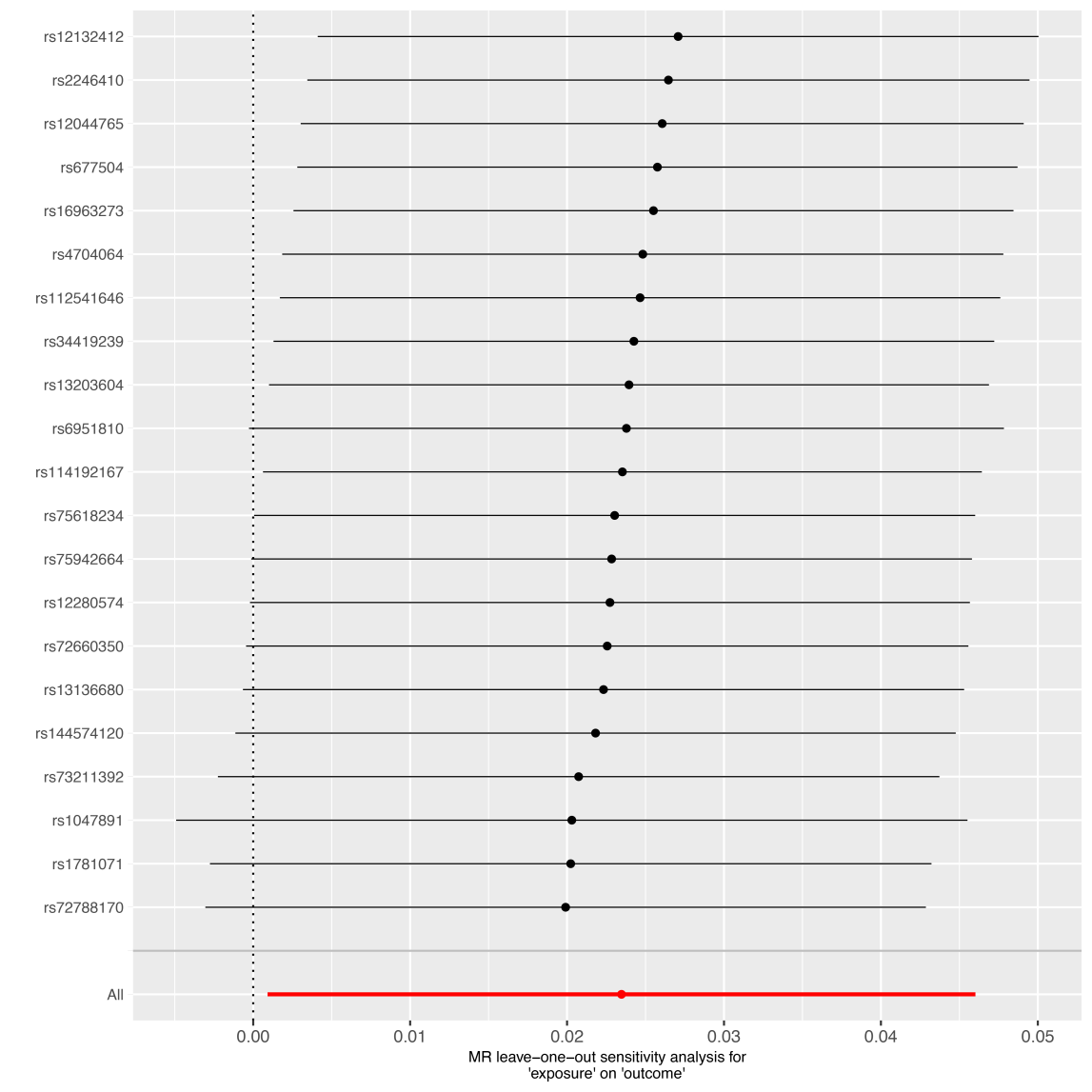


84.GCST90200864


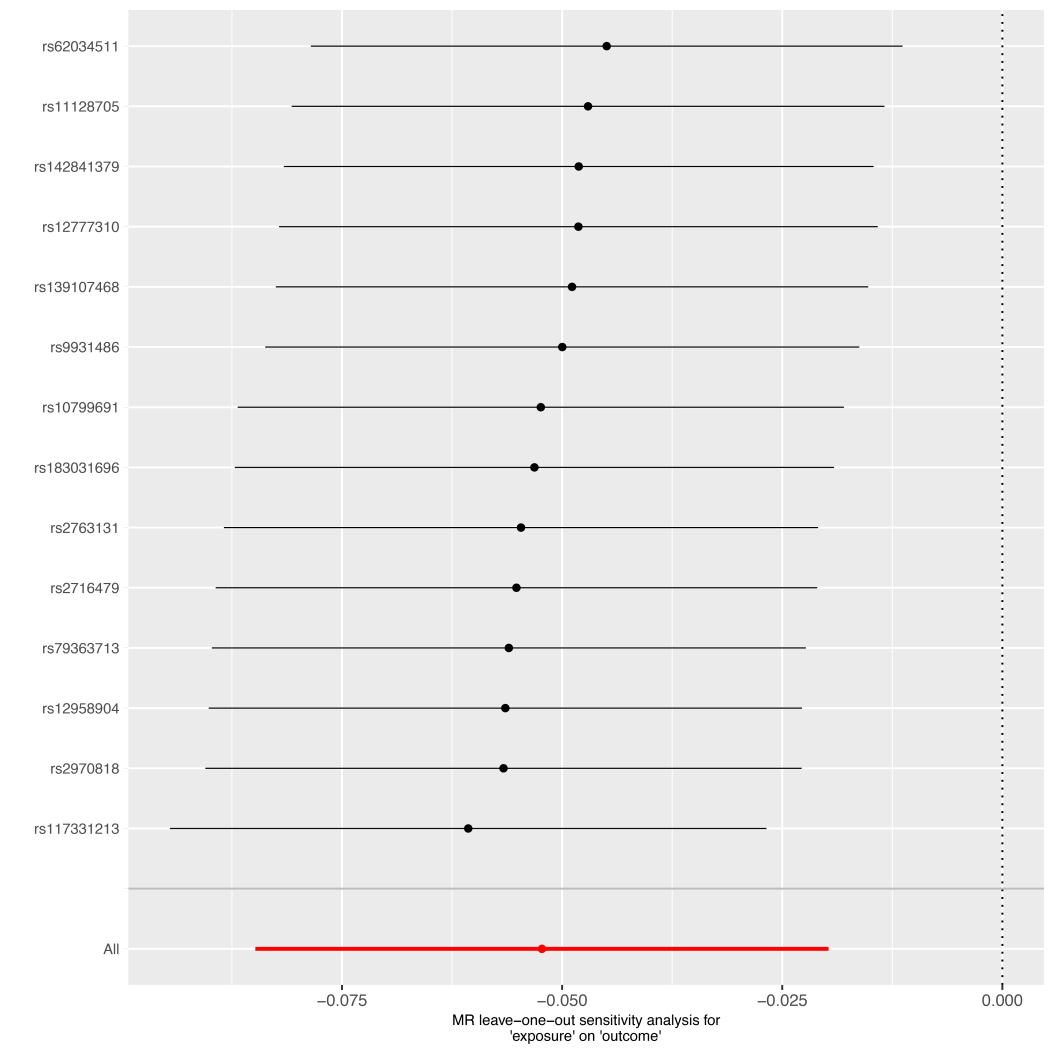


85.GCST90200867


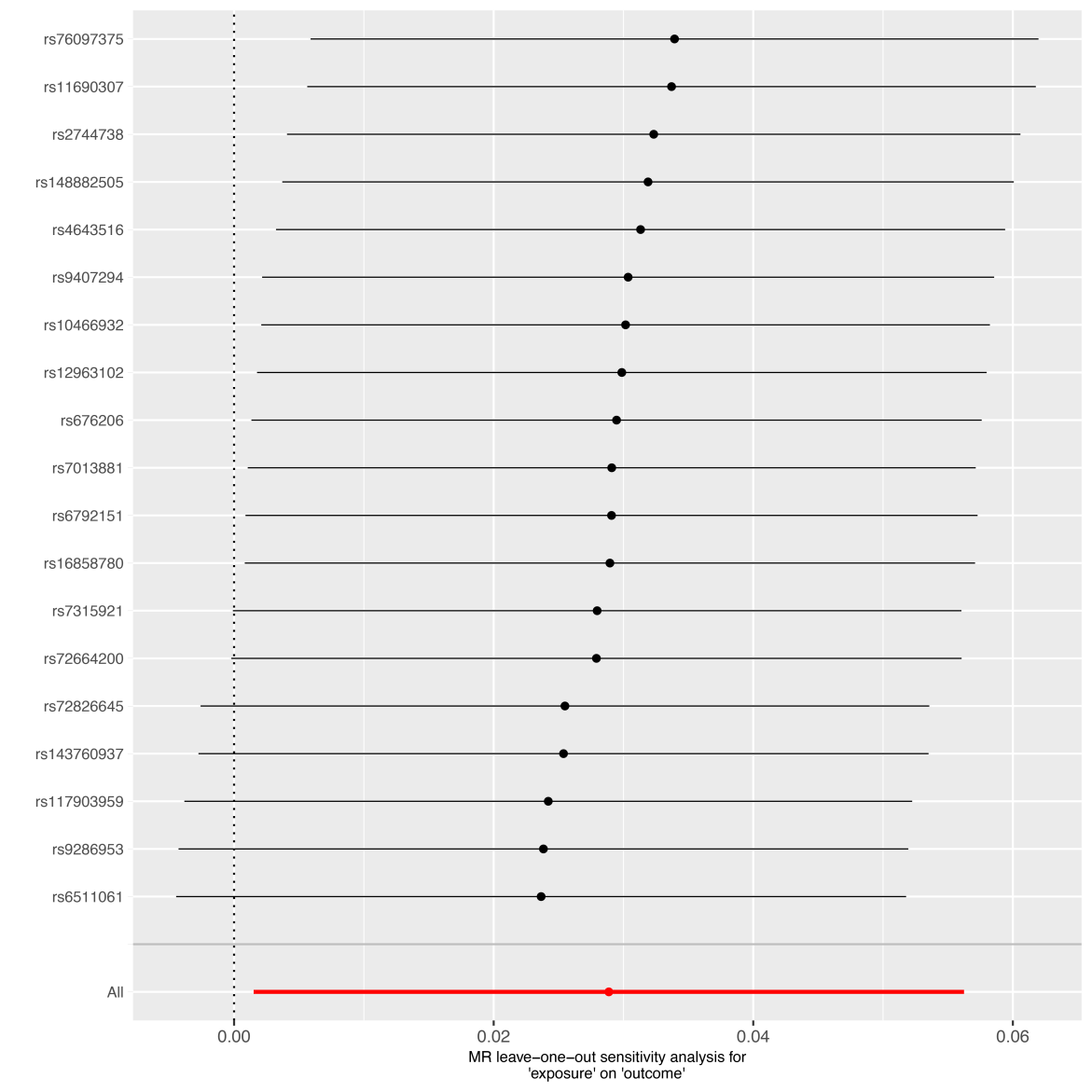


86.GCST90200885


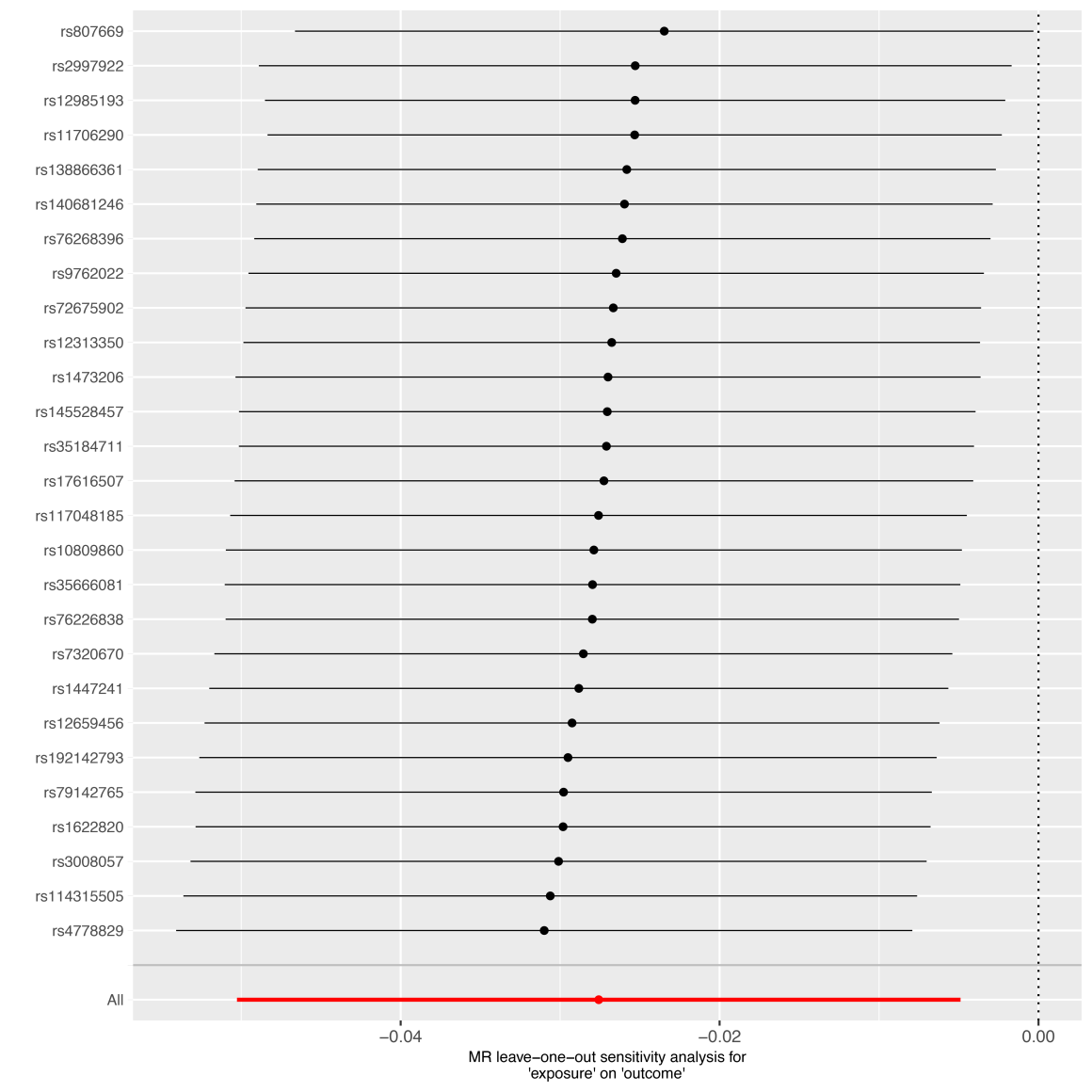


87.GCST90200890


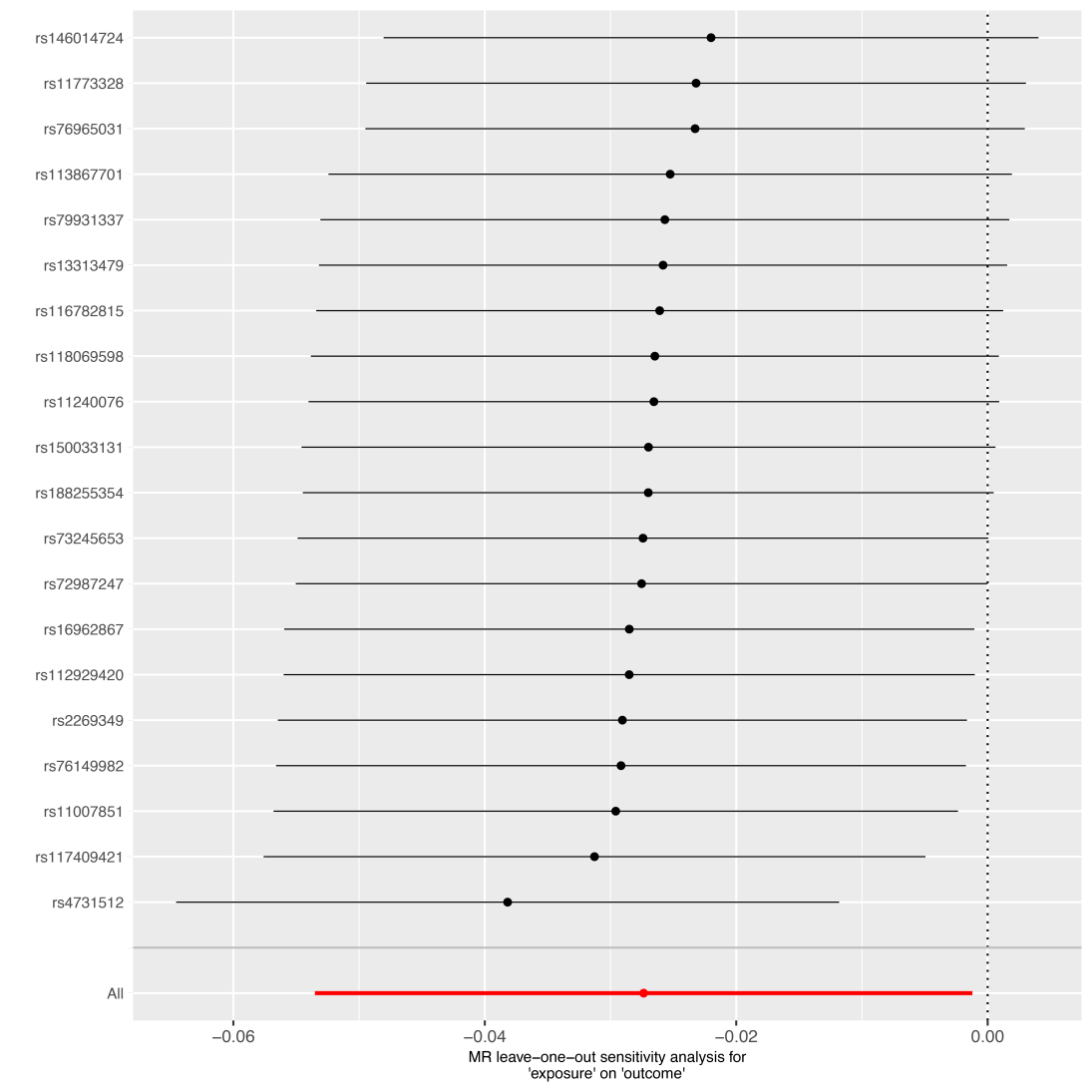


88.GCST90200913


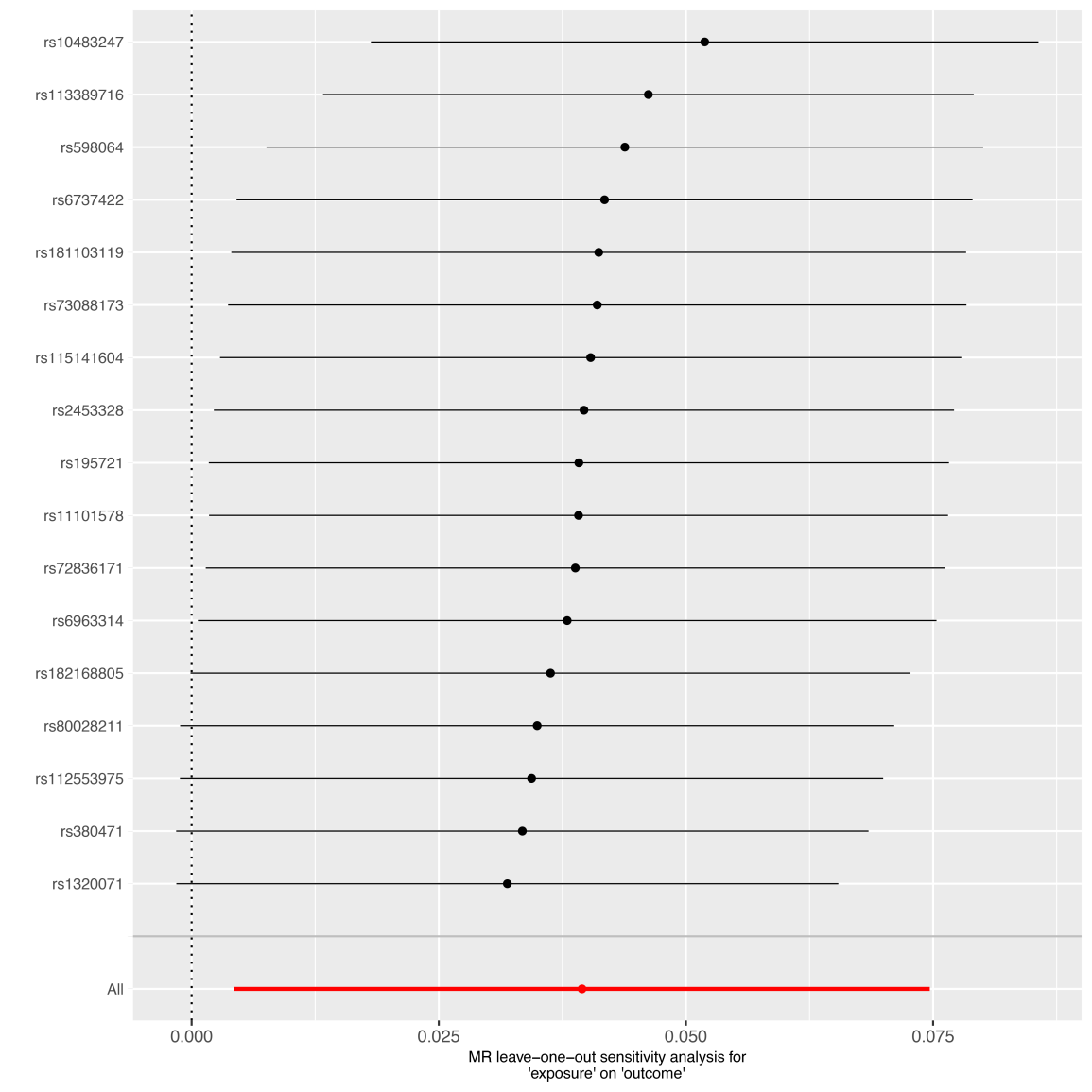


89.GCST90200933


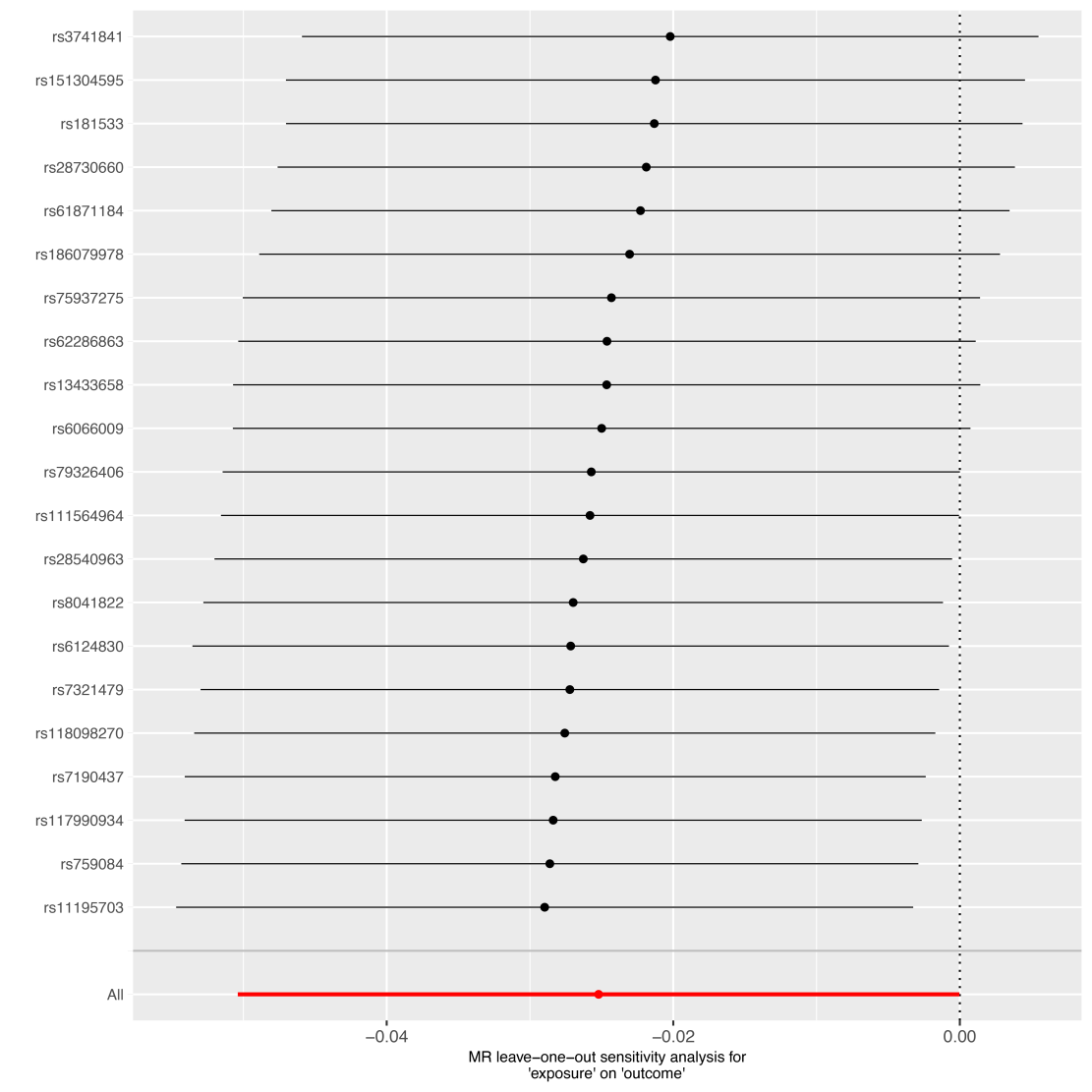


90.GCST90200935


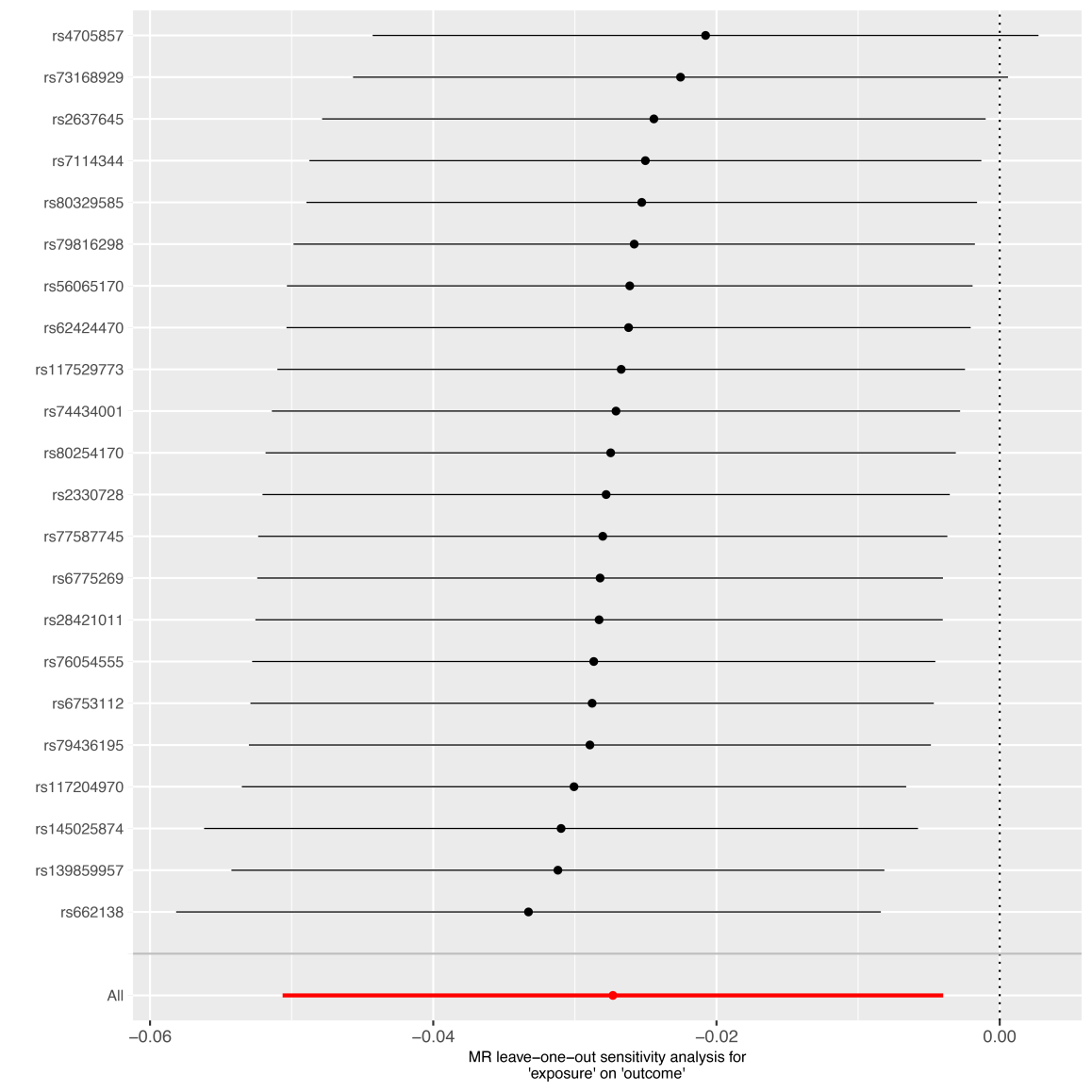


91.GCST90200968


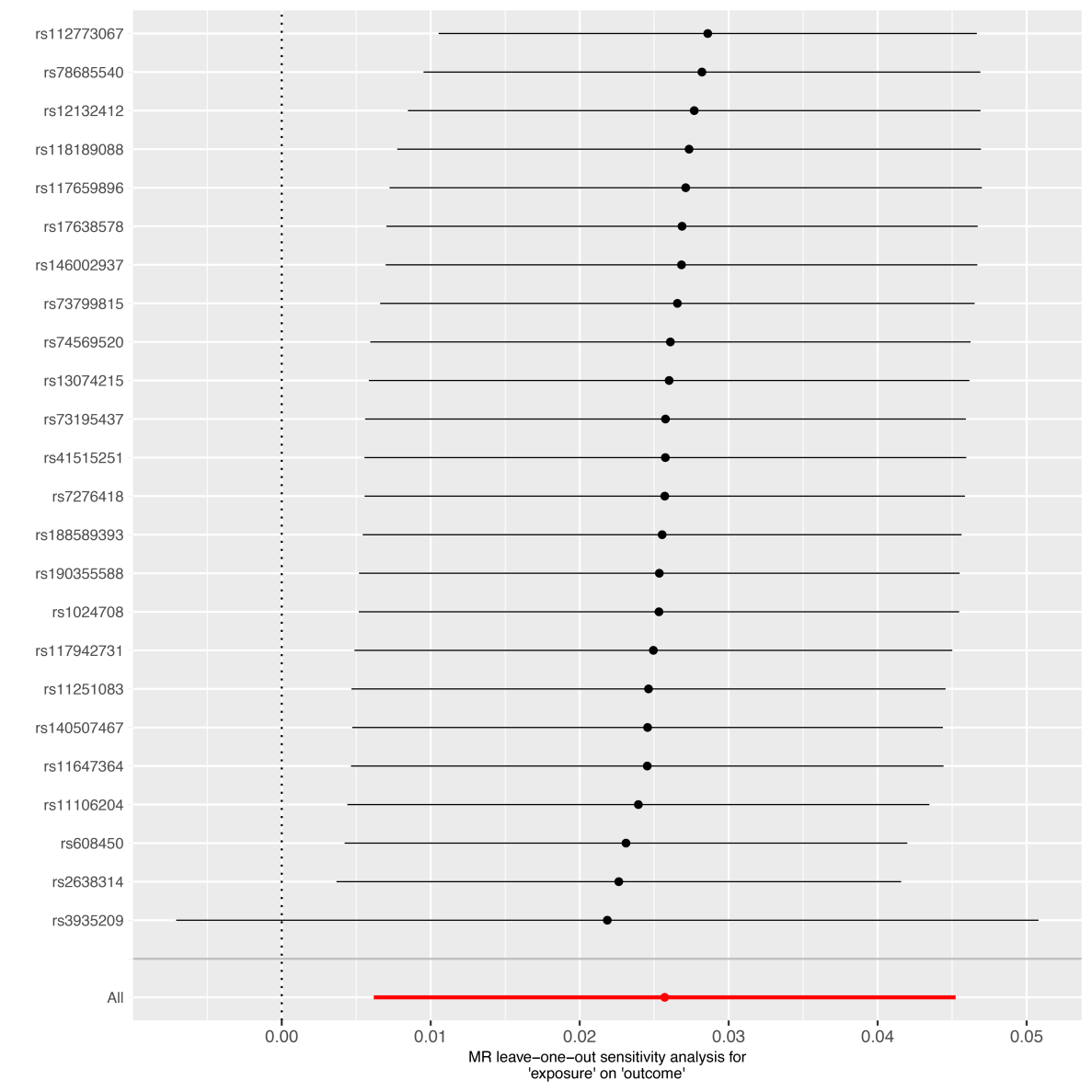


92.GCST90200979


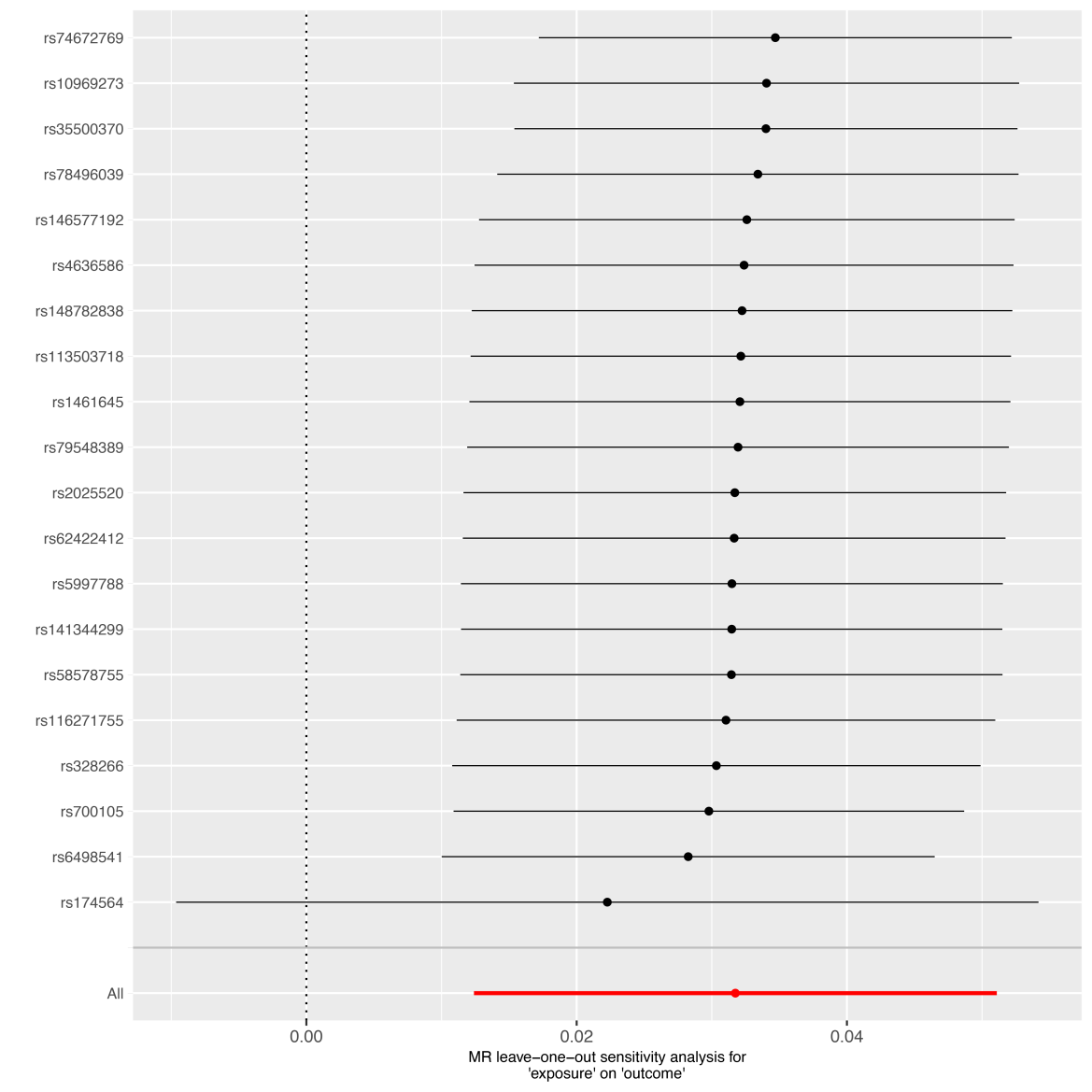


93.GCST90200990


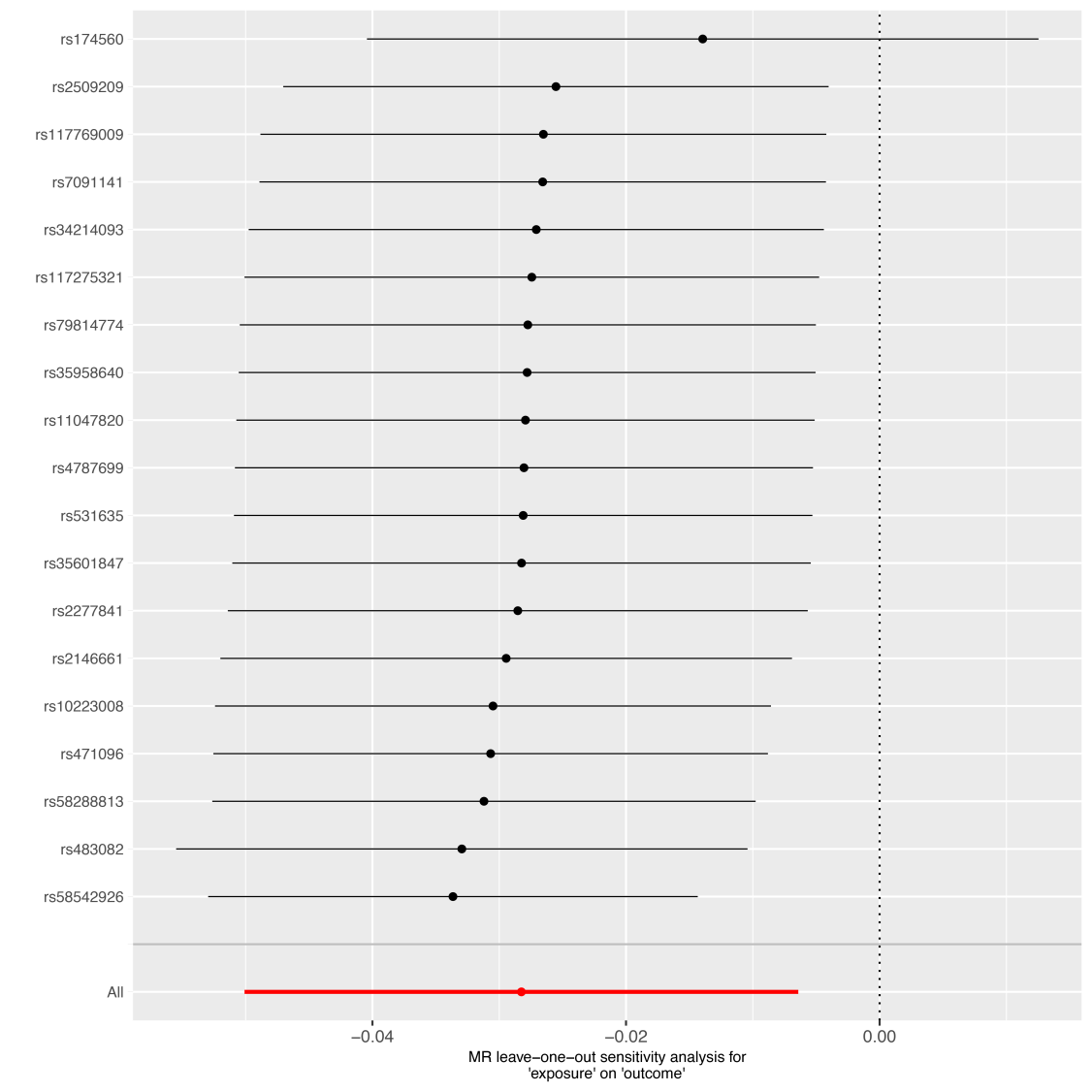


94.GCST90201007


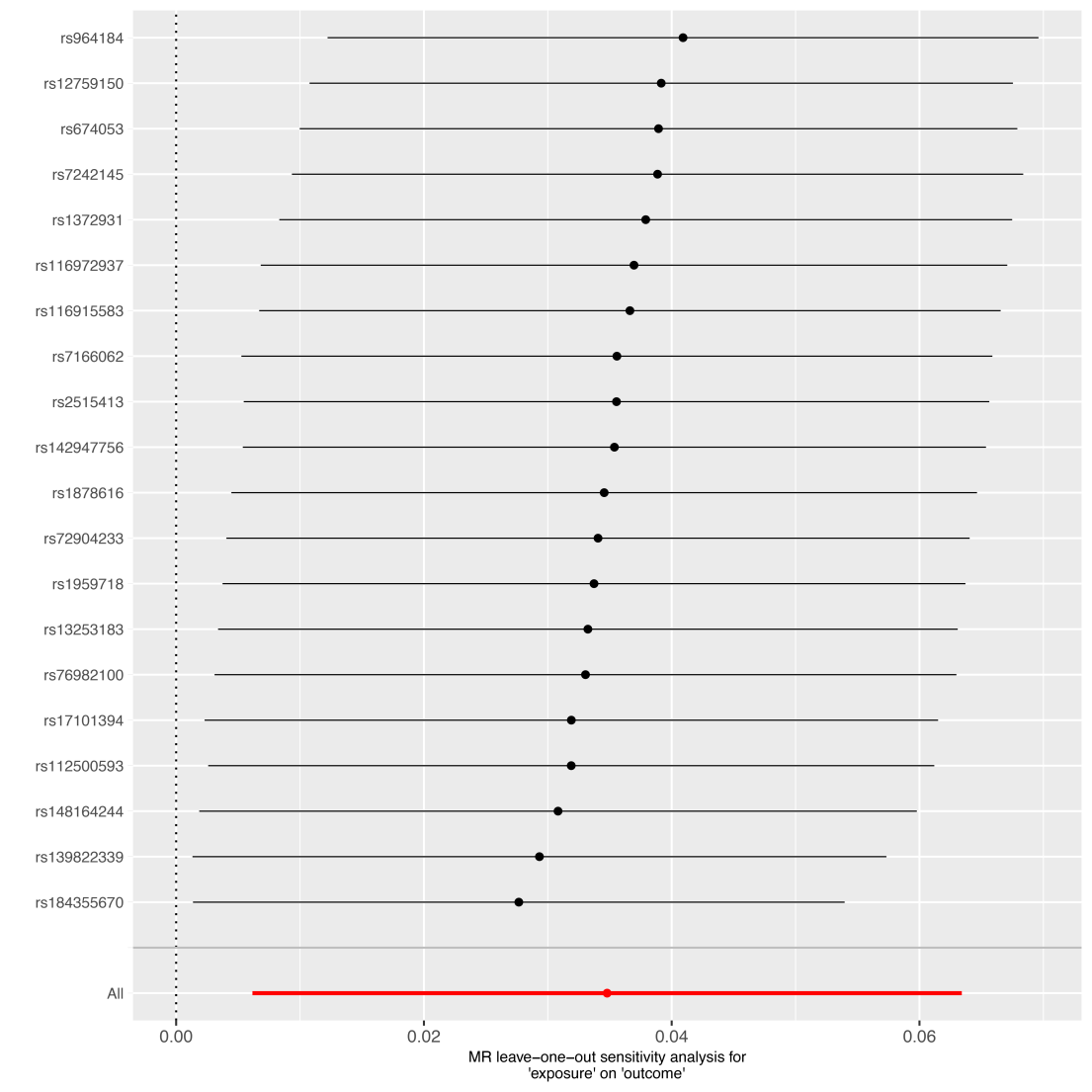


95.GCST90201009


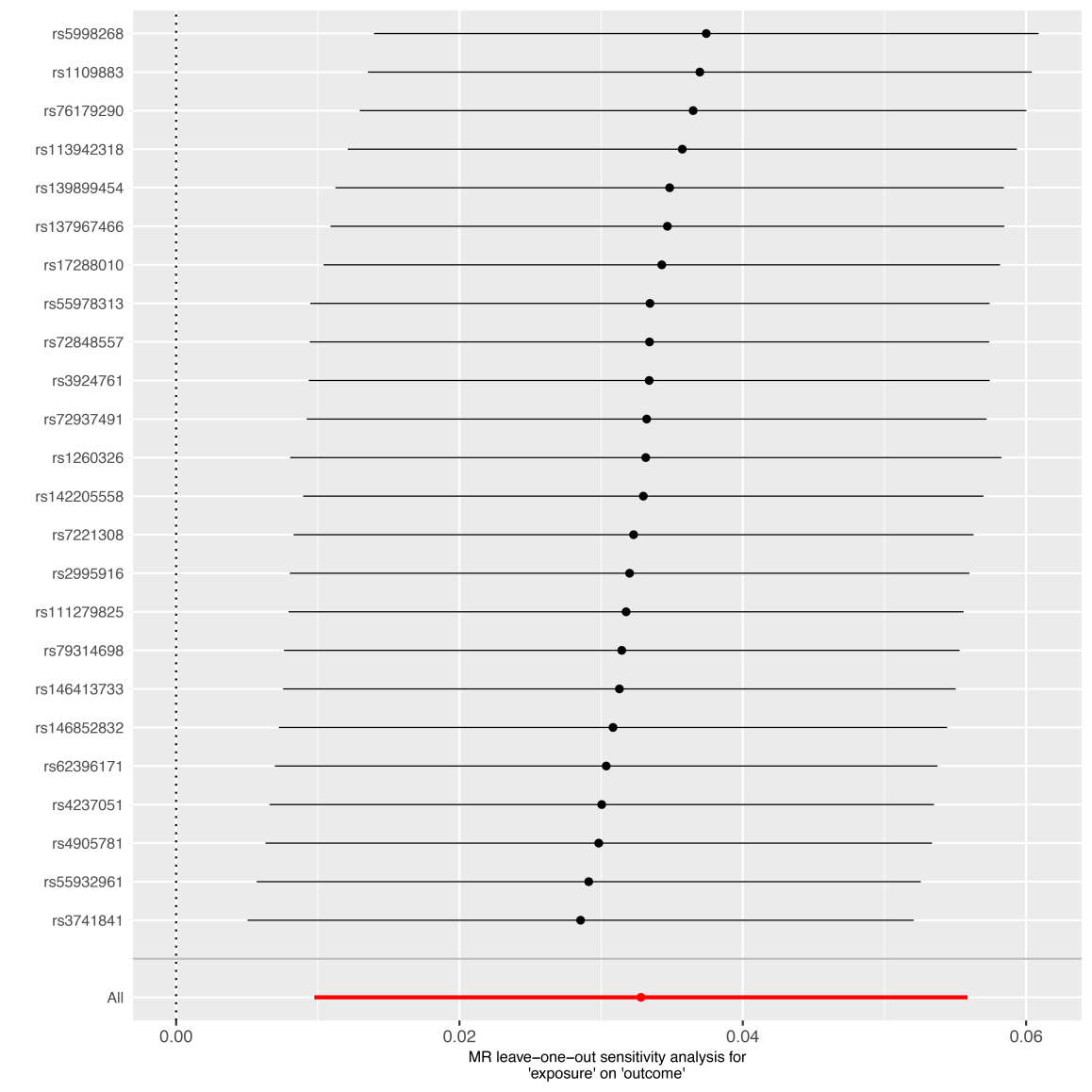


96.GCST90201013


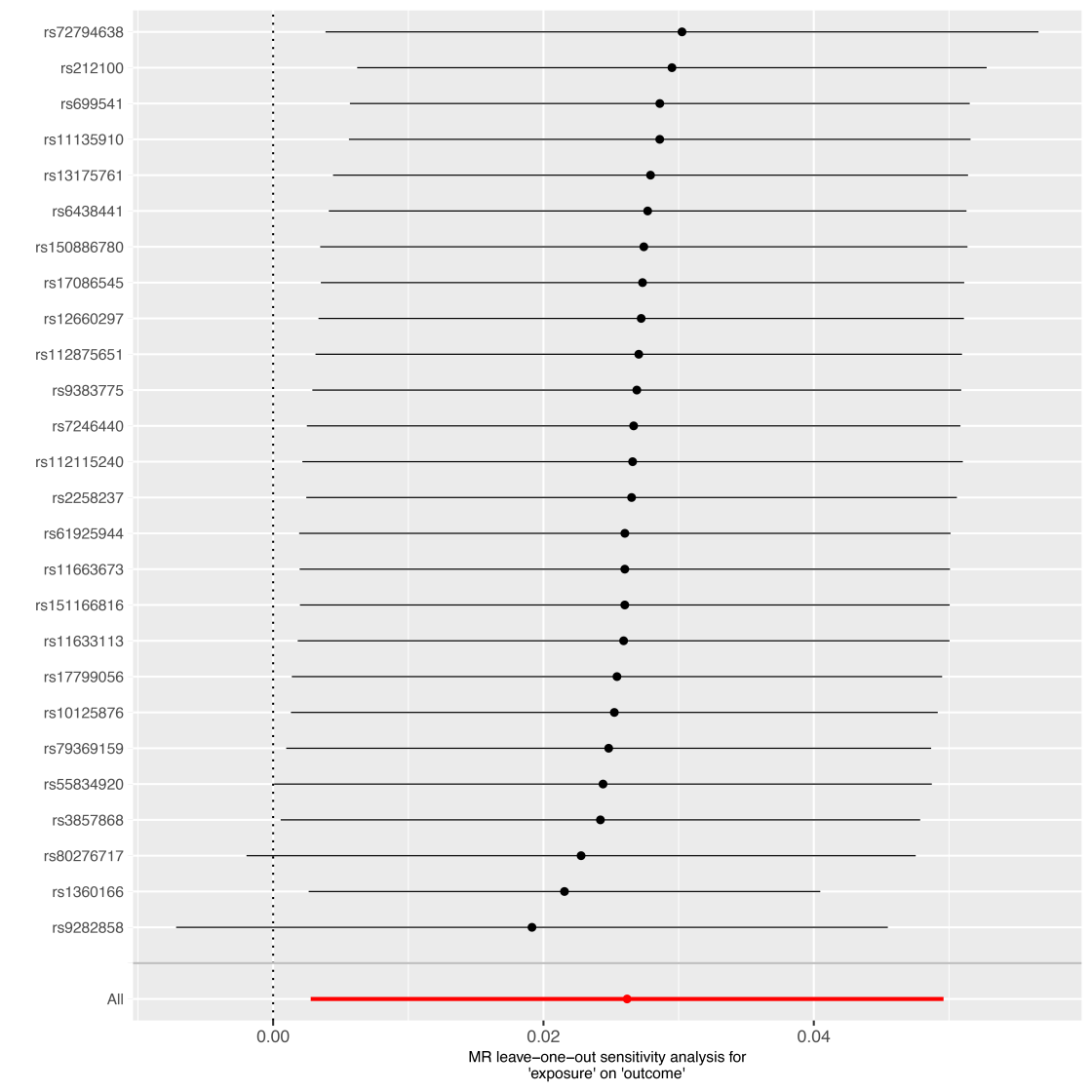


4.Supplementary Table 4: 50 significant SNPS were extracted as instrumental variables (IVs) for Major depressive Disorder (MDD)

|  | chr.exposure | pos.exposure | other_allele.exposure | effect_allele.exposure | beta.exposure | se.exposure | pval.exposure | eaf.exposure | SNP | exposure | mr_keep.exposure | pval_origin.exposure | id.exposure | samplesize.exposure | R2 | F |
| --- | --- | --- | --- | --- | --- | --- | --- | --- | --- | --- | --- | --- | --- | --- | --- | --- |
| 1 | 1 | 18122009 | T | C | -0.0264 | 0.0046 | 9.69E-09 | 0.326 | rs4141983 | ieu-b-102 | TRUE | reported | 934dMV | 500199 | 6.58E-05 | 32.93748645 |
| 2 | 1 | 49675276 | G | C | -0.0449 | 0.0075 | 1.75E-09 | 0.0923 | rs354155 | ieu-b-102 | TRUE | reported | 934dMV | 500199 | 7.16E-05 | 35.84003447 |
| 3 | 1 | 52274078 | T | G | 0.0283 | 0.0043 | 5.11E-11 | 0.5329 | rs7551758 | ieu-b-102 | TRUE | reported | 934dMV | 500199 | 8.66E-05 | 43.31459155 |
| 4 | 1 | 67132262 | T | C | 0.0251 | 0.0043 | 7.29E-09 | 0.5599 | rs7538938 | ieu-b-102 | TRUE | reported | 934dMV | 500199 | 6.81E-05 | 34.0728762 |
| 5 | 1 | 72765116 | G | A | 0.0382 | 0.0044 | 2.90E-18 | 0.6042 | rs2568958 | ieu-b-102 | TRUE | reported | 934dMV | 500199 | 1.51E-04 | 75.37366557 |
| 6 | 1 | 175913828 | C | T | -0.0262 | 0.0045 | 4.53E-09 | 0.378 | rs10913112 | ieu-b-102 | TRUE | reported | 934dMV | 500199 | 6.78E-05 | 33.89813607 |
| 7 | 1 | 197704717 | C | T | -0.03 | 0.0053 | 1.50E-08 | 0.2101 | rs17641524 | ieu-b-102 | TRUE | reported | 934dMV | 500199 | 6.41E-05 | 32.03974373 |
| 8 | 2 | 208049581 | G | A | 0.0263 | 0.0046 | 1.35E-08 | 0.3141 | rs2111592 | ieu-b-102 | TRUE | reported | 934dMV | 500199 | 6.53E-05 | 32.68843262 |
| 9 | 2 | 212618440 | G | A | 0.0265 | 0.0047 | 1.71E-08 | 0.2975 | rs72948506 | ieu-b-102 | TRUE | reported | 934dMV | 500199 | 6.36E-05 | 31.79027579 |
| 10 | 3 | 49214303 | G | T | -0.0292 | 0.0052 | 1.59E-08 | 0.7739 | rs9831648 | ieu-b-102 | TRUE | reported | 934dMV | 500199 | 6.30E-05 | 31.5324183 |
| 11 | 3 | 61255413 | G | A | 0.0248 | 0.0044 | 1.41E-08 | 0.4117 | rs843812 | ieu-b-102 | TRUE | reported | 934dMV | 500199 | 6.35E-05 | 31.76846802 |
| 12 | 3 | 115977242 | T | A | 0.0412 | 0.0074 | 2.41E-08 | 0.0931 | rs76954012 | ieu-b-102 | TRUE | reported | 934dMV | 500199 | 6.20E-05 | 30.99768468 |
| 13 | 3 | 117515519 | T | C | 0.0297 | 0.0048 | 6.03E-10 | 0.284 | rs66511648 | ieu-b-102 | TRUE | reported | 934dMV | 500199 | 7.65E-05 | 38.28500317 |
| 14 | 3 | 158171455 | A | G | -0.0241 | 0.0044 | 3.28E-08 | 0.5774 | rs35469634 | ieu-b-102 | TRUE | reported | 934dMV | 500199 | 6.00E-05 | 30.00039657 |
| 15 | 5 | 87630769 | A | G | 0.0237 | 0.0043 | 4.71E-08 | 0.457 | rs247910 | ieu-b-102 | TRUE | reported | 934dMV | 500199 | 6.07E-05 | 30.37792072 |
| 16 | 5 | 103972357 | G | A | 0.0366 | 0.0046 | 1.43E-15 | 0.3271 | rs30266 | ieu-b-102 | TRUE | reported | 934dMV | 500199 | 1.27E-04 | 63.30598506 |
| 17 | 5 | 164487555 | G | A | 0.029 | 0.0043 | 1.61E-11 | 0.5343 | rs7725715 | ieu-b-102 | TRUE | reported | 934dMV | 500199 | 9.09E-05 | 45.48386357 |
| 18 | 6 | 27182377 | A | C | 0.0704 | 0.012 | 4.51E-09 | 0.0327 | rs150186873 | ieu-b-102 | TRUE | reported | 934dMV | 500199 | 6.88E-05 | 34.41764016 |
| 19 | 6 | 28366151 | A | G | -0.062 | 0.007 | 1.14E-18 | 0.1056 | rs2232423 | ieu-b-102 | TRUE | reported | 934dMV | 500199 | 1.57E-04 | 78.44866592 |
| 20 | 6 | 67000001 | A | G | -0.0261 | 0.0045 | 8.56E-09 | 0.6466 | rs2214123 | ieu-b-102 | TRUE | reported | 934dMV | 500199 | 6.72E-05 | 33.63986549 |
| 21 | 6 | 142996618 | C | G | 0.026 | 0.0043 | 2.24E-09 | 0.4688 | rs2876520 | ieu-b-102 | TRUE | reported | 934dMV | 500199 | 7.31E-05 | 36.56015668 |
| 22 | 6 | 165117329 | A | G | 0.0283 | 0.0051 | 3.49E-08 | 0.2262 | rs9364755 | ieu-b-102 | TRUE | reported | 934dMV | 500199 | 6.16E-05 | 30.79149549 |
| 23 | 7 | 2086814 | T | C | -0.027 | 0.0049 | 4.68E-08 | 0.2529 | rs10235664 | ieu-b-102 | TRUE | reported | 934dMV | 500199 | 6.07E-05 | 30.36222762 |
| 24 | 7 | 12250402 | G | A | 0.031 | 0.0044 | 1.09E-12 | 0.4105 | rs3807865 | ieu-b-102 | TRUE | reported | 934dMV | 500199 | 9.92E-05 | 49.63823128 |
| 25 | 7 | 38724868 | C | T | 0.0363 | 0.0066 | 3.07E-08 | 0.1342 | rs59082935 | ieu-b-102 | TRUE | reported | 934dMV | 500199 | 6.05E-05 | 30.24987905 |
| 26 | 7 | 82448100 | T | C | 0.024 | 0.0043 | 2.11E-08 | 0.4739 | rs2522831 | ieu-b-102 | TRUE | reported | 934dMV | 500199 | 6.23E-05 | 31.15184948 |
| 27 | 7 | 109100414 | T | A | 0.0238 | 0.0043 | 4.12E-08 | 0.4659 | rs4730387 | ieu-b-102 | TRUE | reported | 934dMV | 500199 | 6.12E-05 | 30.63481531 |
| 28 | 7 | 117625599 | C | T | 0.0283 | 0.0044 | 1.16E-10 | 0.4118 | rs150346963 | ieu-b-102 | TRUE | reported | 934dMV | 500199 | 8.27E-05 | 41.36811972 |
| 29 | 9 | 11203149 | A | G | -0.0295 | 0.0044 | 1.68E-11 | 0.4042 | rs1931388 | ieu-b-102 | TRUE | reported | 934dMV | 500199 | 8.99E-05 | 44.95075002 |
| 30 | 9 | 25232978 | G | A | -0.039 | 0.007 | 2.41E-08 | 0.1081 | rs59283172 | ieu-b-102 | TRUE | reported | 934dMV | 500199 | 6.21E-05 | 31.04069221 |
| 31 | 9 | 37182655 | G | A | 0.0339 | 0.0058 | 4.69E-09 | 0.1639 | rs62535714 | ieu-b-102 | TRUE | reported | 934dMV | 500199 | 6.83E-05 | 34.16187292 |
| 32 | 9 | 119731359 | T | C | -0.0281 | 0.0048 | 4.25E-09 | 0.281 | rs2418449 | ieu-b-102 | TRUE | reported | 934dMV | 500199 | 6.85E-05 | 34.27113033 |
| 33 | 10 | 106610839 | A | G | -0.03 | 0.0045 | 2.29E-11 | 0.6434 | rs1021363 | ieu-b-102 | TRUE | reported | 934dMV | 500199 | 8.88E-05 | 44.44426674 |
| 34 | 11 | 61471678 | C | T | -0.0315 | 0.0056 | 1.90E-08 | 0.1886 | rs198457 | ieu-b-102 | TRUE | reported | 934dMV | 500199 | 6.33E-05 | 31.64049849 |
| 35 | 11 | 88756779 | T | C | 0.0291 | 0.0044 | 2.93E-11 | 0.44 | rs4497414 | ieu-b-102 | TRUE | reported | 934dMV | 500199 | 8.74E-05 | 43.74001106 |
| 36 | 11 | 113365141 | G | C | 0.0278 | 0.0044 | 3.57E-10 | 0.622 | rs4936276 | ieu-b-102 | TRUE | reported | 934dMV | 500199 | 7.98E-05 | 39.91926187 |
| 37 | 12 | 52352301 | G | A | 0.0309 | 0.0054 | 7.96E-09 | 0.2034 | rs61914045 | ieu-b-102 | TRUE | reported | 934dMV | 500199 | 6.55E-05 | 32.74369624 |
| 38 | 13 | 31790053 | C | T | -0.034 | 0.0054 | 2.23E-10 | 0.2031 | rs9529218 | ieu-b-102 | TRUE | reported | 934dMV | 500199 | 7.92E-05 | 39.64318854 |
| 39 | 13 | 53860655 | C | T | 0.0255 | 0.0046 | 2.62E-08 | 0.3259 | rs9536381 | ieu-b-102 | TRUE | reported | 934dMV | 500199 | 6.14E-05 | 30.73002836 |
| 40 | 13 | 80921519 | C | T | -0.0264 | 0.0048 | 3.56E-08 | 0.2992 | rs508502 | ieu-b-102 | TRUE | reported | 934dMV | 500199 | 6.05E-05 | 30.24987905 |
| 41 | 14 | 42097937 | A | G | -0.0297 | 0.0043 | 4.74E-12 | 0.5173 | rs1950829 | ieu-b-102 | TRUE | reported | 934dMV | 500199 | 9.54E-05 | 47.706137 |
| 42 | 14 | 75125540 | T | C | 0.0258 | 0.0043 | 1.87E-09 | 0.5196 | rs7152906 | ieu-b-102 | TRUE | reported | 934dMV | 500199 | 7.20E-05 | 35.99985606 |
| 43 | 14 | 103997525 | T | A | -0.0289 | 0.0045 | 1.31E-10 | 0.3664 | rs754287 | ieu-b-102 | TRUE | reported | 934dMV | 500199 | 8.25E-05 | 41.24477336 |
| 44 | 15 | 88945878 | C | G | -0.0292 | 0.0052 | 1.76E-08 | 0.2308 | rs28541419 | ieu-b-102 | TRUE | reported | 934dMV | 500199 | 6.30E-05 | 31.5324183 |
| 45 | 16 | 13800430 | G | C | 0.0327 | 0.0055 | 3.09E-09 | 0.1884 | rs12919291 | ieu-b-102 | TRUE | reported | 934dMV | 500199 | 7.07E-05 | 35.34828841 |
| 46 | 18 | 35155910 | C | T | -0.0292 | 0.0046 | 1.40E-10 | 0.6684 | rs4799949 | ieu-b-102 | TRUE | reported | 934dMV | 500199 | 8.06E-05 | 40.29473491 |
| 47 | 18 | 50861409 | T | C | 0.0253 | 0.0043 | 4.35E-09 | 0.5148 | rs1367635 | ieu-b-102 | TRUE | reported | 934dMV | 500199 | 6.92E-05 | 34.61803357 |
| 48 | 18 | 53099012 | G | C | -0.0345 | 0.0047 | 2.53E-13 | 0.7012 | rs12967143 | ieu-b-102 | TRUE | reported | 934dMV | 500199 | 1.08E-04 | 53.88163155 |
| 49 | 18 | 77580712 | G | A | 0.0323 | 0.0054 | 2.43E-09 | 0.2047 | rs7241572 | ieu-b-102 | TRUE | reported | 934dMV | 500199 | 7.15E-05 | 35.77797766 |
| 50 | 20 | 44692598 | C | T | 0.031 | 0.0049 | 2.40E-10 | 0.2597 | rs13037326 | ieu-b-102 | TRUE | reported | 934dMV | 500199 | 8.00E-05 | 40.02482955 |
